# High performance organic solar cell enabled by manipulating the exciton dissociation and charge transfer via dielectric engineering

**DOI:** 10.1038/s41467-026-74112-x

**Published:** 2026-06-16

**Authors:** Yanan Wei, Xiaobin Gu, Xue Shi, Meng Zhang, Jikai Lv, Xubing Bai, Lixia Wang, Xin Zhang, Xiangyue Meng, Jianqi Zhang, Xiaotao Hao, Qian Peng, Yunhao Cai, Hui Huang

**Affiliations:** 1 College of Materials Science and Opto-Electronic Technology, University of Chinese Academy of Sciences, Beijing, P. R. China; 2 School of Physics, State Key Laboratory of Crystal Materials, Shandong University, Jinan, Shandong P. R. China; 3 Key Laboratory of Nanosystem and Hierarchical Fabrication, National Center for Nanoscience and Technology, Beijing, China; 4 University of the Chinese Academy of Sciences, School of Chemical Sciences, Beijing, P. R. China; 5 School of Chemical Engineering and Technology, State Key Laboratory of Chemical Engineering and Low-Carbon Technology, Tianjin University, Tianjin, China; 6 The International Joint Institute of Tianjin University, Fuzhou, China

**Keywords:** Solar cells, Solar cells

## Abstract

As core processes determining the power conversion efficiency (PCE) of organic solar cells (OSCs), exciton dissociation and charge transfer are fundamentally restricted by the low intrinsic dielectric constant of organic semiconductors. Herein, two dielectric regulators (Drs) named Dr-1 and Dr-2 are judiciously designed with different molecular dipole moments to conduct research on dielectric engineering. The incorporation of S···F noncovalent conformational locks (NoCLs) endows Dr-2 with an extended π-conjugated backbone, improved molecular polarizability, reinforced charge delocalization and a larger dipole moment than Dr-1. Thus, Dr-2-modified OSCs based on the D18:L8-BO system achieve a PCE of 20.85%, surpassing the 20.13% of Dr-1-treated counterparts. Enhanced efficiencies across diverse donor-acceptor systems confirm the universal applicability of this strategy. Furthermore, 300 nm-thick OSCs incorporated with Dr-2 deliver a record-high PCE of 19.56%. This work provides a strategy for designing high-performance dielectric regulators via tuning molecular dipole moment and planarity simultaneously, thereby achieving high-efficiency OSCs.

## Abstract (web summary)

The intrinsically low dielectric constant of organic semiconductors limits their application in solar cells. Wei et al. address this challenge by modulating the dielectric constant using specially designed regulators with an extended π-conjugated backbone, achieving a power conversion efficiency of 20.85%.

## Introduction

As an emerging sustainable technology for clean energy, organic solar cells (OSCs) have recently attracted much interest due to their unique characteristics, including light weight, low cost, and flexibility^1,2^. Recent advances in molecular design^3–7^ and device engineering^8–10^ have propelled the power conversion efficiencies (PCEs) of state-of-the-art OSCs beyond 20%^11–18^. Despite these progresses, further performance enhancement is still restrained by many factors, including severe non-radiative energy loss, low dielectric constant and non-optimized morphology^19–22^. Among them, the intrinsically low dielectric constants (*ε*_r_) of organic semiconductors are critical^22^. The low *ε*_r_ arises from their molecular structures, which are typically composed of elements with low polarizability and dominated by covalent bonding. This leads to weak intermolecular interactions and minimal permanent dipole moments, resulting in the formation of tightly bound Frenkel excitons and polarons. Consequently, organic semiconductors often exhibit high exciton binding energies (*E*_b_), ranging from several hundred meV to over 1.4 eV^23^, which hinders efficient exciton dissociation and charge separation. Furthermore, the strong Coulombic interaction between electrons and holes increases the probability of geminate recombination, ultimately limiting charge collection and device performance^24–26^.

To overcome these limitations, considerable efforts have been devoted to enhancing the dielectric properties of organic semiconductors. These include the incorporation of highly polarizable atoms or dipolar functional groups to increase local polarizability, the extension of π-conjugated backbones to improve charge delocalization and molecular polarizability, and the introduction of a high-permittivity component into the active layer^10,27,28^. However, modifying the molecular structure of active layer materials inevitably alters their intrinsic optoelectronic and morphological characteristics, which can easily lead to adverse effects on device efficiency. Therefore, developing physical tools that can enhance the dielectric constant without compromising the chemical structures and electronic integrity of the active layer represents a more controllable and convenient approach.

Herein, we develop two dielectric regulators (Drs) to improve OSC efficiencies upon tuning the dielectric constants of the active layer. In comparison to Dr-1, Dr-2 possesses an extended π-conjugated backbone, enhanced molecular polarizability, and strengthened charge delocalization upon incorporating S···F noncovalent conformational locks (NoCLs). The experimental and theoretical results show that the incorporated Dr-2 in the active layer of D18:L8-BO effectively forms strong interactions with L8-BO molecules, thereby enhancing the crystallinity and the *ε*_r_ of the blend film, which in turn facilitates exciton dissociation, accelerates charge transport, and suppresses recombination losses. As a result, the efficiency of D18:L8-BO-based OSCs upon incorporation of Dr-2 is significantly enhanced to deliver a PCE of 20.85%, much higher than those treated with Dr-1. The universality of this approach is further demonstrated by consistent efficiency improvements across multiple donor-acceptor systems. In addition, the enhanced dielectric environment is highly favorable for fabricating thick-film devices, with the incorporation of Dr-2 yielding a record-high PCE of 19.56% at an active layer thickness of 300 nm. These findings underscore the potential of dielectric engineering as a general and scalable strategy for developing next-generation high-efficiency OSCs.

## Results

### Molecular design, synthesis and characterizations

**Fig. 1:**
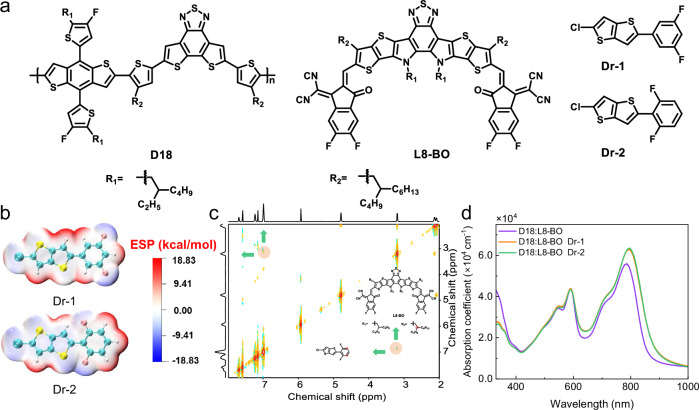
Chemical structures of materials and the related physical properties. **a** Chemical structures of donor, acceptor and dielectric regulator materials. **b** ESP of dielectric regulator materials. **c** 2D ^1^H-  ^1^H NMR spectra of L8-BO:Dr-2 in deuterated tetrachloroethane (d-TCE) solution. **d** Absorption coefficients of D18:L8-BO film with/without regulator materials.

Figure 1a shows the chemical structures of D18, L8-BO and dielectric regulators (Dr-1 and Dr-2). The molecular weight (*M*_n_) of D18 and its polydispersity index are 54.3 KDa and 2.18, respectively, which has been supplemented in the Supplementary Fig. 1. The synthetic details for Dr-1 and Dr-2 are shown in Supplementary Figs. 2, 3. The design rationale includes the incorporation of highly polarizable atoms or dipolar functional groups to increase local polarizability (e.g., halogen atoms), the extension of π-conjugated backbones to improve charge delocalization and molecular polarizability (e.g., S···F noncovalent conformational locks), and the introduction of a high-permittivity component into the active layer (as dielectric regulator). Their structures were fully characterized by ^1^H-/^13^C-NMR spectrometry and high-resolution mass spectral (HRMS) (Supplementary Figs. 4-13). The volatilities of dielectric regulators were investigated via thermogravimetric analysis. Dr-2 exhibits significantly better sublimation than Dr-1 when subjected to thermal annealing at 100 °C, which could be completely eliminated by extending the heating duration (Supplementary Fig. 14). Intramolecular noncovalent interactions were investigated via single-crystal X-ray diffraction (SCXRD) measurements. The crystal structure of Dr-2 is presented in Supplementary Fig. 15, with the corresponding detailed crystallographic data summarized in Supplementary Table 1. Dr-2 crystal is isostructural, belonging to the monoclinic system with space group P2_1_/_n_. At the equilibrium geometry, the intramolecular S···F distance is determined to be 2.85 Å, which is significantly shorter than the sum of the respective van der Waals radii for S and F (3.27 Å), confirming the existence of intramolecular S···F noncovalent conformational locks. The Dr-2 crystal forms a dimeric structure with two distinct stacking motifs via intermolecular π···π interactions, where the corresponding π···π stacking distances (dπ···π) are 3.59 Å and 3.47 Å for the two stacking modes, respectively (Supplementary Fig. 15). Additionally, the quantum chemistry calculations were performed by density functional theory (DFT) method, including the optimization of molecular geometries, natural bond orbitals, dipole moments, surface electrostatic potential (ESP)^29^ for the two dielectric regulators, D18 and L8-BO. Based on the optimized geometries, the descriptor (*S*) value, which quantifies the strength of NoCL, is calculated. The *S* of Dr-2 is 0.456 (larger than 0.14), suggesting the formation of S···F NoCLs^30^. The natural bond orbital analysis indicates that the S···F NoCLs arise from the overlap between the lone pair of the F atom and the antibonding orbital of the S-C bond, corresponding to an *E*^(2)^ value of -0.93 kcal mol^−1^ for the *n*(F)→σ*(S-C) donation^31^, as shown in Supplementary Fig. 16, Supplementary Table 2. Due to the polarization effect of fluorine atoms on the bithiophene through NoCLs, Dr-2 exhibits a significantly higher dipole moment (2.98 D) than Dr-1 (0.07 D) (Fig. 1b, Supplementary Fig. 17), which would decrease *E*_b_ and increase *ε*_r_ in its solid state^32^. As shown in Fig. 1b, Supplementary Fig. 18 and Supplementary Table 3, the D18 molecule has a relatively balanced distribution of positive and negative surfaces, while the positive surface area of the L8-BO molecule is much higher than the negative surface area. Therefore, Dr-2, with the largest negative surface area and the strongest average negative electrostatic potential, is most likely to bind with L8-BO through electrostatic interactions, while Dr-1, with the smallest negative surface area and the weakest average negative electrostatic potential, is the least likely to undergo such binding^33^.

To verify the intermolecular interactions, we employed two-dimensional (2D) liquid-state nuclear magnetic resonance (NMR) spectroscopy with higher resolution for characterization. Owing to the nuclear Overhauser effect, NOESY spectra in 2D NMR furnish information about protons that are ≤ 5 Å apart in space. In the 2D ^1^H - ^1^H NOESY spectra of L8-BO:Dr-1 and L8-BO:Dr-2 solutions, a distinct cross-peaks are clearly observed between the  protons (7.06 ppm for Dr-1 and 6.98 ppm for Dr-2) of the regulators and the  (3.11 ppm in the L8-BO:Dr-1) and  (3.18 ppm in the L8-BO:Dr-2) protons of L8-BO, which directly confirms the intermolecular interactions between L8-BO and the respective regulators (Fig. 1c, Supplementary Fig. 19). In addition, the intermolecular interaction between L8-BO and dielectric regulator was explored via Fourier-transform infrared spectroscopy (FTIR) analysis. The symmetrical stretching vibration (νs) peak of L8-BO exhibits a red shift from 2216 to 2215 and 2214 cm^‐1^ after being treated with Dr-1 and Dr-2, respectively, indicating the existence of intermolecular interaction between L8-BO and dielectric regulators (Supplementary Figs. 20, 21). The equilibrium systems of D18, L8-BO and the dielectric regulators were obtained based on molecular dynamics simulations, and the total Coulomb interaction energy (*E*_elec_) and total Van der Waals interaction energy (*E*_vdW_) between dielectric regulators and donor (or acceptor) molecules were calculated^34^. Obviously, Dr-2 shows a stronger electrostatic attraction with L8-BO than Dr-1 (Supplementary Table 4). In addition, Dr-2 shows stronger van der Waals interactions with the active layer than Dr-1, as its smaller twist angle facilitates insertion into donor and acceptor molecules and regulates molecular stacking. Dr-2, with a smaller twist angle than Dr-1, is more conducive to insertion into donor and acceptor molecules to exhibit strong van der Waals interactions with the active layer materials^35^. Compared with the UV-vis absorption spectra of D18:L8-BO film, dielectric regulator-treated blend films show red shifts of 2 nm (Dr-1) and 3 nm (Dr-2) at 500-600 nm range, and 6 nm (Dr-1) and 11 nm (Dr-2) at 700-900 nm range, respectively, indicating that Dr-2 was more effective than Dr-1 in enhancing D18:L8-BO’s ordered stacking configuration (Supplementary Fig. 22, Supplementary Table 5). The dielectric regulators don’t exhibit absorption peaks in the visible region, thus do not cause parasitic light absorption in the active layer (Supplementary Fig. 23). It is observed that with the deal with both Dr-1 and Dr-2, the absorption coefficient of D18:L8-BO blend is slightly increased, which is beneficial to the light harvesting (Fig. 1d)^36^.

### Device fabrication

**Fig. 2:**
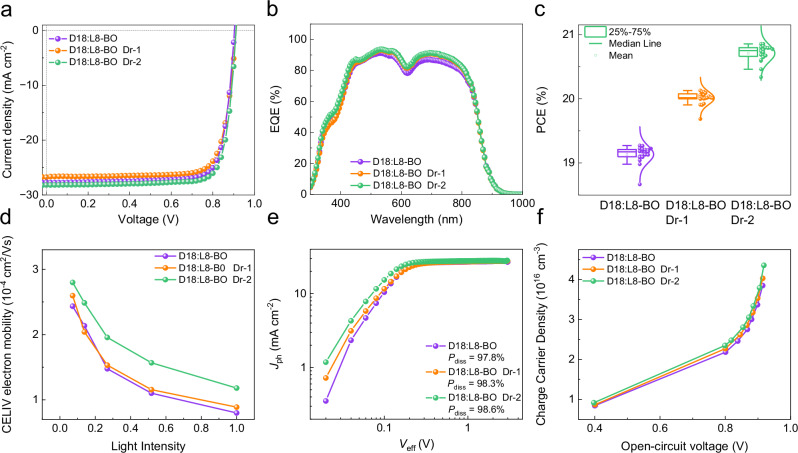
Photovoltaic performance. **a** *J*-*V*, **b** EQE curves and **c** box chart of the PCE measurements for 20 devices of D18:L8-BO with/without dielectric regulators. **d** Electron mobility of devices from Photo-CELIV measurement. **e** *J*_ph_ - *V*_eff_ curves of devices. **f** Charge carrier density (*n*) as a function of the open-circuit voltage of devices.

Furthermore, OSCs with a conventional architecture of indium tin oxides (ITO)/[2-(9*H*-Carbazol-9-yl)ethyl] phosphonic acid (2PACz)/active layer/PNDIT-F3N/Ag were fabricated to study the dielectric regulators’ effect on the performance of corresponding devices. For the D18:L8-BO binary device, a 19.3% PCE is obtained without the dielectric regulator, similar to the reported values (Fig. 2a-c, Table 1)^15^. Then, different amounts of dielectric regulators are added into the binary blend to optimize the device performances (Supplementary Fig. 24, Supplementary Table 6). The D18:L8-BO with 20 wt% Dr-1 based device produces a PCE of 20.1%, with a *V*_oc_ of 0.903 V, a *J*_sc_ of 27.69 mA cm^-2^, and an FF of 80.50%. While the blend with 20 wt% of Dr-2 based devices yields an extraordinary PCE of 20.85%, with a *V*_oc_ of 0.912 V, a *J*_sc_ of 28.18 mA cm^-2^, and an FF of 81.14%, which is one of the highest efficiencies reported for binary OSCs to date. The champion device achieves a certified PCE of 20.38% following the calibration procedures of the South China National Center of Metrology (Supplementary Fig. 25). The external quantum efficiency (EQE) curves of the devices are shown in Fig. 2b. The integrated *J*_sc_ values obtained from the EQE spectra for all the devices are consistent with the trends observed in the *J*-*V* measurements. High IQE values over 95% in the wavelength range of 400-600 nm can be observed, suggesting that high percentage of absorbed photons resulted in efficient electron collection (Supplementary Fig. 26). The Dr-2-treated D18:L8-BO device exhibits a reduced reverse saturation current density, which helps minimize parasitic current under dark conditions and thus enhances the device’s photoelectric response efficiency (Supplementary Fig. 27). Characterization via maximum power point (MPP) tracking  demonstrates that device photostability is enhanced upon incorporation of dielectric regulators. A 20% PCE loss is observed at 350 h and 500 h for the D18:L8-BO and D18:L8-BO Dr-1 devices under simulated AM1.5 G irradiation (100 mW cm^-2^) (Supplementary Fig. 28). By stark contrast, the Dr-2-treated device preserved 82.1% of its initial efficiency after 1000 h of testing. We further apply Dr-2 into the fabrication of a thick-film device (300 nm), D18:L8-BO based OSC obtains a high PCE of 19.56%, with a *V*_oc_ of 0.887 V, a *J*_sc_ of 28.22 mA cm^-2^, and an FF of 78.16% (Supplementary Fig. 29, Supplementary Table 7).To the best of our knowledge, such an efficiency represents the highest value reported for thick-film OSC.

**Table 1:** Photovoltaic performance parameters of devices

Active Layer D18:L8-BO	*V*_oc_ (V)	*J*_sc_ (mA cm^-2^)	FF (%)	PCE^a^ (%)
none	0.911 (0.911 ± 0.001)	26.75 (26.62 ± 0.13)	79.08 (78.89 ± 0.19)	19.27 (19.13 ± 0.13)
Dr-1	0.903 (0.902 ± 0.001)	27.69 (27.57 ± 0.12)	80.50 (80.44 ± 0.06)	20.13 (20.02 ± 0.10)
Dr-2	0.912 (0.912 ± 0.001)	28.18 (28.01 ± 0.17)	81.14 (81.04 ± 0.10)	20.85 (20.71 ± 0.13)

^a^The average parameters were calculated based on 20 independent cells.

The space charge-limited-current (SCLC) measurement was conducted to analyse the hole mobilities (*μ*_h_) and electron mobilities (*μ*_e_) of devices treated with/without the dielectric regulators. The *μ*_h_ and *μ*_e_ are calculated to be 2.89 × 10^-4^ and 3.49 × 10^-4^ cm^2^ V^-1^ s^-1^, 3.56 × 10^-4^ and 3.04 × 10^-4 ^cm^2^ V^-1^ s^-1^, and 5.22 × 10^-4^ and 4.82 × 10^-4^ cm^2^ V^-1^ s^-1^ for D18:L8-BO, D18:L8-BO (Dr-1), and D18:L8-BO (Dr-2) systems, respectively (Supplementary Fig. 30, Supplementary Table 8). Obviously, the Dr-2-treated device demonstrates a more balanced charge transport with a *μ*_h_/*μ*_e_ of 1.08, lower than those of D18:L8-BO and Dr-1-treated devices (1.21 and 1.17). The enhanced and more balanced mobility is beneficial to charge transport and collections, resulting in higher *J*_sc_ and FF in the Dr-2-treated devices.

Furthermore, transient photovoltage (TPV) decay kinetics measurements were employed to determine the charge carrier lifetime and quantify the charge recombination processes within the devices. The charge recombination lifetime (*τ*_rec_) of the D18:L8-BO Dr-2 device is 797.1 µs, which is longer than that of the pristine D18:L8-BO device (540.7 µs) and the D18:L8-BO Dr-1 device (675.2 µs), indicating a reduced charge recombination propensity in the Dr-2-treated device (Supplementary Fig. 31a). Transient photocurrent (TPC) decay measurements were conducted to investigate the charge extraction characteristics of the devices. The D18:L8-BO Dr-2 device exhibits a shorter charge extraction time (*τ*_ext_ = 0.31 µs) than the pristine D18:L8-BO (0.46 µs) and Dr-1 (0.39 µs) counterparts, indicative of fast and efficient charge extraction for the Dr-2-processed device (Supplementary Fig. 31b).

In addition, photo-generated charge extraction by linearly increasing voltage (photo-CELIV) measurements were conducted to study the charge transport inside the devices. In this technique, charge carriers are generated under a short laser flash. After an adjustable delay, the photo-generated charge carriers are extracted under a reverse bias voltage ramp^37^. According to the Eq. (1), the mobility (*μ*) of charge carriers can be obtained.

1 $$\mu=\frac{2{d}^{2}}{3A{t}_{\max }^{2}[1+0.36\frac{\bigtriangleup j}{j(0)}]},{if}\bigtriangleup j\le j(0)$$

where *t*_max_ is the time of maximum current density, *A* is the voltage rise speed of the applied voltage pulse, and *j*(o) is capacitive displacement current^38^. Supplementary Fig. 32 shows the photo-CELIV curve for devices with/without the dielectric regulators. The extraction current increases as the light intensity improves. With the addition of Dr-1/Dr-2 into the active layer, the mobility of charge carriers is improved when compared with D18:L8-BO without the dielectric regulators. Moreover, the Dr-2-treated D18:L8-BO device shows the highest mobility of charge carriers among the devices, which facilitates efficient charge transport (Fig. 2d).

The exciton dissociation processes in the devices were investigated via photocurrent density (*J*_ph_ = *J*_L_ − *J*_D_) versus effective voltage (*V*_eff_ = *V*_0_ − *V*_a_) curves (Fig. 2e)^39^. According to *J*_ph_/*J*_sat_, the exciton dissociation under the short-circuit (*P*_diss_) condition of devices are 97.8%, 98.3% and 98.6% for D18:L8-BO, D18:L8-BO (Dr-1), and D18:L8-BO (Dr-2) devices, respectively. The superior *P*_diss_ of Dr-2-treated D18:L8-BO device clarifies more efficient exciton dissociation. For the charge recombination kinetics, the curves of *V*_oc_ versus *P*_light_ can be described by the equation of *V*_oc_∝ *n*(*kT*/*q*)ln(*P*_light_), where *k* is the Boltzmann constant, *T* is the Kelvin temperature, and *q* is the elementary charge. If trap-assisted recombination is involved, a stronger dependency of *V*_oc_ on the light intensity (>*k*T/*q*) will be achieved^40,41^. The D18:L8-BO, D18:L8-BO (Dr-1) and D18:L8-BO (Dr-2) devices show the slopes of 1.28 *k*T/*q*, 1.23 *k*T/*q* and 1.19 *k*T/*q*, respectively, indicating the most effective suppression of trap-assistant recombination with the assistance of Dr-2 into the blend (Supplementary Fig. 33a). For the bimolecular recombination dynamics, the *J*_sc_ possessed a linear correlation with *P*_light_ (*J*_sc_ ∝ *P*_light_^*α*^), where *α* is the slope of the curve^42^. The D18:L8-BO (Dr-2) device with *α* value of 0.992 shows the highest slope value among all the devices, suggesting the most effective suppression of bimolecular recombination after the treatment of Dr-2 (Supplementary Fig. 33b). For thick film devices, the D18:L8-BO, D18:L8-BO Dr-1 and D18:L8-BO Dr-2 devices show the slopes of 1.38, 1.33 and 1.26 of charge recombination kinetics, indicating effective suppression of trap-assistant recombination with the assistance of regulators (Supplementary Fig. 34a). For the bimolecular recombination dynamics, the D18:L8-BO Dr-2 device with *α* value of 0.991 is the higher than those D18:L8-BO and D18:L8-BO Dr-1 devices, suggesting the effective mitigation of bimolecular recombination after the treat of Dr-2 (Supplementary Fig. 34b). Thus, both the trap-assisted recombination and the bimolecular recombination can be effectively alleviated with the assistance of the planar dielectric regulator (Dr-2).

The correlation of charge carrier density with voltage was investigated through charge extraction measurements. In the bias-assisted charge extraction measurement, the induced extraction current transient under various illumination at corresponding open-circuit condition for different devices are shown in Supplementary Fig. 35. The charge carrier density (*n*_CE_) can be calculated according to the correlation as follow:

2 $$n_{{{\rm{CE}}}}=\frac{1}{{dq}}\left(\int^{{t}_{\infty }}_{\!\!\!\!\!0}j\left(t\right){{{\rm{d}}}}t-{C}_{0}{V}_{{oc}}\right)$$

where *d* is the thickness of device, *q* is elementary charge, *t*_∞_ is the time when all excess charge carriers have been collected (and/or recombined), and *C*_0_*V*_oc_ corresponds to the charge stored on the electrodes. Under the treatment of Dr-2, the corresponding device displays the highest charge carrier density among all the devices, which is beneficial for the generation of photocurrent (Fig. 2f).

### Mechanistic studies and film formation dynamics

**Fig. 3:**
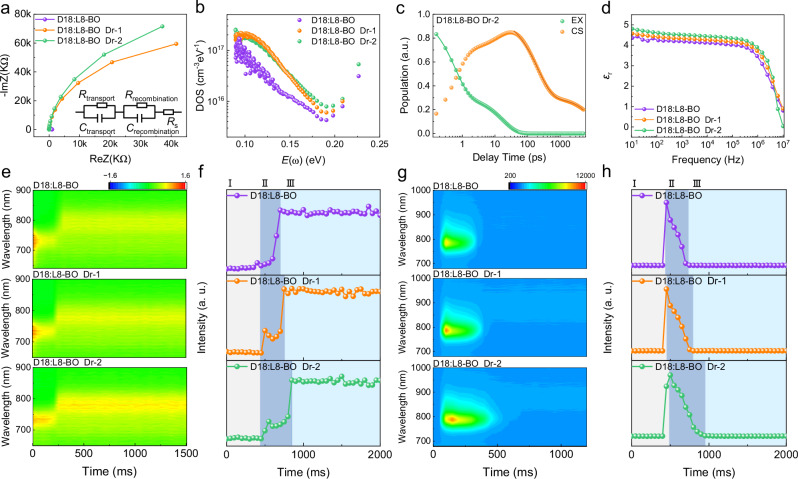
Mechanistic studies and film formation dynamics. **a** The Nyquist plots, **b** defects density of state, **c** kinetics of EX and CS spectra extracted from TA spectra via SVD for Dr-2-treated D18:L8-BO blend films, and **d** dielectric constants of D18:L8-BO, D18:L8-BO (treated with Dr-1) and D18:L8-BO (treated with Dr-2) films, respectively. **e** In situ UV–vis absorption spectra of D18:L8-BO, D18:L8-BO (Dr-1) and D18:L8-BO (Dr-2) deposited on ITO from solution states to film states. **f** The peak intensity evolution as a function of time of the relevant process in (**e**). **g** In situ PL spectra of D18:L8-BO, D18:L8-BO (Dr-1) and D18:L8-BO (Dr-2) deposited on ITO from solution states to film states. **h** The peak intensity evolution as a function of time of the relevant process in (**g**).

The effect of dielectric regulators on the carrier transport of OSCs was further explored by electrochemical impedance spectroscopy measurement, as demonstrated in Fig. 3a, Supplementary Fig. 36 and Supplementary Table 9. The Nyquist plot is divided into three regions including high, middle and low frequency arcs, which are usually overlapped. The series resistance *R*_s_ is the system resistance due to the contacts and wires, which is almost unchanged among all the devices. The charge transport resistances (*R*_transport_) and recombination resistance (*R*_recombination_) are 22.78 Ω and 940 KΩ, 21.45 Ω and 1.20 × 10^5^ KΩ, 17.12 and 1.66 × 10^5^ KΩ for D18:L8-BO, D18:L8-BO (Dr-1) and D18:L8-BO (Dr-2), respectively. The reduction of *R*_transport_ and improved *R*_recombination_ of devices via Dr-2 are beneficial for carrier transport and for suppressing charge recombination, thereby improving the FF of the corresponding devices^43^.

Additionally, the frequency-dependent capacitance spectra can probe the density of the trap state of devices under a dark environment. The built-in potential of devices can be acquired via Mott-Schottky characterization, and thus achieving the transfer from capacitance to density of states (DOS) (Fig. 3b, Supplementary Fig. 37). Thermal activated hopping allows carriers to transport from one localized site to its neighbor rather than band transport for the long-range disorder, whcih results in a widespread Gaussian shape with the equation of ${N}_{{\mbox{t}}}(E)=\frac{{N}_{{\mbox{t}}}}{\sqrt{2\pi }\delta }\exp [-\frac{{({E}_{{\mbox{t}}}-E)}^{2}}{2{\delta }^{2}}]$, where *N*_t_ is the total density, *E*_t_ is the center of the DoS, and *δ* is disorder parameter^44^. As shown in Supplementary Table 10, the D18:L8-BO, D18:L8-BO (Dr-1) and D18:L8-BO (Dr-2) systems exhibit disorder values of 0.090, 0.043 and 0.035, corresponding to trap DoS of 5.52 × 10^16^ cm^-3^ eV^-1^, 3.47 × 10^16^ cm^-3^ eV^-1^ and 1.99 × 10^16^ cm^-3^ eV^-1^, respectively. Thus, the improved molecular energy distribution order and suppressed trap density in the Dr-2-treated device are instrumental in enhancing carrier transport.

To elucidate the electron transfer and recombination kinetics, transient absorption spectroscopy (TAS) was utilized to probe the excited-state dynamics of D18:L8-BO films with and without dielectric regulator modification^45,46^. The 2D TA color plots and the corresponding TA spectra recorded at different time delays are presented in Supplementary Figs. 38, 39. Exciton (EX) dissociation and charge-separated (CS) states in D18:L8-BO blend films were investigated via the analysis using singular value decomposition (SVD) and global fitting algorithms (Fig. 3c, Supplementary Fig. 40 and Supplementary Table 11). The decay of the EX state is ascribed to rapid hole injection and concurrent exciton dissociation. The Dr-2-treated D18:L8-BO film exhibits a faster EX state decay lifetime (17.08 ps) than the pristine D18:L8-BO film (17.21 ps) and Dr-1-modified D18:L8-BO film (17.19 ps), which indicates accelerated hole injection and concomitant exciton dissociation enabled by Dr-2. The CS reaches its maximum concentration at 30 ps, after which a slow recombination process occurs. The Dr-2-treated D18:L8-BO film shows an extended CS state decay lifetime (244.46 ns) compared to the pristine D18:L8-BO film (213.52 ns) and the Dr-1-modified D18:L8-BO film (236.66 ns), a result that facilitates the suppression of charge recombination via the modulation of Dr-2. Therefore, the enhanced photovoltaic performance of the corresponding OSC devices primarily originates from the accelerated hole injection, efficient exciton dissociation, and suppressed charge recombination in the Dr-2-treated D18:L8-BO film.

In addition, the energy transfer and charge transfer process in films were probed via steady-state photoluminescence (PL) spectroscopy and time-resolved photoluminescence (TRPL) spectroscopy (Supplementary Figs. 41, 42). The D18 and L8-BO films exhibit distinct fluorescence, with emission peaks observed at 690 and 890 nm, respectively. While the fluorescence intensities are quenched in the blend films. Meanwhile, the *τ* value of D18:L8-BO blend treated with Dr-2 shows the shortest lifetime (68.9 ps) among all the films. The efficient charge transfer revealed by the shortened *τ* value can be attributed to the improved molecular packing and more continuous charge transport pathways induced by Dr-2. This structural optimization with the synergistic effect of molecular dipole moment and planarity reduces trap-assisted recombination and facilitates charge collection.

Efficient exciton dissociation relies on a reduced exciton binding energy (*E*_b_), which facilitates the separation of the electron-hole pair into free charge carriers. Meanwhile, effective charge transfer requires suppressed recombination losses^47^. Both processes are strongly influenced by the dielectric properties of the active layer materials. The exciton binding energy in organic semiconductors is closely related to their intrinsic dielectric constant and can be expressed as Eq. (3):

3 $${E}_{{\mbox{b}}}=\frac{{q}^{2}}{4\pi {\varepsilon }_{{\mbox{r}}}{\varepsilon }_{0}r}$$

where *q* is the elementary charge, *ε*_0_ is the dielectric constant of vacuum, *ε*_r_ is the relative dielectric constant of the material, and *r* is the distance between the electron and hole^25,48,49^. To obtain the exciton binding energy, temperature dependent PL spectra for D18:L8-BO, D18:L8-BO (treated with Dr-1) and D18:L8-BO (treated with Dr-2) films are shown in Supplementary Fig. 43. The temperature dependence of integrated PL intensity *I*(T) can be explained by Eq. (4), where *E*_b_ is the excitonic binding energy, *k*_B_ is the Boltzmann constant, PL intensity at 0 K is *I*_0_, and *B* is the preexponential factor.

4 $$I(T)=\frac{{I}_{0}}{1+B{e}^{-{E}_{b}/({k}_{B}T)}}$$

Thus, exciton binding energies of D18:L8-BO, D18:L8-BO (treated with Dr-1) and D18:L8-BO (treated with Dr-2) films are 128, 111 and 102 mV, respectively (Supplementary Fig. 44). Upon the handling of the dielectric regulator, the exciton binding energy is reduced. Significantly, D18:L8-BO (treated with Dr-2) film presents the smallest exciton binding energy among the three films, which is beneficial for the exciton dissociation process inside the film.

Simultaneously, charge recombination is governed by Coulomb interactions, and the bimolecular recombination rate (*R*) can be approximated by Eq. (5)^26^:

5 $${{{\rm{Recomb}}}}.{{{\rm{Rate}}}}=\frac{{{{\rm{q}}}}{{{\rm{\mu }}}}}{{{{{\rm{\varepsilon }}}}}_{0}{{{{\rm{\varepsilon }}}}}_{{{{\rm{r}}}}}}$$

Assuming *r* and *μ* remain unchanged, increasing *ε*_r_ will reduce the binding energy of CT excitons and recombination rate^22,50^. The exciton dissociation and charge recombination processes in OSCs are key to determining the photocurrent generation and FF^51^. As shown in Fig. 3d, the dielectric constant values of D18:L8-BO, D18:L8-BO (Dr-1), and D18:L8-BO (Dr-2) are 4.36, 4.57 and 4.81, respectively. The noticeable increase in dielectric constant induced by Dr-2 highlights its strong dielectric regulation capability. Thus, the enhanced dielectric environment facilitates exciton dissociation and charge extraction while suppressing recombination losses.

The morphological evolution from the solution to films was investigated via in situ UV-vis absorption spectra and in situ PL spectra (Fig. 3e-h, Supplementary Figs. 45, 46). The crystallization process can be divided into three stages: solvent evaporation stage, crystal growth stage, and dried film stage^52^. Noticeably, the D18:L8-BO, D18:L8-BO (Dr-1) and D18:L8-BO (Dr-2) need 250, 300 and 400 ms for nucleation and crystal growth stage (Fig. 3f), consistent with the results of in situ PL spectra (Fig. 3g). Consistently, the results in Fig. 3h exhibit extended crystallization time of D18:L8-BO with the aid of Dr-2. Rapid crystallization can cause excessive aggregation of materials, which is not conducive to the stacking and arrangement of molecules^52,53^.With the aid of Dr-2, the D18:L8-BO film undergoes an optimized crystallization process, which promotes favorable molecular packing. This, in turn, enables effective modulation of the dielectric constant and facilitates charge transfer in devices. These findings highlight the intrinsic link between dielectric modulation capability and morphological evolution.

### Morphological analysis and molecular dynamics (MD) simulations

**Fig. 4:**
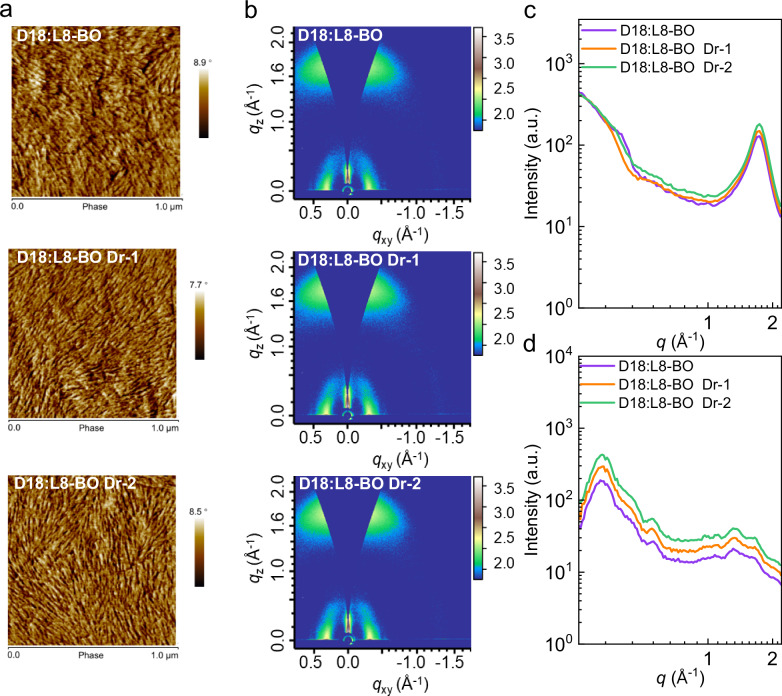
Morphology characterizations. **a** AFM phase images, **b** 2D GIWAXS patterns, **c** OOP and **d** IP extracted line-cut profiles of the corresponding blend films.

Atomic force microscopy (AFM), transmission electron microscope (TEM) and two-dimensional grazing-incidence wide-angle X-ray scattering (GIWAXS) were further carried out to understand the differences in films processed by different methods. AFM height and phase images of blend films are shown in Supplementary Fig. 47, Fig. 4a. Uniform and fibrillar morphologies are observed in all of the blend films, which is beneficial for the charge transport with the assistance of dielectric regulators. The morphology of the D18:L8-BO film shows better dispersity and improved root mean square value (RMS) (from 1.50 nm to 1.54 nm and 1.60 nm, respectively). TEM characterization of the blend films (Supplementary Fig. 48) reveals that the D18:L8-BO (Dr-2) film exhibits a fibrillar morphology, while the pristine D18:L8-BO film with excessive aggregation and the D18:L8-BO (Dr-1) film with some particulate aggregates. This fibrillar structure is favorable for exciton dissociation and facilitates charge transport within the film. GIWAXS measurements of the neat and blend films are presented in Fig. 4b, Supplementary Fig. 49, with the corresponding scattering profiles presented in Supplementary Fig. 50, Fig. 4c, d and Supplementary Tables 12-15. For the neat D18 and L8-BO films, in-plane (100) diffraction peaks appear at *q*_xy_ = 0.30 Å^-1^ (*d* ~ 20.94 Å) and *q*_xy_ = 0.43 Å^-1^ (*d* ~ 14.61 Å), respectively, while both films exhibit the same OOP (010) π-π stacking peak at *q*_z_ = 1.74 Å^-1^ (*d* ~ 3.61 Å), respectively. For the D18:L8-BO, D18:L8-BO (Dr-1) and D18:L8-BO (Dr-2) films, the IP diffraction peaks are observed at *q*_xy_ = 0.32 Å^-1^ (*d* ~ 19.63 Å), *q*_xy_ = 0.33 Å^-1^ (*d* ~ 19.03 Å) and *q*_xy_ = 0.33 Å^-1^ (*d* ~ 19.03 Å), corresponding to crystal coherence length (CCL) of 63.47, 65.45 and 69.05 Å, respectively. Simultaneously, the OOP diffraction peaks are located at *q*_z_ = 1.74 Å^-1^(*d* ~ 3.61 Å), *q*_z_ = 1.74 Å^-1^ (*d* ~ 3.61 Å) and *q*_z_ = 1.75 Å^-1^ (*d* ~ 3.59 Å), corresponding to CCLs of 22.44, 24.17 and 25.13 Å, respectively. The reduced stacking distance and enhanced crystalline correlation lengths of Dr-2-processed blend film indicate a tighter stacking and a higher degree of molecular ordering, which favors efficient charge transport within the film. The above observations offer a clear understanding of the OSCs’ morphology, showing that the dielectric regulator controls molecular packing to some extent. The increased dielectric constant, together with moderate morphological effects, contributes to enhanced exciton dissociation, suppressed recombination, and improved charge transport.

In addition, we conducted a comprehensive study to investigate the morphology and dielectric properties based on D18:L8-BO, D18:Y6, and PM6:L8-BO systems with/without regulators or additives, respectively (Supplementary Figs. 51-54, Supplementary Tables 16, 17). The dielectric and morphology effects of the D18:L8-BO system with conventional additives (DIO and DIB) were studied. The root-mean-square roughness derived from AFM images changes from 1.50 for pristine D18:L8-BO to 3.37 (with DIO) and 1.19 (with DIB), indicating noticeable morphology modulation. In contrast, the dielectric constant remains almost unchanged at ~4.36. These results suggest that conventional liquid additives primarily regulate film morphology without altering the dielectric environment. Upon introduction of dielectric regulators, the dielectric constant of D18:Y6 increases from 3.43 for the pristine system to 3.66 (Dr-1) and 4.19 (Dr-2), while the active layer films maintain unchanged molecular orientation (face-on packing), and the roughness varying slightly from 1.25 to 1.27 and 1.22 nm. Similarly, for PM6:L8-BO, incorporation of dielectric regulators increases the dielectric constant from 4.23 for the pristine system to 4.57 (Dr-1) and 4.92 (Dr-2). The active layer also exhibits face-on molecular packing, and a slight decrease in roughness from 1.59 to 1.53 and 1.49 nm. These results indicate that the introduction of dielectric regulators induces minimal morphological variation while effectively increasing the dielectric constant. Correspondingly, the significant enhancement in device performance can be primarily attributed to the elevated dielectric environment rather than morphological changes.

Furthermore, Kelvin Probe Force Microscopy (KPFM) measurements were investigated to characterize the surface potential variations. Upon the introduction of dielectric regulators, the surface potential variation of the thin film (100 nm) in the dark state are increased from 0.150 V (without) to 0.164 V (Dr-1) and 0.170 V (Dr-2), clearly indicating that dielectric regulator can modulate the local polarization at the donor/acceptor interface (Supplementary Fig. 55). For the thick film (300 nm), the full width at half maximum (FWHM) distributions of the electrostatic potential in the dark state are 16.1, 15.5, and 15.1 mV for D18:L8-BO, D18:L8-BO Dr-1, and D18:L8-BO Dr-2 films, respectively (Supplementary Fig. 56). The uniform surface potential distribution achieved upon the introduction of regulators is highly beneficial for efficient charge transport and for minimizing charge recombination^54,55^. Charge formation is manifested as a shift in the surface potential under illumination compared to the dark state (ΔSP_light-dark_). KPFM characterizations were conducted over the same sample region under three separate conditions: (i) without irradiation (“dark 1”), (ii) under light irradiation (“light”), and (iii) after the cessation of illumination (“dark 2”), employing an experimental configuration detailed in prior work^56^. Upon switching off the illumination, the surface potential immediately reverts to its initial dark-state value. The D18:L8-BO Dr-2 blend film exhibits the highest ΔSP_light-dark_ of 137 mV, in contrast to 95 and 126 mV for both the pristine D18:L8-BO and D18:L8-BO Dr-1 films. This result demonstrates that the Dr-2 treatment promotes a higher level of charge accumulation, which in turn facilitates the enhancement of photocurrent density.

**Fig. 5:**
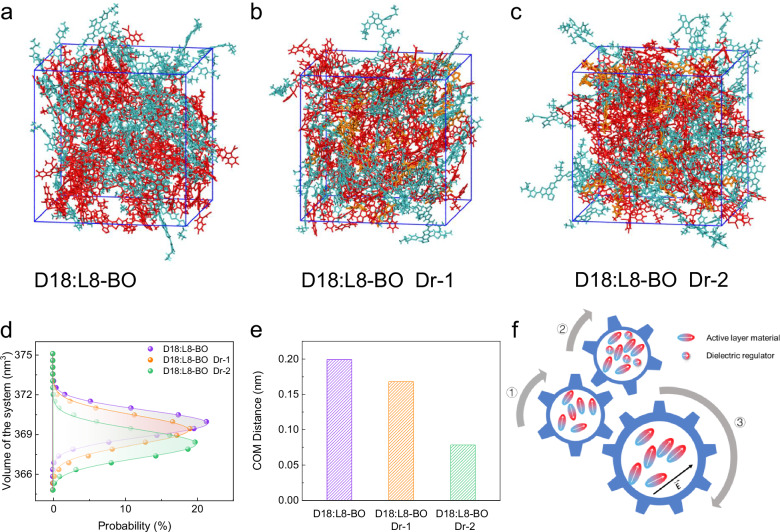
Molecular dynamics (MD) simulations. The final models of molecular dynamics simulations for **a** D18:L8-BO, **b** D18:L8-BO with Dr-1, and **c** D18:L8-BO with Dr-2. **d** Equilibrium volume distributions of the three systems. **e** The average COM distance between D18 and L8-BO of different systems. **f** A schematic diagram of the dielectric regulator on the dielectric constant. ① Active layer without dielectric regulator, ② active layer with dielectric regulator, ③ after annealing of ②.

In addition, to further investigate the effect of the dielectric modulator on the packing circumstances of materials, the molecular dynamics (MD) simulations were performed, and the simulated details can be seen in the supporting information. According to the simulation results, the distribution of donor, acceptor and dielectric modulator is shown in Fig. 5a-c. As shown in Fig. 5d, Supplementary Table 18, D18:L8-BO dealt with Dr-1 shows a medium equilibrium volume distribution. However, the system of D18:L8-BO treated with Dr-2 shows the minimal equilibrium volume distribution among the three systems, indicating the ordered aggregation characteristic of the D18:L8-BO system with the treatment of Dr-2^57^. We can find that the center of mass (COM) distance between D18 and L8-BO was decreased after the addition of a dielectric regulator into the D18:L8-BO film (Fig. 5e). Additionally, the D18:L8-BO film with Dr-2 shows the minimal COM distance, indicating the tightest molecular stacking in the D18:L8-BO film, which is benefit to the charge transport behavior, in conformity with the above volume distribution. Schematic diagram of dielectric regulator on dielectric constant is shown in Fig. 5f. Schematic diagram of parallel, antiparallel and disordered arrangement of dipoles are shown in Supplementary Fig. 57. Furthermore, based on the equilibrium trajectory after evaporation, the static dielectric constants of the three systems were calculated using Gromacs. The finite system Kirkwood g factor (Gk)^58^, which describes the dipole orientation of molecules, is closest to 1 for the D18:L8-BO system after dealing with Dr-2, indicating that the dipoles of molecules in this system are closest to a normal parallel arrangement. Therefore, Dr-2 induces the ordered arrangement of molecules, causing the dipoles of molecules to tend towards parallel arrangement, making the system more easily polarized and increasing the dielectric constant^59^ (Process ②).

### Generality study

To investigate the generality of this strategy, several donor:accpetor systems were treated with/without dielectric regulators. Photovoltaic performances of corresponding photovoltaic devices are presented in Supplementary Figs. 58-60, Supplementary Table 19. The results in different systems show that the photovoltaic performance of devices with dielectric regulators is higher than that of devices without dielectric regulators. Wth the assistence of Dr-2, the devices exhibit optimal photovoltaic performance among all devices. Concretely, the PM6:L8-BO-based device treated with Dr-2 achieves a PCE of 19.62%, with a *V*_oc_ of 0.887 V, a *J*_sc_ of 27.50 mA cm^-2^, and an FF of 80.46% (Supplementary Fig. 58, Supplementary Table 19). Similarly, the Dr-2 treated PM6:Y6-based device yields a PCE of 19.16%, with a *V*_oc_ of 0.840 V, a *J*_sc_ of 28.97 mA cm^-2^, and an FF of 78.75% (Supplementary Fig. 59, Supplementary Table 19). Furthermore, the Dr-2-treated D18:Y6-based OSC produces a PCE of 19.20%, with a *V*_oc_ of 0.840 V, a *J*_sc_ of 29.11 mA cm^-2^ (*J*_EQE_ of 27.70 mA cm^-2^), and an FF of 78.51% (Supplementary Fig. 60, Supplementary Table 19). Hence, this work demonstrates the effectiveness of a design strategy for a dielectric regulator, thereby exploiting a general strategy to improve the device performance.

## Discussion

In summary, this work presents a design strategy for dielectric regulators to overcome the intrinsic limitations associated with the low dielectric constants of organic semiconductors. In comparison to Dr-1, Dr-2 features an extended *π*-conjugated backbone, enhanced molecular polarizability, a larger dipole moment and strengthened charge delocalization via the integration of S···F NoCLs. Upon incorporation of Drs, the dielectric environment and molecular packing in the active layer are simultaneously modulated. Thus, this strategy facilitates exciton dissociation, boosts charge transport, and minimizes recombination losses, collectively contributing to a high PCE of 20.85% in binary OSCs. Furthermore, the approach demonstrates promising thickness tolerance with a record efficiency of 19.56% for a thick-film device (300 nm) and broad compatibility across different systems. The dielectric constant strategy effectively tackles severe charge recombination associated with thicker films, delivering an approach for fabricating efficient thick-film devices. These results highlight the critical role of dielectric environment engineering, and offer a versatile, scalable strategy to guide the design of a dielectric regulator for high-performance OSCs.

## Methods

### Materials

All reagents were purchased from Inno-chem, *J&K*, 3 A Chemicals, Derthon, Energy Chemical, Meryer Chemical Technology Co., Ltd., Beijing Mairuida Technology Co., Ltd, Sigma-Aldrich, and Alfa Aesar Chemical Company, unless specified and used as received. The ITO glass (15 Ω, transmittance ≥ 94.5%) was from South China Science & Technology Company Limited. D18 was from HyperPV, Co., Ltd. L8-BO was from Shenzhen Ruixun Technology Co., Ltd. The metal Ag is from ZhongNuo Advanced Material (Beijing) Technology Co., Ltd (www.znxc.com).

### Materials synthesis

#### General procedure for the synthesis of compounds 1a and 1b

A mixture of 2-bromothieno[3,2-*b*]thiophene (1.10 g, 5.0 mmol), (3,5-difluorophenyl)boronic acid (or (2,6-difluorophenyl)boronic acid) (1.18 g, 7.5 mmol), P(t-Bu)_3_Pd G3 (57.2 mg, 0.1 mmol), and K_3_PO_4_ (5.3 g, 25.0 mmol) in dry THF (20.0 mL) was stirred under protection of argon at 45 °C for 2 h. The resulting mixture was washed with water, followed by extraction with dichloromethane (DCM). The organic layer was dried over anhydrous Na_2_SO_4_. After removing the solvent by rotary evaporation, the residue was then subjected to silica gel column chromatography eluted with petroleum ether (PE) to afford **compound 1** as a white solid.

**Compound 1a** (1.15 g, 91%): ^**1**^**H NMR** (500 MHz, CDCl_3_, δ): 7.51 (s, 1H), 7.43-7.41 (m, 1H), 7.27-7.26 (m, 1H), 7.15-7.12 (m, 2H), 6.77-6.72 (m, 1H). ^**13**^**C NMR** (126 MHz, CDCl_3_, δ): 164.48, 164.38, 162.51, 162.40, 143.52, 140.02, 139.29, 137.95, 128.22, 119.67, 116.86, 108.78, 108.72, 108.61, 108.56, 103.15, 102.95, 102.74.

**Compound 1b** (1.16 g, 92%): ^**1**^**H NMR** (500 MHz, CDCl_3_, δ): 7.72 (s, 1H), 7.44-7.41 (m, 1H), 7.30-7.28 (m, 1H), 7.26-7.22 (m, 1H), 7.04-6.99 (m, 2H). ^**13**^**C NMR** (126 MHz, CDCl_3_, δ): 160.86, 160.81, 158.86, 158.81, 140.69, 139.21, 131.28, 128.70, 128.61, 128.53, 128.19, 121.64, 121.59, 121.54, 119.42, 112.78, 112.65, 112.52, 112.22, 112.18, 112.05, 112.01.

### General procedure for the synthesis of compound Dr-1 and Dr-2

Compound 1 (1.0 g, 4.0 mmol) was added to DMF (15.0 mL) solution with *N*-chlorosuccinimide (587.5 mg, 4.4 mmol), followed by 0.1 mL acetic acid. After being stirred for 3 h at 45 °C, the resulting mixture was quenched with water and extracted with DCM. The organic layer was dried over anhydrous Na_2_SO_4_. After the solvent was removed by rotary evaporation, the residue was then subjected to silica gel column chromatography eluted with PE to afford the target compound as a white solid.

**Dr-1** (974.92 mg, 85%): ^**1**^**H NMR** (500 MHz, CDCl_3_, δ): 7.39-7.33 (m, 1H), 7.13-7.06 (m, 3H), 6.77-6.72 (m, 1H). ^**13**^**C NMR** (126 MHz, CDCl_3_, δ): 164.56, 164.46, 162.58, 162.48, 142.77, 137.47, 137.43, 137.10, 132.22, 118.94, 116.51, 108.67, 108.61, 108.50, 108.45, 103.22, 103.01, 102.81. HRMS-ESI (m/z) calcd for C_12_H_5_ClF_2_S_2_^+^ [M]^+^: 285.9484, found 285.9482.

**Dr-2** (1.01 g, 88%):^**1**^**H NMR** (500 MHz, CDCl3, δ): 7.63-7.61 (m, 1H), 7.26-7.22 (m, 1H), 7.18-7.16 (m, 1H), 7.04-6.99 (m, 2H). ^**13**^**C NMR** (126 MHz, CDCl3, δ): 160.73, 160.68, 158.73, 158.68, 138.32, 136.51, 132.02, 130.56, 128.79, 128.70, 128.62, 121.29, 121.24, 121.18, 118.81, 112.32, 112.25, 112.21, 112.19, 112.08, 112.05. HRMS-ESI (m/z) calcd for C_12_H_5_ClF_2_S_2_ + H^+^ [M + H]^+^: 286.9562, found 286.9565.

### Device fabrication

The devices were fabricated in a device configuration of ITO/2PACZ/active layer/ PNDIT-F3N/Ag. After ultrasonically precleaned with detergent, deionized water, acetone and absolute ethanol in sequence for 30 min, the ITO-coated glass substrates were dried by nitrogen flow. After plasma cleaning for 1 min, 2PACZ was spin-coated onto clean ITO-coated glass substrates (3000 rpm for 20 s) and baked at 100 °C for 5 min. The chloroform solution of D18 and L8-BO with/without dielectric regulator was spin-coated onto the 2PACZ film. The concentration of D18 is 5 mg/ml. Then, a thin PNDIT-F3N layer was spin-coated on top of the active layer at 4200 rpm for 20 s, followed by the deposition of Ag (100 nm) (evaporated under the vacuum condition of 3 × 10^-4^ Pa through a shadow mask).

### Device characterization

The current density-voltage (*J*-*V*) curves were measured with a computer-controlled Keithley 2450 Source Measure Unit under an AM 1.5 G white light source; the optical power at the sample was 100 mW/cm^2^ (Enlitech). Simulator irradiance was characterized using a calibrated spectrometer, and illumination intensity was set using a certified silicon diode (SRC-2020, Enlitech). In addition, the external quantum efficiency (EQE) spectrum was measured using the Enlitech QE-R system. Time-resolved femtosecond transient absorption (TA) spectroscopy was performed on an Ultrafast Helios pump-probe system in collaboration with a regenerative amplified laser system from Coherent. Atomic force microscopy was used to characterize the morphology of the device in the tapping mode. The photo-CELIV, CE, EIS and Mott-Schottky measurements were performed on an all-in-one characterization platform, Paios, developed by Fluxim AG.

### Carrier mobility measurements

The space-charge-limit current (SCLC) method was carried out to test the carrier mobility of devices. The device structure (ITO/2PACZ/Active layer/Ag) was constructed to measure their hole mobility. The electron mobility was measured by constructing the device structure of ITO/ZnO/Active layer/PNDIT-F_3_N/Ag. The dark current was fitted to the model of a single carrier SCLC based on the equation of *J* = 9*ε*_0_*ε*_r_*μV*^2^/8d^3^, which is used to examine the mobility. In the above equation, *J*, *ε*_0_, *ε*_r_, *μ*, and *d* are the current density, the permittivity of free space, the relative dielectric constant, the charge carrier mobility, and the thickness of the active layer, respectively. The carrier mobility was obtained by the slope of *J*^0.5^ ~ *V* curves.

### Bias-assisted charge extraction (BACE)

In a BACE measurement, the device is illuminated with a steady-state light source with varying intensities while being held at a constant bias equal to the *V*_OC_ at that intensity. The *V*_OC_ and short circuit current (*J*_sc_) of the device have been measured in advance for each particular light intensity.

### Capacitance-voltage measurement

The capacitance-voltage measurement of devices was performed at different applied DC voltages, and the structure of the device was the same as that of an organic solar cell. The deep trap state can be studied at low-frequency response. Here, the additional capacitance at applied voltage (V) close to the build-in voltage (*V*_bi_) is assumed to be caused by trapped carriers being releasing to the mobility edge. The trapped charge density of accessible trap states can be calculated according to Eq. (6).

6 $$\frac{1}{{C}^{2}} {{{\rm{\alpha }}}}-\frac{2}{{N}_{t}{\varepsilon }{qA}^{2}}$$

where *C* is the capacitance and *q* is the elementary charge.

### PL and TRPL measurements

The steady-state PL spectra and TRPL experiments were acquired through a confocal optical microscope (Nanofinder FLEX2, Tokyo Instruments, Inc.) equipped with a time-correlated single-photon counting (TCSPC) module (Becker & Hickl, SPC-150). All of the PL spectra were measured using a charge-coupled device (CCD) sensor (DU420A-OE, Andor). The excitation wavelength was fixed at 400 nm. The excitation power of all the PL spectra and TRPL experiments was fixed at 1 μW. The diameter of the laser spot was about 8 μm, and its area was calculated as S = 5.02 × 10^-7^ cm^2^.

### Morphology characterization

AFM was carried out to measure the morphology of the device in a tapping mode under ambient conditions.

### Photostability characterization

Photostability of encapsulated devices was characterized via maximum power point tracking (MPPT) under simulated AM1.5 G illumination (100 mW cm^-2^) using a multi-channel solar cells stability test system (MSCLT-1) from Wuhan 91PVKSolar Technology Co., Ltd., China.

### Density functional theory (DFT) calculations

All the geometrical and electronic structures of D18, L8-BO, Dr-1 and Dr-2 were calculated at the B3LYP-D3 level of theory^60,61^ using the basis set 6-31 G(d)^62–64^ in the Gaussian16 Program^65^, including the geometric structures, the restrained electrostatic potential (RESP) charges^66^ and the electrostatic potential (ESP) ^29^ map. ESP visualization was performed using VMD^67^.

### Molecular dynamics (MD) simulations

Molecular dynamics (MD) simulations were performed on D18:L8-BO, D18:L8-BO:Dr-1, and D18:L8-BO:Dr-2 systems using GROMACS 2019.6^68^. All molecules were parameterized with the General AMBER Force Field (GAFF)^69^ via AmberTools, with partial atomic charges derived through the restrained electrostatic potential (RESP) fitting method in Multiwfn^70^. Molecular ratios mirrored those in actual devices within the initial 10 nm × 10 nm × 10 nm box. To simulate the effects of additives accurately, relaxed potential energy surface scans at the B3LYP-D3/6-31 + G(d) level were conducted for Dr-1 and Dr-2 molecules, then important proper dihedral parameters were refitted to meet the potential energy surfaces.

### Reporting summary

Further information on research design is available in the Nature Portfolio Reporting Summary linked to this article.

## Supplementary information

Supplementary Information

Reporting Summary

Transparent Peer Review file

## Source data

Source Data

## Data Availability

The data generated in this study are provided in the Source Data file published alongside this paper. Source data are provided with this paper.
