# Supplementary material for: High performance organic solar cell enabled by manipulating the exciton dissociation and charge transfer via dielectric engineering
Source: Nat Commun. 2026 Jun 16;17:7649. doi: 10.1038/s41467-026-74112-x (PMC13434753; doi:10.1038/s41467-026-74112-x)
Supplement: Supplementary file 1 [file 41467_2026_74112_MOESM1_ESM.pdf]

Supplementary Information

High performance organic solar cell enabled by manipulating the exciton dissociation and charge transfer via dielectric engineering

Yanan Wei[1,7], Xiaobin Gu[1,7], Xue Shi[2], Meng Zhang[1], Jikai Lv[1], Xubing Bai[1], Lixia Wang[1], Xin Zhang[1], Xiangyue Meng[1], Jianqi Zhang[3], Xiaotao Hao[2], Qian Peng[4], Yunhao Cai[*1], Hui Huang[*1,5,6]

[1]College of Materials Science and Opto-Electronic Technology, University of Chinese Academy of Sciences, Beijing 100049, P. R. China

[2]School of Physics, State Key Laboratory of Crystal Materials, Shandong University, Jinan, Shandong 250100, P. R. China

[3]Key Laboratory of Nanosystem and Hierarchical Fabrication, National Center for Nanoscience and Technology, Beijing 100190, China

[4]University of the Chinese Academy of Sciences, School of Chemical Sciences, Beijing 100049, P. R. China

[5]School of Chemical Engineering and Technology, State Key Laboratory of Chemical Engineering and Low-Carbon Technology, Tianjin University, Tianjin 300072, China.

[6]The International Joint Institute of Tianjin University, Fuzhou 350205, China.

[7]These authors contributed equally: Yanan Wei, Xiaobin Gu.

* Corresponding author. Email address: caiyunhao@ucas.ac.cn, huihuang@ucas.ac.cn

Keywords: Organic solar cells, dielectric regulator, exciton dissociation, charge transfer

1. Supplementary Figures

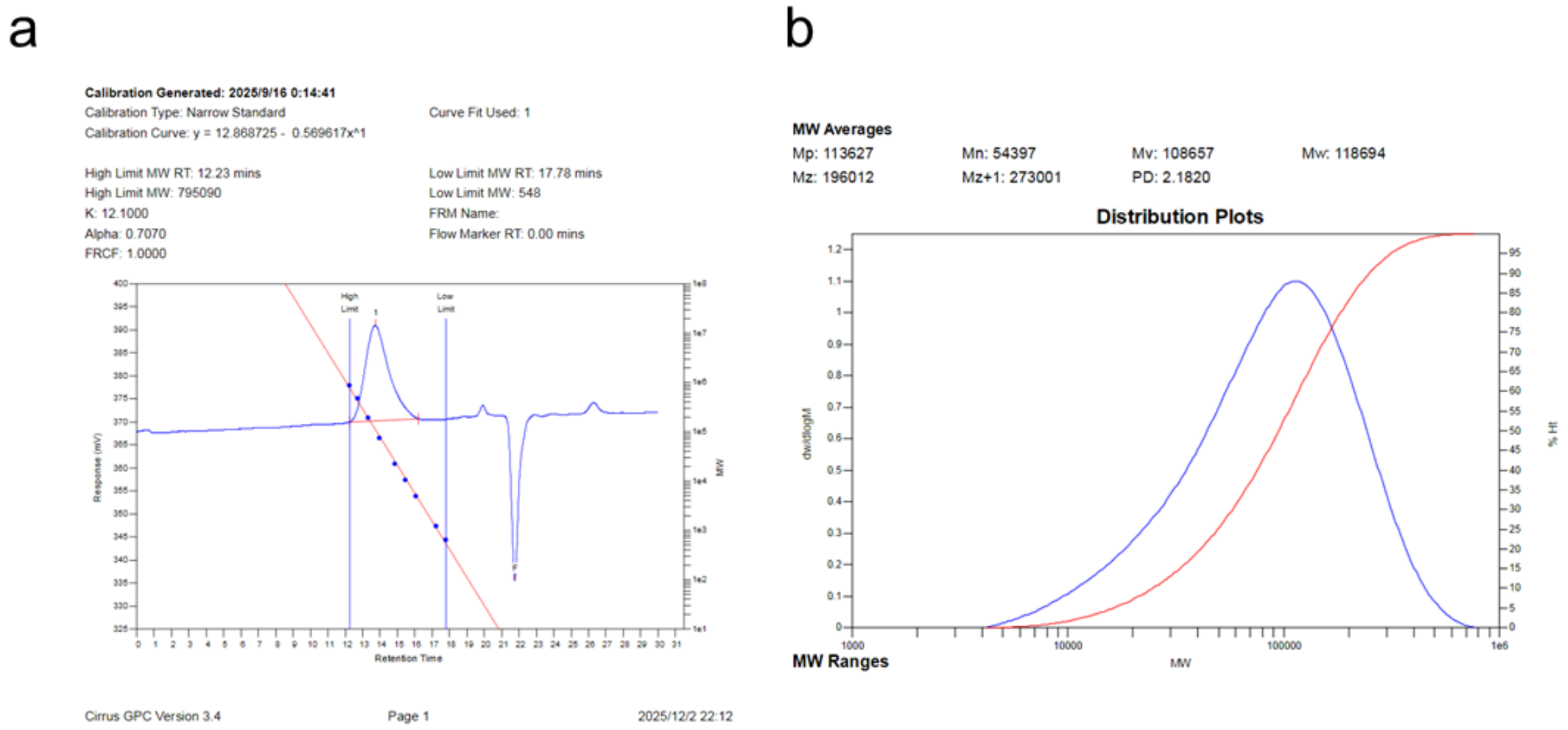

Supplementary Fig. 1. Gel Permeation Chromatography (GPC) trace of D18.

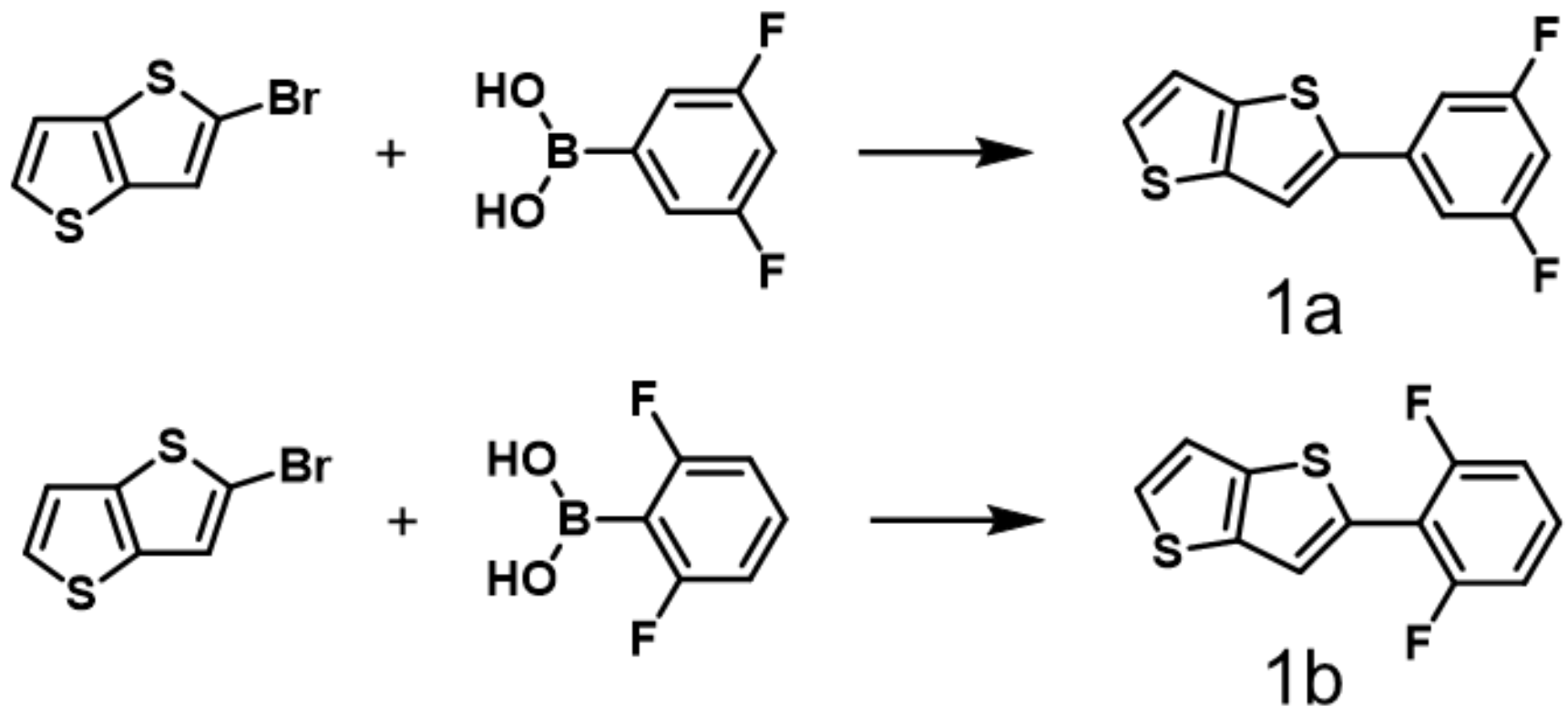

Supplementary Fig. 2. Synthetic routes for compound 1a and 1b.

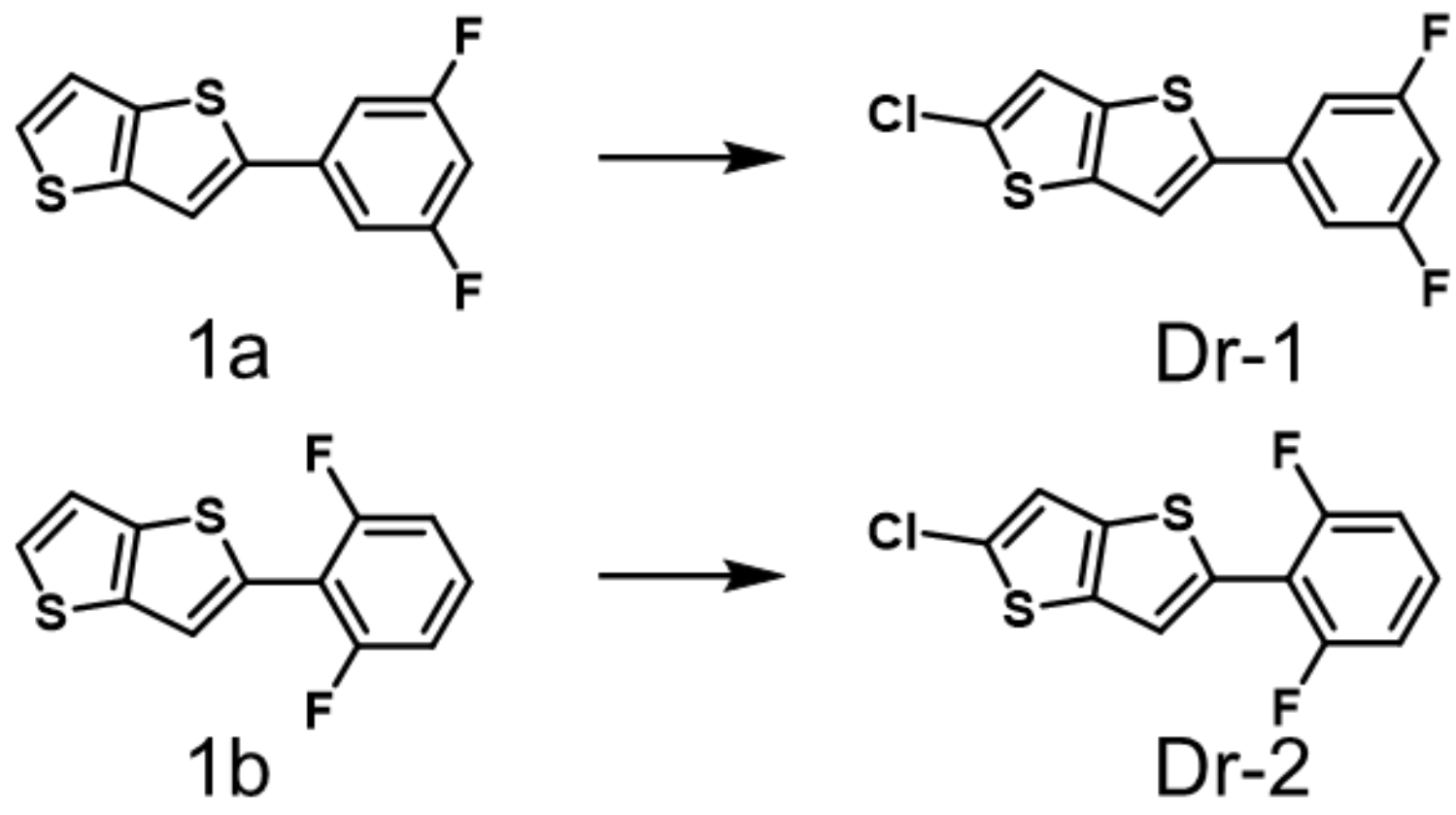

Supplementary Fig. 3. Synthetic routes for Dr-1 and Dr-2.

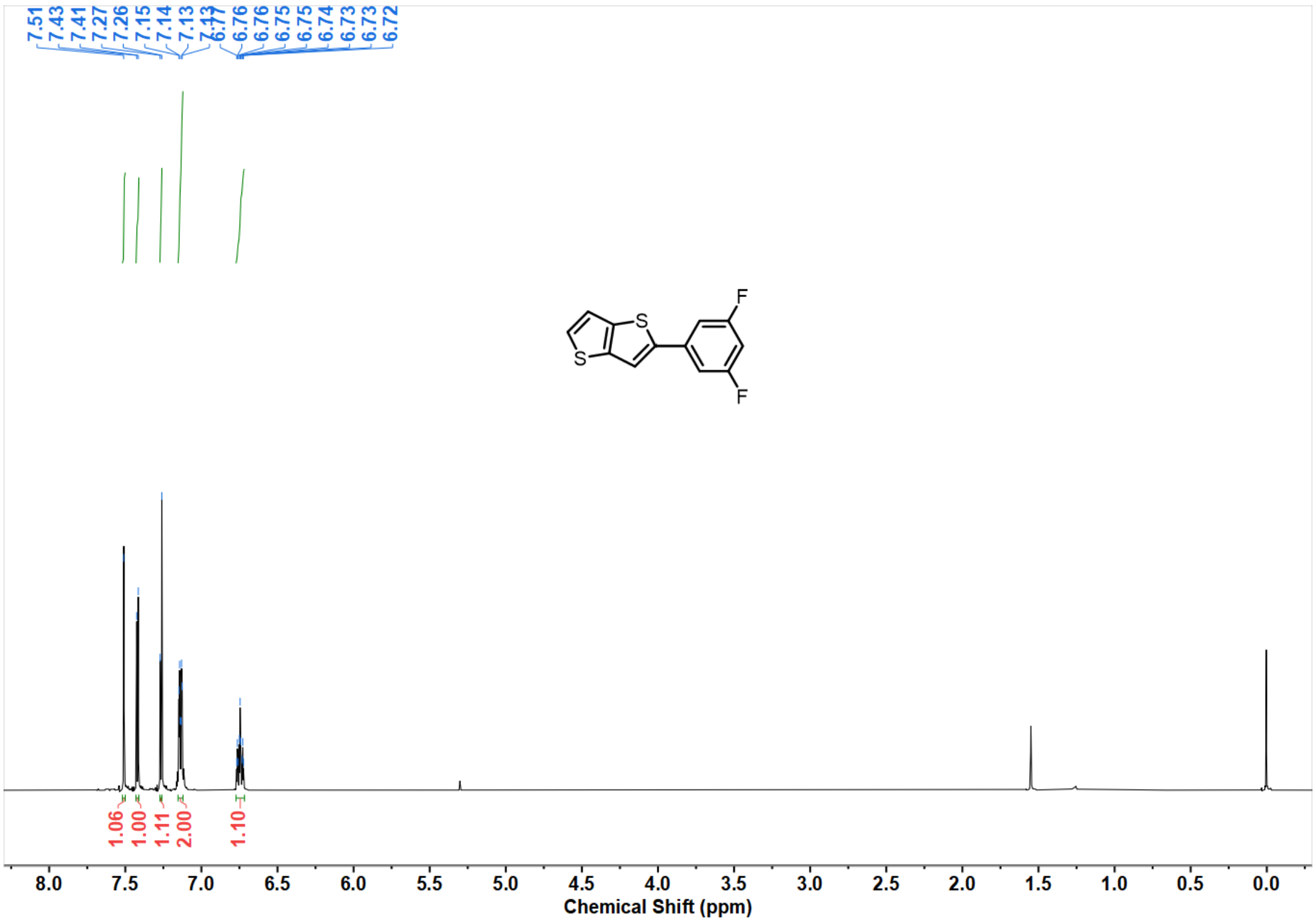

Supplementary Fig. 4. ^{1}H NMR (500 MHz) spectrum of compound 1a.

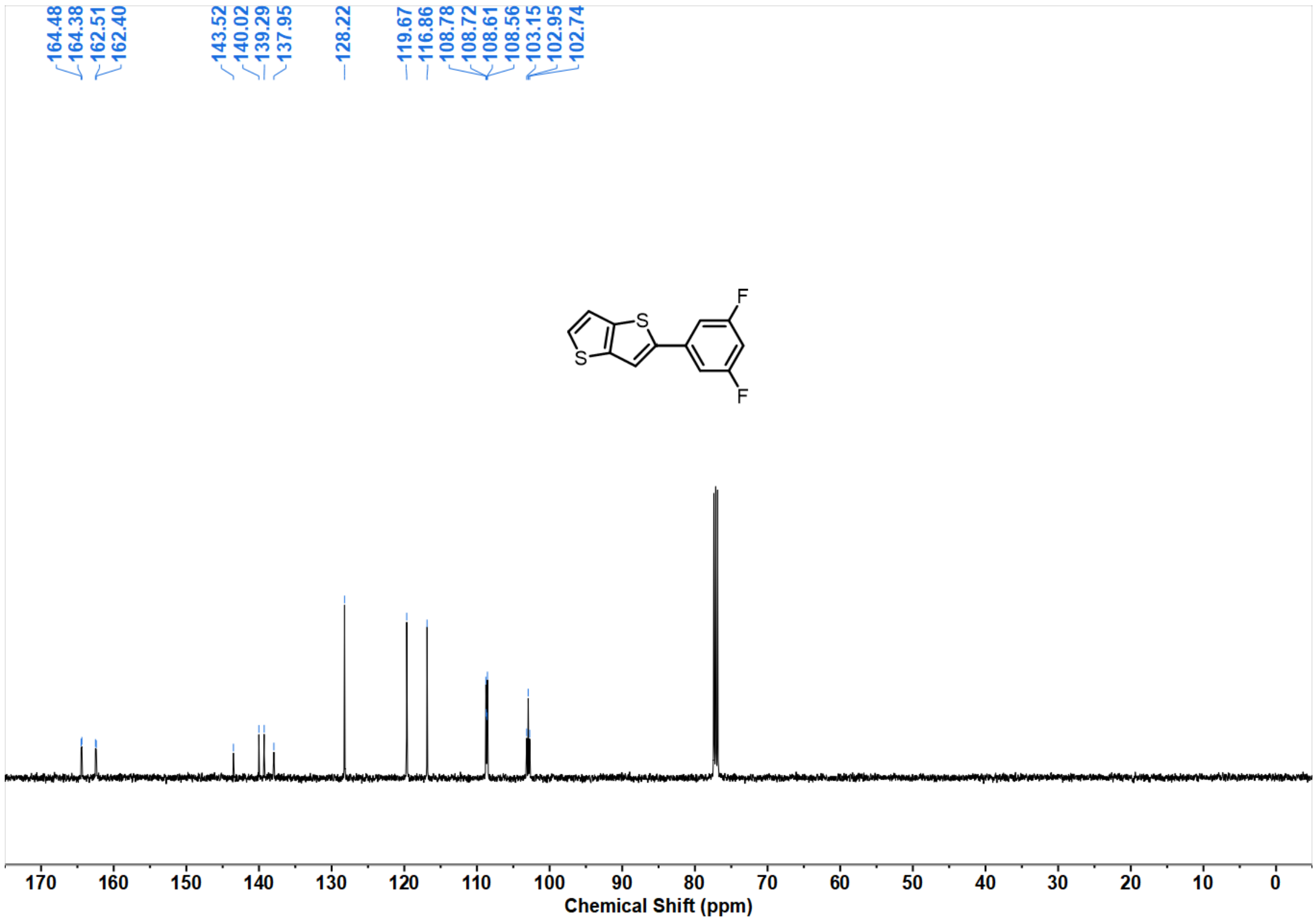

Supplementary Fig. 5. ^{13}C NMR (126 MHz) spectrum of compound 1a.

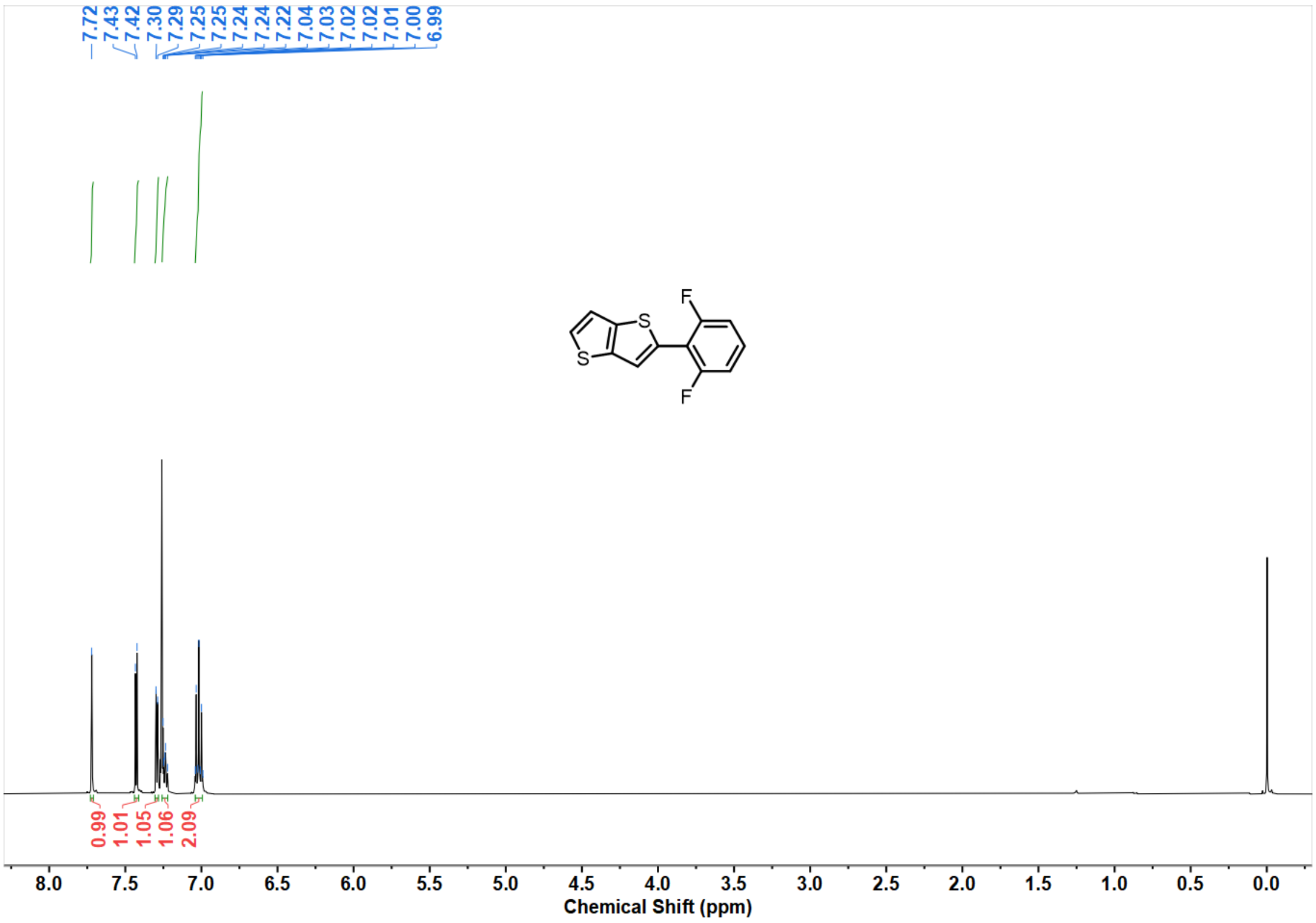

Supplementary Fig. 6. ^{1}H NMR (500 MHz) spectrum of compound 1b.

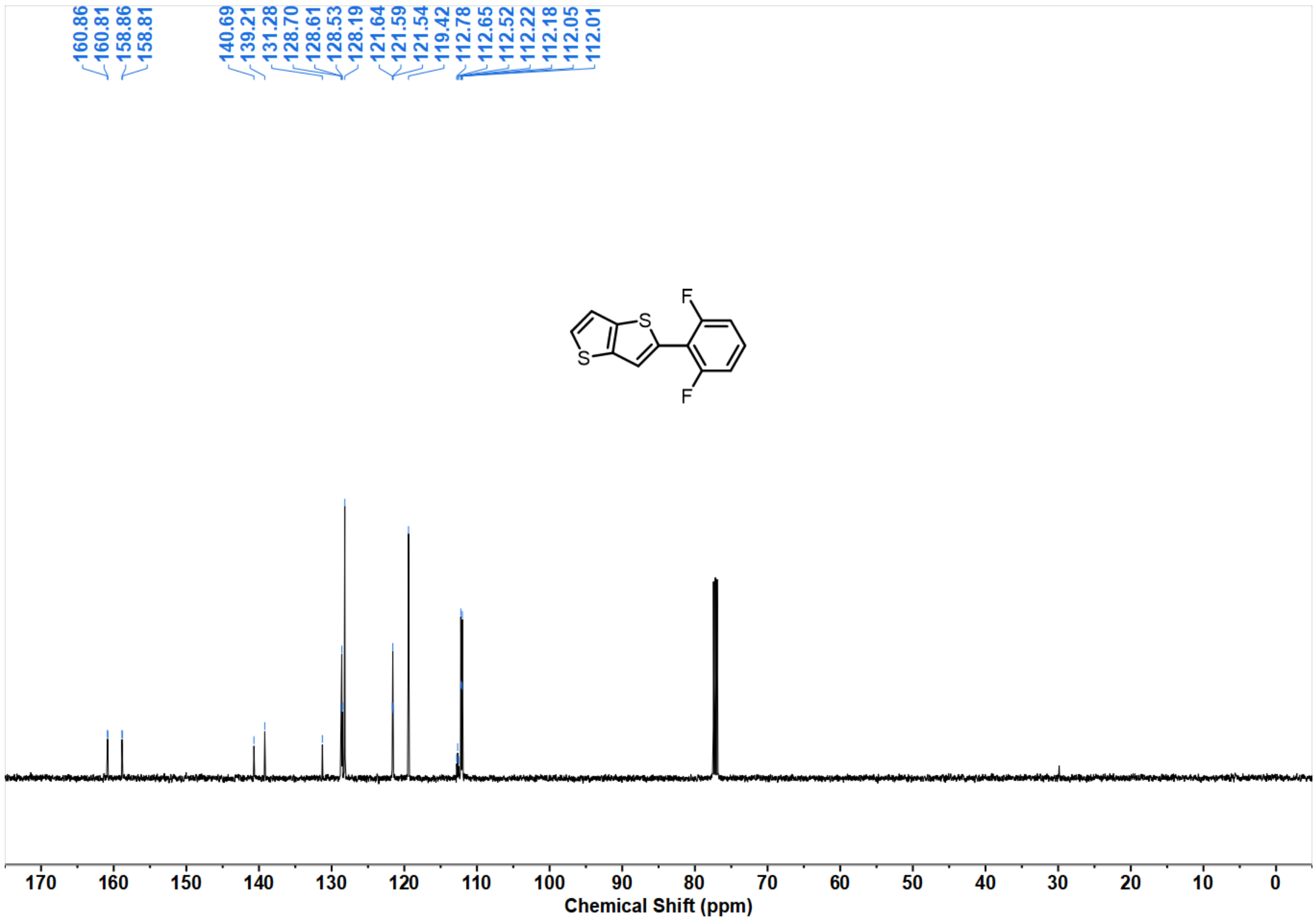

Supplementary Fig. 7. ^{13}C NMR (126 MHz) spectrum of compound 1b.

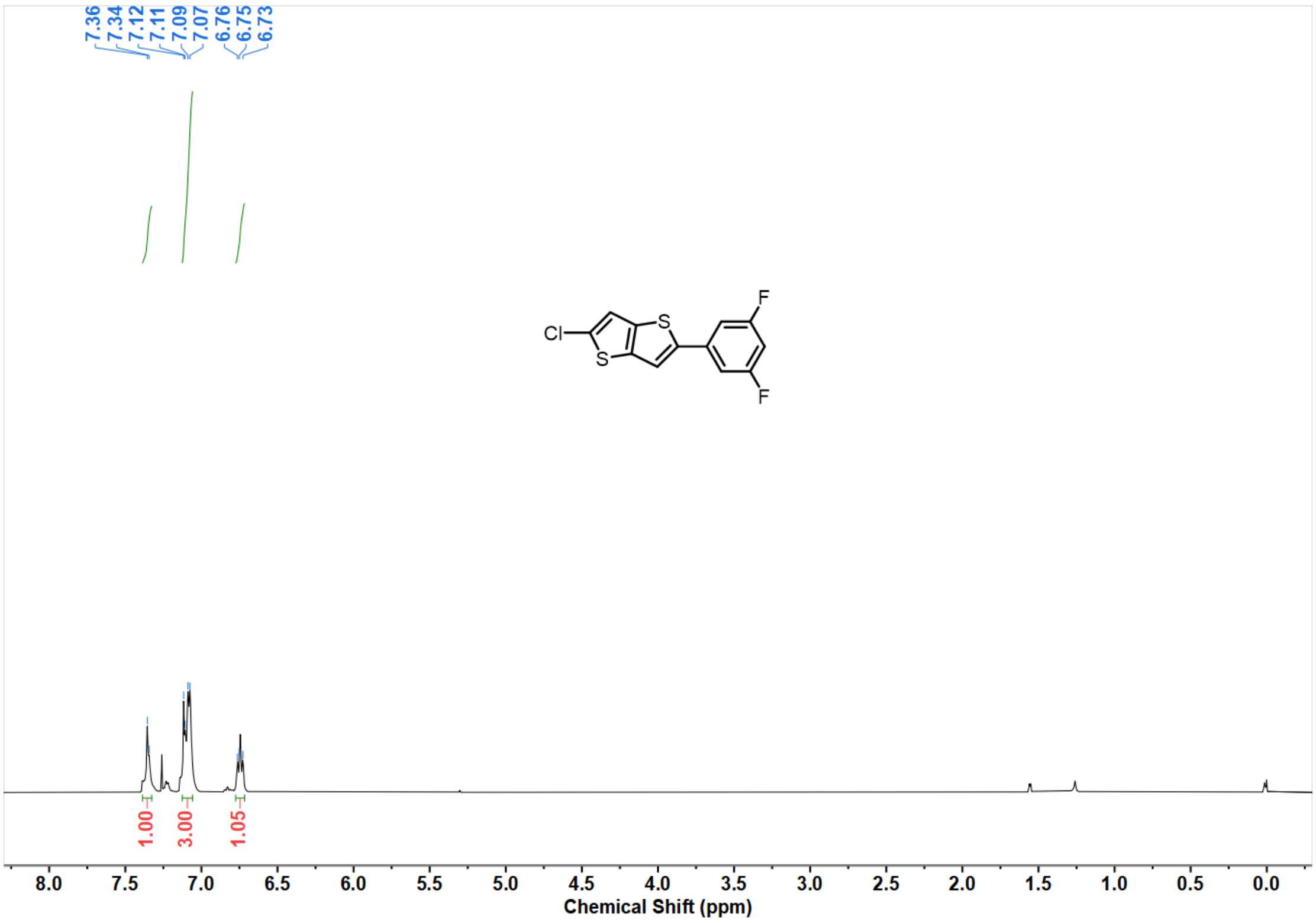

Supplementary Fig. 8. ^{1}H NMR (500 MHz) spectrum of Dr-1.

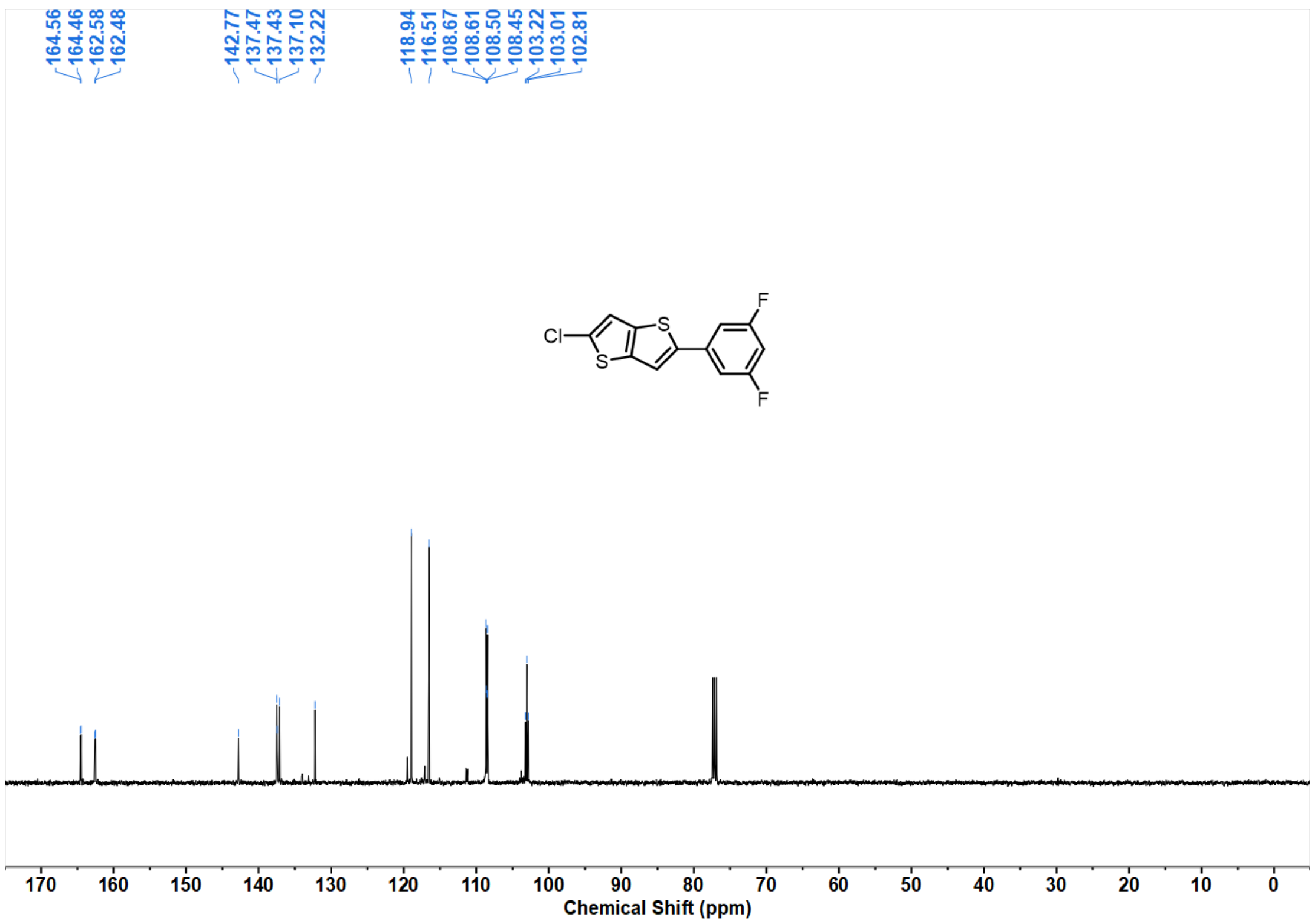

Supplementary Fig. 9. ^{13}C NMR (126 MHz) spectrum of Dr-1.

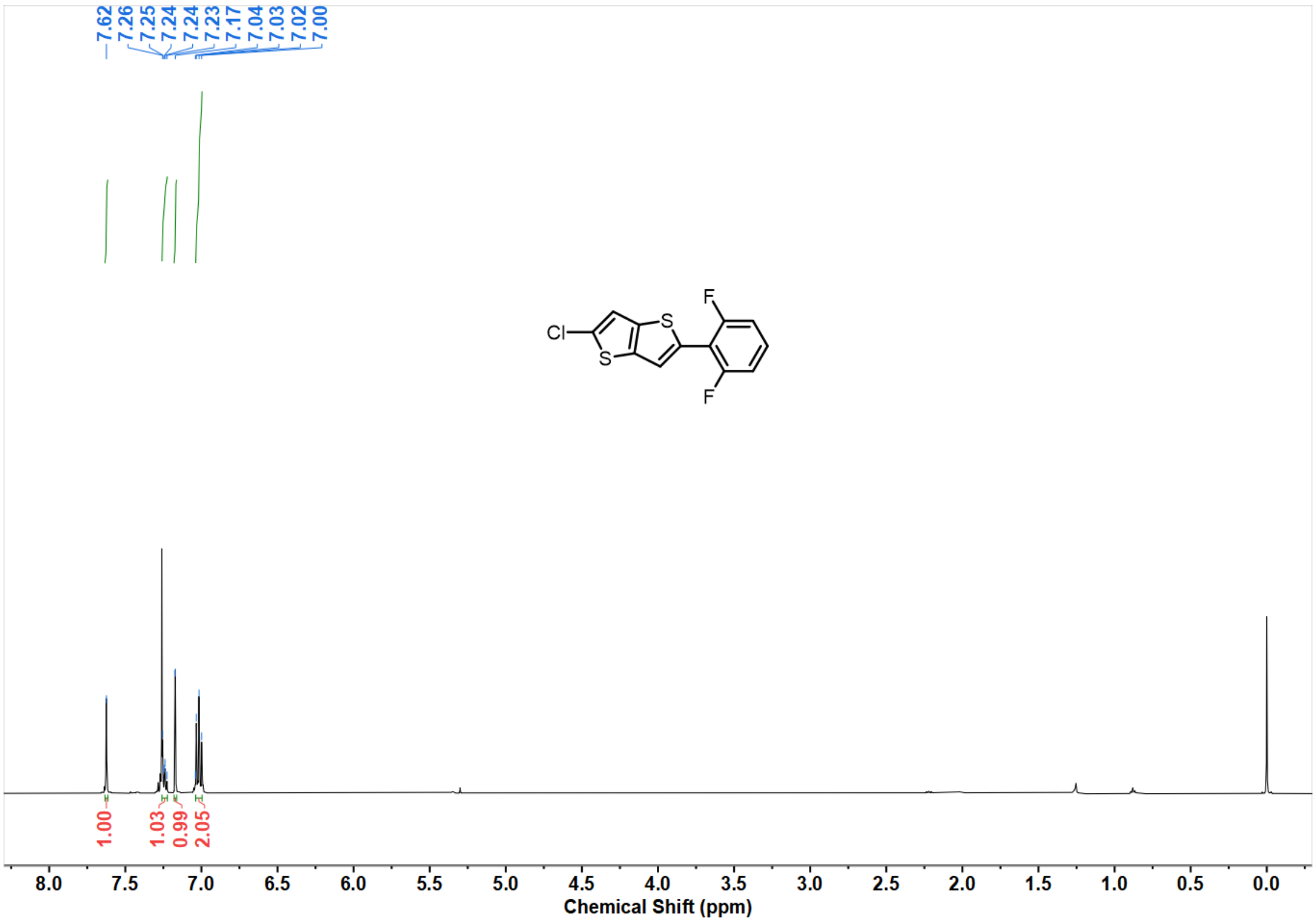

Supplementary Fig. 10. ^{1}H NMR (500 MHz) spectrum of Dr-2.

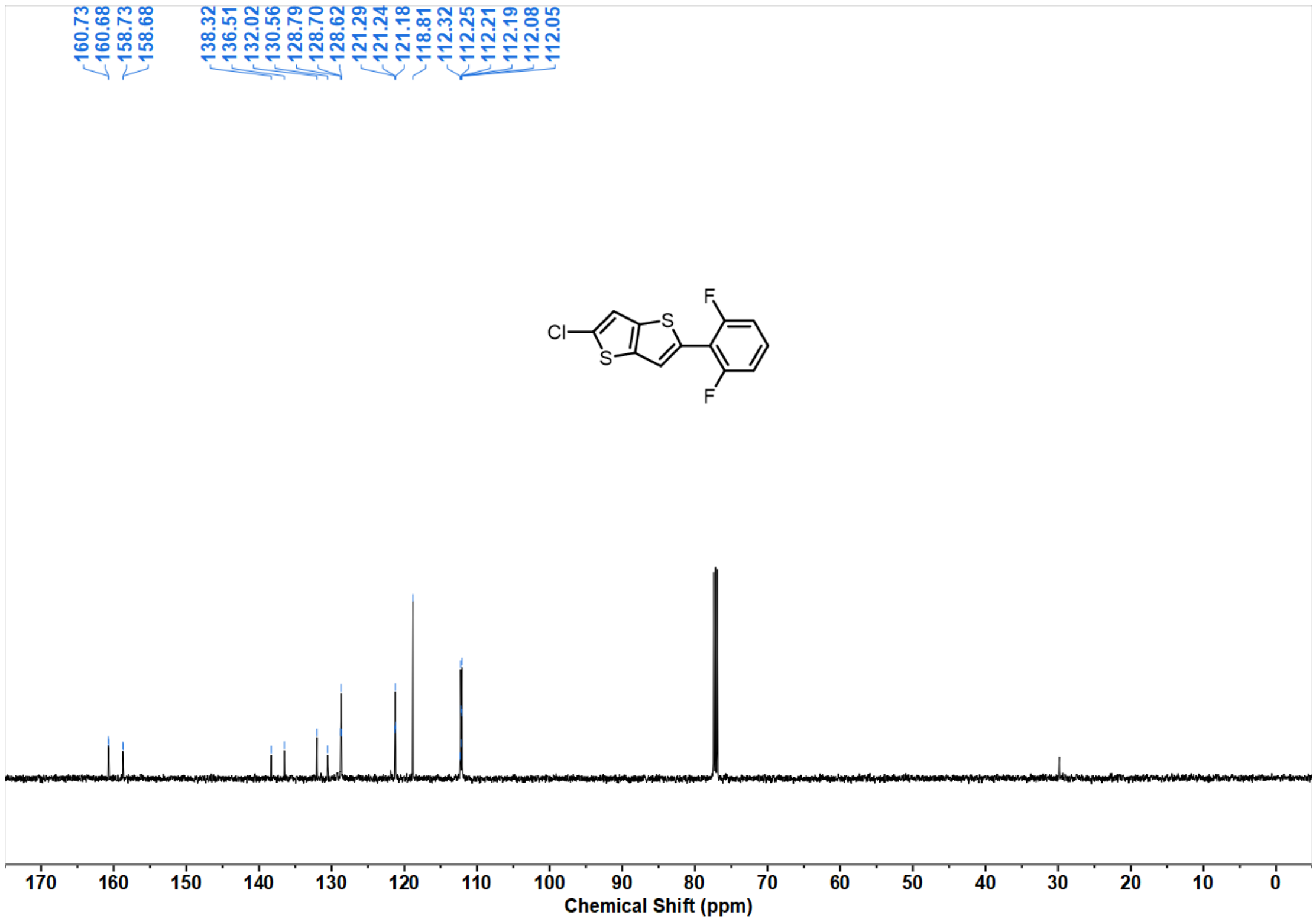

Supplementary Fig. 11. ^{13}C NMR (126 MHz) spectrum of Dr-2.

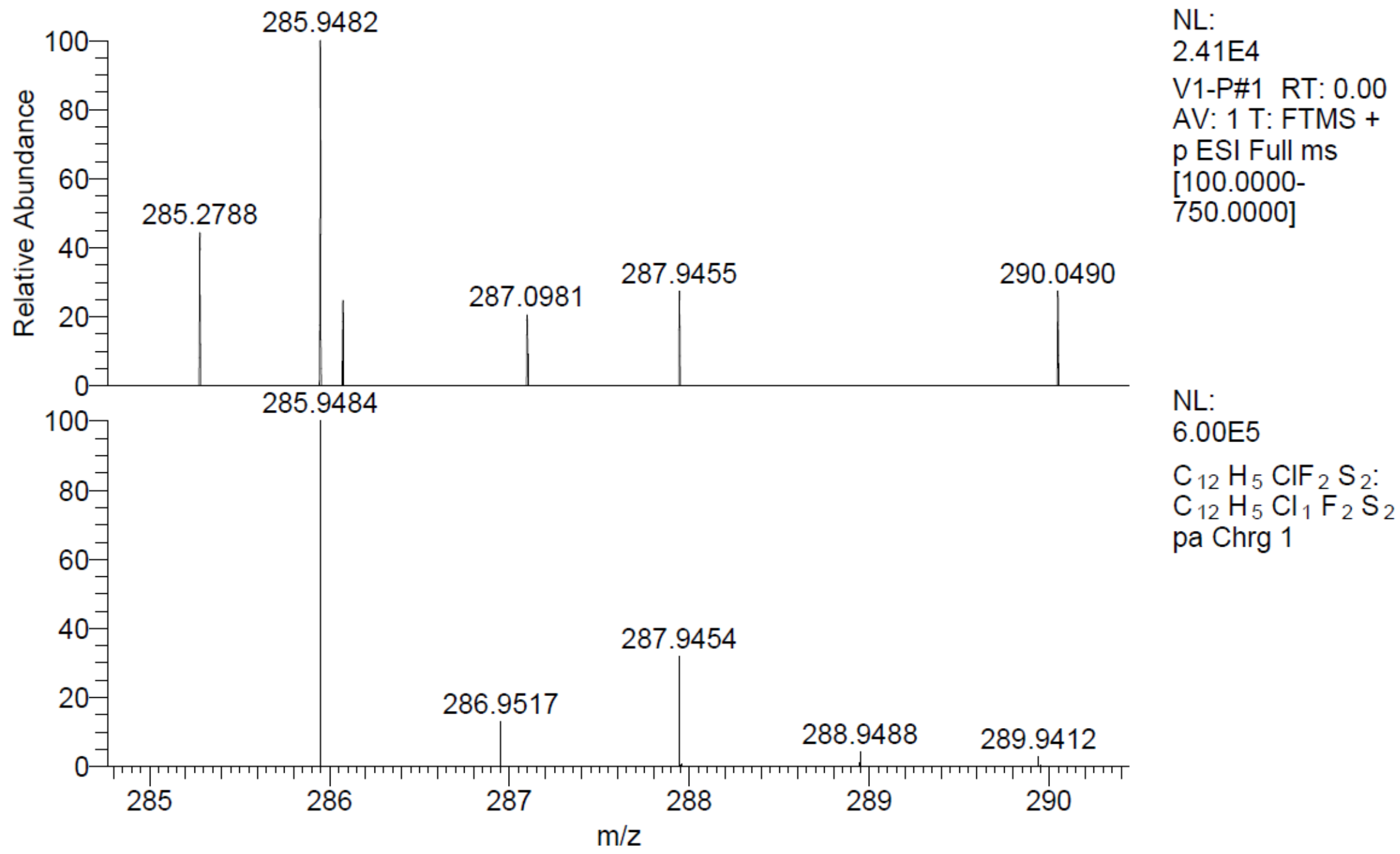

Supplementary Fig. 12. The HRMS (ESI+) analysis of Dr-1.

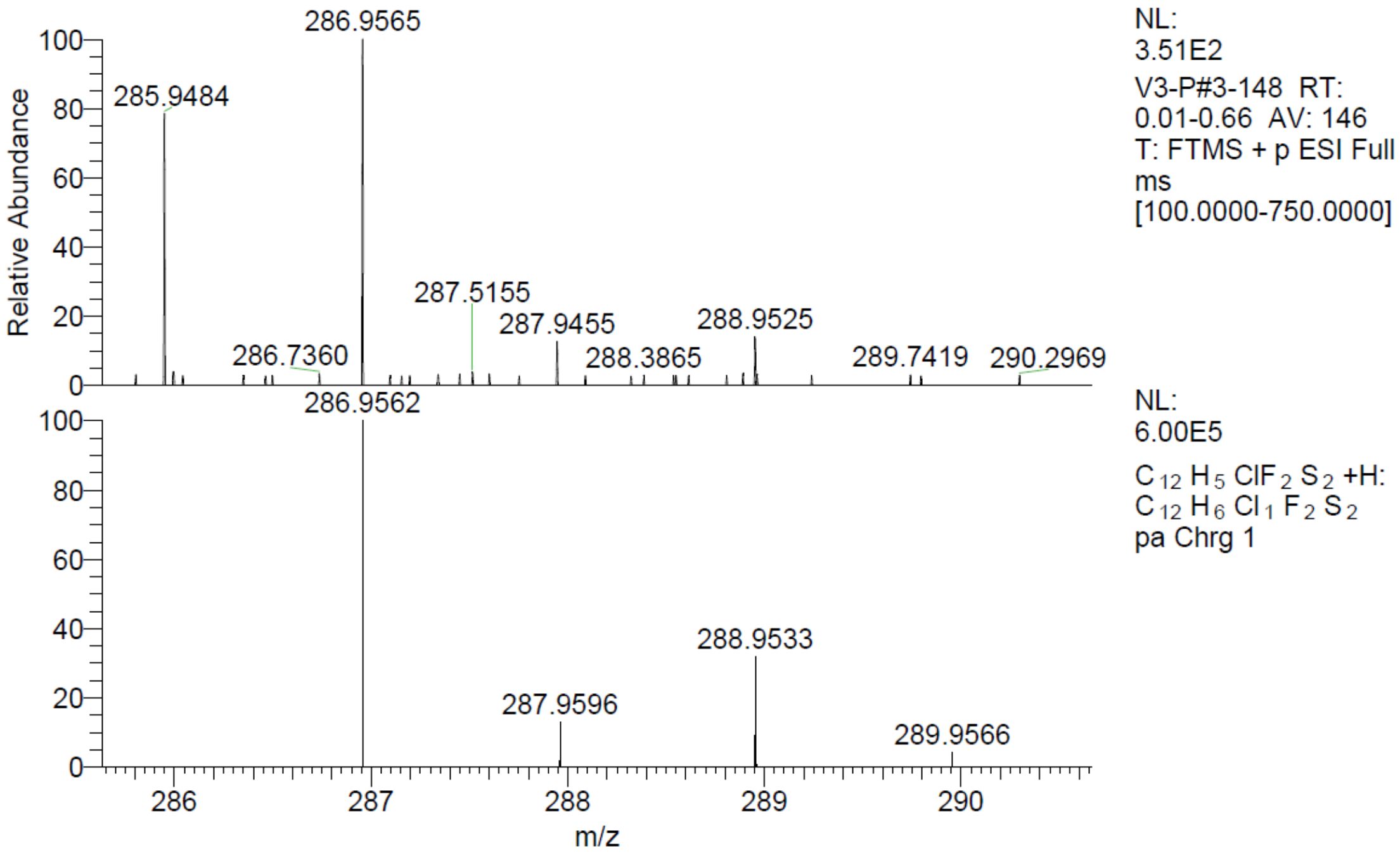

Supplementary Fig. 13. The HRMS (ESI+) analysis of Dr-2.

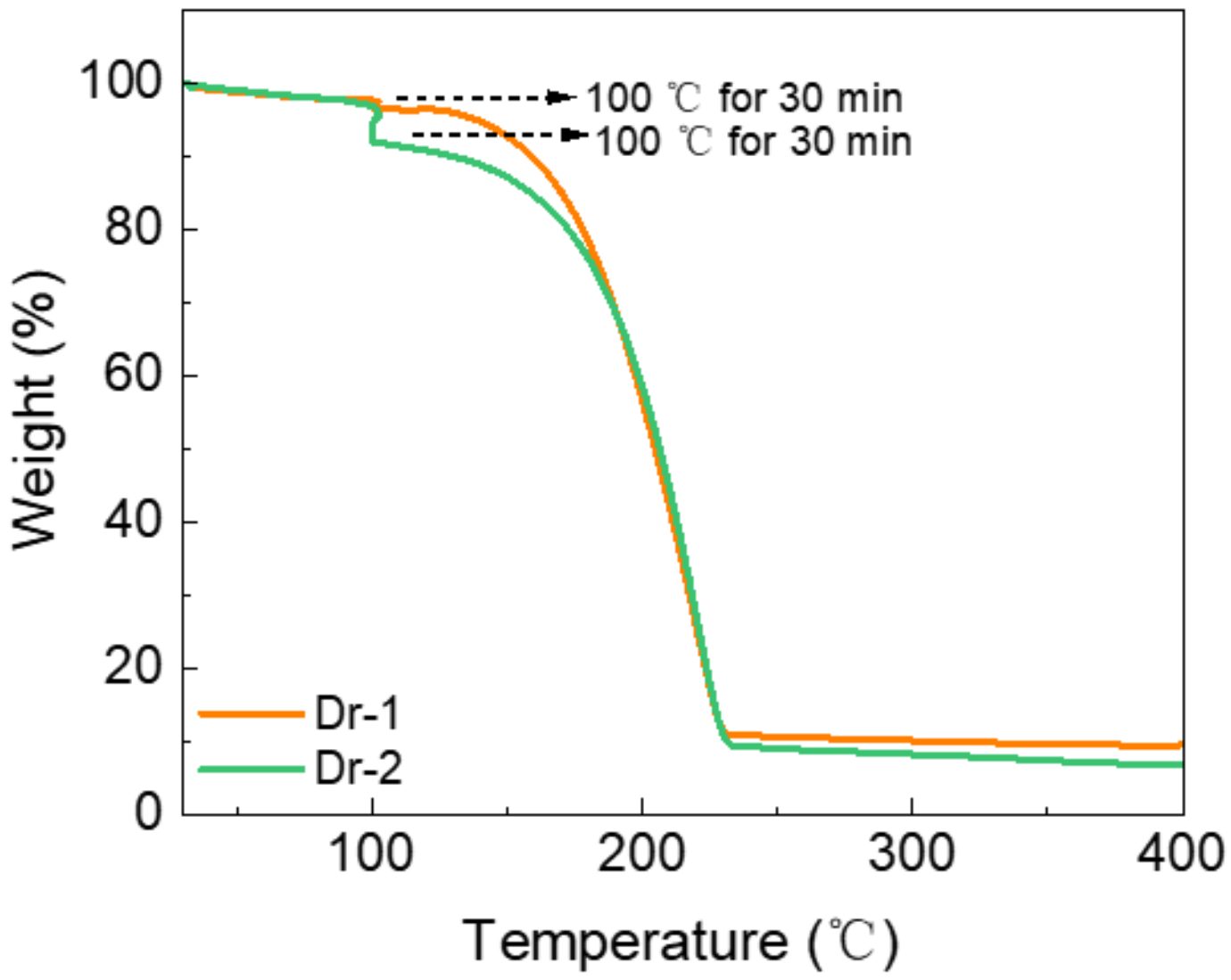

Supplementary Fig. 14. TGA curves of Dr-1 and Dr-2 at a scan rate of 10 ℃/min and maintaining the sample at 100 ℃ for 30 minutes.

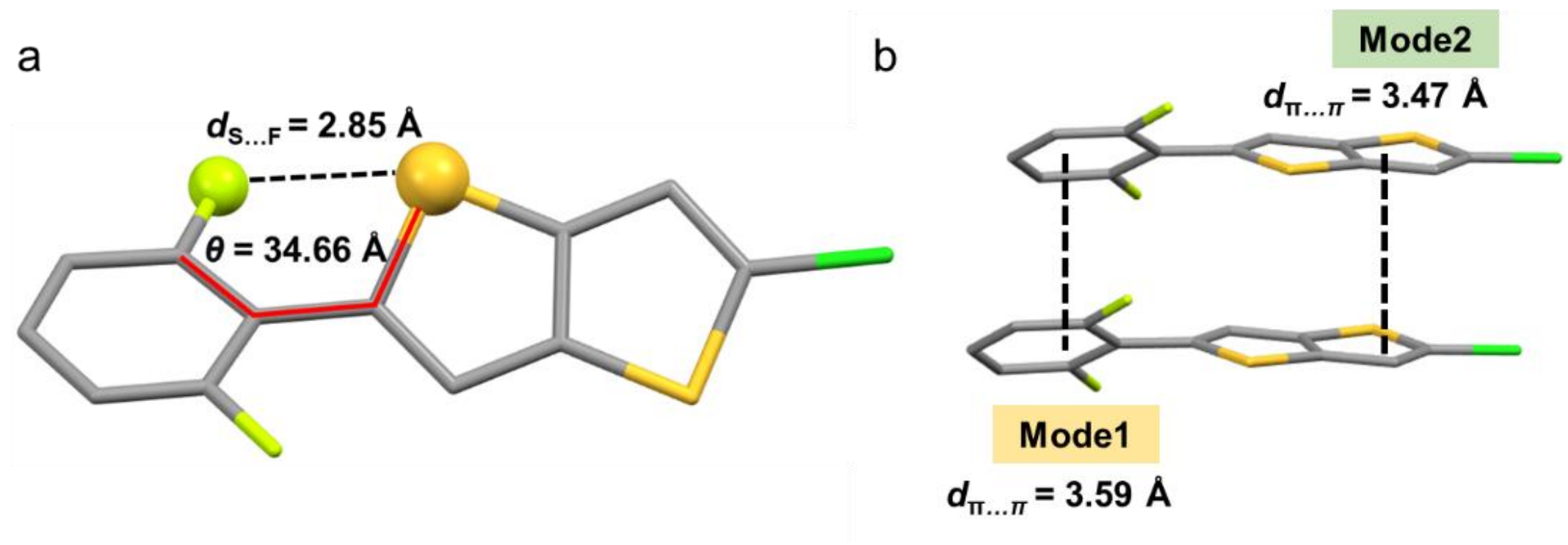

Supplementary Fig. 15. Single-crystal structures and molecular packing of Dr-2.

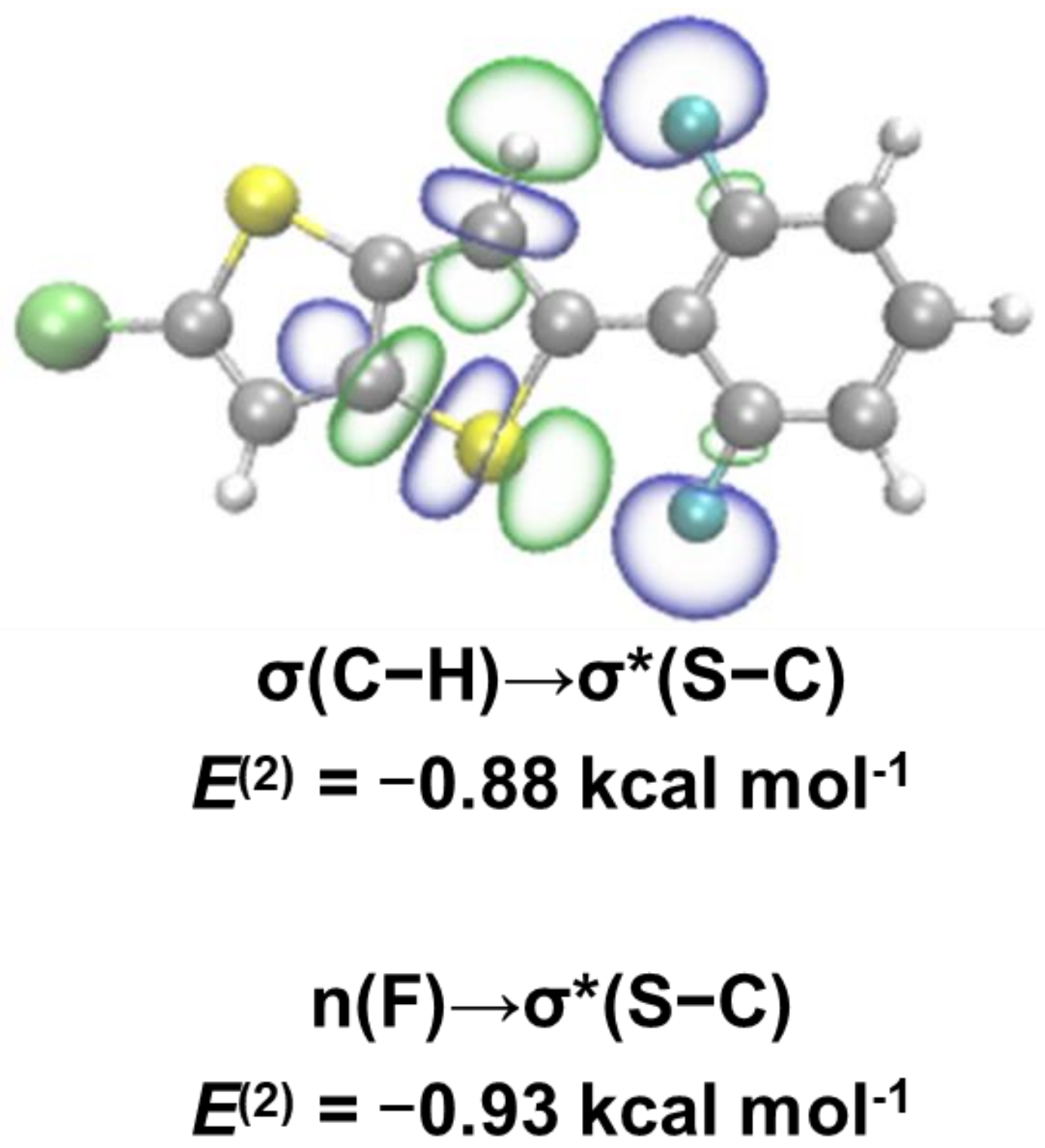

Supplementary Fig. 16. Natural bond orbital (NBO) analysis of Dr-2.

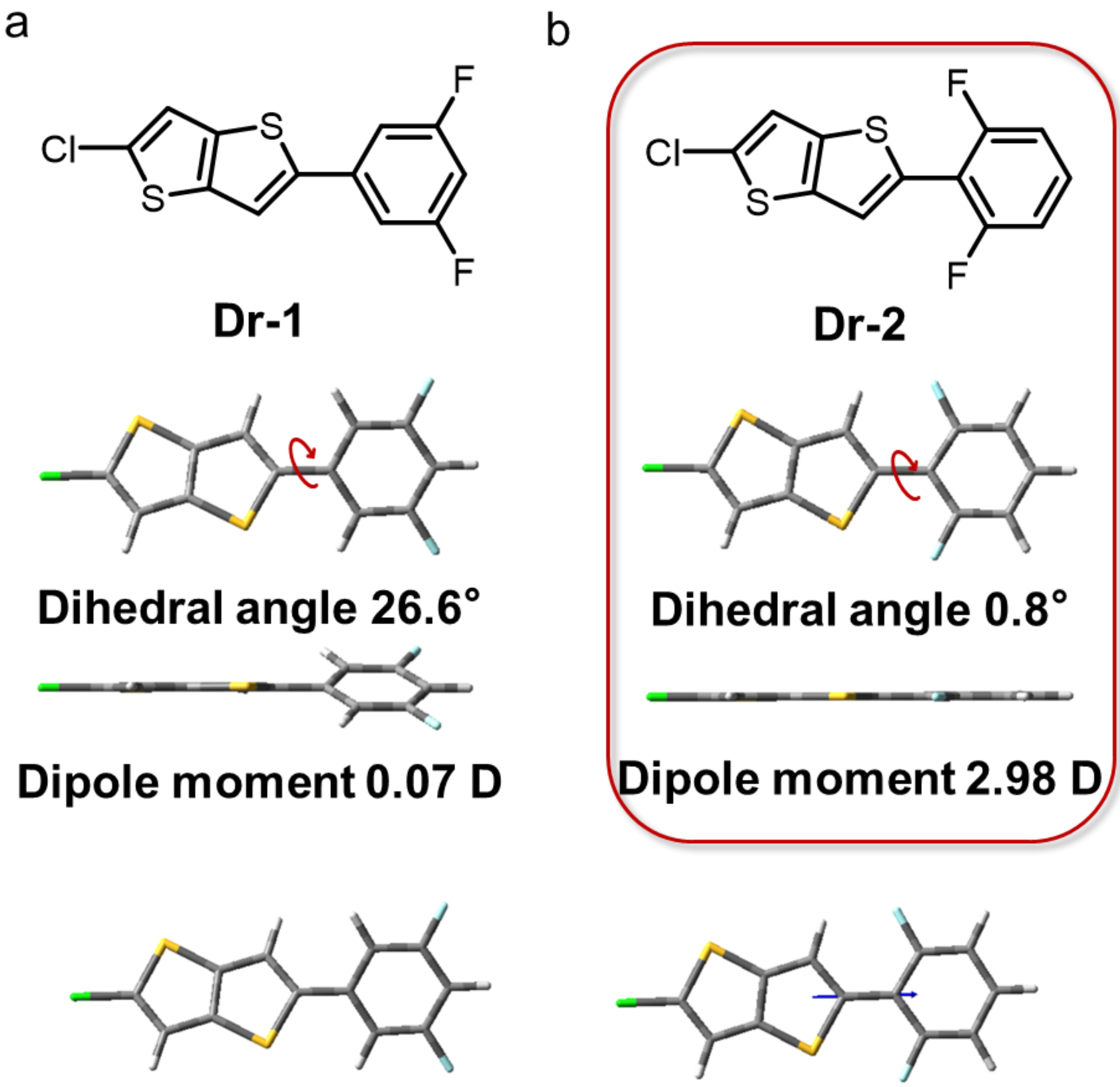

Supplementary Fig. 17. Dipole moment of **a** Dr-1 and **b** Dr-2 from DFT calculation.

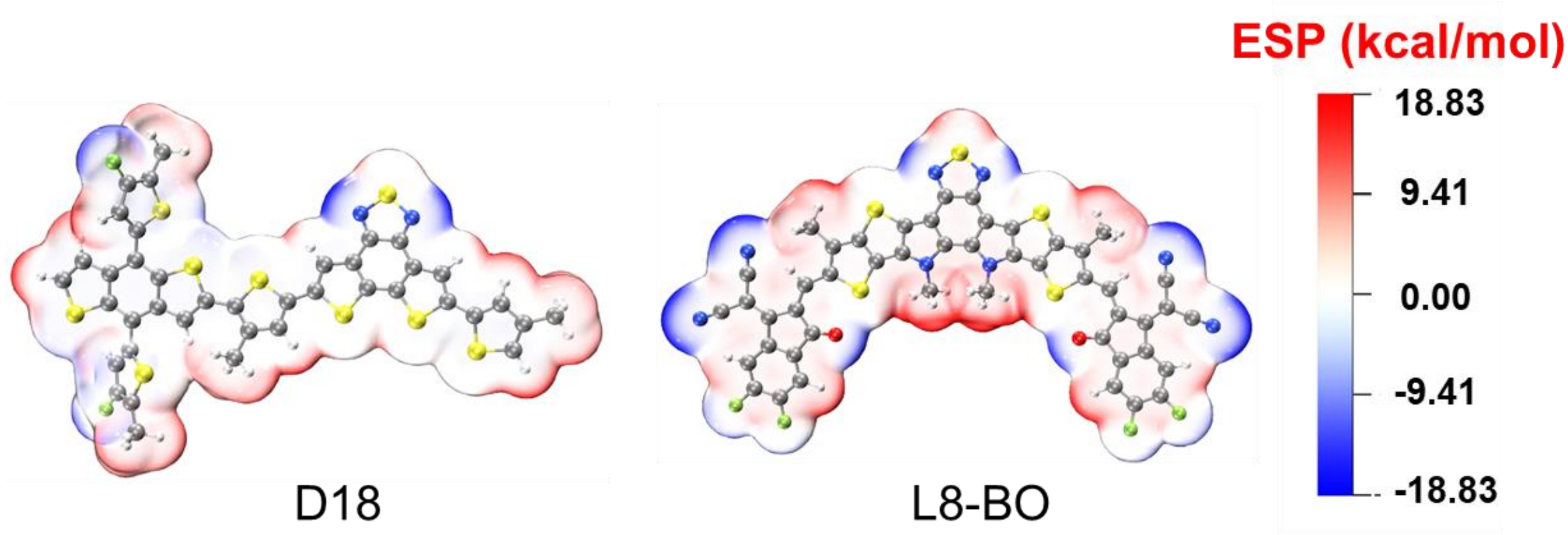

Supplementary Fig. 18. ESP of D18 and L8-BO materials.

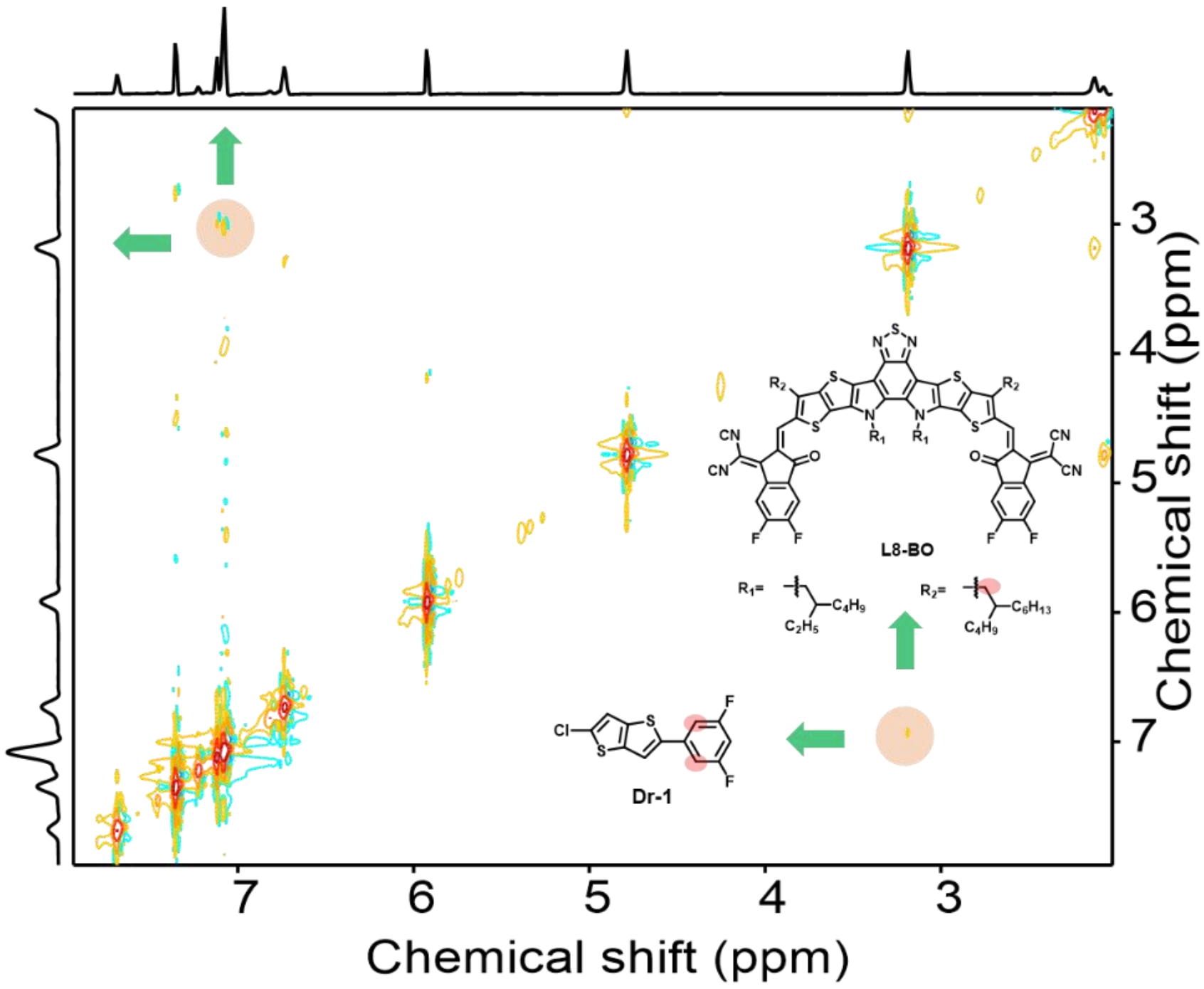

Supplementary Fig. 19. 2D $^1H-^1H$ NMR spectra of L8-BO:Dr-1 in deuterated tetrachloroethane (d-TCE) solution.

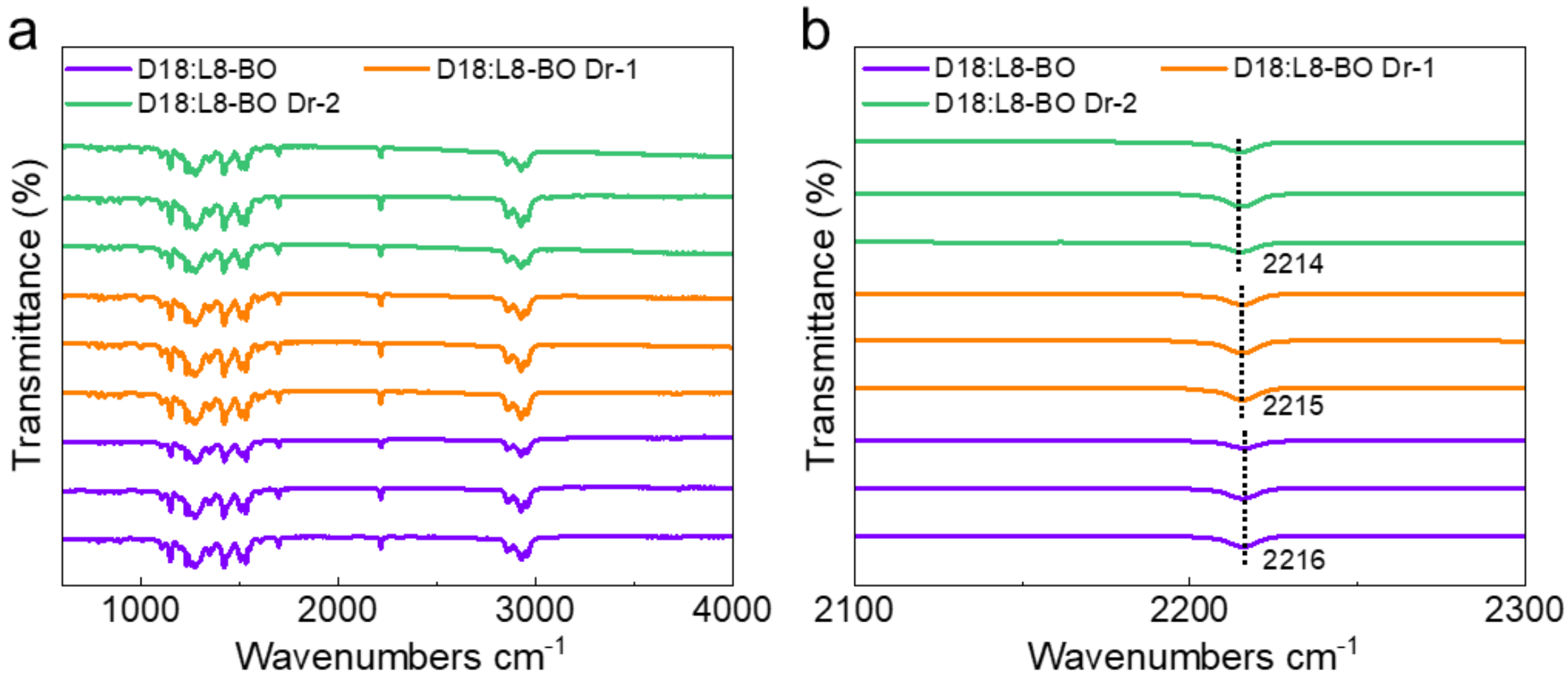

Supplementary Fig. 20. Fourier transform infrared (FTIR) spectra of D18, L8-BO and D18:L8-BO films with/without dielectric regulator.

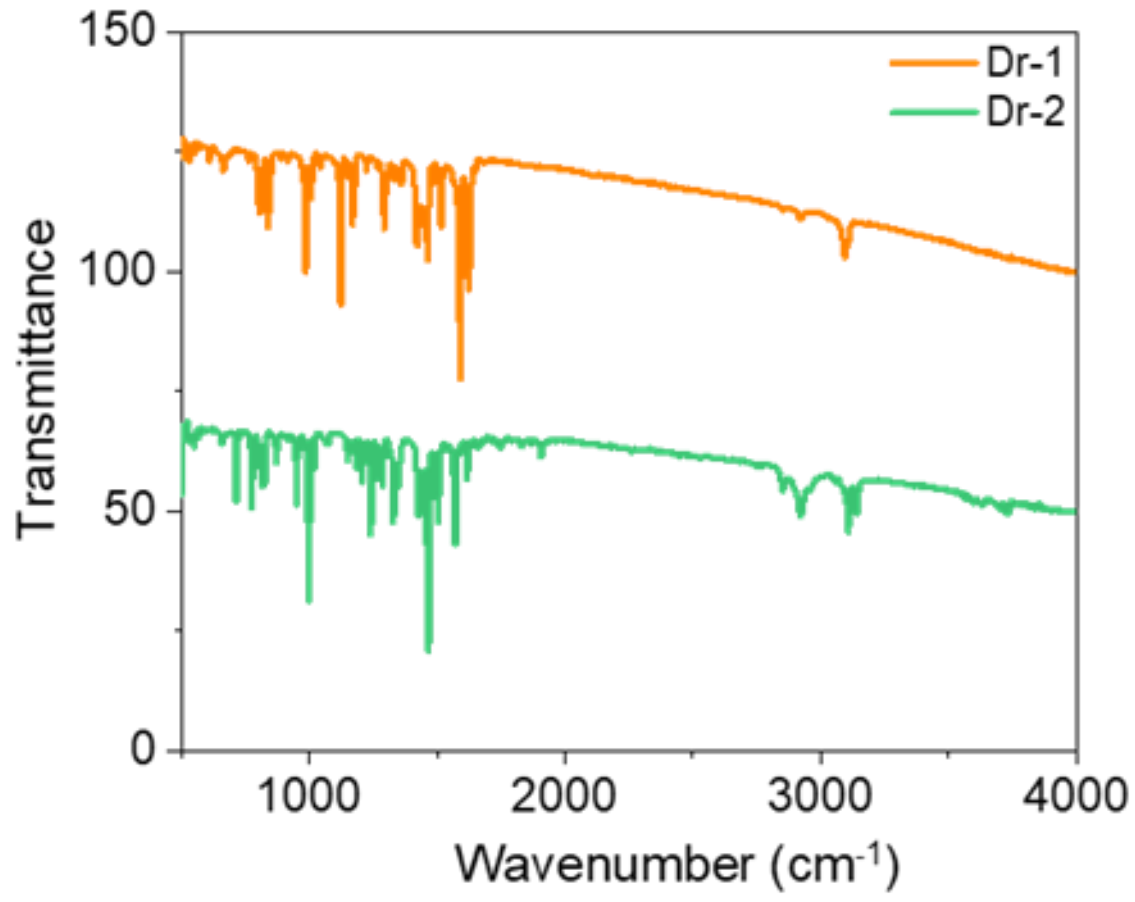

Supplementary Fig. 21. Fourier transform infrared (FTIR) spectra of Dr-1 and Dr-2 materials.

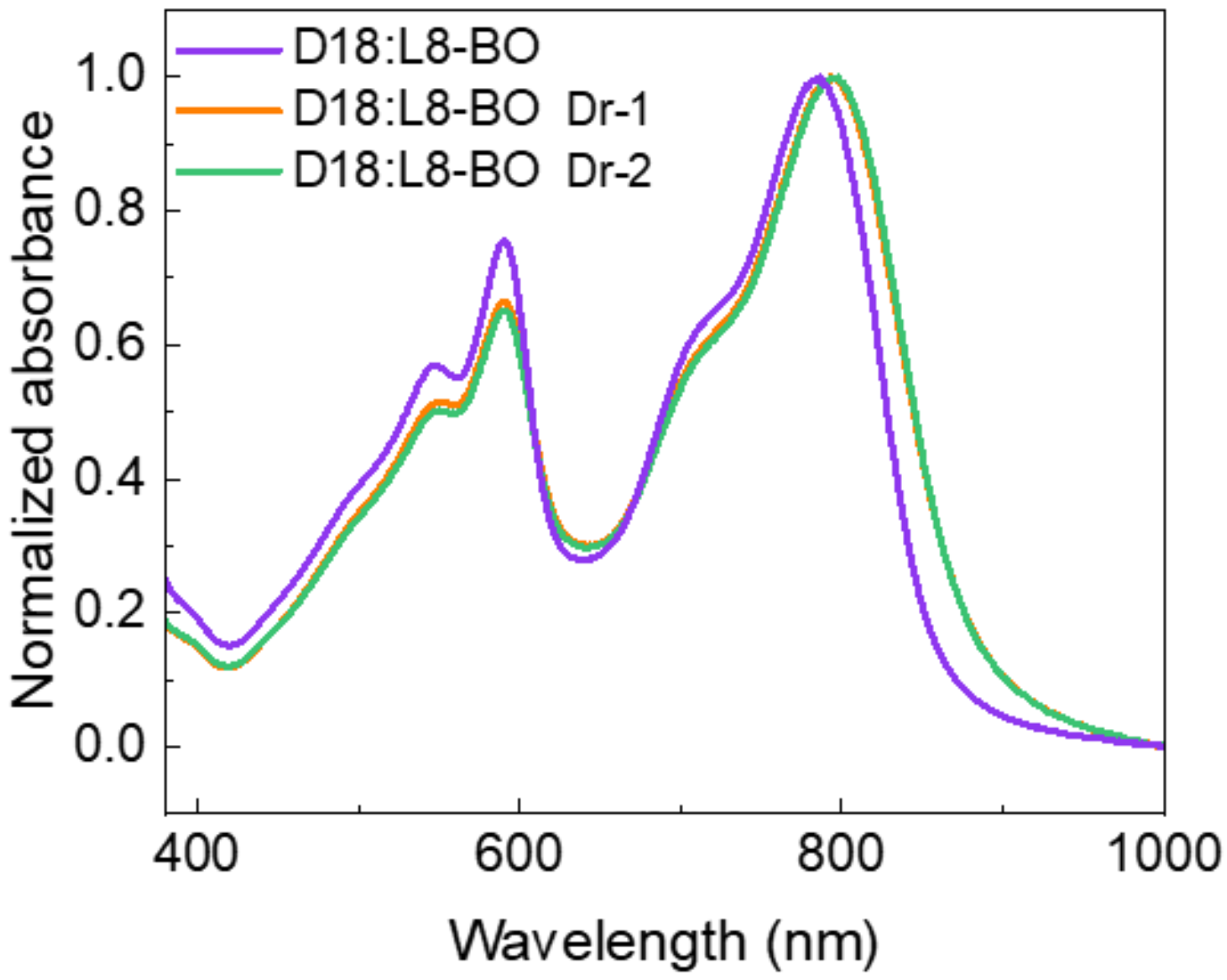

Supplementary Fig. 22. Normalized UV-vis absorption spectra of D18:L8-BO film with/without regulator materials.

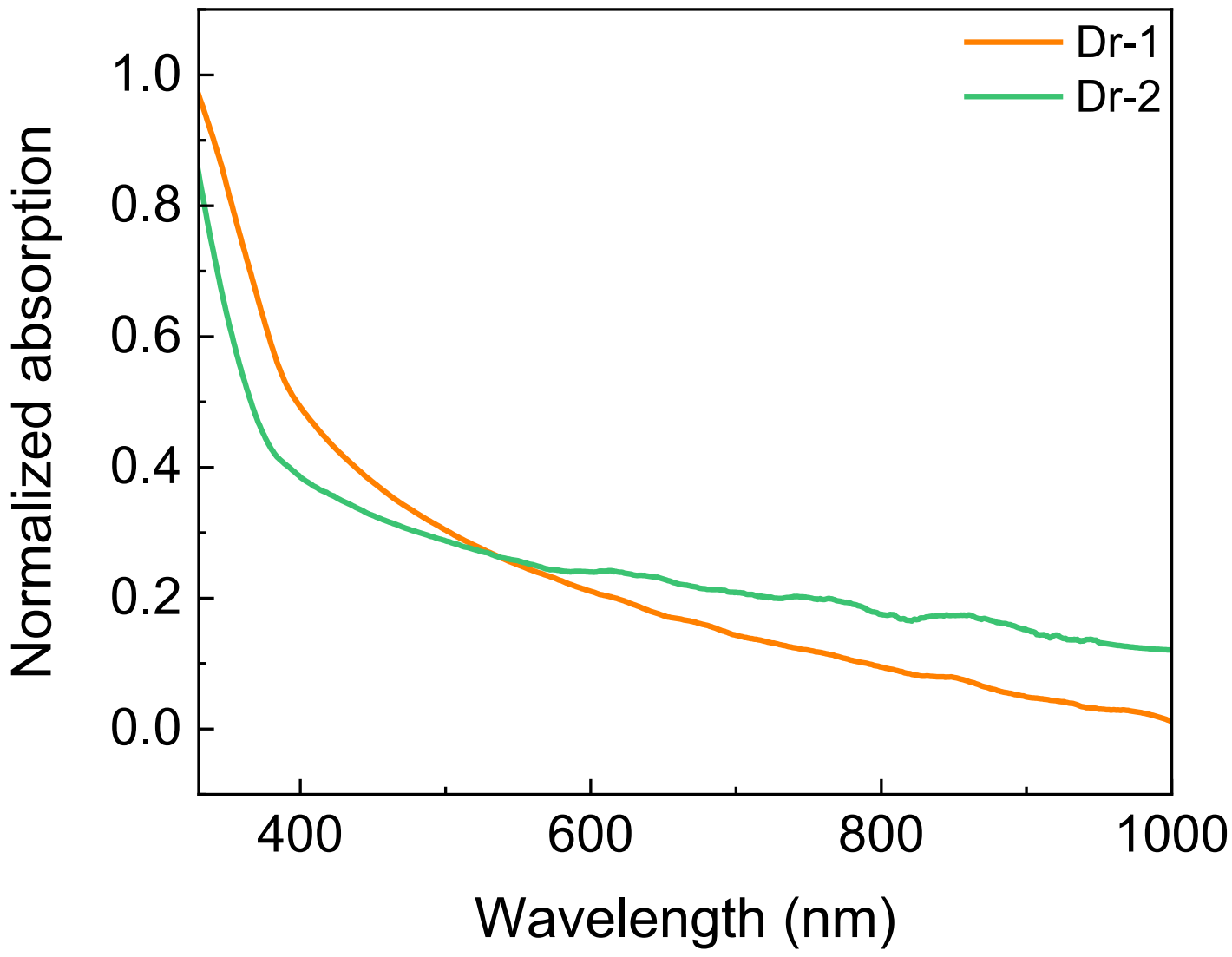

Supplementary Fig. 23. Normalized UV-vis absorption spectra of Dr-1 and Dr-2 films.

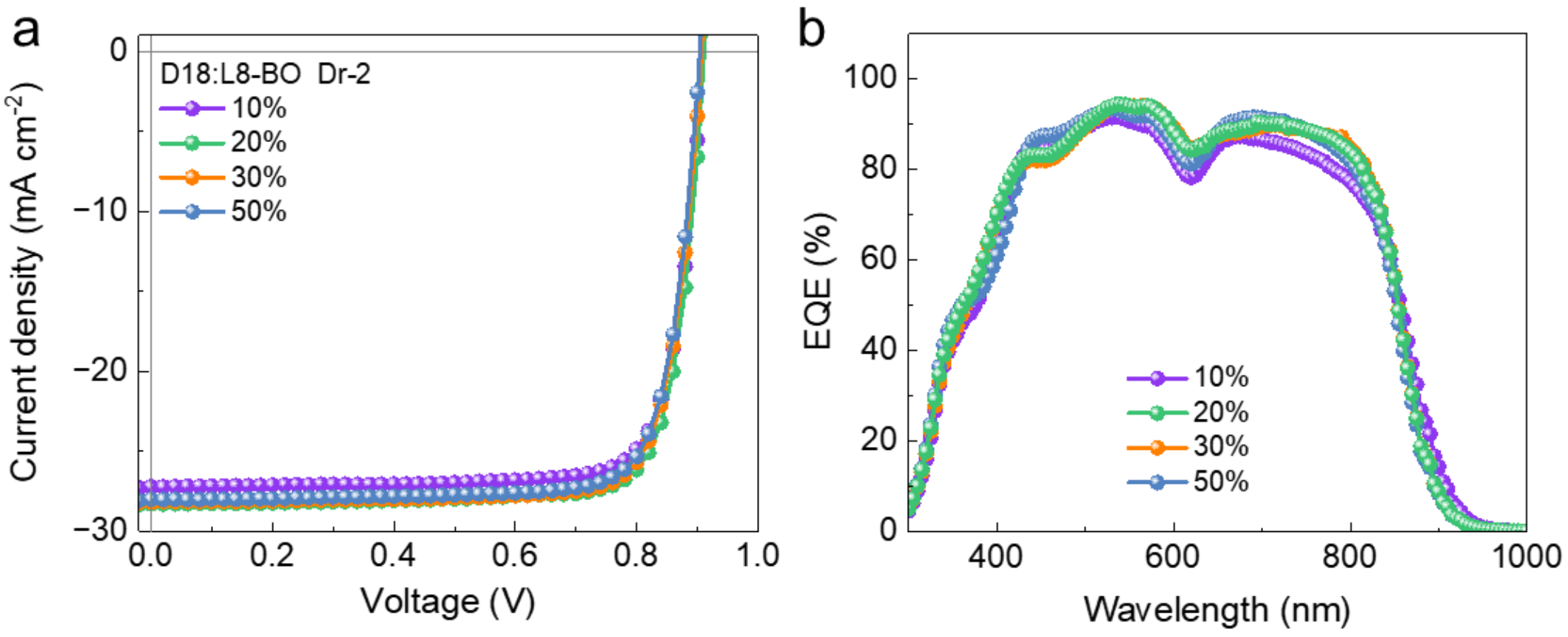

Supplementary Fig. 24. *J*-*V* and EQE curves of devices with different ratio of Dr-2 contents.

华南国家计量测试中心
广东省计量科学研究院
SOUTH CHINA NATIONAL CENTER OF METROLOGY
GUANGDONG INSTITUTE OF METROLOGY

校 准 结 果
RESULTS OF CALIBRATION

证书编号 NYX202600034 Certificate No. 原始记录号 NYX202600034 Record No. 第 3 页, 共 5 页 Page of

1、外观检查 符合要求
Apparent Insp Pass.

2、测试条件： 温度(25±2)℃；辐照度1000W/m²。
Test condition Temperature: (25±2)℃; Irradiance: 1000W/m².

3、电流-电压特性曲线和功率-电压特性曲线：
I-V and P-V curves:

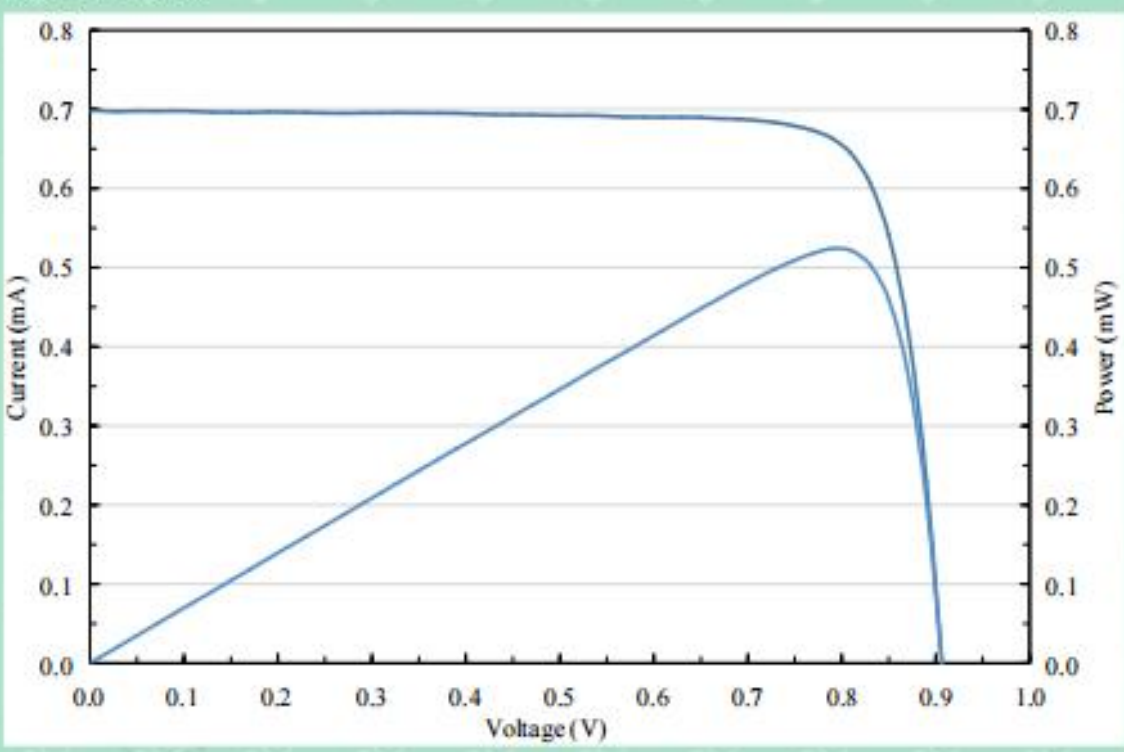

图1 电流-电压特性曲线和功率-电压特性曲线
Figure1 I-V and P-V characteristic curves

4、光电性能参数：
Results of photoelectric properties(Reverse scan and forward scan):

表1(Table 1)

面积	短路电流密度 J_{sc}	短路电流 I_{sc}	开路电压 V_{oc}	填充因子 FF	最大功率 P_m	最佳工作电流 I_m	最佳工作电压 V_m	转换效率 η
Area	Short ciruit current density	Short ciruit current	Open circuit voltage	Fill factor	Maximum power	Optimum working current	Optimum working voltage	Efficiency
cm^2	mA/cm^2	mA	V	%	mW	mA	V	%
0.0257	27.17	0.698	0.906	82.78	0.524	0.663	0.790	20.38

Supplementary Fig. 25. Certification of the photovoltaic efficiency of the D18:L8-BO device with the treatment of Dr-2 from South China National Center of Metrology.

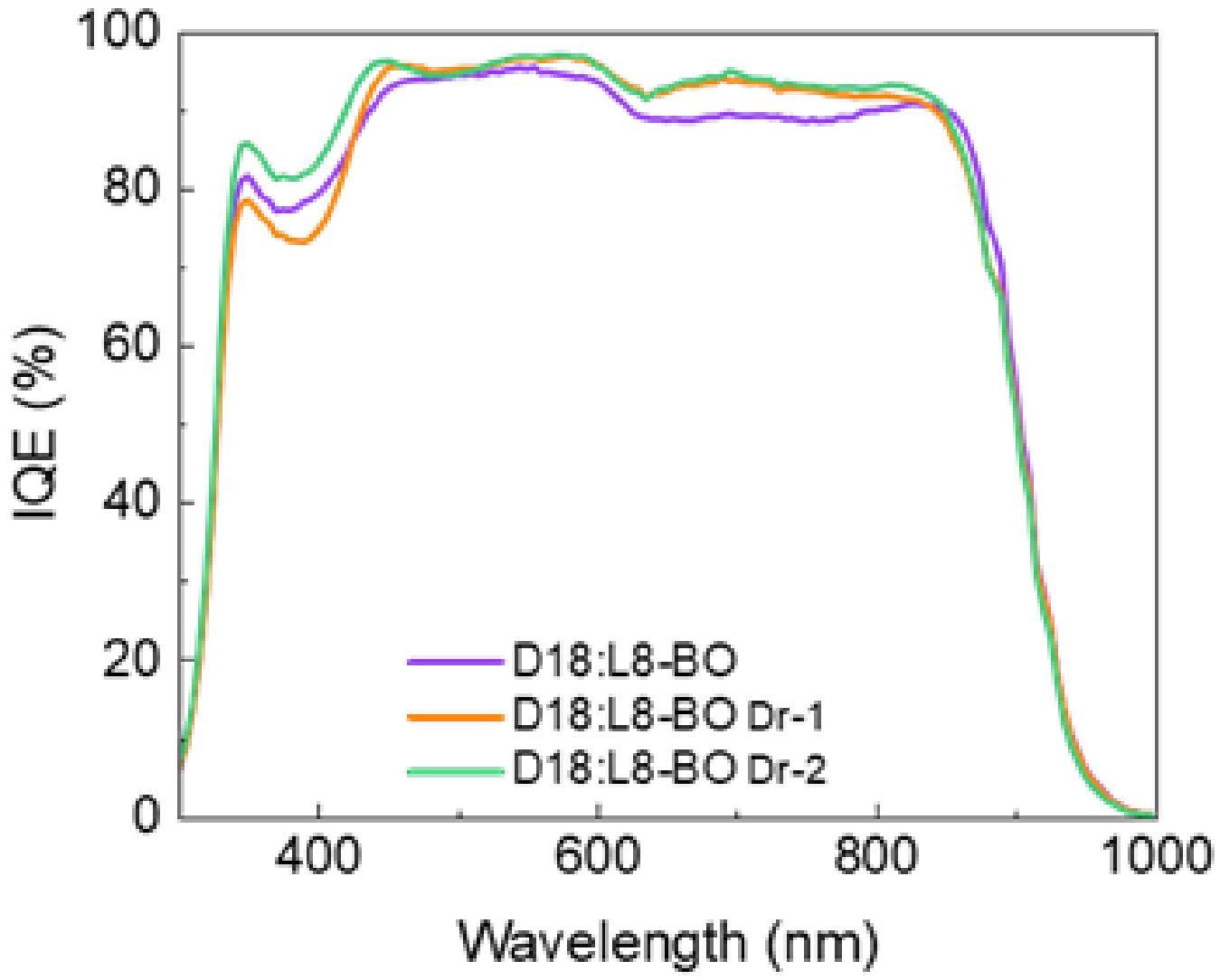

Supplementary Fig. 26. The internal quantum efficiency curves of devices with/without regulators.

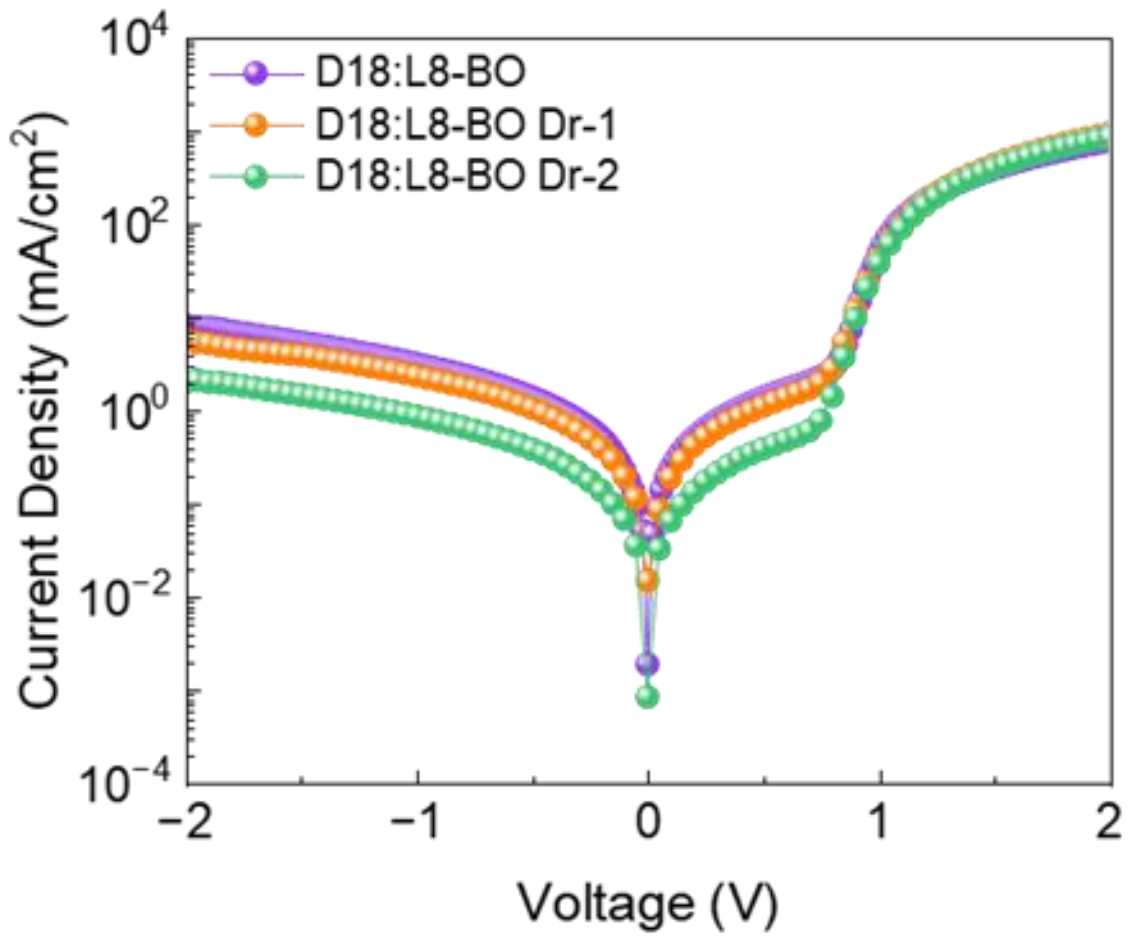

Supplementary Fig. 27. Dark current curves of different device.

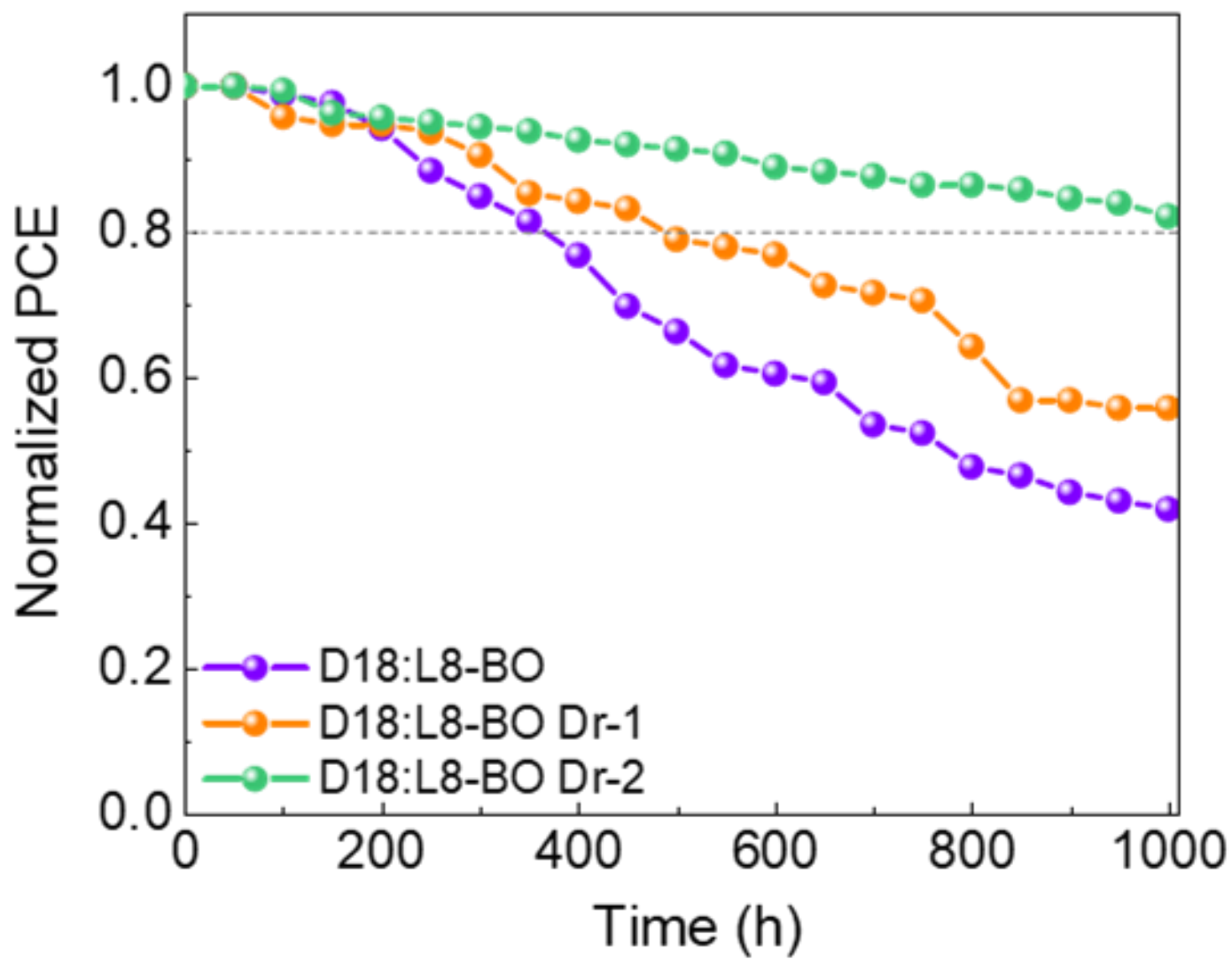

Supplementary Fig. 28. Photostability of encapsulated devices measured using maximum power point tracking (MPPT) under simulated AM1.5G irradiation (100 mW cm^{-2}).

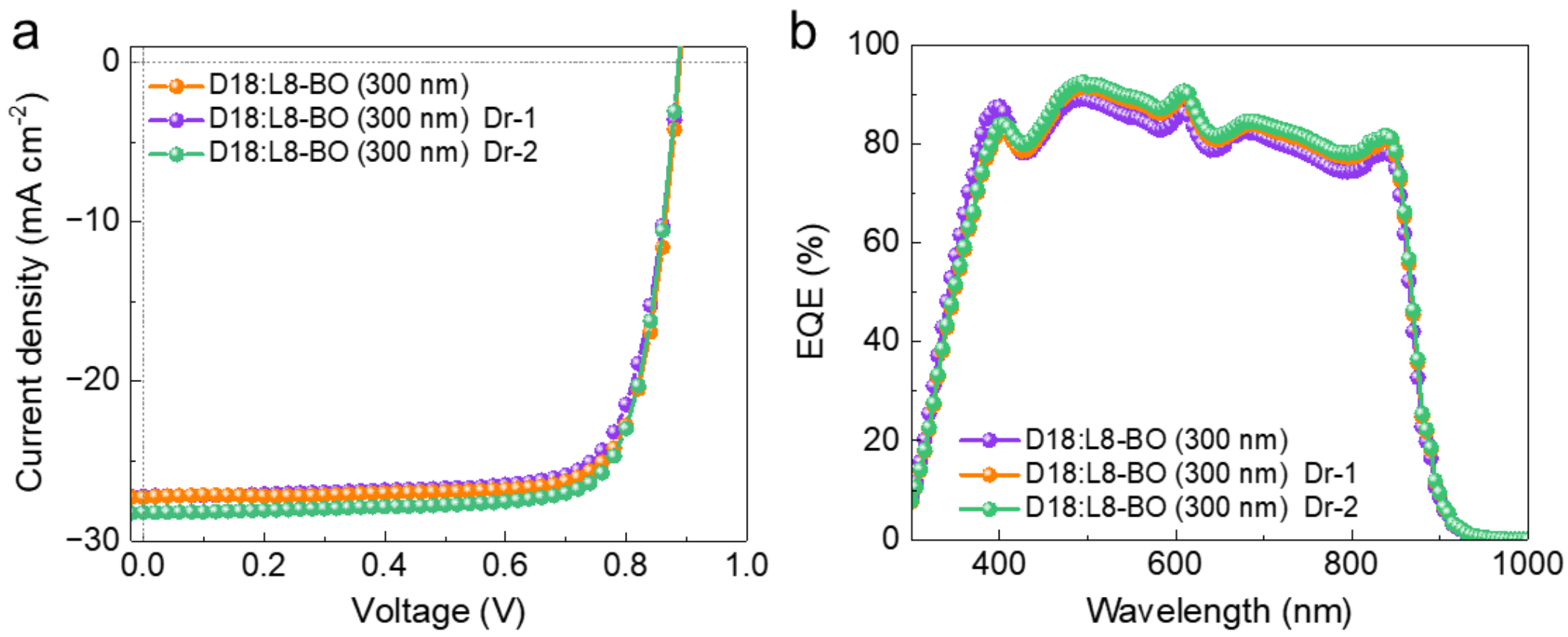

Supplementary Fig. 29. a *J-V* and **b** EQE curves of D18:L8-BO systems (300 nm) with/without dielectric regulators.

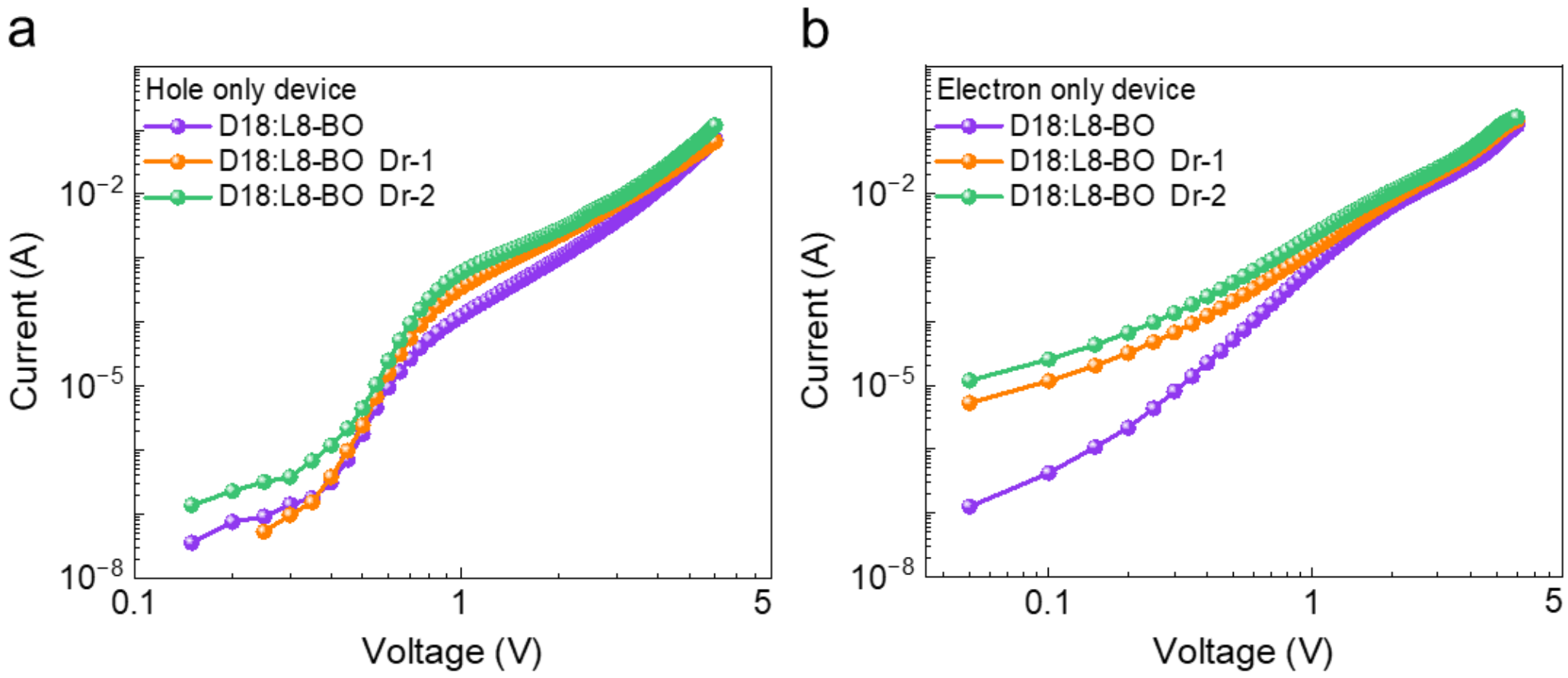

Supplementary Fig. 30. *I*-*V* traces of **a** hole only devices. **b** electron only devices.

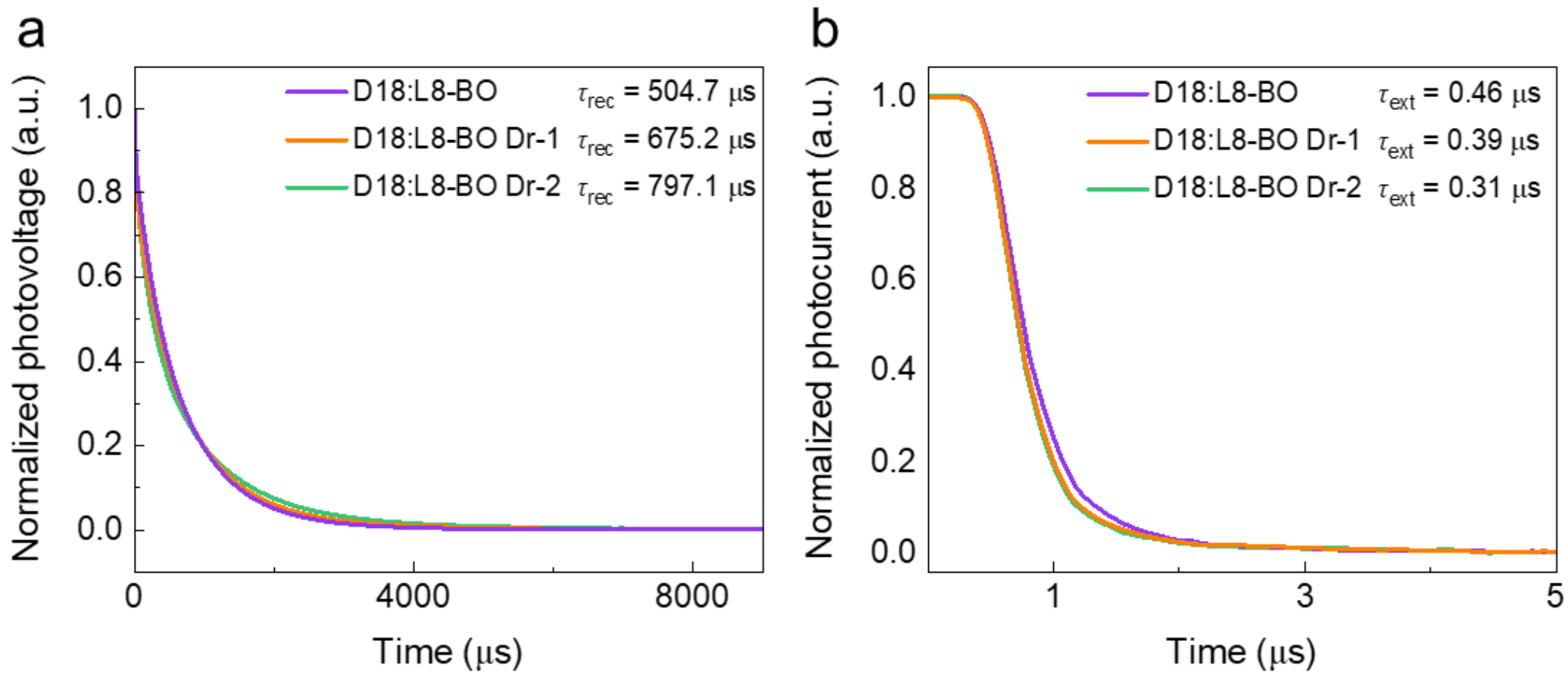

Supplementary Fig. 31. a TPV, and b TPC curves of device with/without regulators.

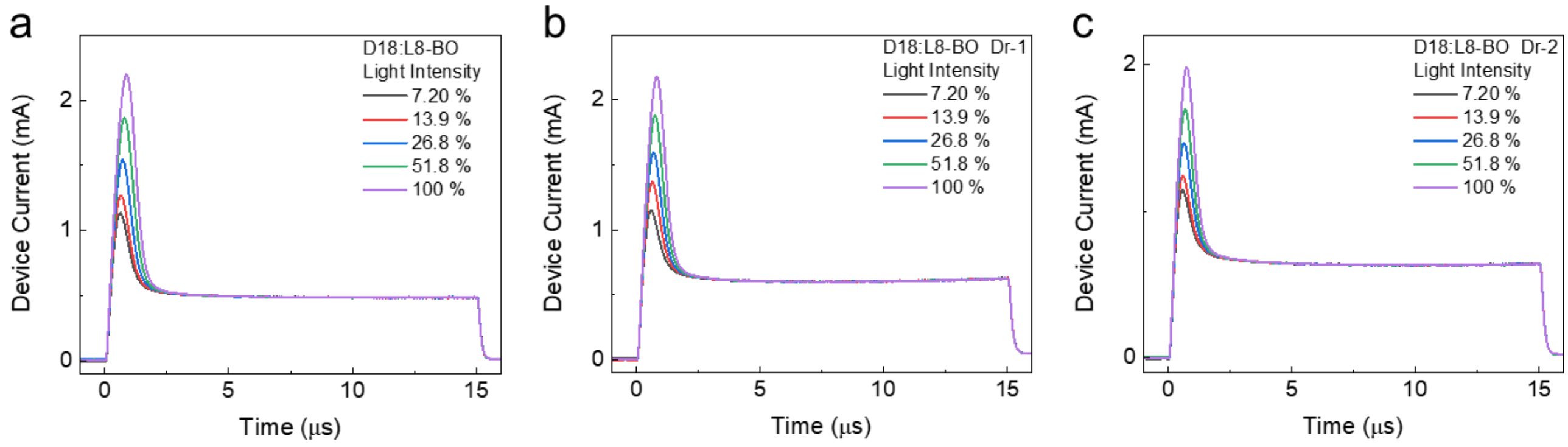

Supplementary Fig. 32. Photo-CELIV curve for devices with/without dielectric regulators under various light intensities.

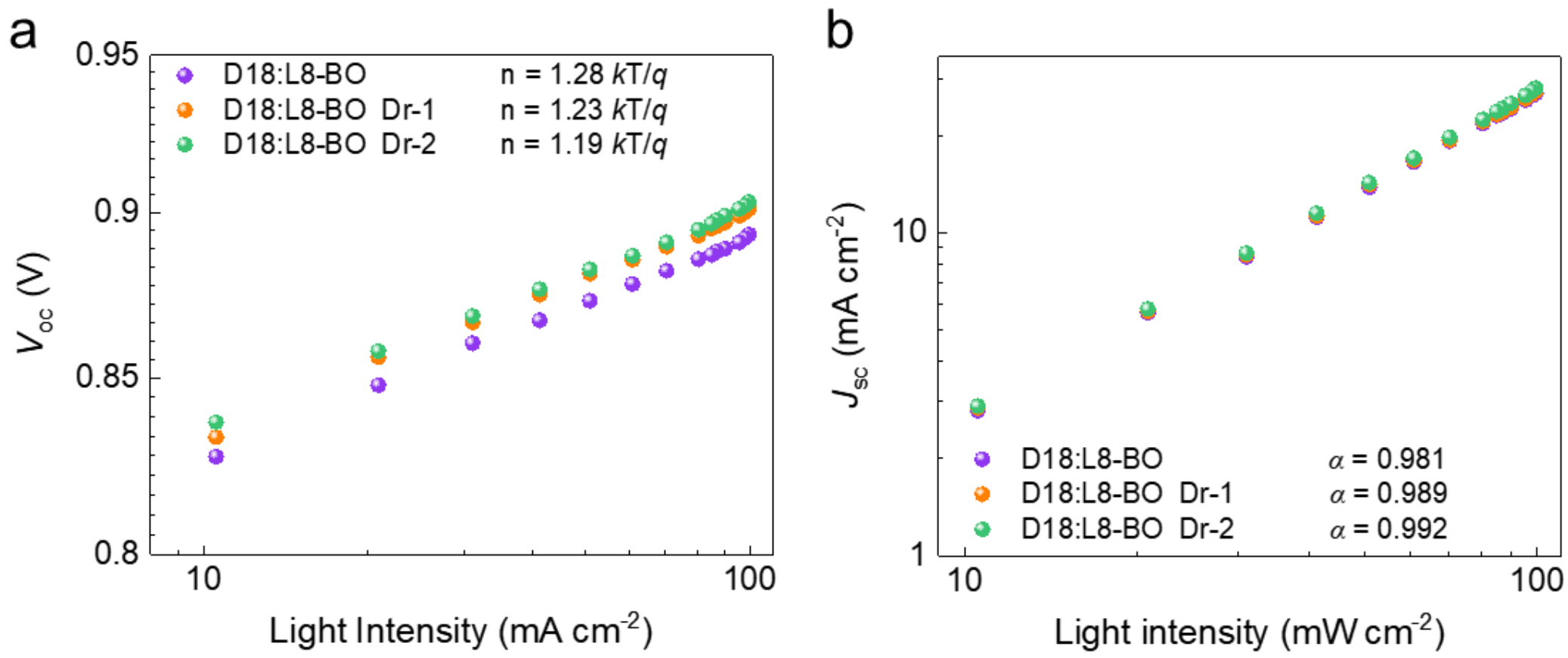

Supplementary Fig. 33. a V_{oc} versus light intensity plots and **b** J_{sc} versus light intensity plots of OSCs.

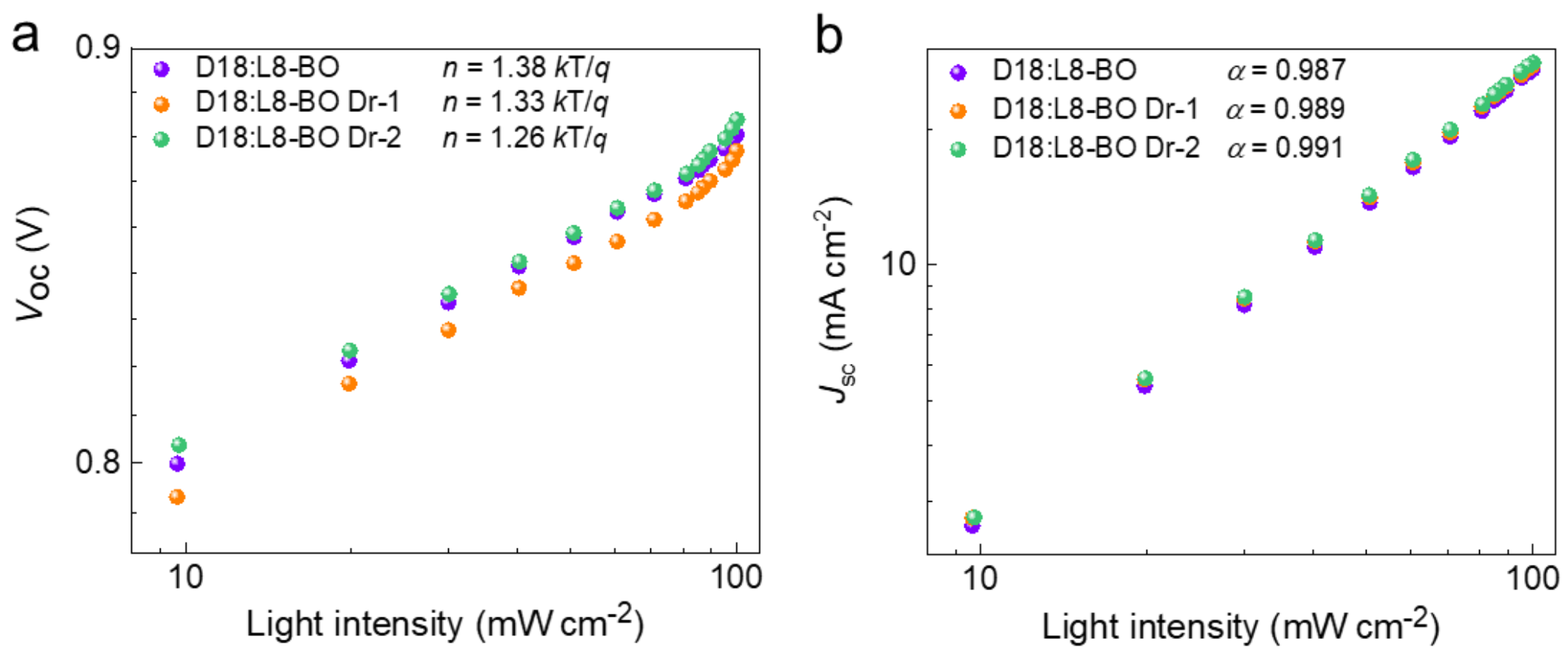

Supplementary Fig. 34. a V_{oc} versus light intensity plots and b J_{sc} versus light intensity plots of D18:L8-BO systems (300 nm) with/without dielectric regulators.

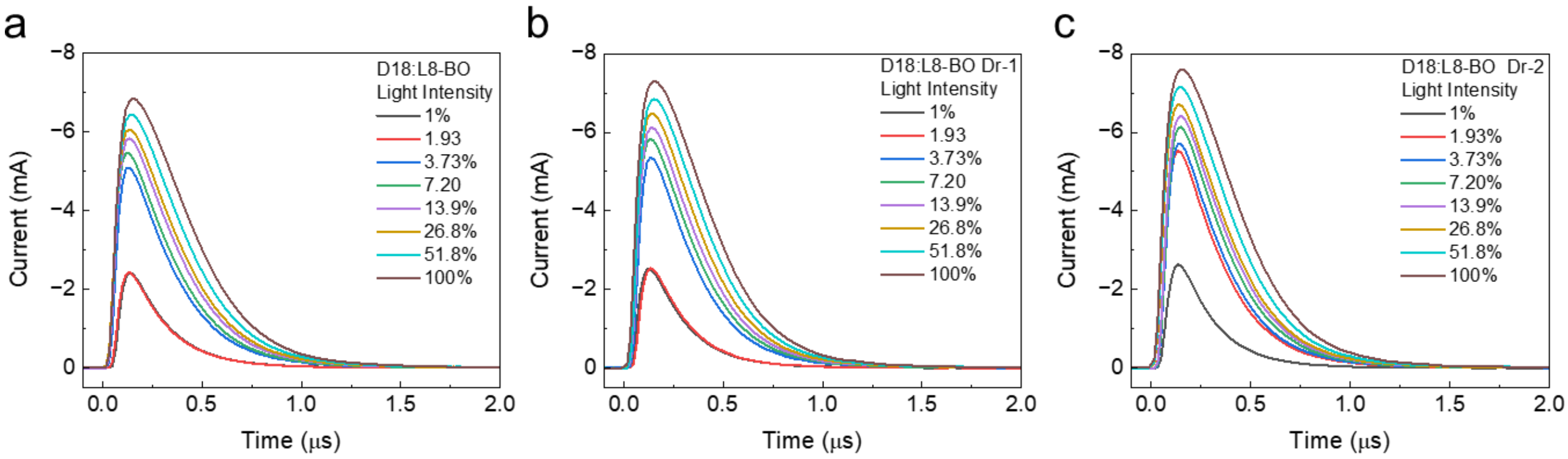

Supplementary Fig. 35. The induced extraction current transient under various illumination at corresponding open-circuit conditions for different devices.

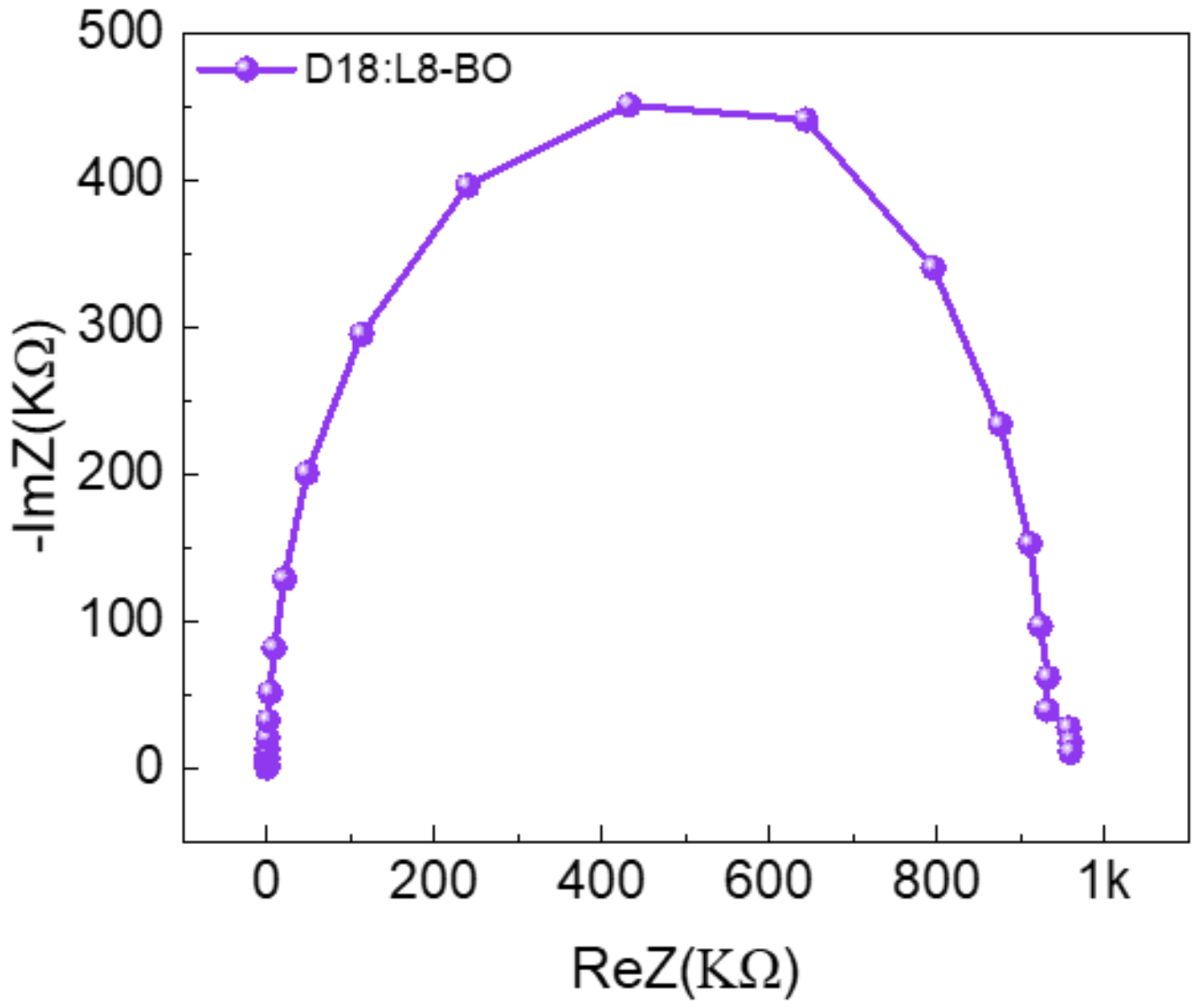

Supplementary Fig. 36. The Nyquist plots of D18:L8-BO.

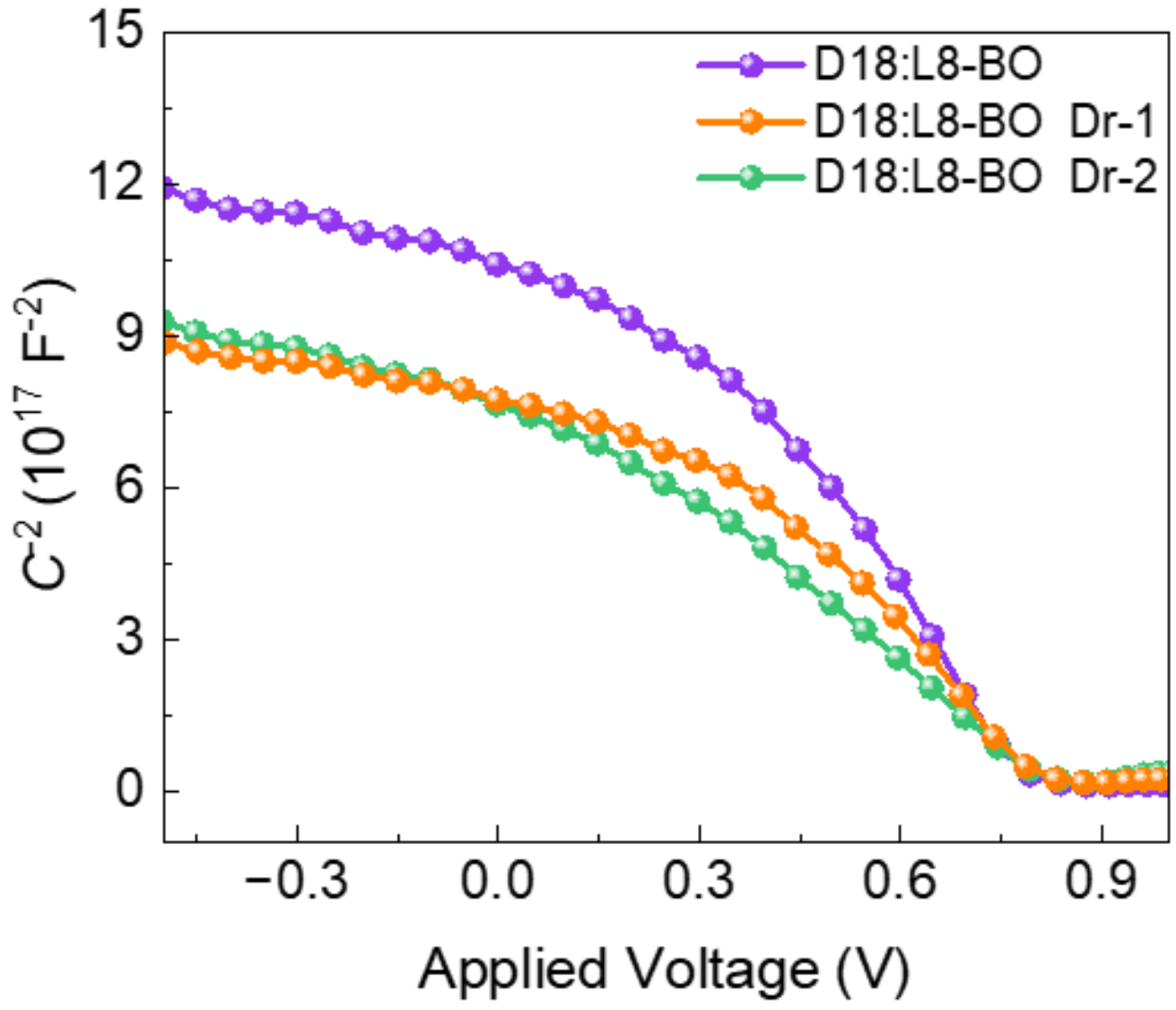

Supplementary Fig. 37. The built-in potential of devices measured following the Mott-Schottky method in the dark.

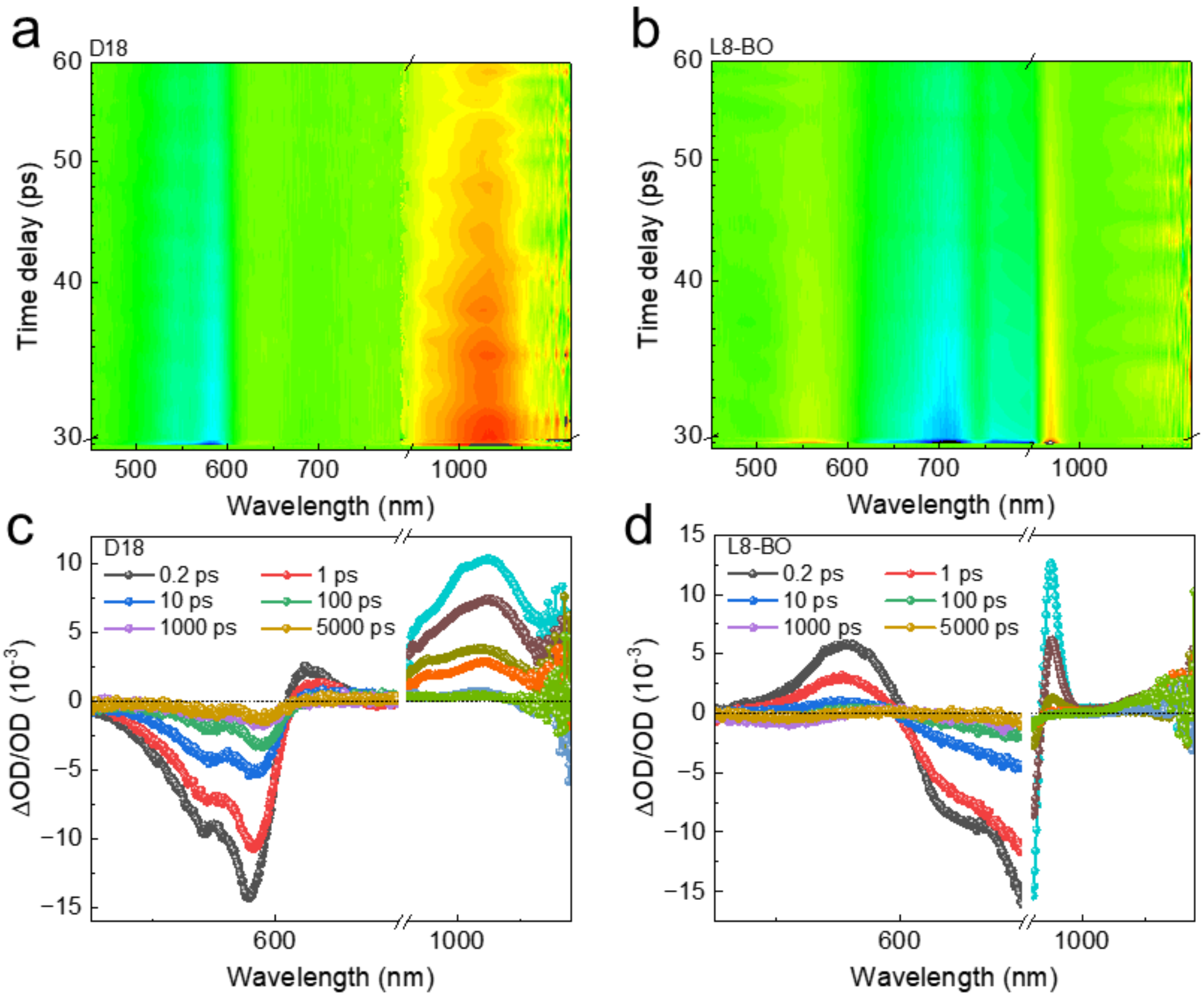

Supplementary Fig. 38. 2D color plots of TA spectra of **a** D18 and **b** L8-BO films. **c-d** corresponding TA spectra recorded at different time delays.

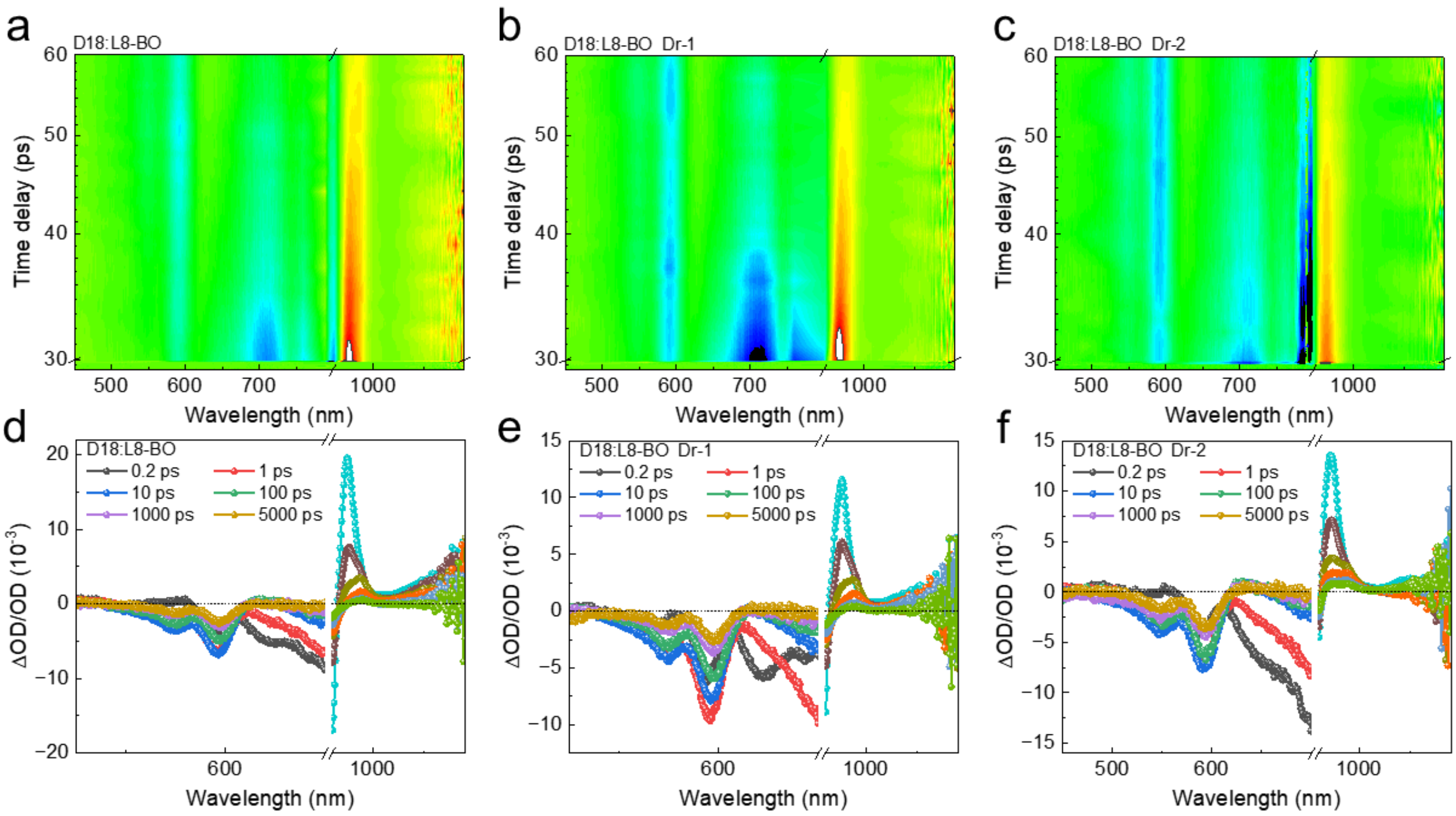

Supplementary Fig. 39. 2D color plots of TA spectra of **a** D18:L8-BO. **b** D18:L8-BO (Dr-1). **c** D18:L8-BO (Dr-2) films. **d-f** corresponding TA spectra recorded at different time delays.

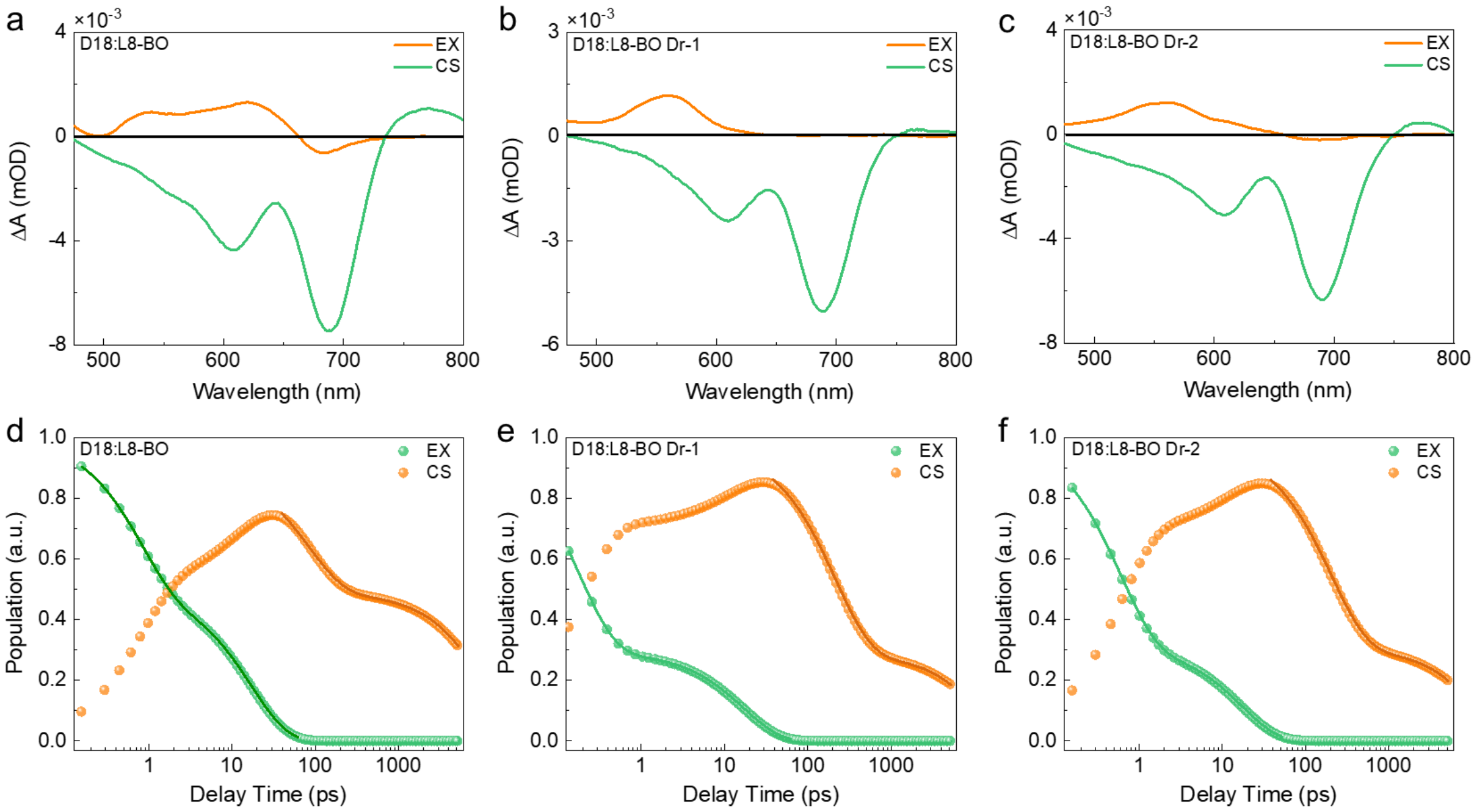

Supplementary Fig. 40. a-c EX and CS spectra and d-f their kinetics extracted by SVD of D18:L8-BO film with/without regulators.

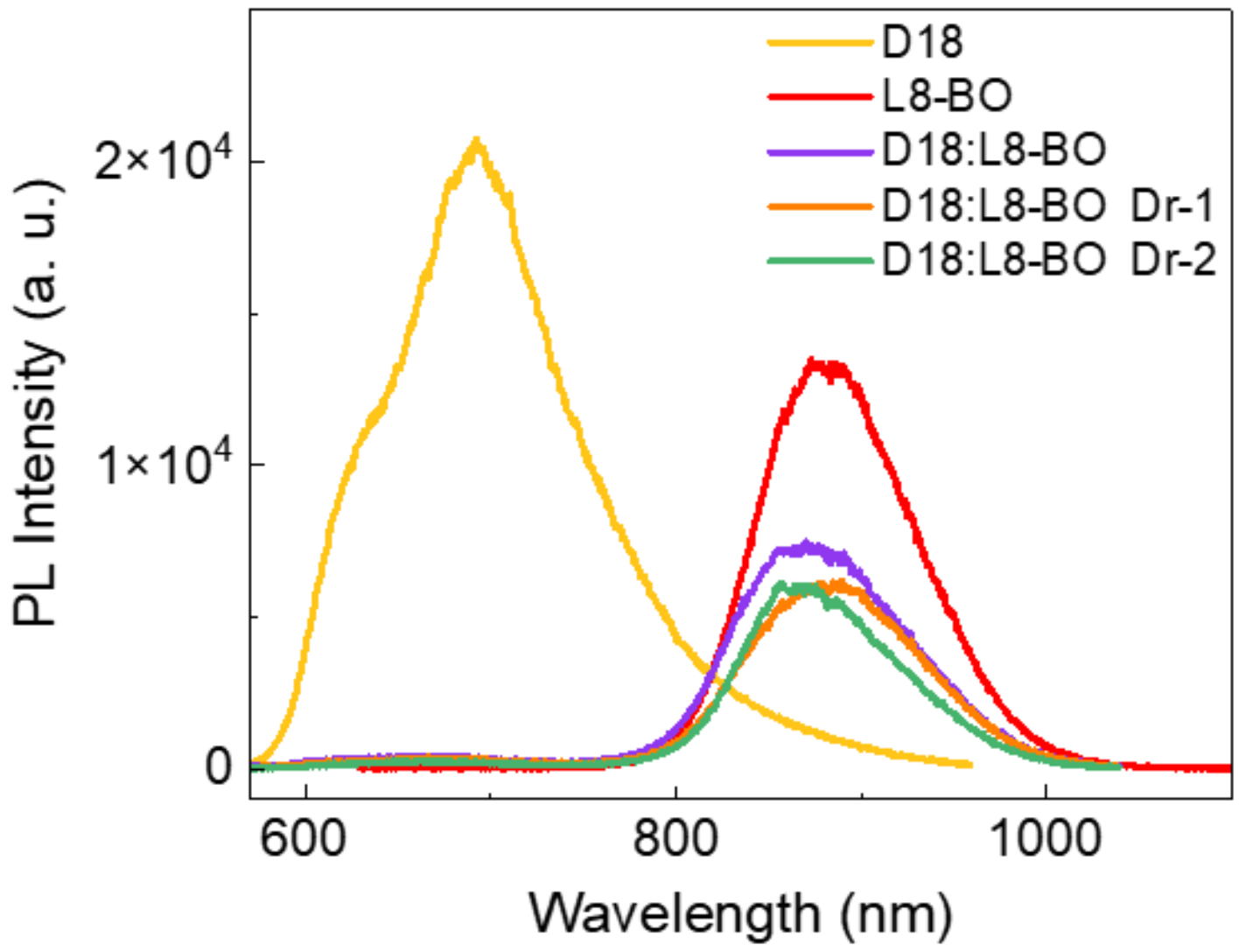

Supplementary Fig. 41. (a) PL spectra of D18, L8-BO, D18:L8-BO, D18:L8-BO (Dr-1) and (Dr-2) films.

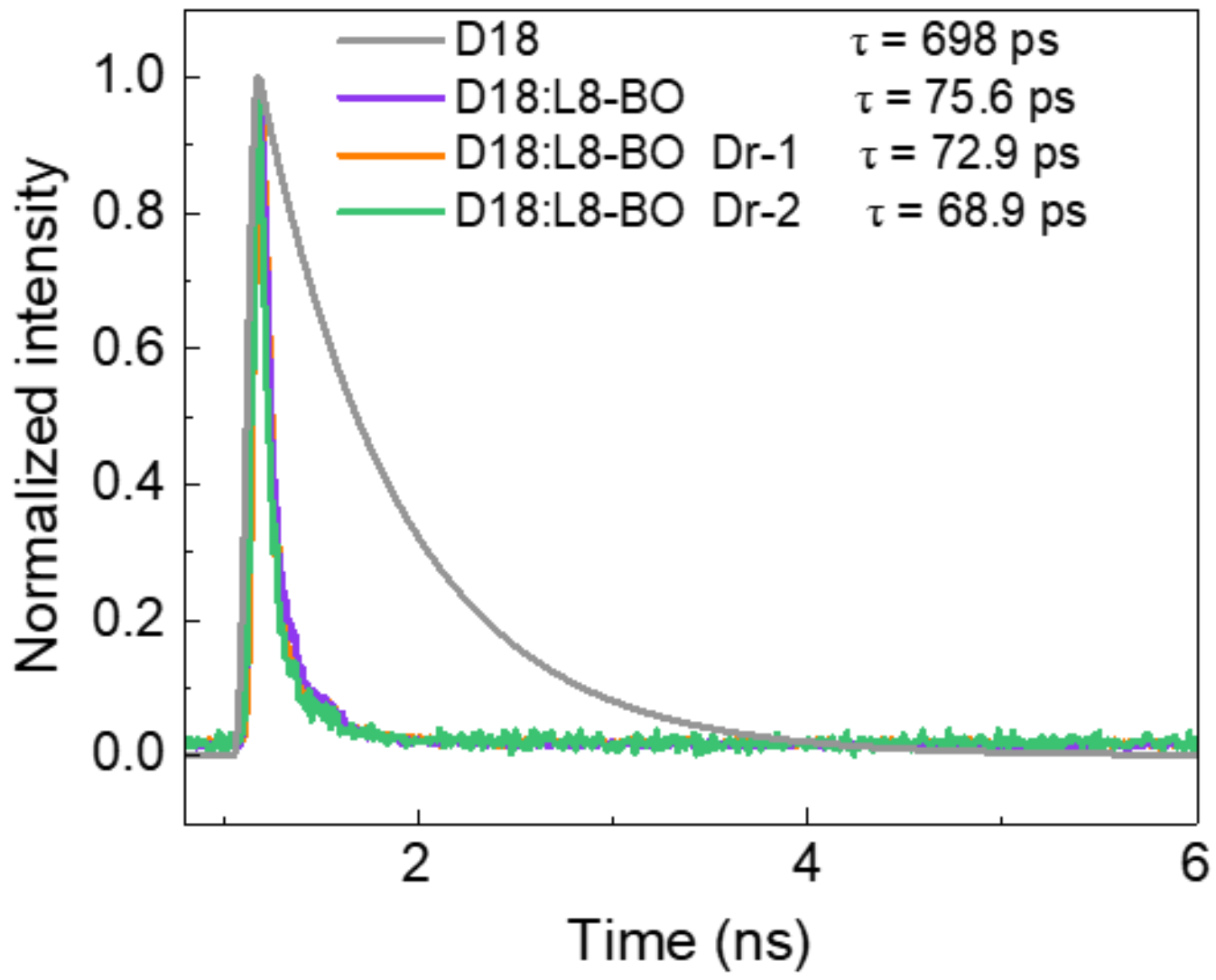

Supplementary Fig. 42. Time-resolved photoluminescence spectra of D18, D18:L8-BO, D18:L8-BO (treated with Dr-1) and D18:L8-BO (treated with Dr-2) films, respectively.

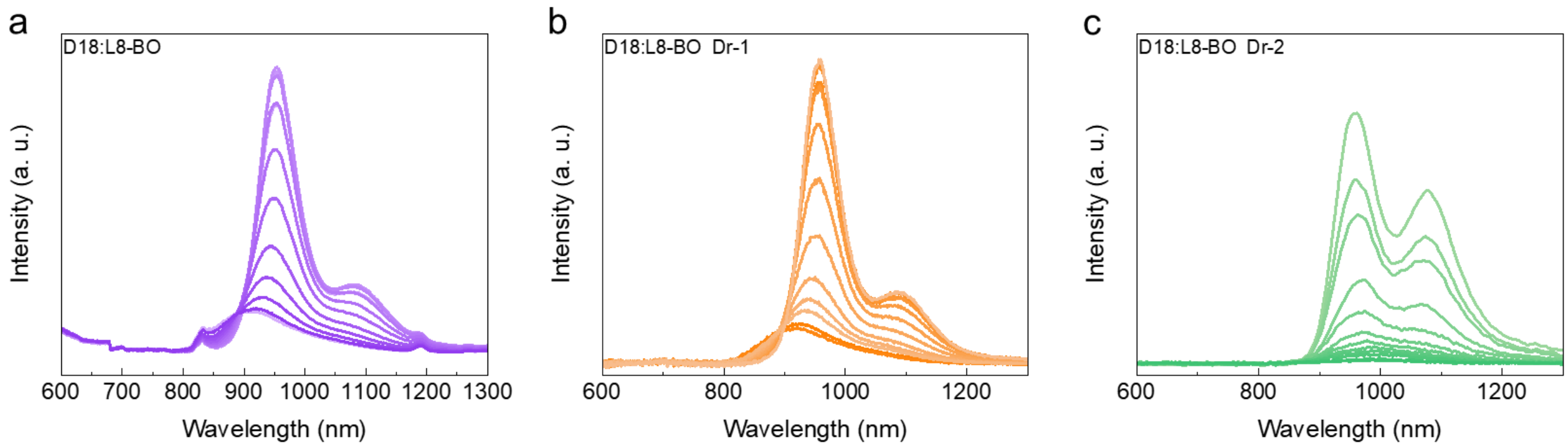

Supplementary Fig. 43. Temperature dependent photoluminescence of D18:L8-BO, D18:L8-BO (treated with Dr-1) and D18:L8-BO (treated with Dr-2) films, respectively.

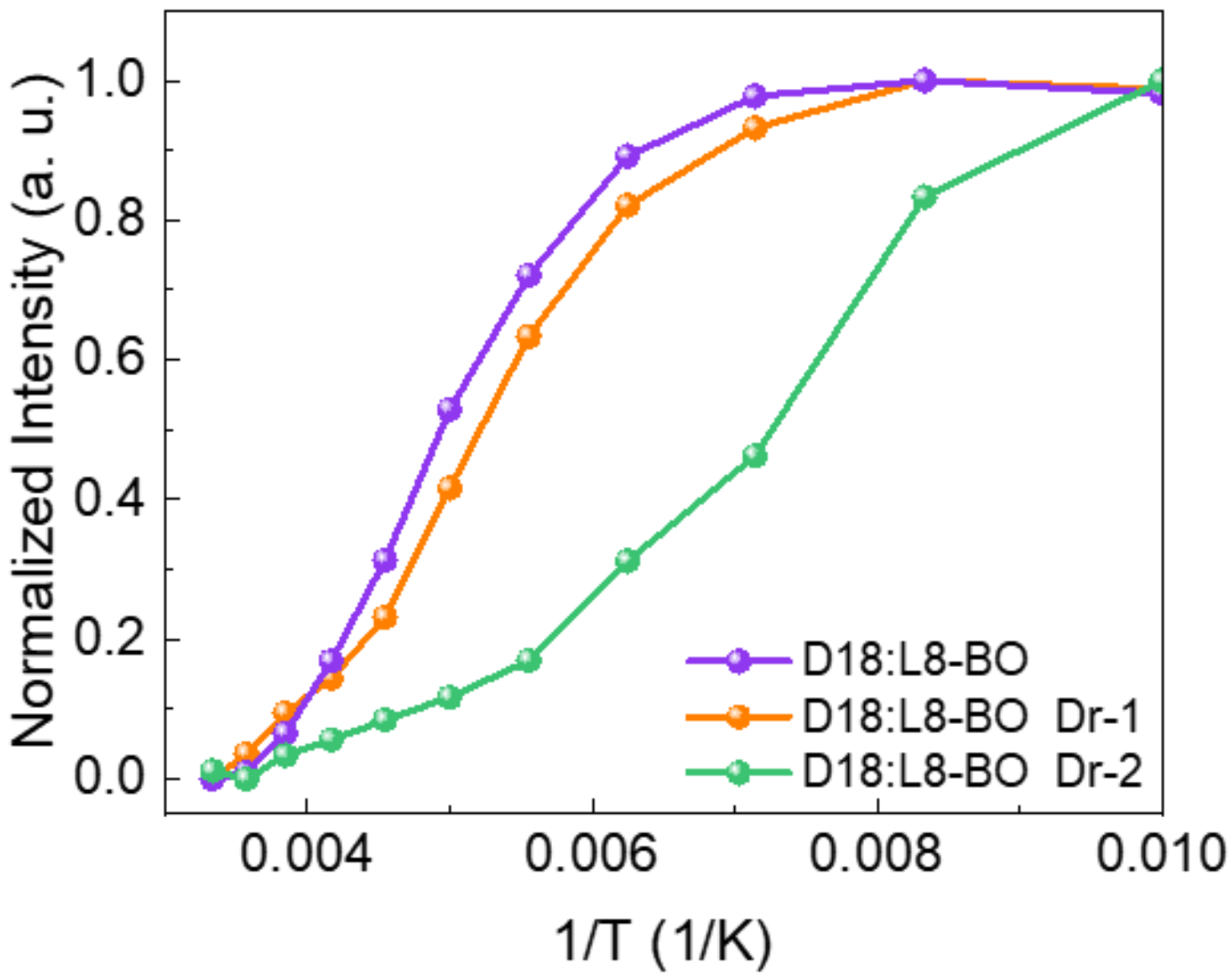

Supplementary Fig. 44. Exciton binding energies of D18:L8-BO, D18:L8-BO (treated with Dr-1) and D18:L8-BO (treated with Dr-2) films measured by temperature dependent photoluminescence.

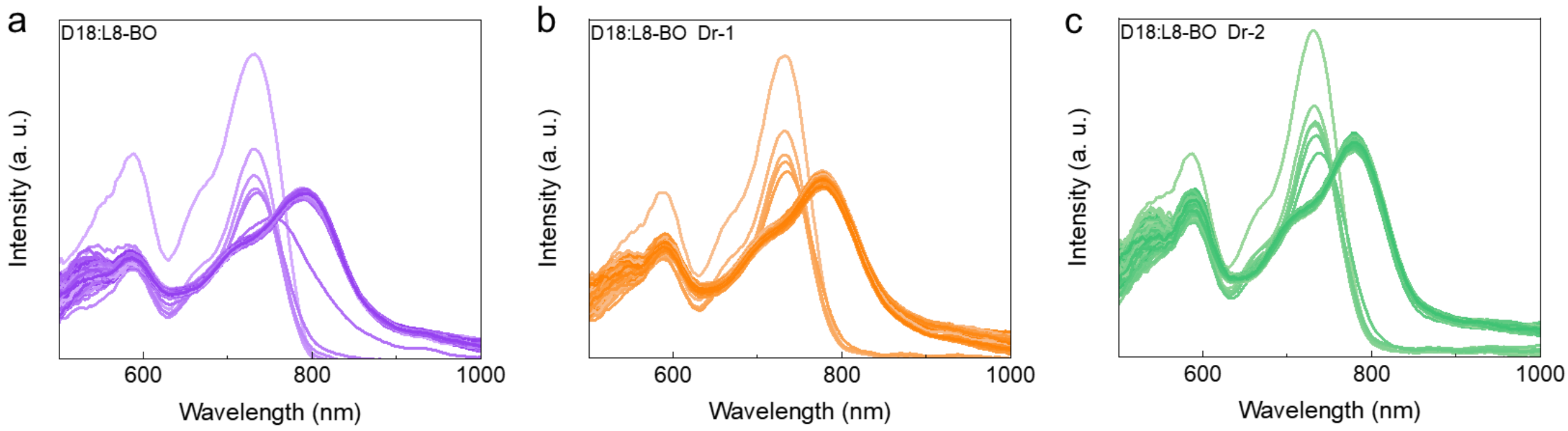

Supplementary Fig. 45. The UV–vis absorption spectra evolution as a function of time of **a** D18:L8-BO, **b** D18:L8-BO (Dr-1) and **c** D18:L8-BO (Dr-2), respectively.

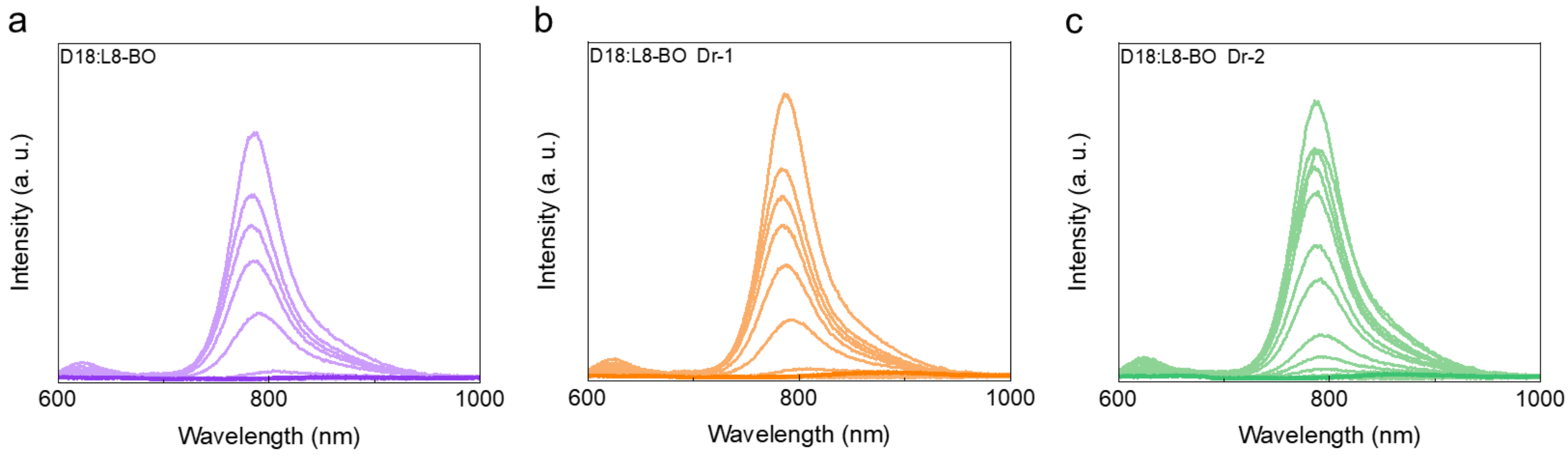

Supplementary Fig. 46. The PL spectra evolution as a function of time of **a** D18:L8-BO, **b** D18:L8-BO (Dr-1) and **c** D18:L8-BO (Dr-2), respectively.

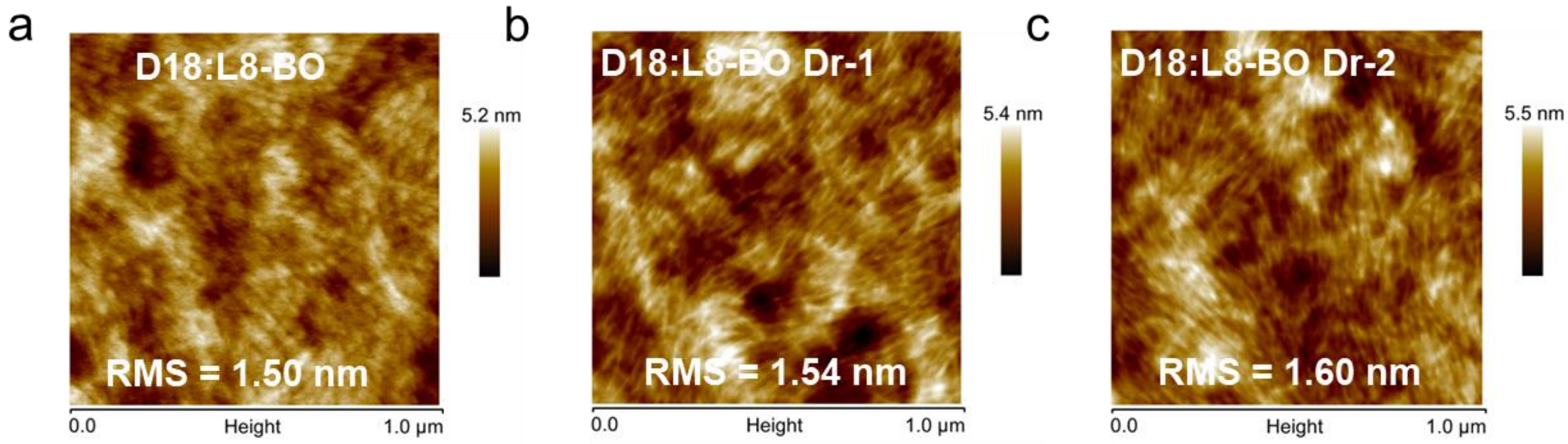

Supplementary Fig. 47. AFM height images of the blend films.

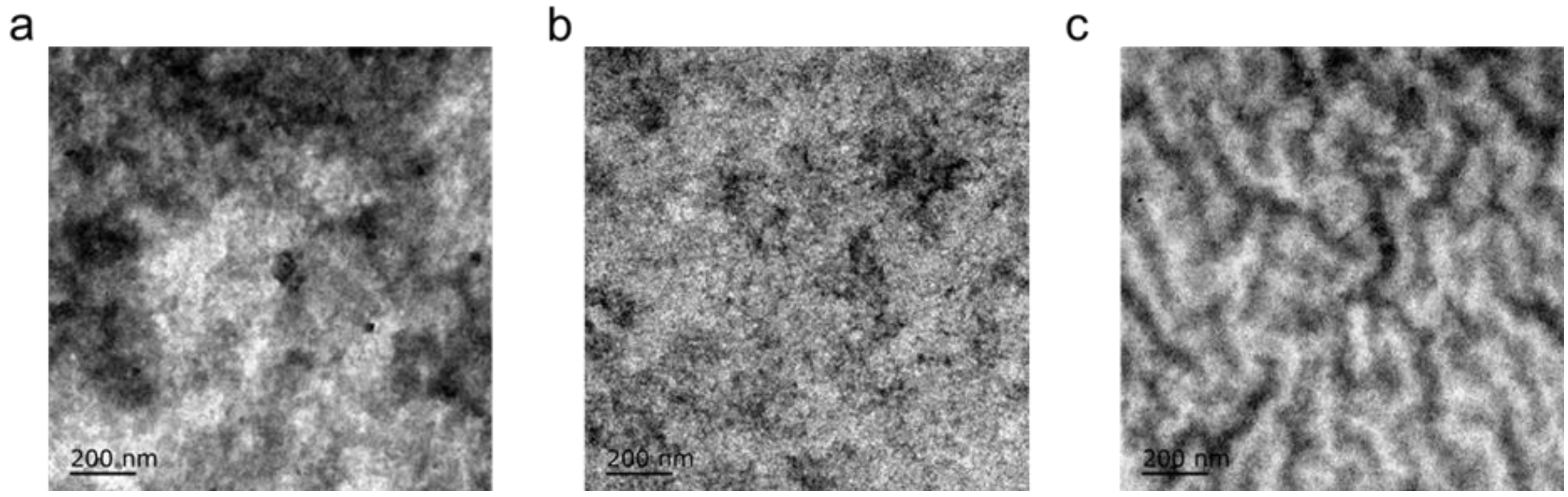

Supplementary Fig. 48. TEM images of (a) D18:L8-BO, (b) D18: L8-BO (Dr-1) and (c) D18:L8-BO (Dr-2) films.

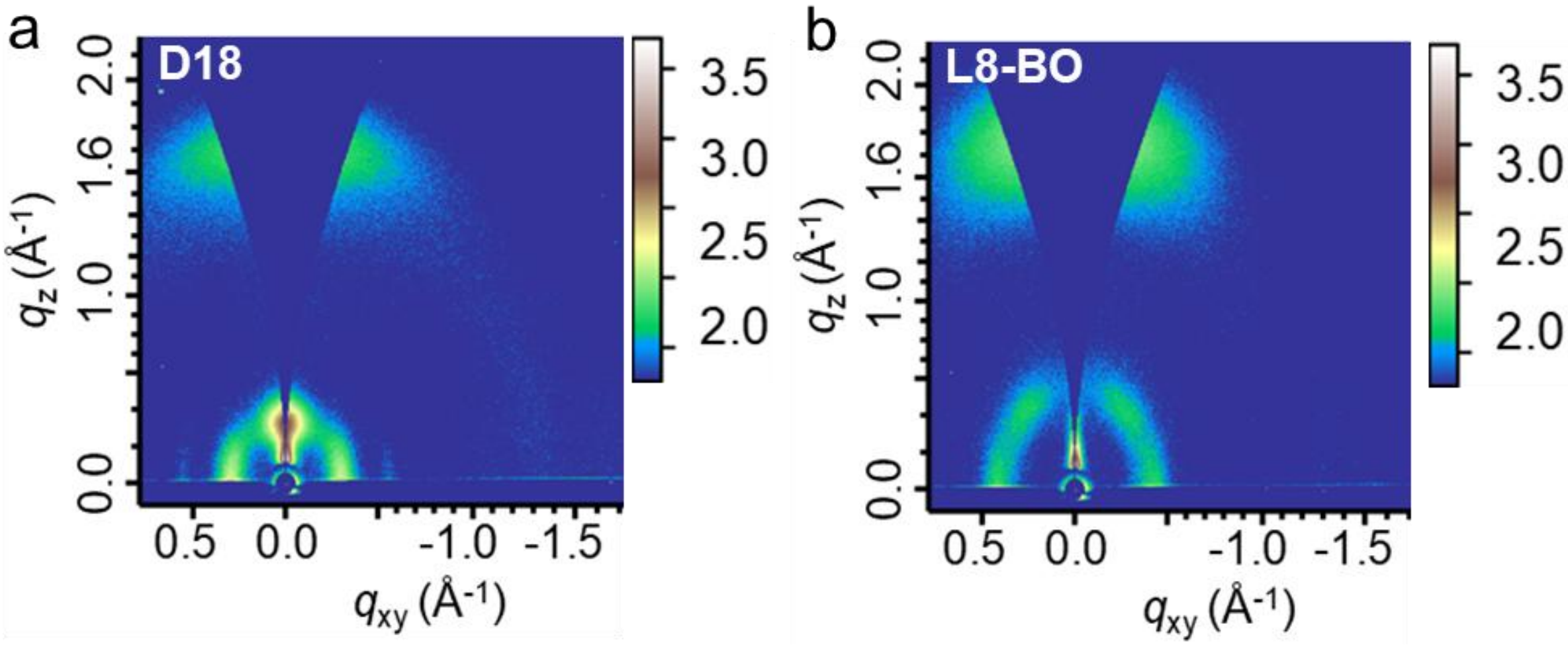

Supplementary Fig. 49. 2D GIWAXS patterns of D18 and L8-BO film.

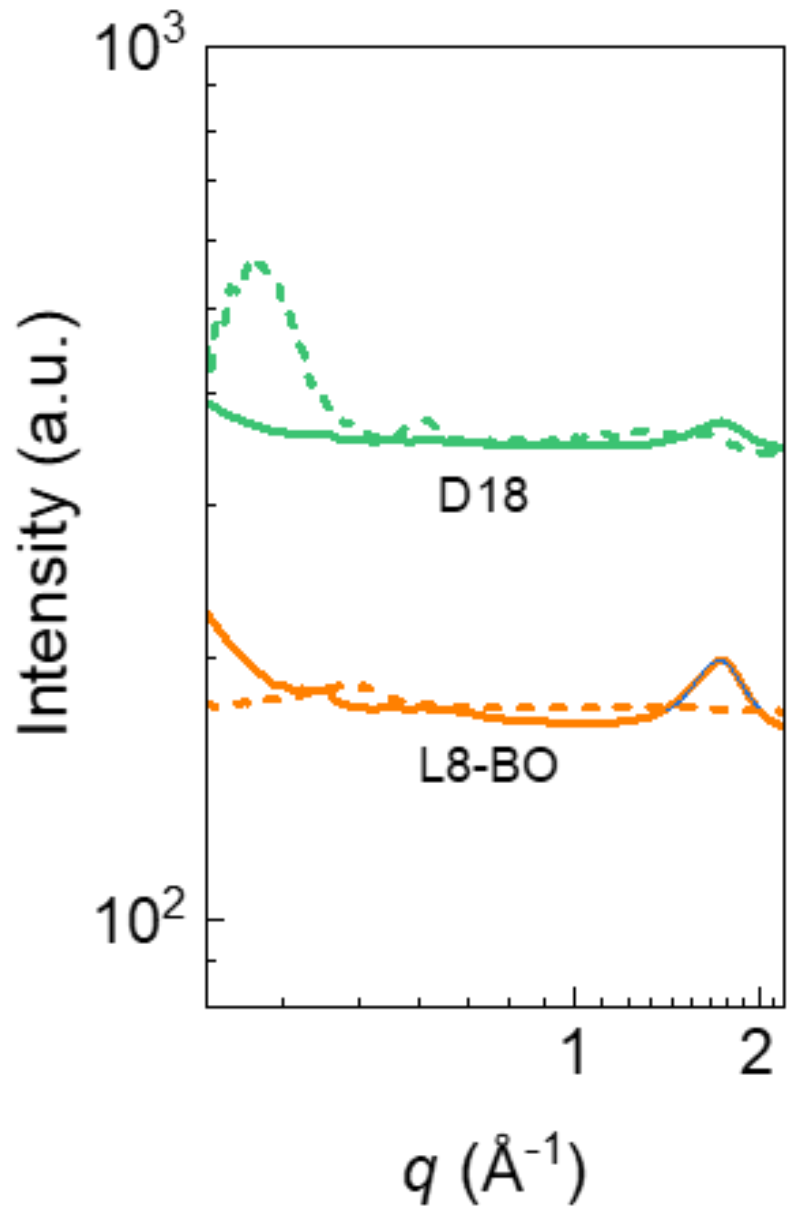

Supplementary Fig. 50. IP (dotted line) and OOP (solid line) extracted line-cut profiles of the corresponding D18, L8-BO film.

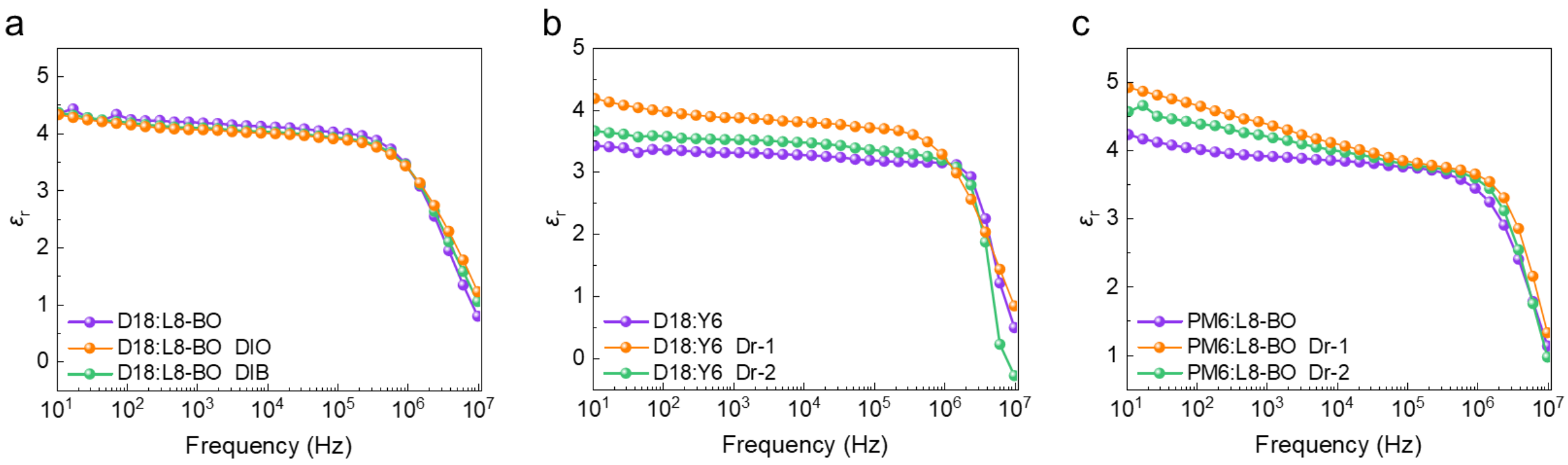

Supplementary Fig. 51. Dielectric constants of **a** D18:L8-BO, **b** D18:Y6, and **c** PM6:L8-BO systems with/without additives and dielectric regulators, respectively.

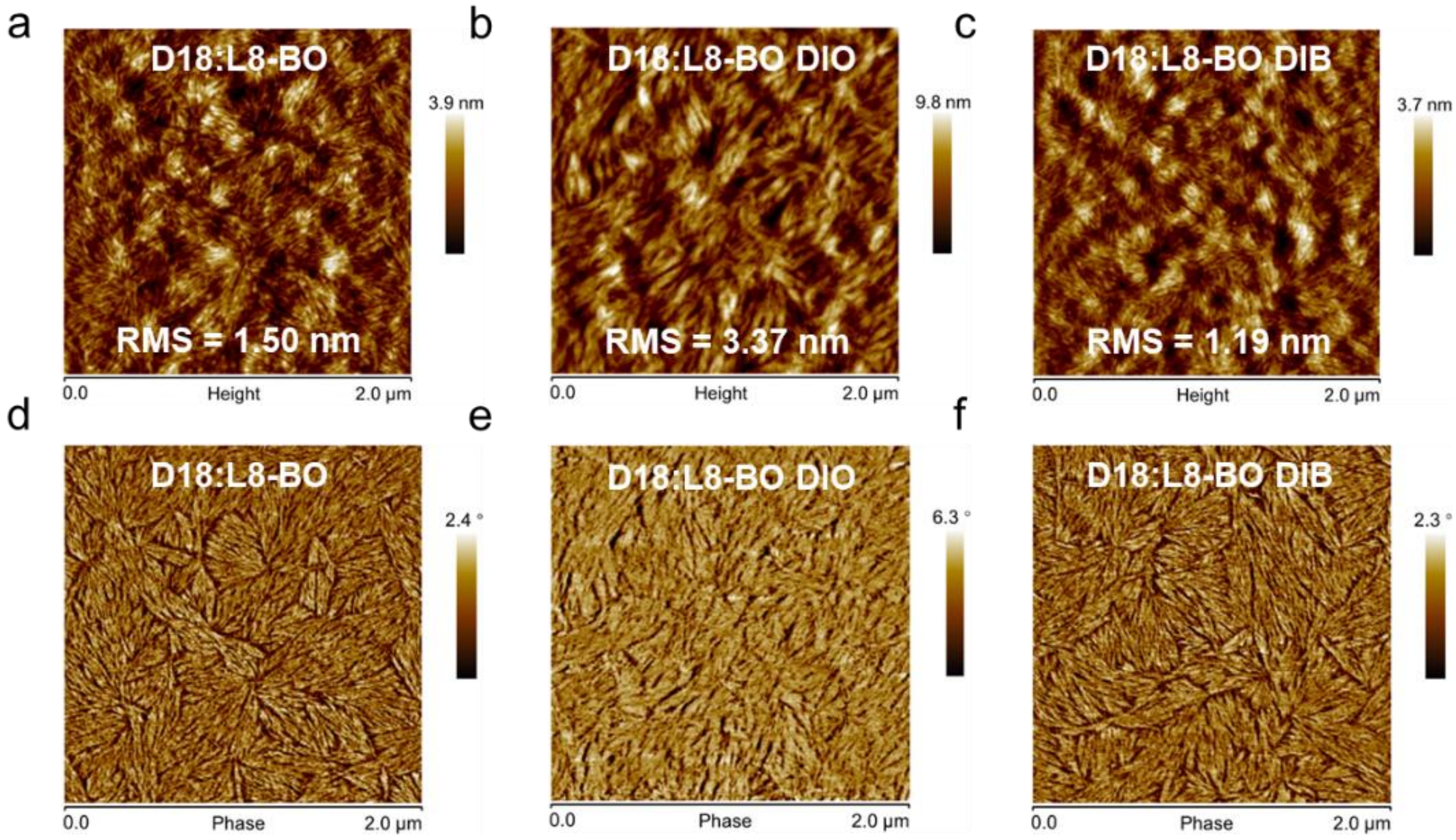

Supplementary Fig. 52. AFM image of D18:L8-BO system without additive, with DIO and DIB, respectively.

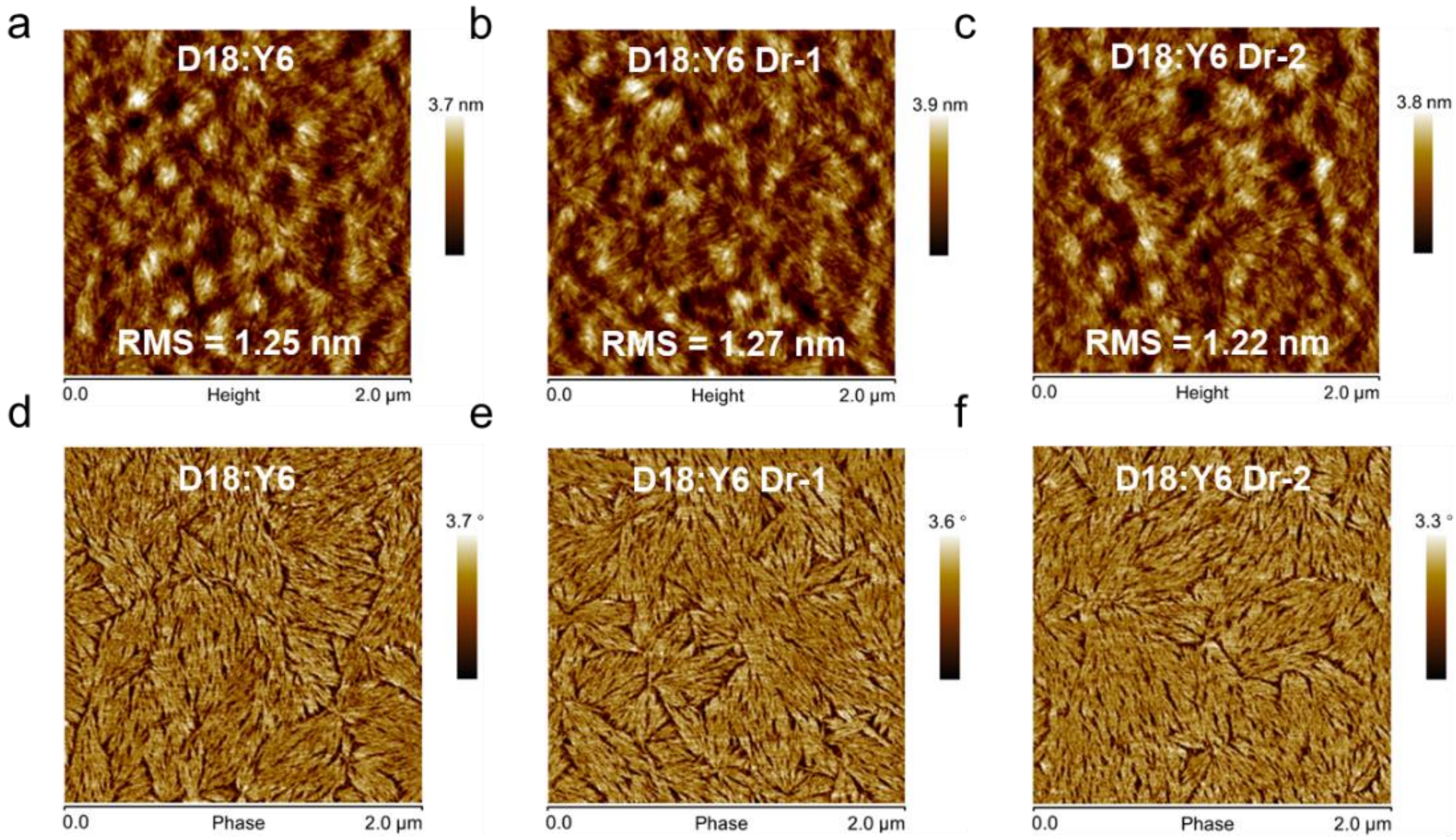

Supplementary Fig. 53. AFM image of D18:Y6 system with/without dielectric regulators.

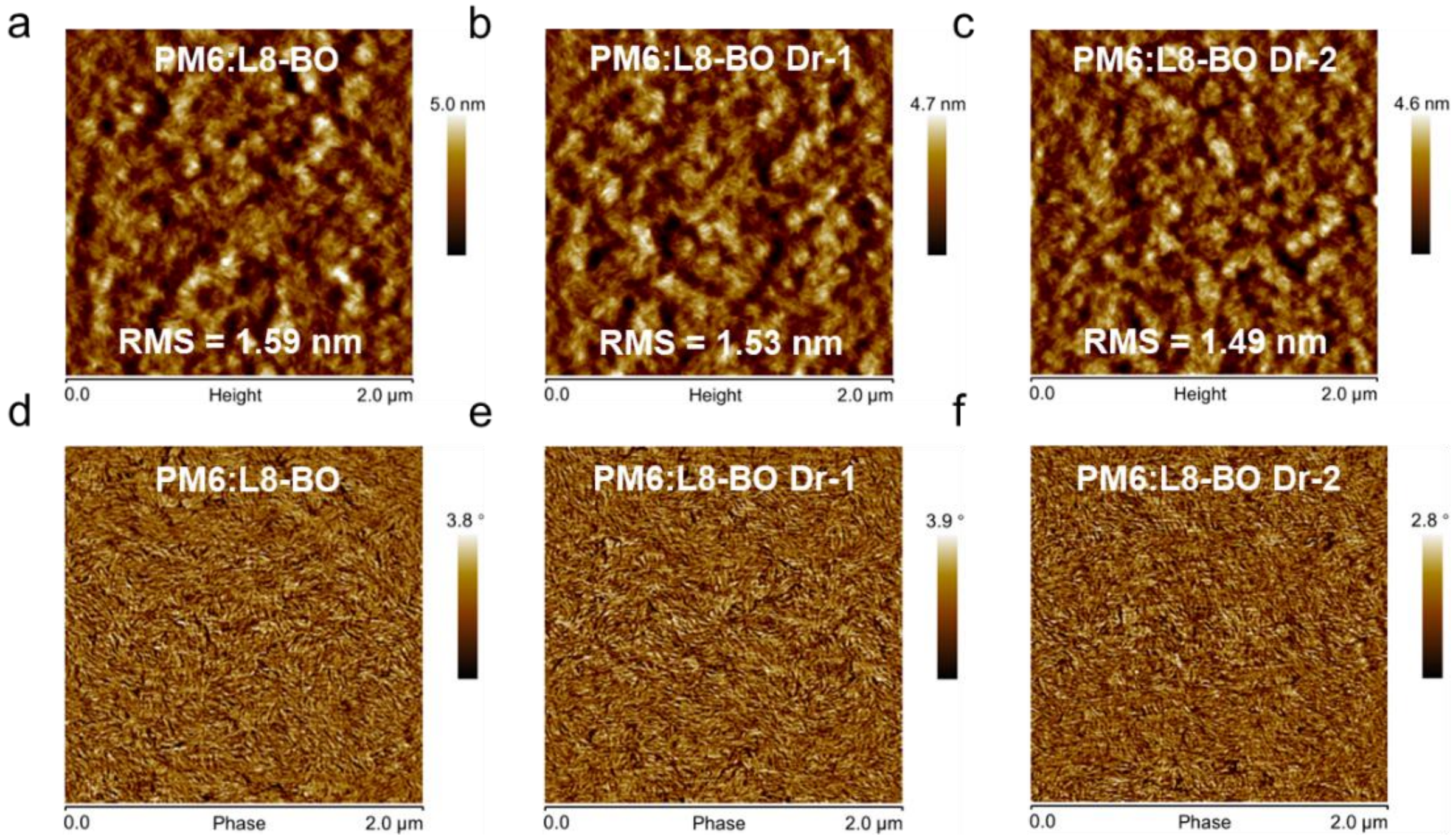

Supplementary Fig. 54 AFM image of PM6:L8-BO system with/without dielectric regulators.

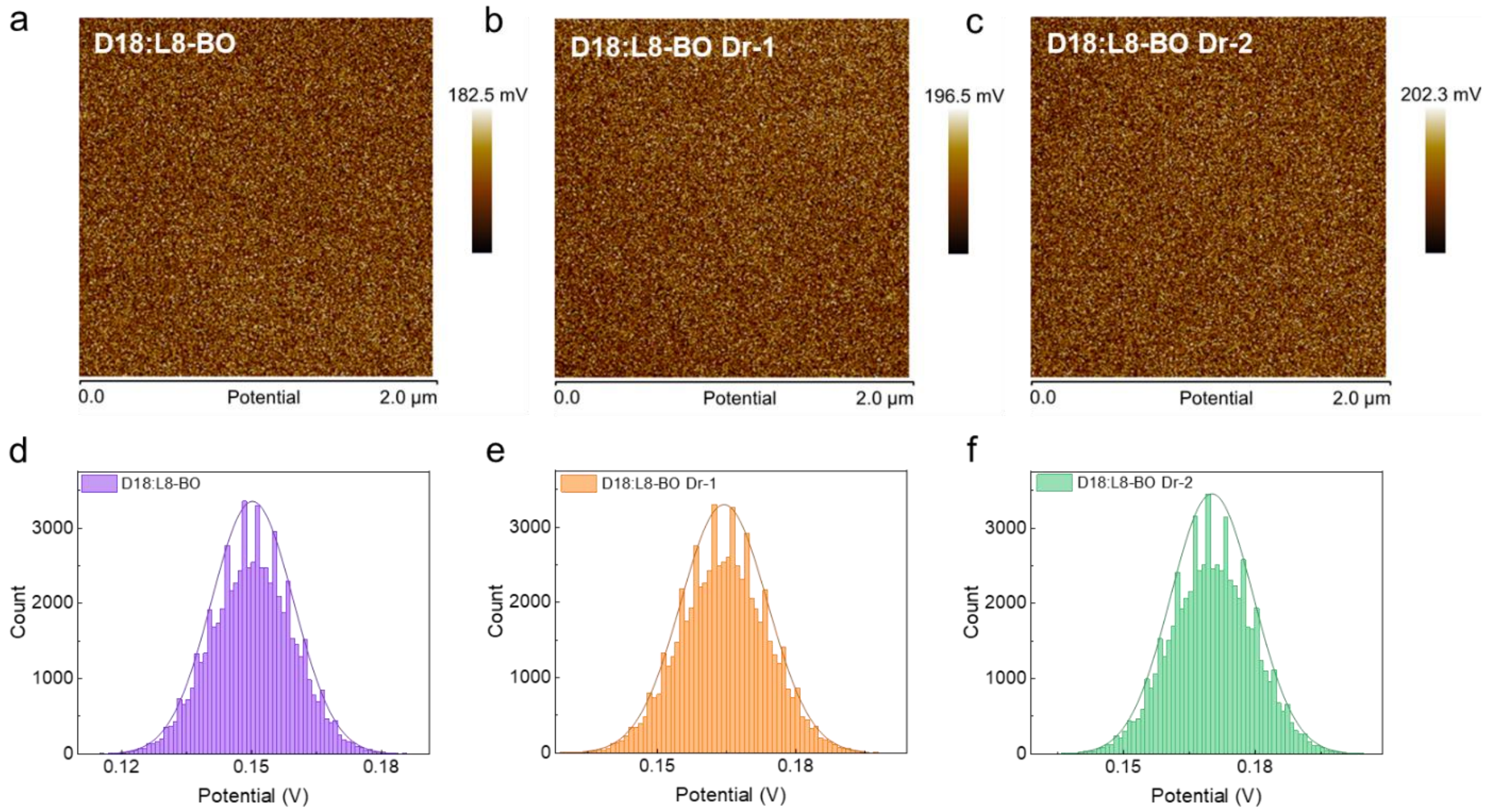

Supplementary Fig. 55. Kelvin probe atomic force microscopy (KPFM) images and surface contact potential distributions of D18:L8-BO, D18:L8-BO Dr-1 and D18:L8-BO Dr-2 films (~100 nm) under dark, respectively.

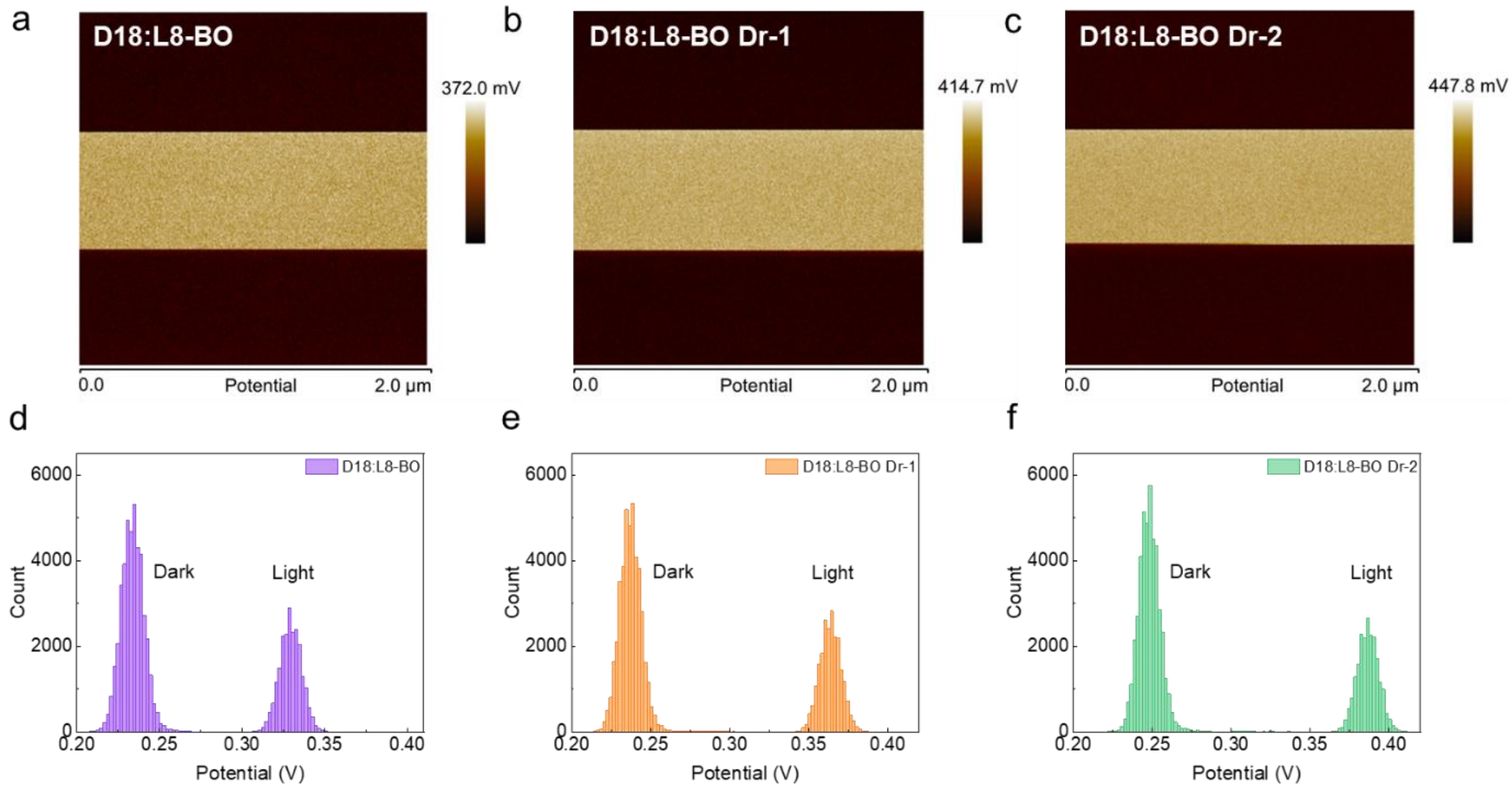

Supplementary Fig. 56. Kelvin probe atomic force microscopy (KPFM) images and surface contact potential distributions of thick-film (~300 nm) based on D18:L8-BO, D18:L8-BO Dr-1 and D18:L8-BO Dr-2 systems under dark and illumination, respectively.

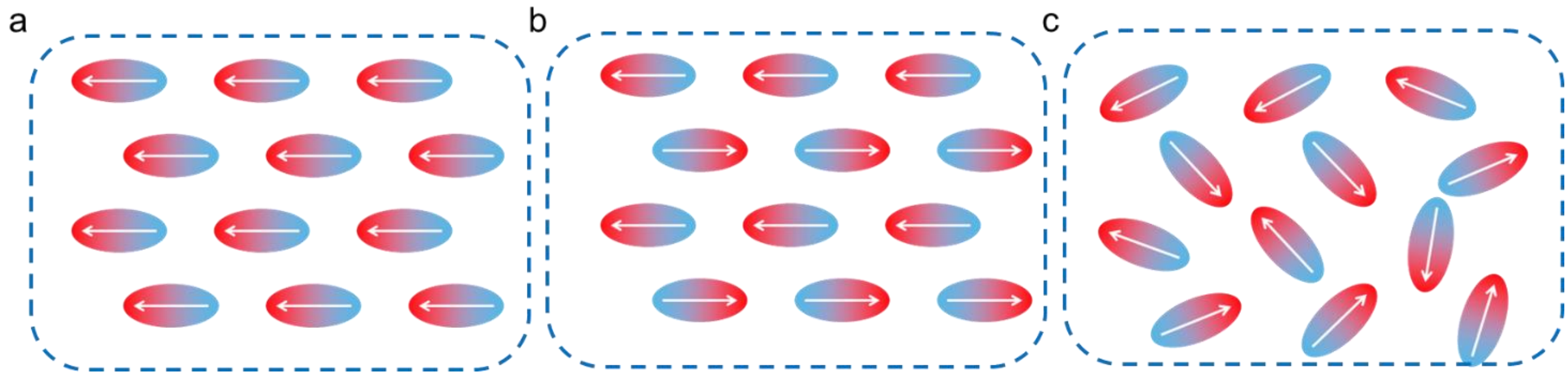

Supplementary Fig. 57. Schematic diagram of **a** parallel, **b** antiparallel and **c** disordered arrangement of dipoles.

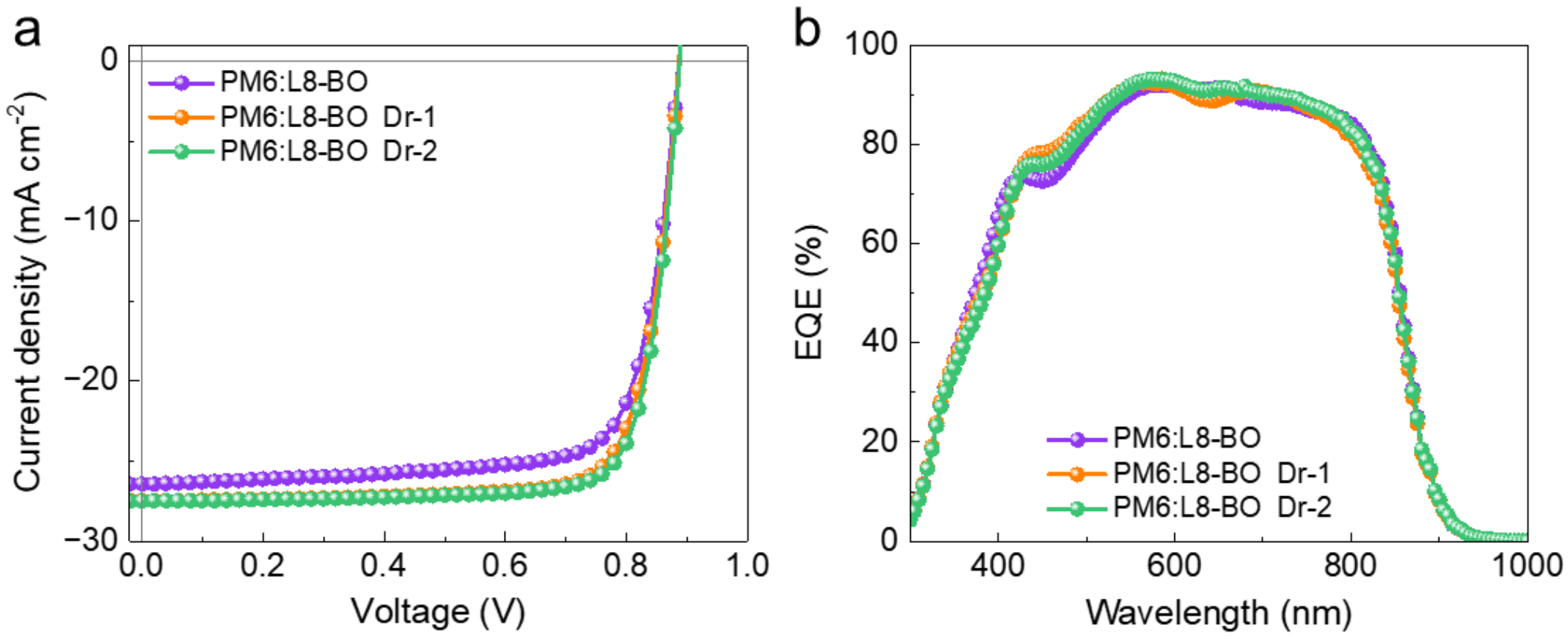

Supplementary Fig. 58. a *J*-*V* and **b** EQE curves of PM6:L8-BO systems with/without dielectric regulators.

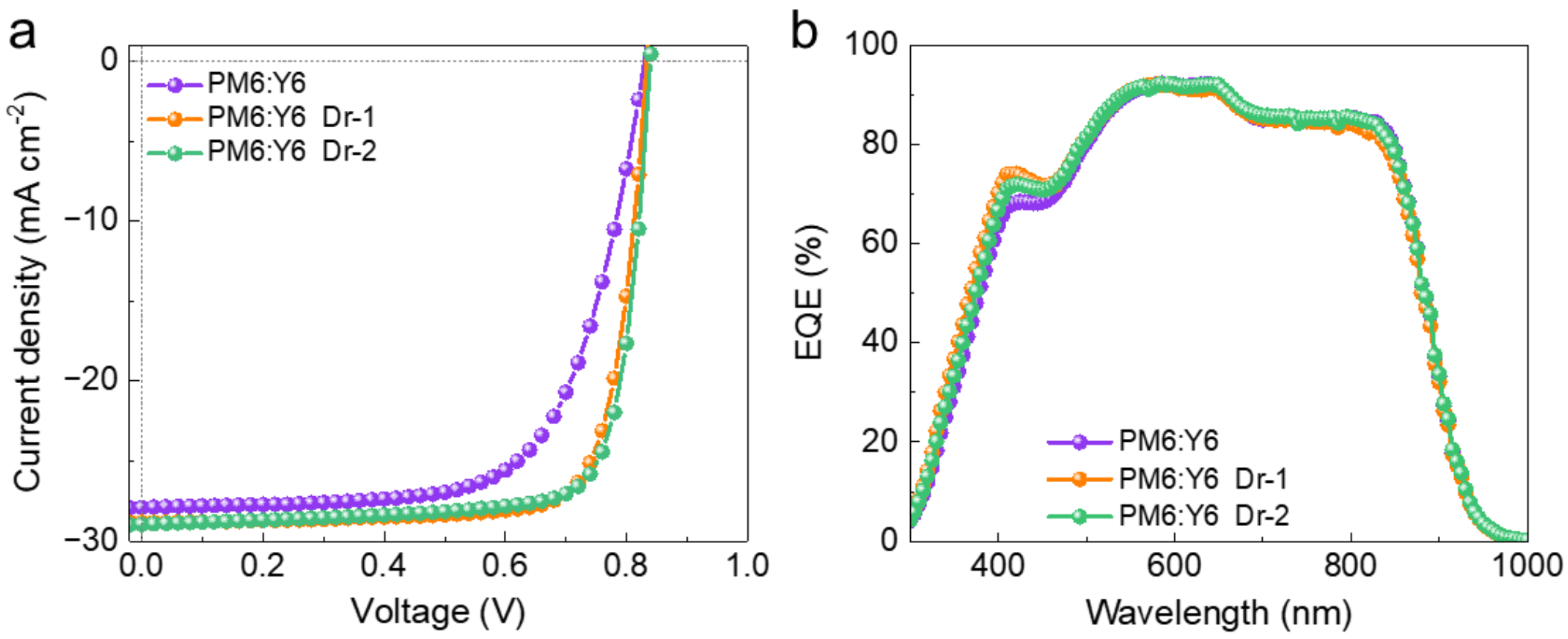

Supplementary Fig. 59. a *J*-*V* and **b** EQE curves of PM6:Y6 systems with/without dielectric regulators.

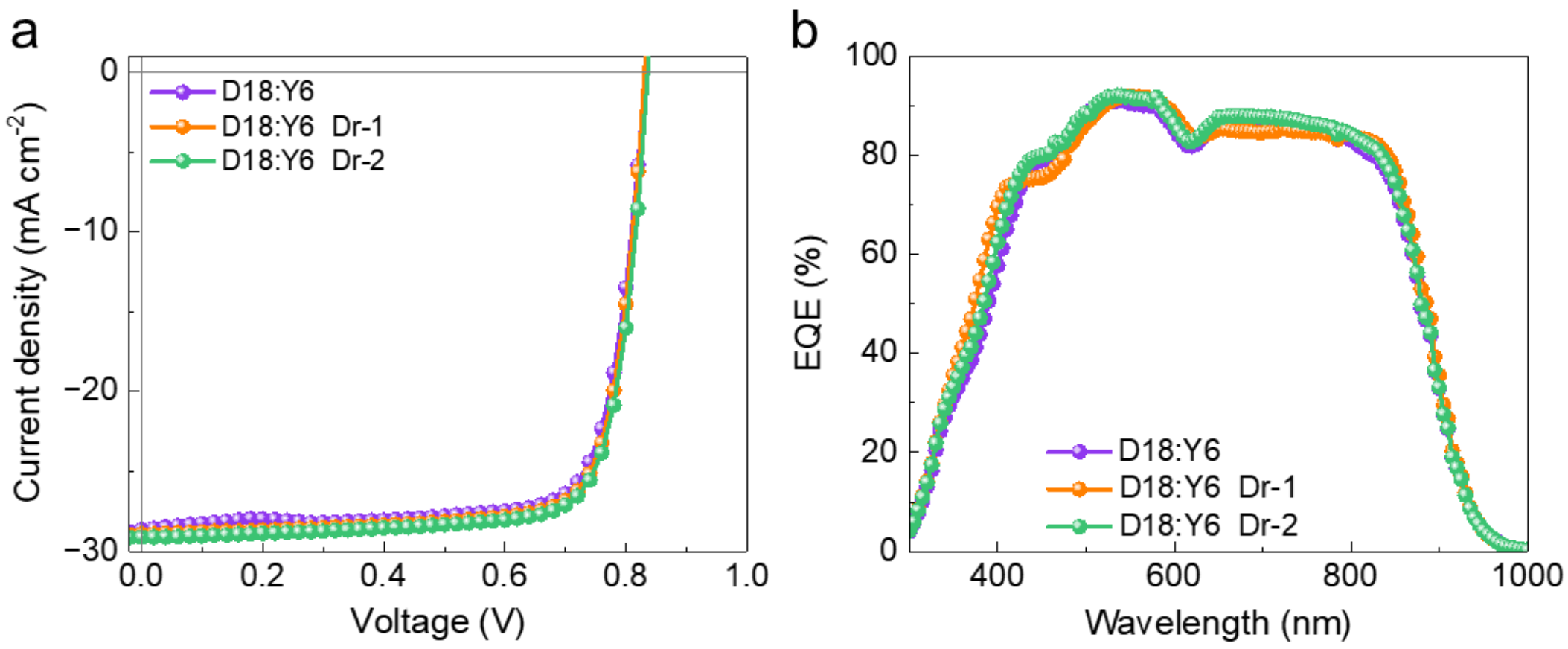

Supplementary Fig. 60. a *J*-*V* and **b** EQE curves of D18:Y6 systems with/without dielectric regulators.

2. Supplementary Tables

Supplementary Table 1. Crystallographic data for Dr-2.

Informations	Dr-2
CCDC Deposition Number	2526058
Empirical Formula	$C_{12}H_5ClF_2S_2$
Formula weight	286.73
Temperature/K	193
Crystal system	monoclinic
Space group	$P2_1/n$
a/Å	3.81710(10)
b/Å	23.0030(6)
c/Å	12.7856(3)
α/°	90
β/°	92.5130(10)
γ/°	90
Volume/$Å^3$	1121.56(5)
Z	4
ρ_{calc}/(g/cm^3)	1.698
μ/mm^{-1}	6.512
F(000)	576.0
Crystal size/mm^3	0.13 × 0.11 × 0.08
Radiation	CuKα (λ = 1.54178)
2θ range for data collection/°	7.686 to 148.646
Index ranges	$-4 \leq h \leq 3$, $-28 \leq k \leq 27$, $-15 \leq l \leq 15$
Reflections collected	11363
Independent reflections	2272 [R_{int} = 0.0316, R_{sigma} = 0.0241]
Data/restraints/parameters	2272/0/154
Goodness-of-fit on F^2	1.051
Final R indexes [I>=2σ (I)]	R_1 = 0.0242, wR_2 = 0.0656
Final R indexes [all data]	R_1 = 0.0260, wR_2 = 0.0668
Largest diff. peak/hole/e $Å^{-3}$	0.28/-0.24

Supplementary Table 2. The optimized dihedral angles (θ), orbital plane angles (α), bond lengths (d), and descriptors (S) for Dr-2.

	θ [°]	α [°]	d [Å]	$r_w{}^a$ [Å]	Δd^b [Å]	S
**Dr-2**	0.84	168.45	2.68	3.20	0.52	0.456

$^a r_w$ is the sum of van der Waals radii of two involved atoms. $^b\Delta d$ is defined as $r_w - d$.

Supplementary Table 3. The molecular positive surface area, negative surface area, positive average value and negative average value of ESP for Dr-1, Dr-2, D18, and L8-BO.

Molecule	Positive surface area (Bohr^2)	Negative surface area (Bohr^2)	Positive average value (kcal/mol)	Negative average value (kcal/mol)
Dr-1	468.92	476.88	8.67	-4.20
Dr-2	406.92	524.98	9.11	-5.58
D18	3569.45	3038.59	5.82	-5.61
L8-BO	2012.95	865.12	12.00	-11.03

Supplementary Table 4. The total Coulomb interaction energy (E_{elec}) and total Van der Waals interaction energy (E_{vdW}) between dielectric regulators and donor and acceptor molecules

Film	E_{elec} (kcal/mol)	E_{vdW} (KJ/mol)
D18:Dr -1	-68.12	-6917.35
D18:Dr -2	-84.18	-7173.46
L8-BO:Dr -1	-289.75	-8342.01
L8-BO:Dr -2	-363.96	-7840.90

Supplementary Table 5. Maximum absorption peak position of blend films.

Film	Maximum peak position (nm)		Absorption coefficient (cm^{-1})
D18:L8-BO	588	787	5.60×10^4
D18:L8-BO Dr-1	590	793	6.29×10^4
D18:L8-BO Dr-2	591	798	6.35×10^4

Supplementary Table 6. Summary of photovoltaic parameters of the D18:L8-BO with different Dr-2 contents.

**Active Layer**	**Contents (%)**	V_{oc} **(V)**	J_{sc} **(mA cm^{-2})**	**FF (%)**	**PCE[a] (%)**
D18:L8-BO Dr-2	10	0.910 (0.910±0.001)	27.22 (27.14±0.20)	80.53 (80.20±0.45)	19.95 (19.80±0.14)
	20	0.912 (0.912±0.001)	28.18 (28.02±0.19)	81.14 (80.90±0.30)	20.85 (20.68±0.11)
	30	0.907 (0.907±0.001)	28.20 (28.04±0.15)	80.79 (80.37±0.51)	20.66 (20.44±0.14)
	50	0.904 (0.904±0.001)	28.00 (27.73±0.29)	80.56 (80.36±0.23)	20.4 (20.14±0.18)

[a]The average parameters were calculated based on 10 independent cells.

Supplementary Table 7. Photovoltaic parameters of D18:L8-BO systems (300 nm) with/without dielectric regulators.

**Active Layer**	**Condition**	V_{oc} **(V)**	J_{sc} **(mA cm^{-2})**	**FF (%)**	**PCE^{a} (%)**
D18:L8-BO (300 nm)	none	0.888	27.21	76.74	18.38
	Dr-1	0.887	27.28	78.63	19.03
	Dr-2	0.887	28.22	78.16	19.56

Supplementary Table 8. Hole mobilities and electron mobilities of devices with/without different dielectric regulators.

**Active layer** D18:L8-BO	μ_h **(cm^2 V^{-1} s^{-1})**	μ_e**(cm^2 V^{-1} s^{-1})**	μ_h/μ_e
None	2.89×10^{-4}	3.49×10^{-4}	1.21
Dr-1	3.56×10^{-4}	3.04×10^{-4}	1.17
Dr-2	5.22×10^{-4}	4.82×10^{-4}	1.08

Supplementary Table 9. Relevant parameters from the Nyquist curves of the D18:L8-BO-based OSCs processed with/without dielectric regulator.

**Active layer**	R_s (Ω)	$R_{transport}$ (Ω)	$R_{recombination}$ (KΩ)
D18:L8-BO	20.68	22.78	940
D18:L8-BO DkReg-1	21.36	21.45	1.20×10^{5}
D18:L8-BO DkReg-2	21.23	17.12	1.66×10^{5}

R_S corresponds to the electrode resistance including ITO and Ag. Parallel $R_{recombination}$ correspond to resistance of the 2PACz and PNDIT-F3N interlayer. Parallel $R_{transport}$ correspond to the bulk resistance of the BHJ layer.

Supplementary Table 10. Corresponding data of DOS measurement for D18:L8-BO, D18:L8-BO (treated with Dr-1) and D18:L8-BO (treated with Dr-2) devices.

**Active layer**	***δ* (eV)**	**E_t (eV)**	**N_t ($cm^{-3}eV^{-1}$)**
D18:L8-BO	0.090	0.164	5.52×10^{16}
D18:L8-BO Dr-1	0.043	0.161	3.47×10^{16}
D18:L8-BO Dr-2	0.035	0.157	1.99×10^{16}

Supplementary Table 11. EX lifetime and CS lifetime of D18: L8-BO film with/without regulators.

Lifetime	D18: L8-BO	D18: L8-BO Dr-1	D18: L8-BO Dr-2
EX lifetime (ps)	17.21	17.19	17.08
Lifetime of CS state decay (ns)	213.52	236.66	244.46

Supplementary Table 12. Crystal Coherence lengths of the (100) peak and the d-spacing for the films.

Sample	q_{xy} (Å^{-1})	d-spacing (Å)	FWHM (Å^{-1})	CCL (Å)
D18	0.30	20.94	0.10	62.83
L8-BO	0.43	14.61	0.18	34.91

Supplementary Table 13. Crystal Coherence lengths of the (010) peak and the d-spacing for the films.

Sample	q_z (Å^{-1})	d-spacing (Å)	FWHM (Å^{-1})	CCL (Å)
D18	1.74	3.61	0.37	16.98
L8-BO	1.74	3.61	0.34	18.48

Supplementary Table 14. Crystal Coherence lengths of the (100) peak and the d-spacing for the blend films.

Active layer	q_{xy} (Å^{-1})	d-spacing (Å)	FWHM (Å^{-1})	CCL (Å)
D18:L8-BO	0.32	19.63	0.099	63.47
D18:L8-BO Dr-1	0.33	19.03	0.096	65.45
D18:L8-BO Dr-2	0.33	19.03	0.091	69.05

Supplementary Table 15. Crystal Coherence lengths of the (010) peak and the d-spacing for the blend films.

**Active layer**	**q_z (Å^{-1})**	**d-spacing (Å)**	**FWHM (Å^{-1})**	**CCL (Å)**
D18:L8-BO	1.74	3.61	0.28	22.44
D18:L8-BO Dr-1	1.74	3.61	0.26	24.17
D18:L8-BO Dr-2	1.75	3.59	0.25	25.13

Supplementary Table 16. Summary of device performance parameters related to dielectric and morphology effects of D18:L8-BO with/without additives.

System	Additives	Dielectric constant	RMS (nm)	PCE (%)
D18:L8-BO	none	4.36	1.50	19.27
	DIO	4.33	3.37	19.46
	DIB	4.37	1.19	19.96

Supplementary Table 17. Summary of device performance parameters related to dielectric and morphology effects of D18:Y6 and PM6:L8-BO with/without dielectric regulators.

System	Regulators	Dielectric constant	RMS (nm)	PCE (%)
D18:Y6	none	3.43	1.25	18.46
	Dr-1	3.66	1.27	18.86
	Dr-2	4.19	1.22	19.20
PM6:L8-BO	none	4.23	1.59	17.93
	Dr-1	4.57	1.53	19.25
	Dr-2	4.92	1.49	19.62

Supplementary Table 18. Equilibrium volume and RMSD of three systems.

System	D18:L8-BO	D18:L8-BO Dr -1	D18:L8-BO Dr -2
Equilibrium volume	369.874	369.325	368.421
COM	0.199	0.168	0.078

Supplementary Table 19. Research of different active layer systems with/without dielectric regulators.

Active Layer	Condition	V_{oc} (V)	J_{sc} (mA cm^{-2})	FF (%)	PCE (%)
PM6:L8-BO	none	0.886	26.42	76.56	17.93
	Dr-1	0.886	27.47	79.06	19.25
	Dr-2	0.887	27.50	80.46	19.62
PM6:Y6	none	0.830	27.92	67.12	15.55
	Dr-1	0.834	28.82	78.92	18.97
	Dr-2	0.840	28.97	78.75	19.16
D18:Y6	none	0.831	28.65	77.56	18.46
	Dr-1	0.830	28.93	78.53	18.86
	Dr-2	0.840	29.11	78.51	19.20
