# Supplementary material for: High performance organic solar cell enabled by manipulating the exciton dissociation and charge transfer via dielectric engineering
Source: Nat Commun. 2026 Jun 16;17:7649. doi: 10.1038/s41467-026-74112-x (PMC13434753; doi:10.1038/s41467-026-74112-x)
Supplement: Supplementary file 2 [file 41467_2026_74112_MOESM2_ESM.pdf]

nature portfolio

Corresponding author(s): Yunhao Cai, Hui Huang.

Solar Cells Reporting Summary

Nature Portfolio wishes to improve the reproducibility of the work that we publish. This form is intended for publication with all accepted papers reporting the characterization of photovoltaic devices and provides structure for consistency and transparency in reporting. Some list items might not apply to an individual manuscript, but all fields must be completed for clarity.

For further information on Nature Research policies, including our data availability policy, see Authors & Referees.

• Experimental design

Please check the following details are reported in the manuscript, and provide a brief description or explanation where applicable.

1. Dimensions

Area of the tested solar cells

- [x] Yes — The active area of the cells was 5 mm^2 and was measured by the aperture of 2.57 mm^2.
- [ ] No — *Explain why this information is not reported/not relevant.*

Method used to determine the device area

- [x] Yes — The area of the device was defined by a shadow mask, which is certified by South China National Center of Metrology.
- [ ] No — *Explain why this information is not reported/not relevant.*

2. Current-voltage characterization

Current density-voltage (J-V) plots in both forward and backward direction

- [ ] Yes — *State where this information can be found in the text.*
- [x] No — Photo-current hysteresis is not observed in organic solar cells, thus backward direction scan was not applied in this system.

Voltage scan conditions

- [x] Yes — The current-voltage curves characteristics of photovoltaic devices were obtained along the forward scan direction from −0.5 to 1 V, with a scan step of 20 mV and a dwell time of 1 ms.
- [ ] No — *Explain why this information is not reported/not relevant.*

Test environment

- [x] Yes — The current-voltage curves were measured under a temperature of 25 °C in a glove box filled with nitrogen. (Section "Device Characterization" in Methods)
- [ ] No — *Explain why this information is not reported/not relevant.*

Protocol for preconditioning of the device before its characterization

- [ ] Yes — *Provide a description of the protocol.*
- [x] No — No preconditioning protocol.

Stability of the J-V characteristic

- [x] Yes — The stability of the J-V characteristic was measured under standard test condition for 5 min.
- [ ] No — *Explain why this information is not reported/not relevant.*

3. Hysteresis or any other unusual behaviour

Description of the unusual behaviour observed during the characterization

- [ ] Yes — *Provide a description of hysteresis or any other unusual behaviour observed during the characterization.*
- [x] No — No hysteresis or other unusual behaviour was observed during the characterization of the solar cells. In general, organic solar cells do not have hysteresis problems.

Related experimental data

- [ ] Yes — *Provide a description of the related experimental data.*
- [x] No — No hysteresis or other unusual behaviour was observed during the characterization of the solar cells.

4. Efficiency

External quantum efficiency (EQE) or incident photons to current efficiency (IPCE)

- [x] Yes — EQE curve is shown in Fig. 2b.
- [ ] No — *Explain why this information is not reported/not relevant.*

A comparison between the integrated response under the standard reference spectrum and the response measure under the simulator

☒ Yes
☐ No

The integrated Jsc values from EQE spectra are consistent with that from J-V measurements within 5% mismatch. (Figs. 2a and 2b)

Explain why this information is not reported/not relevant.

For tandem solar cells, the bias illumination and bias voltage used for each subcell

☐ Yes
☒ No

Provide a description of the measurement conditions.

We only fabricated single-junction solar cells.

5. Calibration

Light source and reference cell or sensor used for the characterization

☒ Yes
☐ No

A standard silicon cell was used to calibration the light source intensity. (Section "Device Characterization" in Methods)

Explain why this information is not reported/not relevant.

Confirmation that the reference cell was calibrated and certified

☒ Yes
☐ No

The reference cell was calibrated by South China National Center of Metrology.

Explain why this information is not reported/not relevant.

Calculation of spectral mismatch between the reference cell and the devices under test

☒ Yes
☐ No

The light spectrum used for measurements matches well with the reference silicon cell, and we did not calculate the spectral mismatch between the reference cell and the test devices.

Explain why this information is not reported/not relevant.

6. Mask/aperture

Size of the mask/aperture used during testing

☒ Yes
☐ No

All samples were measured with a 2.57 mm^2 mask for cells.

Explain why this information is not reported/not relevant.

Variation of the measured short-circuit current density with the mask/aperture area

☐ Yes
☒ No

Report the difference in the short-circuit current density values measured with the mask and aperture area.

We measured all the devices with the same aperture.

7. Performance certification

Identity of the independent certification laboratory that confirmed the photovoltaic performance

☒ Yes
☐ No

A recorded efficiency of 20.38% was certified by South China National Center of Metrology.

Explain why this information is not reported/not relevant.

A copy of any certificate(s)

☒ Yes
☐ No

See"Supplementary Fig. 21"

Explain why this information is not reported/not relevant.

8. Statistics

Number of solar cells tested

☒ Yes
☐ No

The device performance statistics was based on around 20 devices.

Explain why this information is not reported/not relevant.

Statistical analysis of the device performance

☒ Yes
☐ No

Results were included in Table 1.

Explain why this information is not reported/not relevant.

9. Long-term stability analysis

Type of analysis, bias conditions and environmental conditions

☐ Yes
☒ No

Provide a description of the type of analysis, bias conditions and environmental conditions (e.g. illumination type, temperature, atmosphere humidity, encapsulation method, preconditioning temperature, bias) for each long-term stability analysis carried out; see ref. 7 and 8 for details.

Photostability were measured (See "Supplementary 24")
