# Supplementary material for: High performance organic solar cell enabled by manipulating the exciton dissociation and charge transfer via dielectric engineering
Source: Nat Commun. 2026 Jun 16;17:7649. doi: 10.1038/s41467-026-74112-x (PMC13434753; doi:10.1038/s41467-026-74112-x)
Supplement: Supplementary file 3 [file 41467_2026_74112_MOESM3_ESM.pdf]

nature portfolio

Peer Review File

High performance organic solar cell enabled by manipulating the exciton dissociation and charge transfer via dielectric engineering

Corresponding Author: Professor Hui Huang

This file contains all reviewer reports in order by version, followed by all author rebuttals in order by version.

Version 0:

Reviewer comments:

Reviewer #1

(Remarks to the Author)
In this manuscript, Wei and co-authors present a study into dielectric regulation as a strategy to enhance the performance of OSCs. The study addresses the low dielectric constants of organic semiconductors, which restrict efficient exciton dissociation and charge transfer of OSCs. The authors designed two dielectric regulators through F-isomerization, yielding molecules with distinct dipole moments and backbone conformations to systematically probe structure–dielectric relationships. The incorporation of Dr-2 into the D18:L8-BO blend leads to an impressive PCEs of 20.85%. Particularly noteworthy is the compatibility of the dielectric modulation strategy with thick-film device fabrication, affording a record-high efficiency. These findings convincingly demonstrate that tailoring the dielectric microenvironment can effectively mitigate the severe recombination issues especially encountered in thick films. Overall, the manuscript offers very meaningful insights into dielectric engineering and presents a rational molecular design strategy for advancing OSCs. I strongly recommend its publication in Nature Communications after the following revisions.
1. Please provide TEM images of the films processed under the different conditions to better elucidate the impact of dielectric regulators on film nanostructure.
2. It is recommended that the fourier-transform infrared spectra of Dr-1 and Dr-2 be included to support the structural analysis of the dielectric regulators.
3. The authors state that S···F NoCLs induce higher planarity, better conjugation, and larger dipole moment in Dr-2. Could the authors explain how the NoCLs influence charge delocalization and dielectric response within the active layer?
4. The authors are encouraged to provide dark current measurements to clarify how the introduction of dielectric regulators influences the dark current characteristics of the devices.
5. Given the strong dependence of OSC performance on polymer quality, please report the molecular weight and polydispersity index of the D18 batch used in this study.
6. Please provide the UV absorption spectra of the two dielectric regulators.
7. There are a few format errors in the article and references, the authors should carefully check the manuscript.

Reviewer #2

(Remarks to the Author)
This manuscript reports two fluorine-isomerized dielectric modifiers (Dr-1 and Dr-2) designed to optimize exciton dissociation and charge transport by enhancing the dielectric constant of the active layer, ultimately achieving a power conversion efficiency (PCE) of 20.85% in the D18:L8-BO system. Although the manuscript covers synthesis, characterization, device physics, and simulations, it exhibits some shortcomings in the rigor of its core scientific logic, the causality of its mechanism arguments, and the depth of its research paradigm. Consequently, it should be revised carefully.
1. The manuscript attributes the performance enhancement to “increased dielectric constant,” but this represents merely an observational correlation rather than a causal demonstration. All observed performance improvements, such as accelerated exciton dissociation, enhanced charge mobility, and suppressed recombination, could entirely stem from changes in the active layer morphology, crystallinity, and molecular packing order induced by the introduction of Dr-2. These are well-known effects commonly associated with solid-state additives. The author failed to design key experiments to isolate the “dielectric effect” from the “morphology effect.” The current data only demonstrates that Dr-2 simultaneously optimizes both morphology and dielectric environment, but cannot determine which is the dominant factor.

2. The manuscript claims to provide a “general strategy” for designing high-performance dielectric regulators. However, only Dr-1 and Dr-2, a pair of closely related structures, are presented. The structural optimization is very limited and cannot substantiate such a strong conclusion as a “universal molecular design principle.”
3. The authors repeatedly emphasize that Dr-2 improves device performance by increasing ε_r, thereby reducing Eb and lowering recombination rates. Yet Dr-2 also significantly alters the blend-film morphology, molecular packing, and charge-carrier mobility and balance, factors that themselves have a substantial impact on charge generation, transport, and recombination. The present experimental design, therefore, makes it difficult to distinguish the “pure dielectric effect” from the contributions of improved morphology and packing. Several discussions in the paper diverge after this point.
4. The manuscript reports excessively high JSC values, with EQE clearly exceeding 92%, which is inconsistent with objective principles.
5. The “macroscopic” dielectric constant (~4.3 to 4.8) measured via the thin-film capacitance method exhibits minimal variation and fails to reflect the true changes in the local dielectric environment at the donor/acceptor interface—the very factor critical for exciton dissociation and charge separation. The absence of direct detection of local polarization capabilities at the interface (e.g., via photoabsorption spectroscopy) leaves the core argument built upon fragile foundations.
6. The manuscript employs ESP analysis, FTIR red shift, and MD simulations to demonstrate the “strong interaction” between Dr-2 and L8-BO. However, the FTIR red shift of only 1–3 cm^{-1} may fall within the range of instrumental error and does not constitute quantitative evidence of interaction strength. The accuracy of force field parameters used in MD simulations for describing such weak noncovalent interactions is questionable. More conclusive evidence, such as solid-state NMR, is lacking.
7. The failure to distinguish characteristic times for processes such as exciton dissociation, charge separation, and charge recombination through femtosecond transient absorption global fitting prevents the kinetic data from providing effective support for dielectric regulation mechanisms.
8. The manuscript fails to demonstrate changes in charge transport length within thick films using techniques such as scanning Kelvin probe microscopy (SKPM), thereby lacking proof of substantial improvement in thick-film charge transport through dielectric regulation. Furthermore, the lack of comparison between the external quantum efficiency (EQE) spectra of thick-film and thin-film devices precludes ruling out the contribution of enhanced light absorption in thick films to Jsc. Consequently, the conclusion that “dielectric modulation suppresses recombination in thick films” lacks direct evidence.
9. Stability is a core metric for the practical application of OSCs, yet the manuscript did not conduct any stability assessment experiments (such as atmospheric stability, thermal stability, or light stability).

Reviewer #3

(Remarks to the Author)
This work enhances exciton dissociation and charge transfer by modulating dielectric constants through additive engineering, thereby achieving high-efficiency organic photovoltaic cells.

--The primary issue is that similar studies have been extensively reported in the literature. For instance, "Elevating dielectric constant via additive engineering: Achieving 19.23% certified efficiency in thick-film binary organic solar cells, Joule, 2025, 9, 102135," and several reviews have summarized similar additive effects, such as "Dielectric Constant: A Key Factor Limiting the Future Development of Organic Solar Cells, Solar RRL, 2025, 9, e202500608."
Additionally, there is a large amount of literature and reviews on dielectric engineering, For instance, "Dielectric constant engineering of nonfullerene acceptors enables a record fill factor of 83.58% and a high efficiency of 20.80% in organic solar cells, Energy Environ. Sci., 2025, 18, 4982–4995," and "On the Understandings of Dielectric Constant and Its Impacts on the Photovoltaic Efficiency in Organic Solar Cells, Chinese Journal of Chemistry, 2021, 39, 381–390."
The previously published work demonstrates the lack of innovation in this study.

-- The authors achieved a power conversion efficiency as high as 20.85% but did not undergo third-party certification. This is a significant shortcoming for work with such high efficiency.

-- The design rationale for Dr-1 and Dr-2 is not clearly explained. Dielectric constants can be modulated through various methods, such as incorporating non-conductive polymers, what are the basis of these newly synthesized molecules.

--It is vaguely mentioned that Dr-2 exhibits intramolecular F…S interactions, yet no crystal structure is provided. Moreover, would such weak intramolecular interactions remain intact under heating conditions?

-- As the most critical evidence for intermolecular interactions, FTIR shows shifts of only 2–3 wavenumbers, which falls within the margin of error. However, the authors exaggerated the significance of these changes.

-- Morphological analyses, including GIWAXS, do not reveal significant changes, yet the authors claim that "the dielectric regulator effectively controls molecular packing."

-- Figure 5f illustrates the process of modulating dielectric constants via additives, but it lacks robust evidence. Furthermore, during the annealing process to remove additives, could the orientation of molecules in the active layer not change?

Based on the aforementioned shortcomings, the reviewer considers this work unsuitable for publication in Nature Communications.

Version 1:

Reviewer comments:

Reviewer #1

(Remarks to the Author)
I have reviewed the revised version of the manuscript submitted by the authors. The revisions have fully addressed my previous comments, and I am very satisfied with the current version.

Therefore, I recommend that the manuscript be accepted for publication as it is, without further modifications.

Reviewer #2

(Remarks to the Author)
The authors have addressed the questions well. This work can be aaccepted.

Open Access This Peer Review File is licensed under a Creative Commons Attribution 4.0 International License, which permits use, sharing, adaptation, distribution and reproduction in any medium or format, as long as you give appropriate credit to the original author(s) and the source, provide a link to the Creative Commons license, and indicate if changes were made.
In cases where reviewers are anonymous, credit should be given to 'Anonymous Referee' and the source.
The images or other third party material in this Peer Review File are included in the article’s Creative Commons license, unless indicated otherwise in a credit line to the material. If material is not included in the article’s Creative Commons license and your intended use is not permitted by statutory regulation or exceeds the permitted use, you will need to obtain permission directly from the copyright holder.
To view a copy of this license, visit https://creativecommons.org/licenses/by/4.0/

Reviewer #1:

In this manuscript, Wei and co-authors present a study into dielectric regulation as a strategy to enhance the performance of OSCs. The study addresses the low dielectric constants of organic semiconductors, which restrict efficient exciton dissociation and charge transfer of OSCs. The authors designed two dielectric regulators through F-isomerization, yielding molecules with distinct dipole moments and backbone conformations to systematically probe structure–dielectric relationships. The incorporation of Dr-2 into the D18:L8-BO blend leads to an impressive PCEs of 20.85%. Particularly noteworthy is the compatibility of the dielectric modulation strategy with thick-film device fabrication, affording a record-high efficiency. These findings convincingly demonstrate that tailoring the dielectric microenvironment can effectively mitigate the severe recombination issues especially encountered in thick films. Overall, the manuscript offers very meaningful insights into dielectric engineering and presents a rational molecular design strategy for advancing OSCs. I strongly recommend its publication in Nature Communications after the following revisions.

Reply: We greatly thank the reviewer for her/his effort in evaluating and providing positive comments upon our work. Following the reviewers' suggestions, we have performed experiments and added corresponding descriptions in the revised manuscript. The details are as follows:

Comment 1: Please provide TEM images of the films processed under the different conditions to better elucidate the impact of dielectric regulators on film nanostructure.

Reply 1: Thanks for the reviewer's comment. The TEM images of different films and corresponding description have been added in the revised manuscript (Supplementary Fig. 44, also referred to as Figure R1) as follows: TEM characterization of the blend films (Supplementary Fig. 44) reveals that the D18:L8-BO (Dr-2) film exhibits a fibrillar morphology, while the pristine D18:L8-BO film with excessive aggregation and the D18:L8-BO (Dr-1) film with some particulate aggregates. This fibrillar structure is favorable for exciton dissociation and facilitates charge transport within the film.

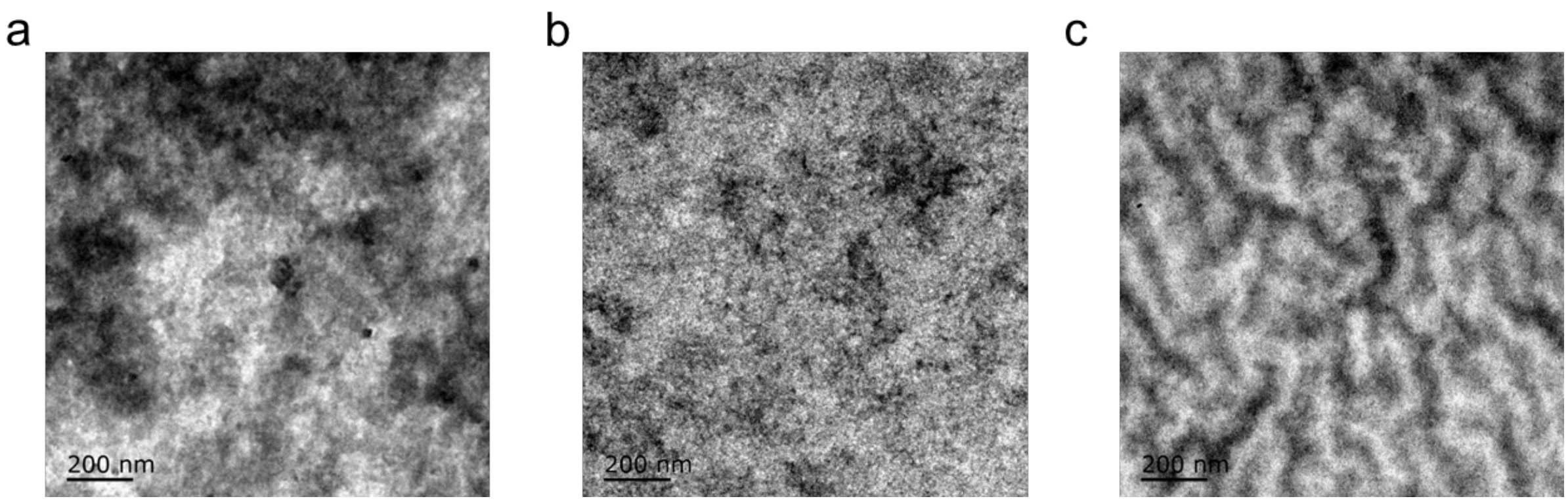

Figure R1. TEM images of (a) D18:L8-BO, (b) D18: L8-BO (Dr-1) and (c) D18:L8-BO (Dr-2) films.

Comment 2: It is recommended that the fourier-transform infrared spectra of Dr-1 and Dr-2 be included to support the structural analysis of the dielectric regulators.

Reply 2: The Fourier transform infrared (FTIR) spectra of Dr-1 and Dr-2 materials are added to support the structural analysis of the dielectric regulators (Supplementary Fig. 17, also referred to as Figure R2). The signals observed in the region of 2800-3000 cm^{-1} for the regulator can be assigned to the stretching vibration of C–H bonds.

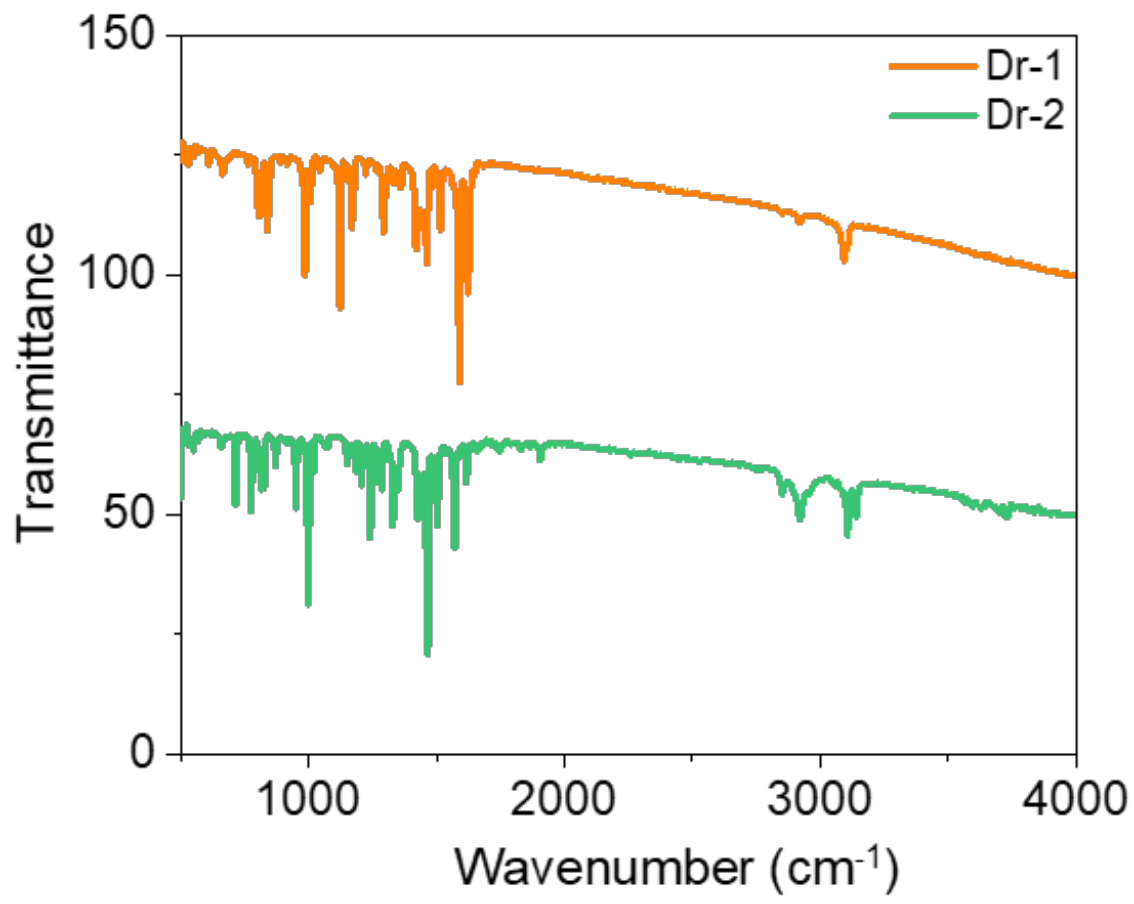

Figure R2. FTIR spectra of Dr-1 and Dr-2 materials.

Comment 3: The authors state that S···F NoCLs induce higher planarity, better conjugation, and larger dipole moment in Dr-2. Could the authors explain how the NoCLs influence charge delocalization and dielectric response within the active layer?

Reply 3: Thank you for your insightful question regarding the influence of S···F noncovalent interactions (NoCLs) on charge delocalization and dielectric response in the active layer. The S···F NoCLs in Dr-2 enforce a rigid and planar backbone through chalcogen-halogen interactions, which restrict torsional rotations. This planarization extends the effective conjugation length, expanding the π-electron delocalization domain in the molecule. The intermolecular interaction between regulators and active layer materials induces tight packing. Together, these effects enhance charge delocalization, leading to more efficient charge transport within the active layer.

Regarding the dielectric response, the larger dipole moment resulting from S···F NoCLs contributes significantly to an increased dielectric constant (ε) of the materials. The permanent dipoles introduced by these polar interactions align under an external electric field, augmenting the internal polarization of the material. A higher dielectric constant effectively screens Coulombic interactions between charge carriers and counterions, reducing charge recombination and enabling more efficient charge separation at the donor-acceptor interface. Additionally, the improved molecular packing from NoCLs enhances the material's structural order, which further optimizes the dielectric response by creating a more uniform environment for dipole alignment and charge carrier movement.

Comment 4: The authors are encouraged to provide dark current measurements to clarify how the introduction of dielectric regulators influences the dark current characteristics of the devices.

Reply 4: Thanks for the comments. The dark currents of devices were tested, and corresponding analysis has been added in revised manuscript (Supplementary Fig. 23, also referred to as Figure R3) as follows: The Dr-2-treated D18:L8-BO device exhibited a reduced reverse saturation current density, which helps minimize parasitic current under dark conditions and thus enhances the device's photoelectric response efficiency.

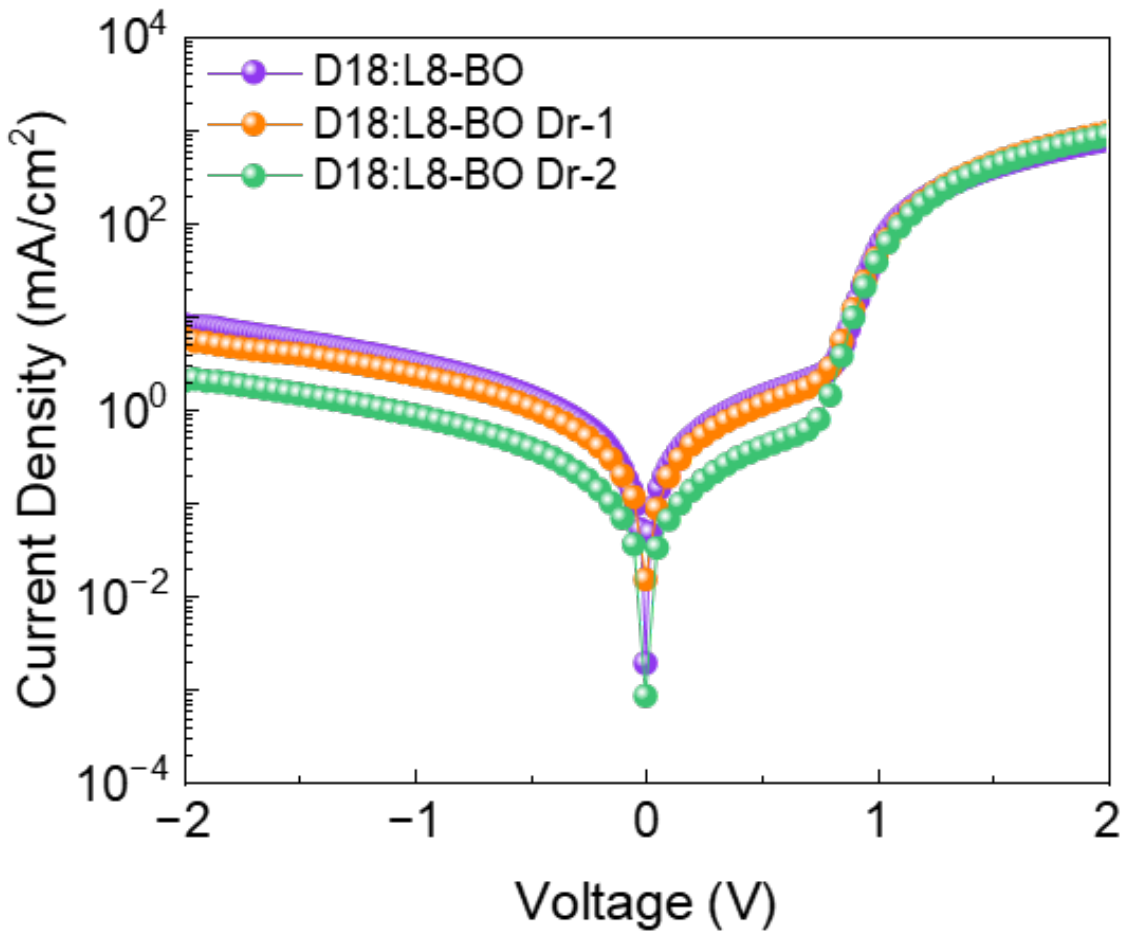

Figure R3. Dark current curves of different device.

Comment 5: Given the strong dependence of OSC performance on polymer quality, please report the molecular weight and polydispersity index of the D18 batch used in this study.

Reply 5: Thanks for the reviewer's suggestion. Thanks for the comments. We have performed the Gel Permeation Chromatography (GPC) of D18, and corresponding analysis has been added in revised manuscript (Supplementary Fig. 1, also referred to as Figure R4) as follows: The molecular weight (M_n) of D18 and its polydispersity index are 54.3 KDa and 2.18, respectively, which has been supplemented in the Supplementary Fig. 1.

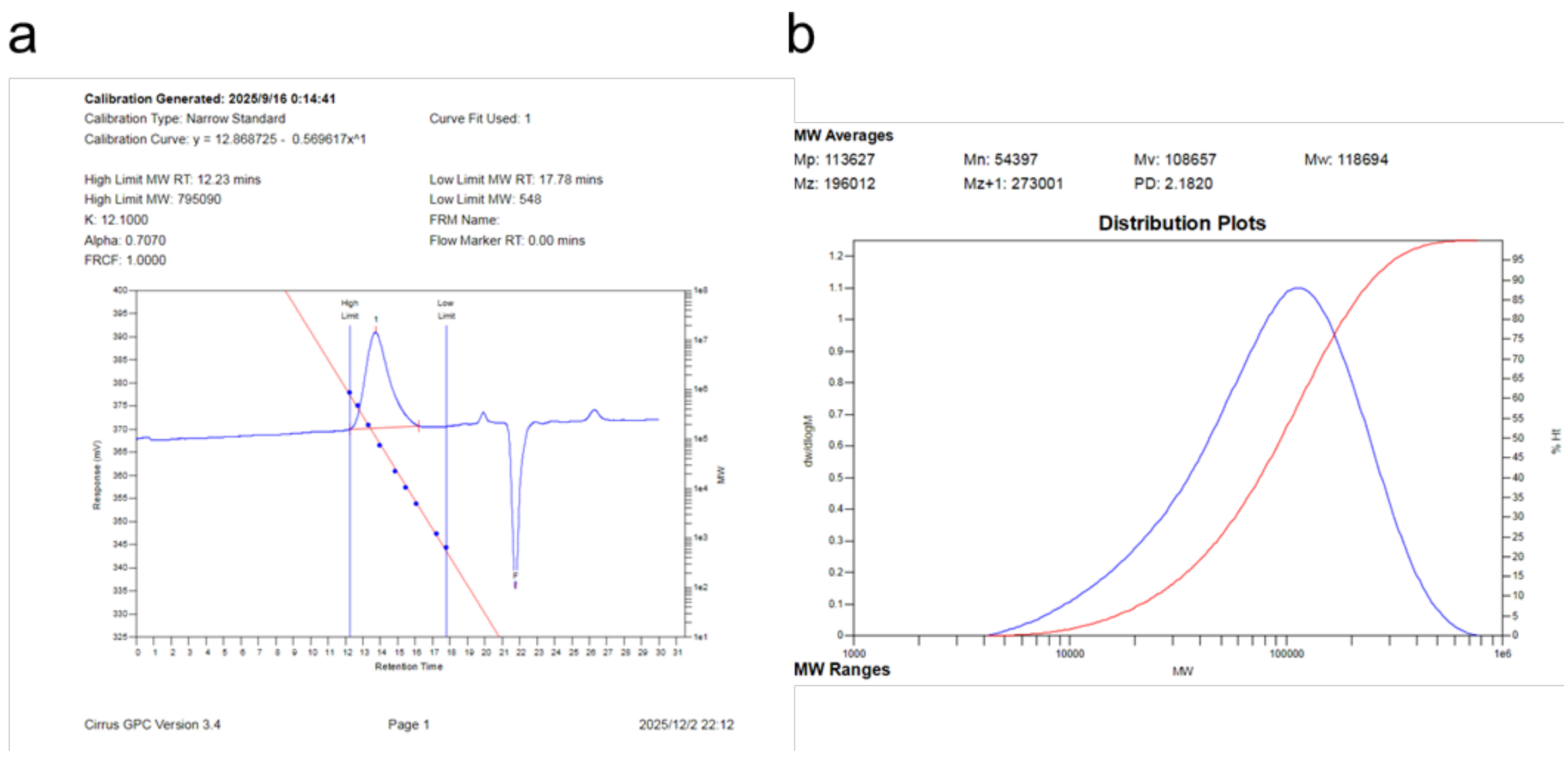

Figure R4. Gel Permeation Chromatography (GPC) trace of D18.

<u>**Comment 6:**</u> Please provide the UV absorption spectra of the two dielectric regulators.

<u>**Reply 6:**</u> We have performed the UV-vis absorption spectra of Dr-1 and Dr-2 films, and corresponding analysis has been added in revised manuscript (Supplementary Fig. 19, also referred to as Figure R5) as follows: The dielectric regulators don't exhbit absorption peaks in the visible region, thus do not cause parasitic light absorption in the active layer (Supplementary Fig. 19).

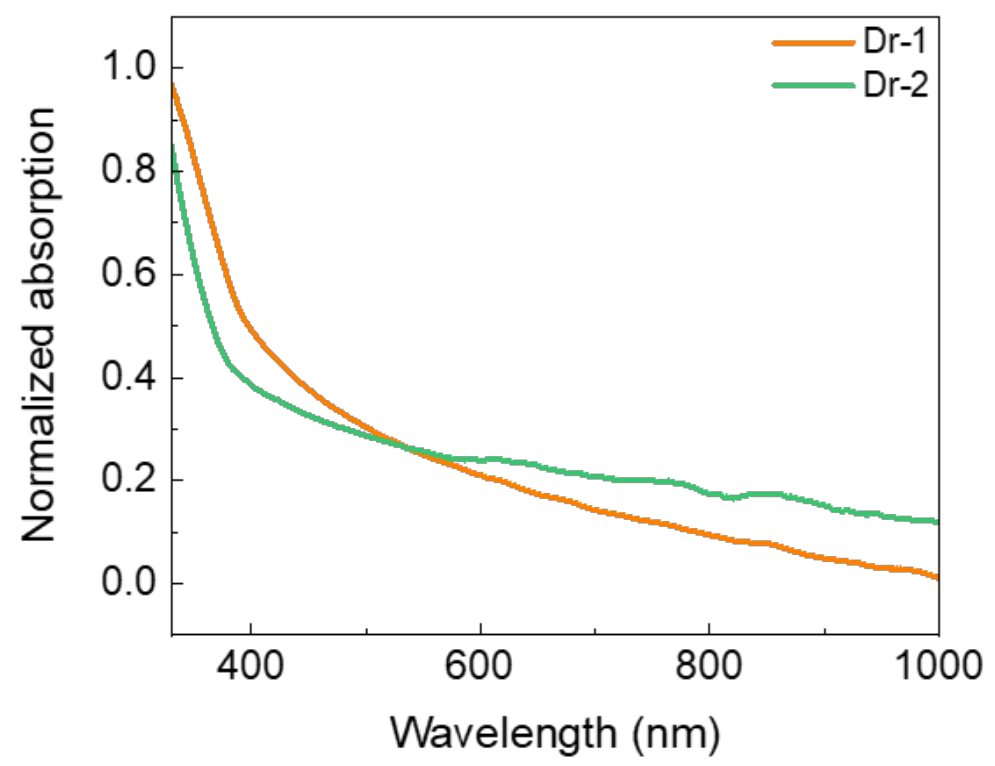

Figure R5. Normalized UV-vis absorption spectra of Dr-1 and Dr-2 films.

<u>**Comment 7:**</u> There are a few format errors in the article and references, the authors should carefully check the manuscript.

<u>**Reply 7**</u>**:** We appreciate the reviewer's comment. We have rechecked and polished the manuscript carefully.

Reviewer #2:

This manuscript reports two fluorine-isomerized dielectric modifiers (Dr-1 and Dr-2) designed to optimize exciton dissociation and charge transport by enhancing the dielectric constant of the active

layer, ultimately achieving a power conversion efficiency (PCE) of 20.85% in the D18:L8-BO system. Although the manuscript covers synthesis, characterization, device physics, and simulations, it exhibits some shortcomings in the rigor of its core scientific logic, the causality of its mechanism arguments, and the depth of its research paradigm. Consequently, it should be revised carefully.

Reply: We are grateful to the reviewer for the insightful and constructive comments. We highly value your suggestions and will meticulously revise the manuscript to address each of the points you raised, ensuring the work meets higher academic standards.

Comment 1: The manuscript attributes the performance enhancement to “increased dielectric constant,” but this represents merely an observational correlation rather than a causal demonstration. All observed performance improvements, such as accelerated exciton dissociation, enhanced charge mobility, and suppressed recombination, could entirely stem from changes in the active layer morphology, crystallinity, and molecular packing order induced by the introduction of Dr-2. These are well-known effects commonly associated with solid-state additives. The author failed to design key experiments to isolate the “dielectric effect” from the “morphology effect.” The current data only demonstrates that Dr-2 simultaneously optimizes both morphology and dielectric environment, but cannot determine which is the dominant factor.

Reply 1: We appreciate the reviewer’s insightful comment regarding the distinction between the dielectric effect and morphology-induced effects. We agree that the introduction of regulators can simultaneously influence the dielectric environment as well as the morphology, crystallinity, and molecular packing of the active layer. However, in our study the overall morphology change is not significant (Fig. 4 in manuscript and Table R1), suggesting that morphology changes alone cannot explain the observed performance enhancement.

On the other hand, the incorporation of Dr-2 leads to a clear increase (4.36 to 4.81) in the dielectric constant of the active layer, which is a significant improvement considering the typically low dielectric constants of organic semiconductors. This enhanced dielectric environment reduces Coulombic attraction between electron-hole pairs, facilitating exciton dissociation, and lowers the binding energy of charge-transfer states. These effects are supported by transient spectroscopy and charge transport measurements, which show accelerated exciton dissociation, improved free carrier generation, and reduced bimolecular recombination.

To specifically address the reviewer’s concern, we designed two complementary experiments to decouple these two effects: We summarized the device performance parameters associated with dielectric and morphology effects from the experimental characterizations of dielectric constant,

GIWAXS, AFM and PCE results (Figure R6-9 and Table R1-2, also referred to as Supplementary Fig. 47-50 and Supplementary Table 16-17).

In the first experiment, conventional solvent additives (DIO and DIB) were introduced into the D18:L8-BO system to primarily modulate the film morphology. The root-mean-square roughness derived from AFM images changes from 1.50 for pristine D18:L8-BO to 3.37 (with DIO) and 1.19 (with DIB), indicating noticeable morphology modulation. In contrast, the dielectric constant remains almost unchanged at ~4.36. These results suggest that conventional liquid additives primarily regulate film morphology without altering the dielectric environment. As a result, although a certain degree of performance improvement can still be achieved, the overall enhancement remains limited (Table R1).

In the second experiment, dielectric regulators (Dr-1 and Dr-2) were incorporated into two other systems D18:Y6 and PM6:L8-BO systems to tune the dielectric environment. Upon introduction of dielectric regulators, the dielectric constant of D18:Y6 increases from 3.43 for the pristine system to 3.66 (Dr-1) and 4.19 (Dr-2), while the active layer films maintain unchanged molecular orientation (face-on packing), and the roughness varying slightly from 1.25 to 1.27 and 1.22 nm. Similarly, for PM6:L8-BO, incorporation of dielectric regulators increases the dielectric constant from 4.23 for the pristine system to 4.57 (Dr-1) and 4.92 (Dr-2). The active layer also exhibits face-on molecular packing, and a slight decrease in roughness from 1.59 to 1.53 and 1.49 nm. These results indicate that the introduction of dielectric regulators induces minimal morphological variation, while effectively increasing the dielectric constant. Correspondingly, the significant enhancement in device performance can be primarily attributed to the elevated dielectric environment rather than morphological changes (Table R2).

Taken together, these results demonstrate that even with minimal morphological variation, increasing the dielectric constant leads to a pronounced improvement in device performance, highlighting the dominant role of the dielectric environment. To clarify this point, we have revised the manuscript (Page 15-16) to explicitly state that the increased dielectric constant contributes in conjunction with the modest morphology effects to the enhanced exciton dissociation, reduced recombination, and improved charge transport.

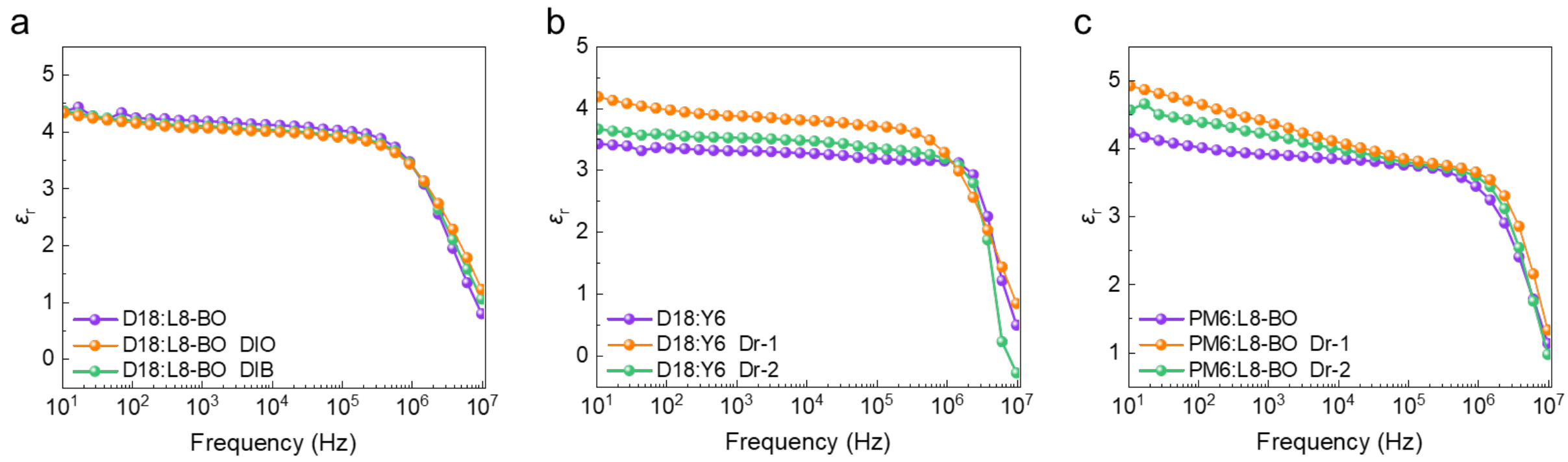

Figure R6. Dielectric constants of **a** D18:L8-BO, **b** D18:Y6, and **c** PM6:L8-BO systems with/without additives and dielectric regulators, respectively.

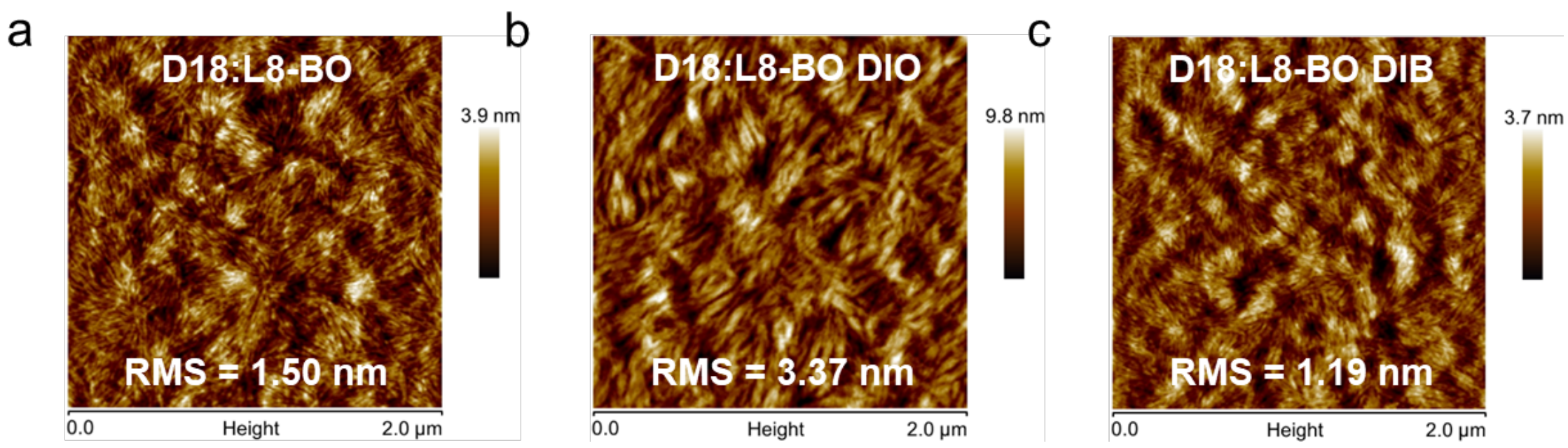

Figure R7. AFM image of D18:L8-BO system without additive, with DIO and DIB, respectively.

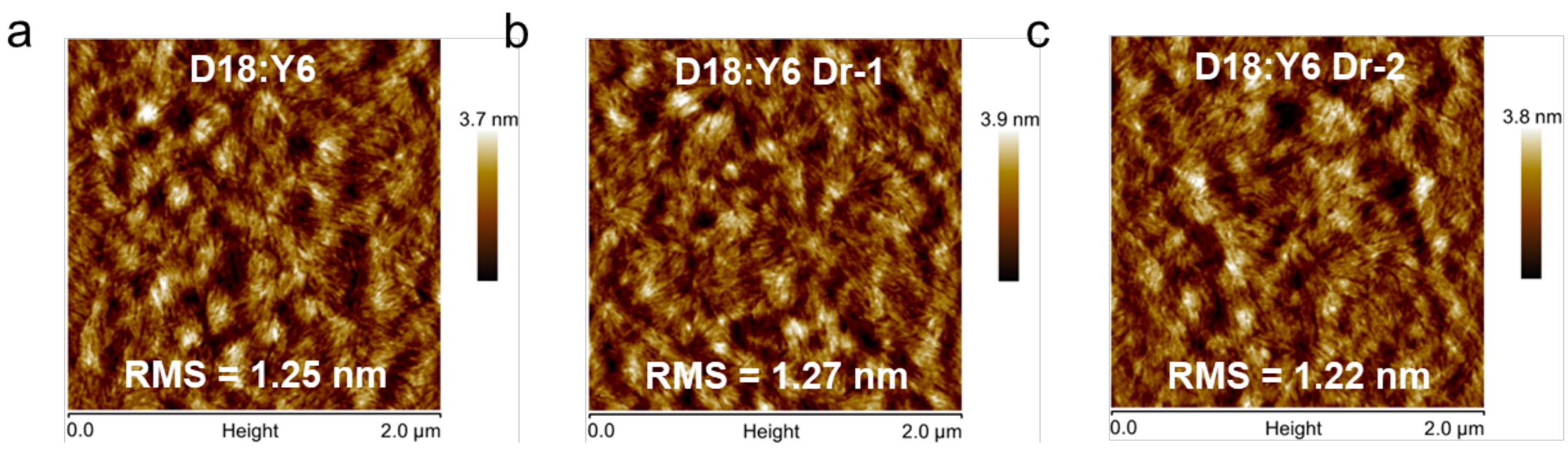

Figure R8. AFM image of D18:Y6 system with/without dielectric regulators.

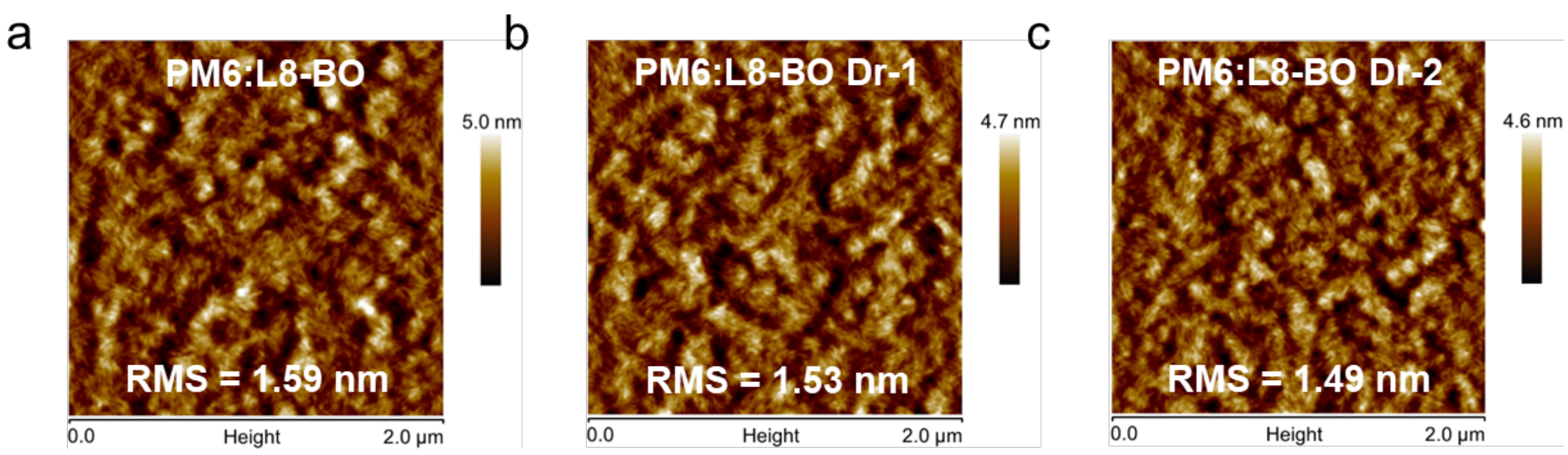

Figure R9. AFM image of PM6:L8-BO system with/without dielectric regulators.

Table R1. Summary of device performance parameters related to dielectric and morphology effects of D18:L8-BO with/without additives.

System	Additives	Dielectric constant	RMS (nm)	PCE (%)
D18:L8-BO	none	4.36	1.50	19.27
	DIO	4.33	3.37	19.46
	DIB	4.37	1.19	19.96

Table R2. Summary of device performance parameters related to dielectric and morphology effects of D18:Y6 and PM6:L8-BO with/without dielectric regulators.

System	Regulators	Dielectric constant	RMS (nm)	PCE (%)
	none	3.43	1.25	18.46
D18:Y6	Dr-1	3.66	1.27	18.86
	Dr-2	4.19	1.22	19.20
	none	4.23	1.59	17.93
PM6:L8-BO	Dr-1	4.57	1.53	19.25
	Dr-2	4.92	1.49	19.62

Comment 2: The manuscript claims to provide a "general strategy" for designing high-performance dielectric regulators. However, only Dr-1 and Dr-2, a pair of closely related structures, are presented. The structural optimization is very limited and cannot substantiate such a strong conclusion as a "universal molecular design principle."

Reply 2: Thank you for the comments. We would like to clarify that we did not intend to emphasize a universal molecular design principle. Instead, as shown in (Supplementary Table. 19, also referred to as Table R3), our aim was to demonstrate that the proposed strategy can be extended to a broader range of donor-acceptor systems.

Also, in order to avoid over-claim, we rephrase our manuscript as follows: "To investigate the generality of this strategy, several donor:accpetor systems were treated with/without dielectric regulators"

Table R3. Research of different active layer systems with/without dielectric regulators.

Active Layer	Condition	V_{oc} (V)	J_{sc} (mA cm^{-2})	FF (%)	PCE (%)
	None	0.886	26.42	76.56	17.93
PM6:L8-BO	Dr-1	0.886	27.47	79.06	19.25
	Dr-2	0.887	27.50	80.46	19.62
	None	0.830	27.92	67.12	15.55
PM6:Y6	Dr-1	0.834	28.82	78.92	18.97
	Dr-2	0.840	28.97	78.75	19.16
	None	0.831	28.65	77.56	18.46
D18:Y6	Dr-1	0.830	28.93	78.53	18.86
	Dr-2	0.840	29.11	78.51	19.20

Comment 3: The authors repeatedly emphasize that Dr-2 improves device performance by increasing ε_r, thereby reducing E_b and lowering recombination rates. Yet Dr-2 also significantly alters the blend-film morphology, molecular packing, and charge-carrier mobility and balance, factors that themselves have a substantial impact on charge generation, transport, and recombination. The present experimental design, therefore, makes it difficult to distinguish the "pure dielectric effect" from the

contributions of improved morphology and packing. Several discussions in the paper diverge after this point.

Reply 3: We thank the reviewer for raising this important point. We agree that Dr-2 induces concurrent changes in the dielectric environment, film morphology, and charge-transport properties, which are intrinsically coupled in OSCs. However, we respectfully argue that the increase in ε_r plays the primary and initiating role, while the morphological and transport improvements act as secondary or amplifying effects.

Importantly, several key observations cannot be sufficiently explained by morphology optimization alone. First, the pronounced reduction in exciton binding energy, together with the accelerated exciton dissociation dynamics and suppressed recombination, are all directly linked to enhanced Coulomb screening, which is a consequence of an increased dielectric constant. Conventional morphology-driven effects, such as enhanced crystallinity or domain purity, typically improve charge transport but do not directly account for the observed stabilization of charge-transfer states and reduced recombination. Second, while Dr-2 does modify molecular packing and carrier mobility, the extent of these morphological changes is relatively moderate and does not scale proportionally with the substantial improvements in charge generation efficiency and recombination suppression.

As shown in Reply 1, we conducted a comprehensive study and designed two experiments to address the reviewer's concern. We summarize the device performance parameters associated with dielectric and morphology effects from the experimental characterizations (Table R1-2). Based on these results, we clarify in the revised manuscript that Dr-2 improves device performance primarily by increasing ε_r and reducing Coulomb interactions, which facilitates charge separation and suppresses recombination, while the accompanying morphology and packing improvements further reinforce these benefits. The discussion has been carefully revised to better reflect this causal hierarchy and to avoid ambiguity regarding the dominant role of the dielectric effect.

Comment 4: The manuscript reports excessively high J_{SC} values, with EQE clearly exceeding 92%, which is inconsistent with objective principles.

Reply 4: To clarify the high EQE values and provide a more comprehensive understanding of the photoelectric conversion process, we have measured the transmittance spectra of Glass/ITO/2PACZ and analyzed the internal quantum efficiency (IQE) of the devices. It can be seen that the transmittance of Glass/ITO/2PACZ can be up to 94.49%, since customized ITO glass with high transmittance (⩾ 94.5%) was used in this work (Figure R10). Moreover, the IQE curves of all the devices are provided

in Figure R11, and corresponding analysis has been added in revised manuscript (Supplementary Fig. 22) as follows: High IQE values over 95% in the wavelength range of 400-600 nm can be observed, suggesting that high percentage of absorbed photons resulted in efficient electron collection. The make and model of the ITO (South China Science & Technology Company Limited, 15Ω, transmittance ≥ 94.5%) has been included in the experimental section. Additionally, high EQE values over 93% have been reported in prior literature, showing that our result (over 92%) is not an isolated case (Table R4).

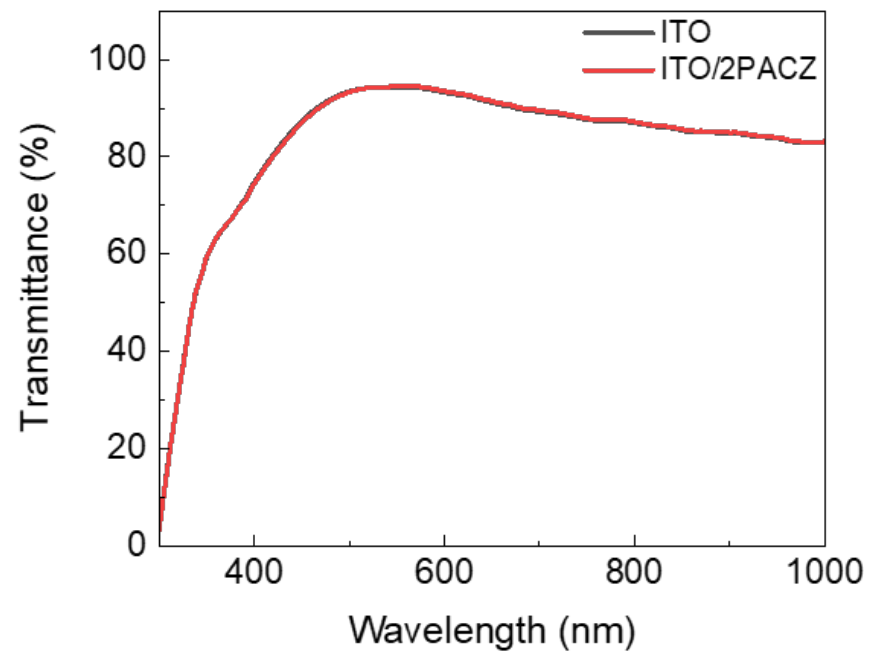

Figure R10. The transmittance spectra of Glass/ITO and Glass/ITO/2PACZ.

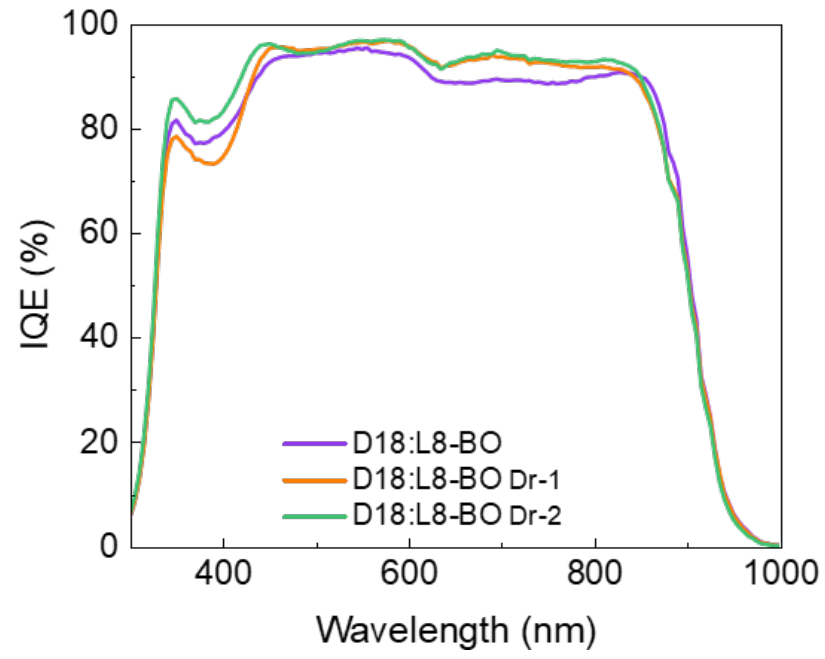

Figure R11. The internal quantum efficiency curves of devices with/without regulators.

Table R4. The maximum value of EQE reported in the literature.

EQE (%)	References
~94	*Nat. Energy*, 2022, 7, 1076-1086.
~93	*Nat. Mater.*, 2025, 24, 1626-1634.
>93	*Nat Energy*, 2025, 10, 1251-1261.
~94	*Nat. Mater.*, 2025, 24, 444-453.
~93	*Nat. Mater.*,2025, 24, 433-443.

Comment 5: The "macroscopic" dielectric constant (~4.3 to 4.8) measured via the thin-film capacitance method exhibits minimal variation and fails to reflect the true changes in the local dielectric environment at the donor/acceptor interface-the very factor critical for exciton dissociation and charge separation. The absence of direct detection of local polarization capabilities at the interface (e.g., via photoabsorption spectroscopy) leaves the core argument built upon fragile foundations.

Reply 5: We sincerely thank the reviewer for this insightful and important comment. Owing to the intrinsic characteristics of organic semiconductors such as the dominance of carbon-based backbones,

weak intermolecular polarization, and structural disorder, the relative ε_r is generally low and difficult to substantially enhance. Prior studies on dielectric constant modulation in high-performance OSCs suggest that the achievable enhancement in dielectric constant is limited to a relatively narrow range (Table R5). In this context, the observed increase from ~4.3 to ~4.8, although seemingly modest, is consistent with the range of dielectric modulation reported in high-performance OSC systems and is considered non-negligible. More importantly, even a moderate increase in dielectric constant can lead to a reduction in Coulombic attraction between electron-hole pairs, thereby facilitating exciton dissociation and charge separation. The fact that this dielectric change is accompanied by a pronounced improvement in device performance further supports its physical relevance.

Regarding the reviewer's suggestion on direct probing of local polarization (e.g., via photoabsorption spectroscopy), we agree that such techniques would provide valuable additional insight. We first performed KPFM measurements on the thin films (~100 nm) under dark conditions to characterize the surface potential variations, and the results have been added to the revised manuscript (Supplementary Fig. 51, also referred to as Figure R12). KPFM provides spatially resolved surface potential information that is sensitive to local charge distribution and electrostatic potential variations, and can thus serve as a probe of local polarization behavior. Upon the introduction of dielectric regulators, the surface potential variation of the thin film in the dark state were increased from 0.150 V (without) to 0.164 V (Dr-1) and 0.170 V (Dr-2). This enhancement suggests that the dielectric regulators induce measurable changes in the local electrostatic environment, which is consistent with enhanced polarization capability.

Table R5. The changes in dielectric constant (ε_r) of high-performance organic solar cells in recent literature.

**References**	ε_r	$\triangle\varepsilon_r$
*Adv.Mater.*2022, *34*, 2202089	2.36 → 2.50	0.14
J. Am. Chem. Soc. 2023, *145*, 13686−13695	2.77 → 3.28	0.51
Adv. Mater. 2024, *36*, 2403294	4.17 → 4.82	0.65

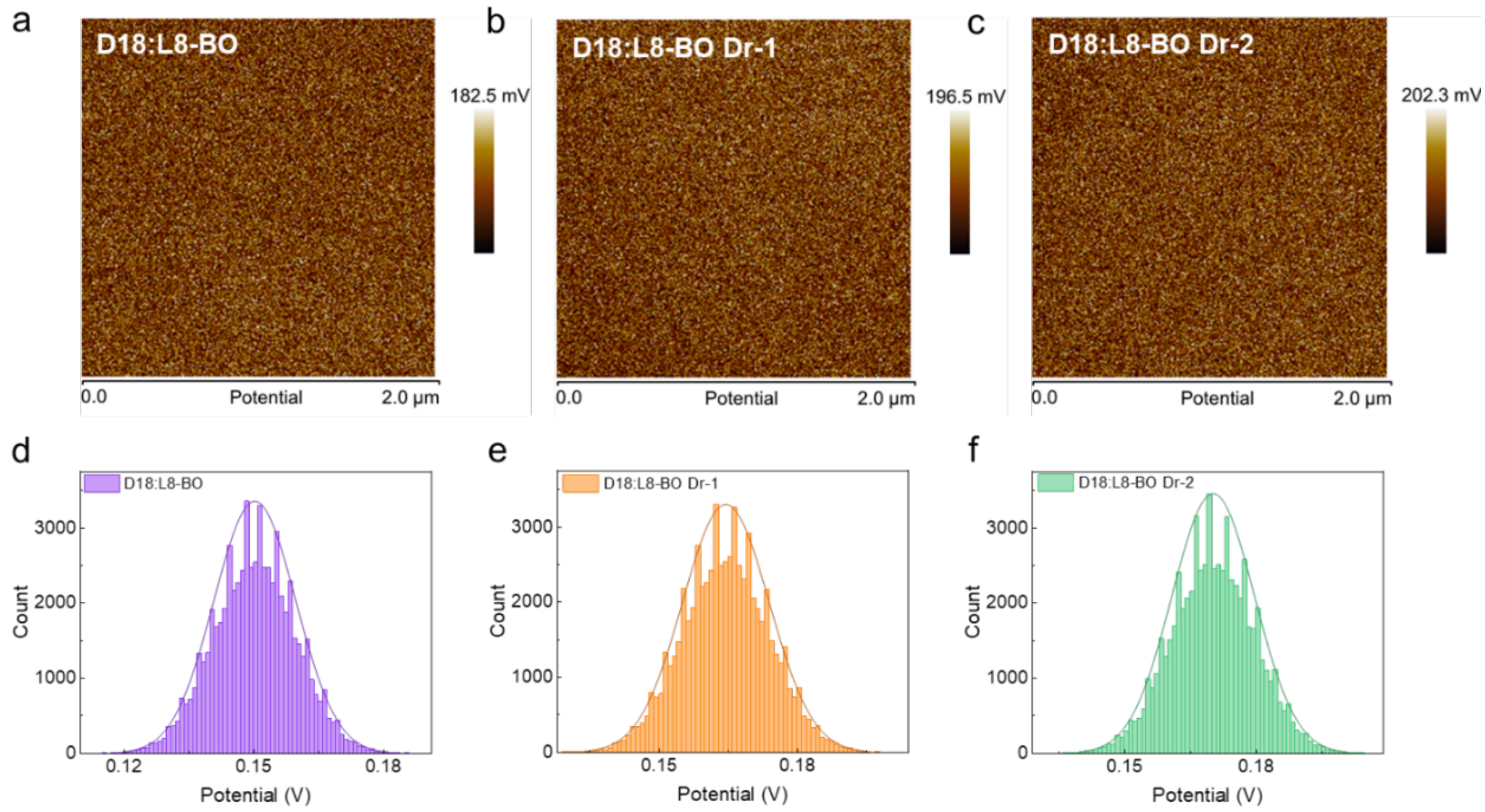

Figure R12. Kelvin probe atomic force microscopy (KPFM) images and surface contact potential distributions of D18:L8-BO, D18:L8-BO Dr-1 and D18:L8-BO Dr-2 films (~100 nm) under dark, respectively.

Furthermore, transient photovoltage (TPV) decay kinetics measurements were employed to determine the charge carrier lifetime and quantify the charge recombination processes within the devices. The charge recombination lifetime (τ_{rec}) of the D18:L8-BO Dr-2 device was 797.1 µs, which is longer than that of the pristine D18:L8-BO device (540.7 µs) and the D18:L8-BO Dr-1 device (675.2 µs), indicating a reduced charge recombination propensity in the Dr-2-treated device (Figure R13, also referred to as Supplementary Fig. 27a). Transient photocurrent (TPC) decay measurements were conducted to investigate the charge extraction characteristics of the devices. The D18:L8-BO Dr-2 device exhibited a shorter charge extraction time (τ_{ext} = 0.31 µs) than the pristine D18:L8-BO (0.46 µs) and Dr-1 (0.39 µs) counterparts, indicative of fast and efficient charge extraction for the Dr-2-processed device (Figure R13, also referred to as Supplementary Fig. 27b). The combined evidence from KPFM and device-level transient measurements supports the conclusion that the incorporation of dielectric regulators leads to a meaningful modulation of the local electrostatic environment, which in turn facilitates charge separation and suppresses recombination.

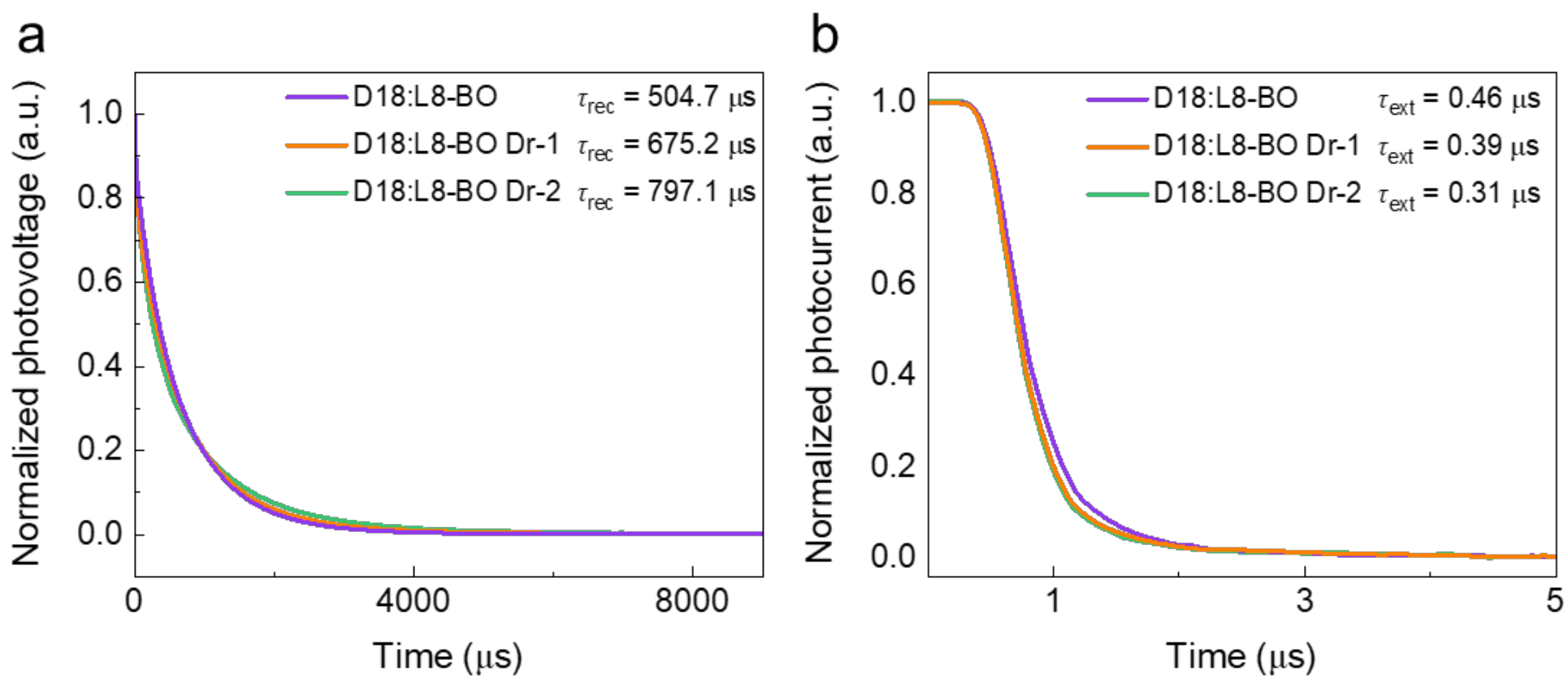

Figure R13. a TPV, and **b** TPC curves of device with/without regulators.

Comment 6: The manuscript employs ESP analysis, FTIR red shift, and MD simulations to demonstrate the “strong interaction” between Dr-2 and L8-BO. However, the FTIR red shift of only 1-3 cm^{-1} may fall within the range of instrumental error and does not constitute quantitative evidence of interaction strength. The accuracy of force field parameters used in MD simulations for describing such weak noncovalent interactions is questionable. More conclusive evidence, such as solid-state NMR, is lacking.

Reply 6: To verify the reliability of the observed redshift, the FTIR test under each condition was performed in three replicate measurements with strict control of experimental variables (e.g., sample preparation, test temperature, spectral resolution). The red shift was a reproducible observation in all three independent measurements, with no significant deviation among replicate data (Supplementary Fig. 16, also referred to as Figure R14). This consistency confirms that the observed spectral shift is a genuine spectral response to the intermolecular interaction rather than random instrumental error. We have added this key experimental detail in the revised manuscript to make the data more rigorous.

To verify the intermolecular interactions, we employed two-dimensional (2D) liquid-state nuclear magnetic resonance (NMR) spectroscopy with higher resolution for characterization. Corresponding analysis has been added in revised manuscript (Figure 1c, also referred to as Figure R15) as follows: Owing to the nuclear Overhauser effect, NOESY spectra in 2D NMR furnish information about protons that are ≤ 5 Å apart in space. In the 2D 1H−1H NOESY spectra of the L8-BO:Dr-2 solution, a distinct cross-peak between the 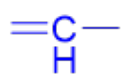 protons of Dr-2 (6.98 ppm) and the 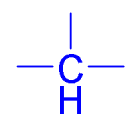 protons of L8-BO (3.18 ppm) were clearly detected, which can verify the possibility of intermolecular interaction between L8-BO and Dr-2.

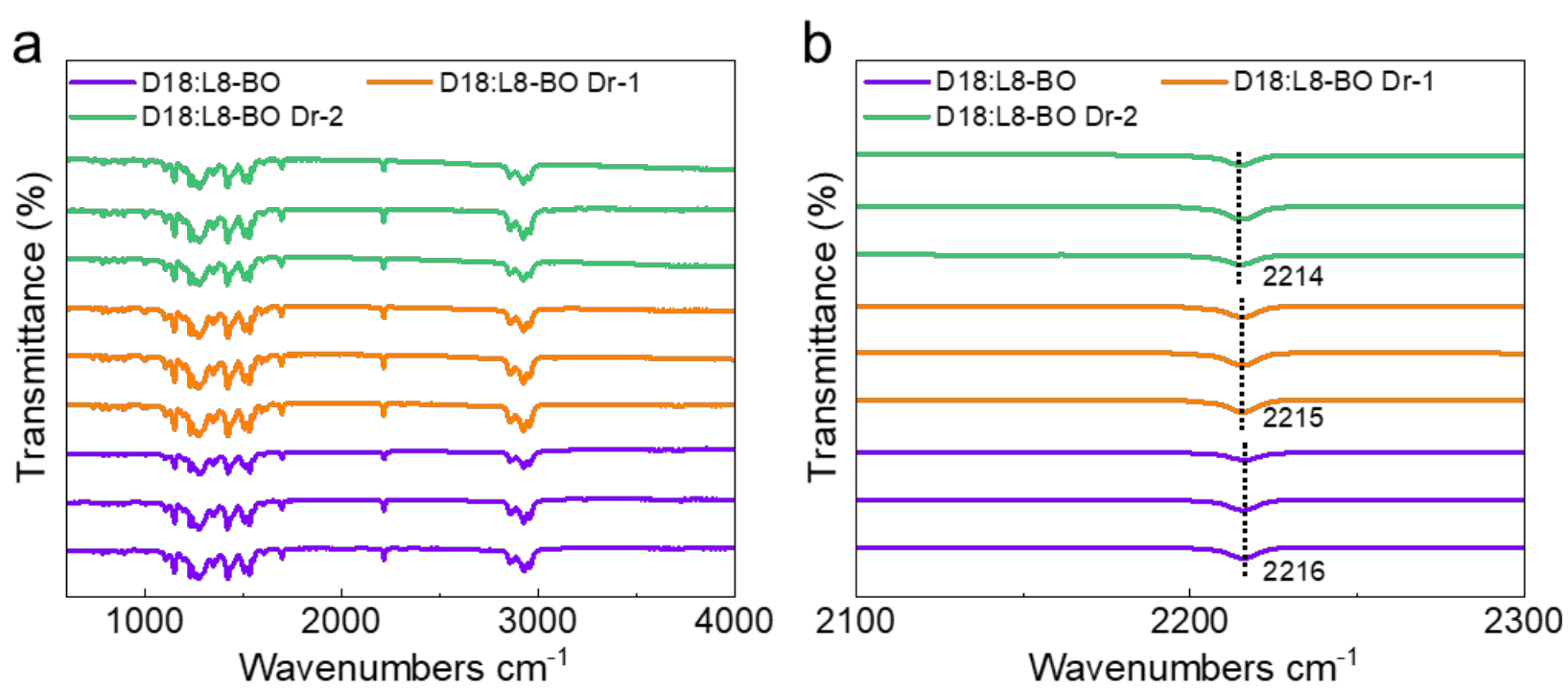

Figure R14. FTIR of pure and blend films with/without regulator materials.

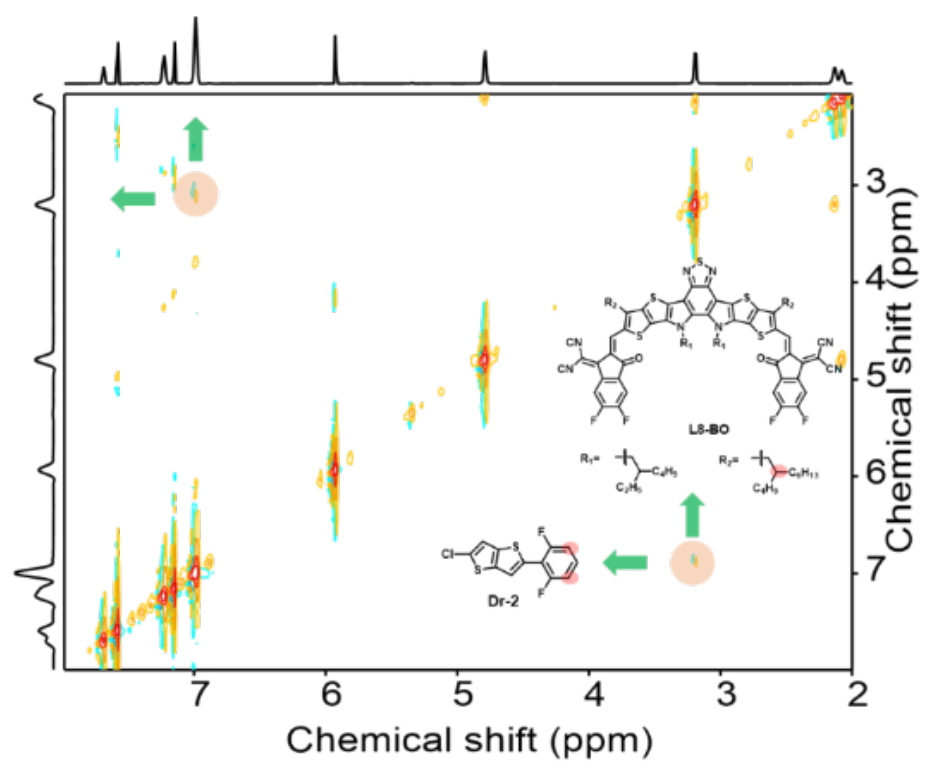

Figure R15. 2D ^{1}H–^{1}H NMR spectra of L8-BO:Dr-2 in deuterated tetrachloroethane (d-TCE) solution.

Comment 7: The failure to distinguish characteristic times for processes such as exciton dissociation, charge separation, and charge recombination through femtosecond transient absorption global fitting prevents the kinetic data from providing effective support for dielectric regulation mechanisms.

Reply 7: We appreciate the reviewer's insightful comment regarding the lack of distinguished characteristic times for key photophysical processes in the original fs-TA global fitting. Based on this suggestion, we reprocessed the fs-TA dataset with an optimized fitting model that constrains the kinetic parameters of different photophysical processes, thus realizing the accurate separation of their characteristic times. Combined with our previously characterized charge carrier lifetime (TPV) and charge extraction properties (TPC), the revised fs-TA kinetic results now form a complete photophysical picture and provide compelling experimental support for the dielectric regulation mechanism of the Dr-2 regulator. Corresponding analysis has been added in revised manuscript (Supplementary Fig. 36 and Supplementary Table 11, also referred to as Figure R16 and Tabe R6) as follows: In transient absorption spectroscopy, exciton (EX) dissociation and charge-separated (CS) states in D18:L8-BO blend films were investigated via the analysis using singular value decomposition (SVD) and global fitting algorithms (Supplementary Fig. 36 and Supplementary Table 11). The decay of the EX state is ascribed to rapid hole injection and concurrent exciton dissociation. The Dr-2-treated D18:L8-BO film exhibits a faster EX state decay lifetime (17.08 ps) than the pristine D18:L8-BO film (17.21 ps) and Dr-1-modified D18:L8-BO film (17.19 ps), which indicates accelerated hole injection and concomitant exciton dissociation enabled by Dr-2. The CS reaches its maximum concentration at 30 ps, after which a slow recombination process occurs. The Dr-2-treated D18:L8-BO film shows an extended CS state decay lifetime (244.46 ns) compared to the pristine D18:L8-BO film (213.52 ns) and the Dr-1-modified D18:L8-BO film (236.66 ns), a result that facilitates the suppression of charge recombination via the modulation of Dr-2. Therefore, the enhanced photovoltaic performance of the corresponding OSC devices primarily originates from the accelerated hole injection, efficient exciton dissociation, and suppressed charge recombination in the Dr-2-treated D18:L8-BO film.

The data is well described by a double exponential decay model as follows:

$$y = A_1 \exp(-x/\tau_1) + A_2 \exp(-x/\tau_2) + y_0$$

Thus, τ_1 and τ_2 along with their relative amplitudes A_1 and A_2 are gained by Origin Pro. The average lifetime $\langle\tau\rangle$ is subsequently calculated from these values using the following equation:

$$\langle\tau\rangle = (A_1\tau_1^2 + A_2\tau_2^2) / (A_1\tau_1 + A_2\tau_2)$$

Furthermore, transient photovoltage (TPV) decay kinetics measurements were employed to determine the charge carrier lifetime and quantify the charge recombination processes within the devices. The charge recombination lifetime (τ_{rec}) of the D18:L8-BO Dr-2 device was 797.1 μs, which is longer than that of the pristine D18:L8-BO device (540.7 μs) and the D18:L8-BO Dr-1 device (675.2 μs), indicating a reduced charge recombination propensity in the Dr-2-treated device (Figure R13, also referred to as Supplementary Fig. 27a). Transient photocurrent (TPC) decay measurements were conducted to investigate the charge extraction characteristics of the devices. The D18:L8-BO Dr-2 device exhibited a shorter charge extraction time (τ_{ext} = 0.31 μs) than the pristine D18:L8-BO (0.46 μs) and Dr-1 (0.39 μs) counterparts, indicative of fast and efficient charge extraction for the Dr-2-processed device (Figure R13, also referred to as Supplementary Fig. 27b).

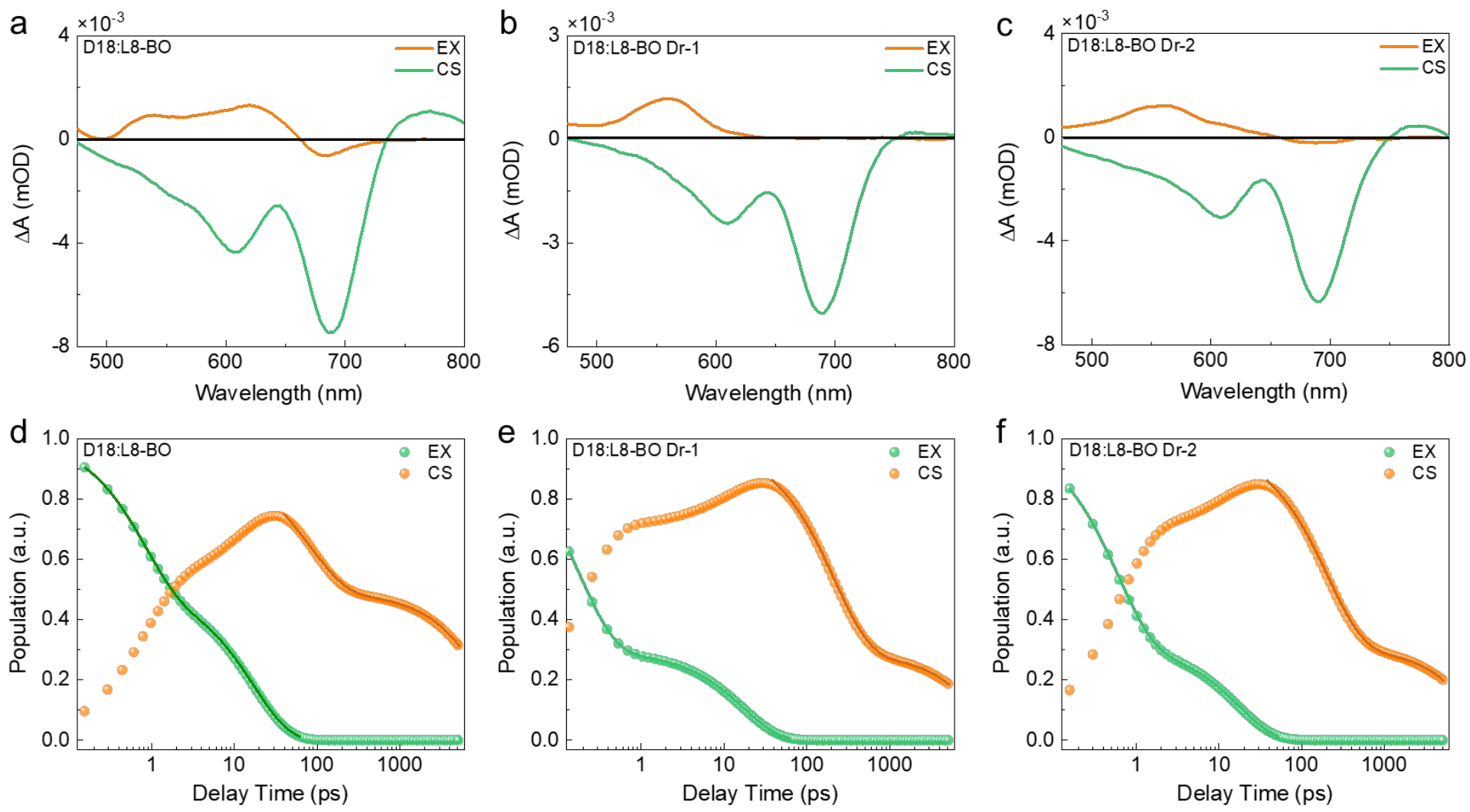

Figure R16. a-c EX and CS spectra and **d-f** kinetics extracted by SVD of the film with/without regulators.

Table R6. EX lifetime and CS lifetime of D18: L8-BO film with/without regulators.

lifetime	D18: L8-BO	D18: L8-BO Dr-1	D18: L8-BO Dr-2
EX lifetime (ps)	17.21	17.19	17.08
Lifetime of CS state decay (ns)	213.52	236.66	244.46

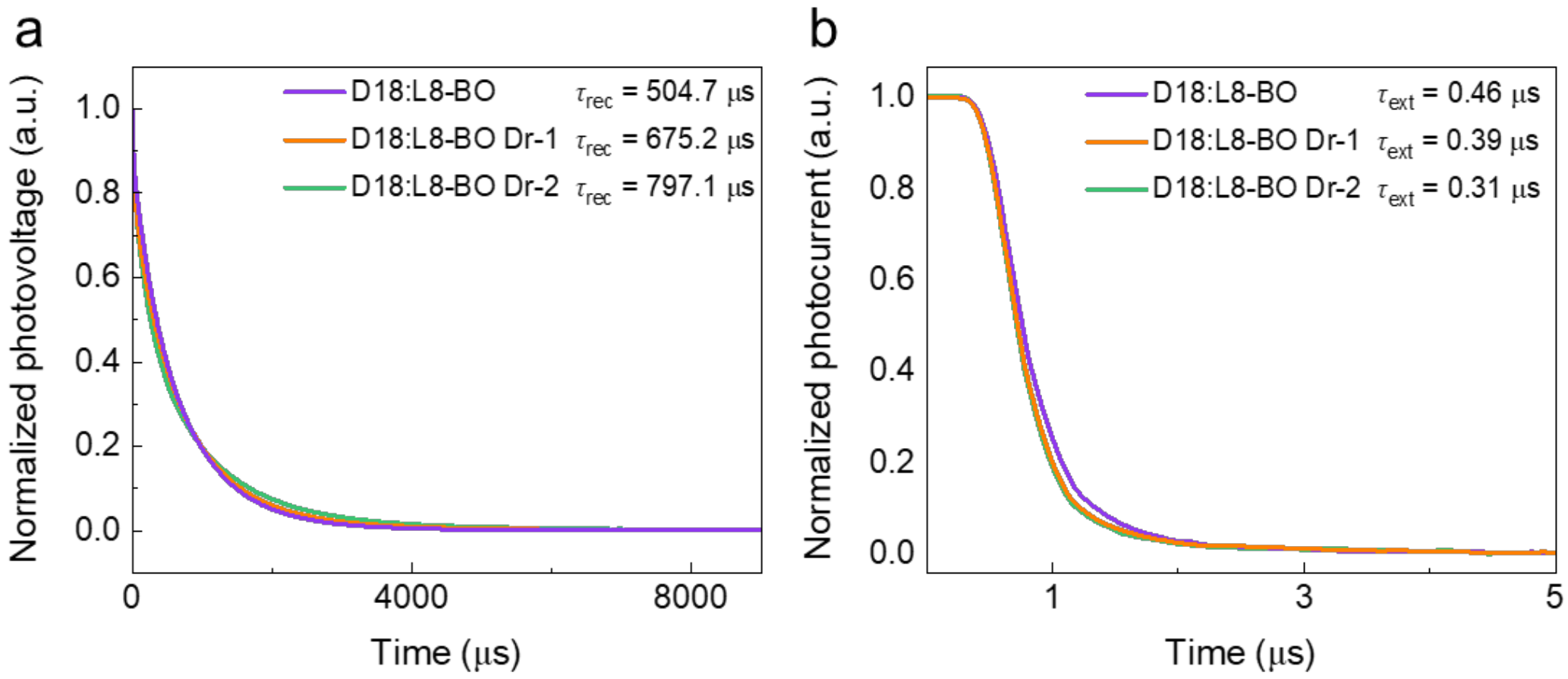

Figure R13. a TPV, and **b** TPC curves of device with/without regulators.

<u>Comment 8:</u> The manuscript fails to demonstrate changes in charge transport length within thick films using techniques such as scanning Kelvin probe microscopy (SKPM), thereby lacking proof of substantial improvement in thick-film charge transport through dielectric regulation. Furthermore, the lack of comparison between the external quantum efficiency (EQE) spectra of thick-film and thin-film devices precludes ruling out the contribution of enhanced light absorption in thick films to J_{sc}. Consequently, the conclusion that "dielectric modulation suppresses recombination in thick films" lacks direct evidence.

<u>Reply 8:</u> The Kelvin probe force microscopy (KPFM) characterization of D18:L8-BO, D18:L8-BO Dr-1 and D18:L8-BO Dr-2 films with 300 nm were measured, and corresponding surface contact potential distributions are presented in Figure R17 (also referred to as Supplementary Fig. 52). The corresponding analysis has been added in revised manuscript as follows: For the thick film (300 nm), the full width at half maximum (FWHM) distributions of the electrostatic potential in the dark state are 16.1, 15.5, and 15.1 mV for D18:L8-BO, D18:L8-BO Dr-1, and D18:L8-BO Dr-2 films, respectively (Supplementary Fig. 52). The uniform surface potential distribution achieved upon the introduction of regulators is highly beneficial for efficient charge transport and for minimizing charge recombination (*Science*, 2021, *373*, 561-567. *Adv. Mater.*, 2025, DOI: 10.1002/adma.202514741.). Charge formation is manifested as a shift in the surface potential under illumination compared to the dark state ($\Delta SP_{light-dark}$). KPFM characterizations were conducted over the same sample region under three separate conditions: (i) without irradiation ("dark 1"), (ii) under light irradiation ("light"), and (iii) after the cessation of illumination ("dark 2"), employing an experimental configuration detailed in prior work (*Energ. Environ. Sci.*, 2013, *6*, 1565-1572). Upon switching off the illumination, the surface potential immediately reverted to its initial dark-state value. The D18:L8-BO Dr-2 blend film exhibits the highest $\Delta SP_{light-dark}$ of 137 mV, in contrast to 95 and 126 mV for both the pristine D18:L8-

BO and D18:L8-BO Dr-1 films. This result demonstrates that the Dr-2 treatment promotes a higher level of charge accumulation, which in turn facilitates the enhancement of photocurrent density.

We agree that in thick-film devices, enhanced light absorption may contribute to the increase in J_{sc}, and thus it is necessary to distinguish optical effects from electrical improvements. We have compared the external quantum efficiency (EQE) spectra of thin- and thick-film devices (Figure R18). The comparison shows that while the thick-film devices exhibit slightly enhanced photo-response in certain wavelength regions, the overall enhancement in EQE is not sufficient to fully account for the observed increase in J_{sc}. This suggests that optical absorption is not the dominant factor. More importantly, the dielectric-regulated devices exhibit consistently improved charge collection efficiency across the spectral range, rather than a localized enhancement associated with increased absorption.

Besides, to investigate the effect of dielectric modulation on charge recombination in thick films, we studied the dependence of the V_{oc} and J_{sc} on light intensity, with the results presented in Figure R19 (also referred to as Supplementary Fig. 30). The corresponding analysis has been added in revised manuscript as follows: For thick film devices, the D18:L8-BO, D18:L8-BO Dr-1 and D18:L8-BO Dr-2 devices show the slopes of 1.38, 1.33 and 1.26 of charge recombination kinetics, indicating effective suppression of trap-assistant recombination with the assistance of regulators (Supplementary Fig. 30a). For the bimolecular recombination dynamics, the D18:L8-BO Dr-2 device with α value of 0.991 is the higher than those D18:L8-BO and D18:L8-BO Dr-1 devices, suggesting the effective mitigation of bimolecular recombination after the treat of Dr-2 (Supplementary Fig. 30b). These results provide strong evidence that the improved device performance, particularly in thick films, primarily originates from suppressed recombination, rather than solely from enhanced light absorption.

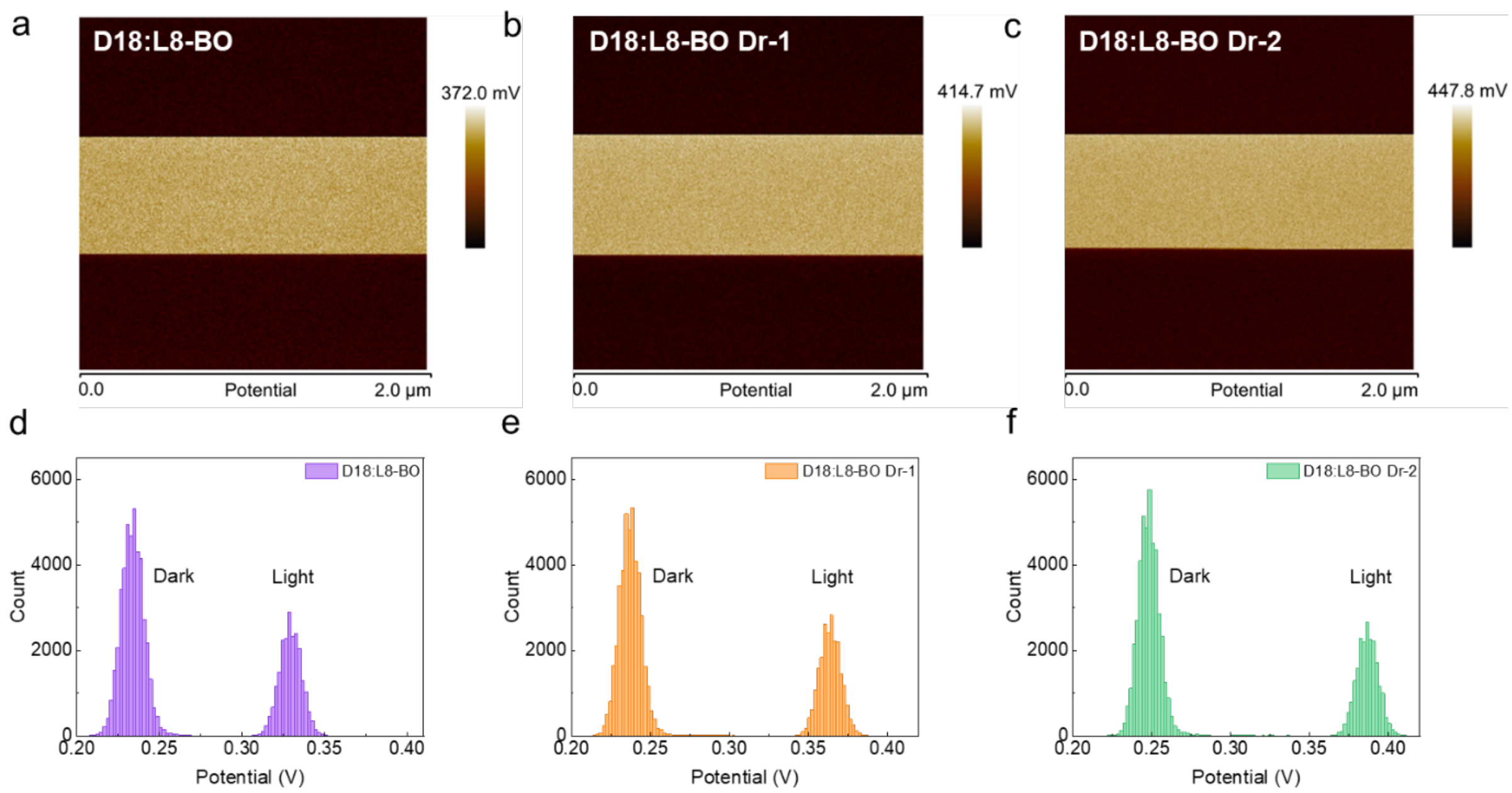

Figure R17. Kelvin probe atomic force microscopy (KPFM) images and surface contact potential distributions of thick-film based on D18:L8-BO, D18:L8-BO Dr-1 and D18:L8-BO Dr-2 systems under dark and illumination, respectively.

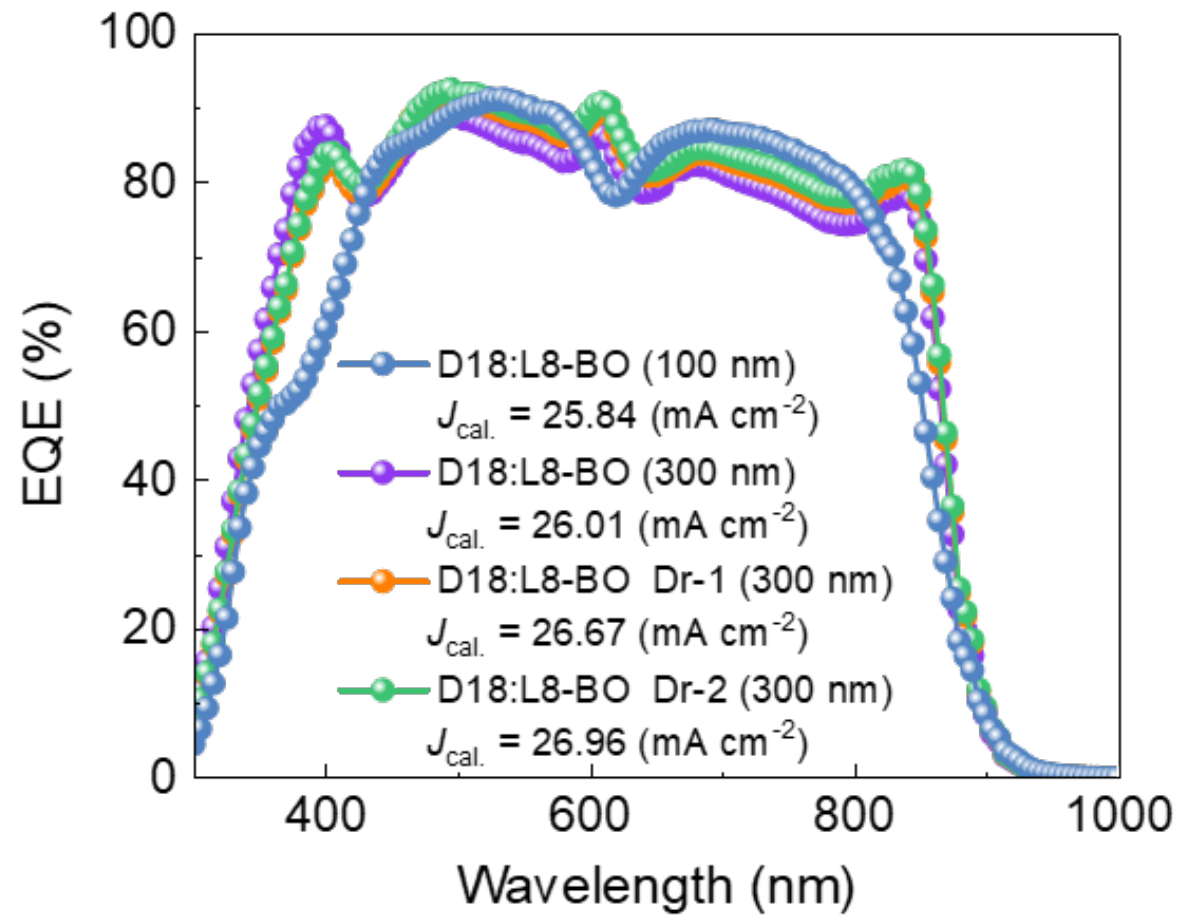

Figure R18. a V_{oc} versus EQE curves of **a** thin-film (~100 nm) and **b** thick-film (~300 nm) devices.

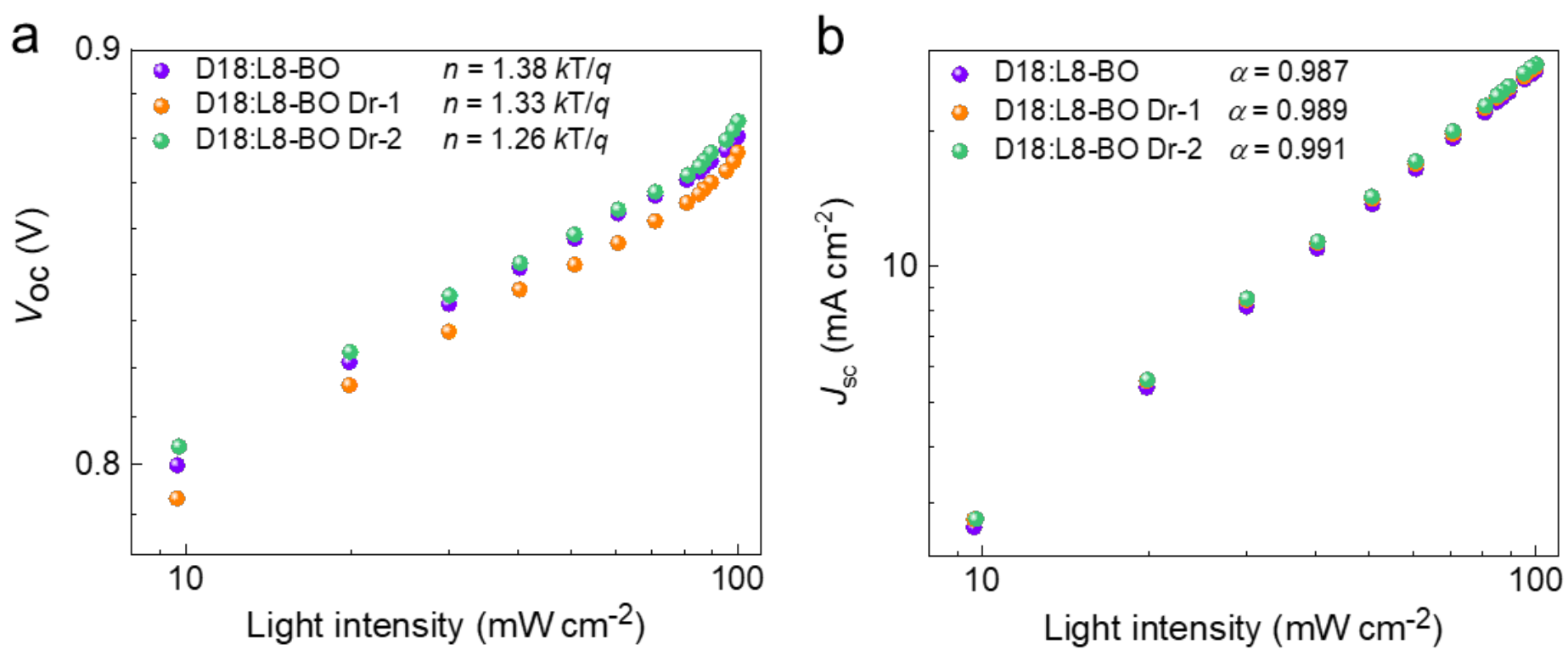

Figure R19. a V_{oc} versus light intensity plots and **b** J_{sc} versus light intensity plots of D18:L8-BO systems (300 nm) with/without dielectric regulators.

Comment 9: Stability is a core metric for the practical application of OSCs, yet the manuscript did not conduct any stability assessment experiments (such as atmospheric stability, thermal stability, or light stability).

Reply 9: We compared the photostability of the devices with/without dielectric regulators (Figure R20, also referred to as Supplementary Fig. 24). Corresponding descriptions have been added in the revised manuscript as follows: Characterization via maximum power point (MPP) tracking demonstrated that device photostability is enhanced upon incorporation of dielectric regulators. A 20% PCE loss was observed at 350 h and 500 h for the D18:L8-BO and D18:L8-BO Dr-1 devices under simulated AM1.5G irradiation (100 mW cm^{-2}) (Supplementary Fig. 24). By stark contrast, the Dr-2-treated device preserved 82.1% of its initial efficiency after 1000 h of testing.

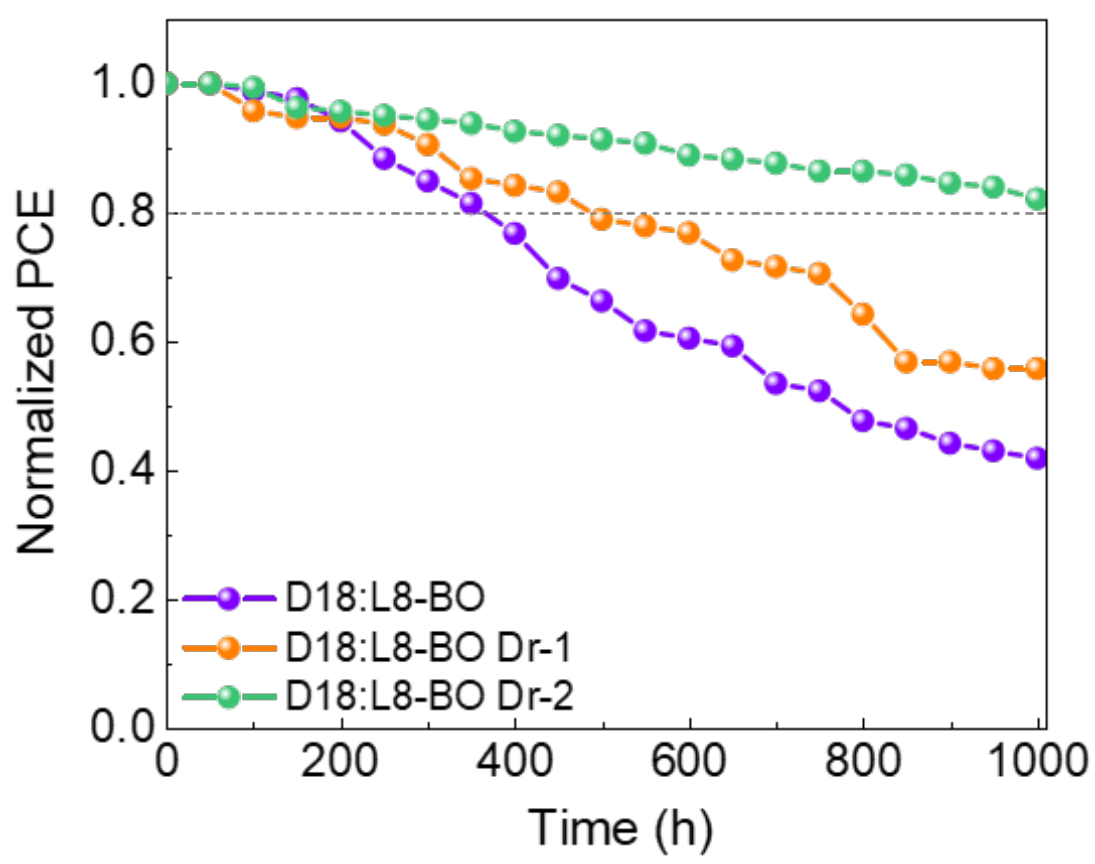

Figure R20. The photostability of the devices with/without dielectric regulators (encapsulated, AM 1.5G radiation with illumination of 100 mW cm^{-2}).

Reviewer #3:

Comment 1: This work enhances exciton dissociation and charge transfer by modulating dielectric constants through additive engineering, thereby achieving high-efficiency organic photovoltaic cells. The primary issue is that similar studies have been extensively reported in the literature. For instance, "Elevating dielectric constant via additive engineering: Achieving 19.23% certified efficiency in thick-film binary organic solar cells, Joule, 2025, 9, 102135," and several reviews have summarized similar additive effects, such as "Dielectric Constant: A Key Factor Limiting the Future Development of Organic Solar Cells, Solar RRL, 2025, 9, e202500608."

Additionally, there is a large amount of literature and reviews on dielectric engineering, For instance, "Dielectric constant engineering of nonfullerene acceptors enables a record fill factor of 83.58% and a high efficiency of 20.80% in organic solar cells, Energy Environ. Sci., 2025, 18, 4982–4995," and "On the Understandings of Dielectric Constant and Its Impacts on the Photovoltaic Efficiency in Organic Solar Cells, Chinese Journal of Chemistry, 2021, 39, 381–390."

The previously published work demonstrates the lack of innovation in this study.

Reply 1: We are grateful for your thorough review and valuable suggestions. In regard to your concern about the novelty of our research, we would like to take this chance to clarify its unique contributions. The comparison of innovative points between our work and literature to improve dielectric constant are shown in Table R7. The innovations of this work lie in:

(1) The rational molecular design and optimization of functional regulators;

(2) A systematic investigation into how the conformational structure of regulator molecules affects device performance, offering valuable guidance for establishing structure–performance relationships toward high-performance regulator design;

(3) The achievement of dominant dielectric regulation accompanied by subtle morphology optimization.

As evidenced by a review of relevant literature, previous studies have neither carried out molecular design of regulators nor examined the influence of regulator conformation on device performance. Our research exactly fills this void, which serves as a strong testament to the innovation of our work.

Table R7. Comparison of innovative points between our work and literature to improve dielectric constant.

Active layer	PCE (%)	Key Strategy
D18:L8-BO (*Joule*, 2025, 9, 102135)	19.23 (300 nm) 20.87 (100 nm)	• TBP was purchased from J&K Co. without implementing corresponding molecular design and conformation optimization<br>• **Dominantly morphology optimization** (RMS from 0.94 to 4.52 nm), accompanied by slight enhancement of dielectric constant (△ε_r = 0.09 ~ 0.19).
D18-Cl/BTP-C3F (*Energy Environ. Sci.*, 2025, 18, 4982–4995)	20.80	• **Molecular design of active layer materials**<br>• Improved morphology and dielectric properties, efficient charge extraction and reduced recombination losses
(*Solar RRL*, 2025, 9, e202500608)	Review	• **Molecular design of active layer materials** including backbone modification and side chains modification<br>•**The Addition of Additive or a Third Componen**t: ① Volatile liquid additives (fullerene systems); ② Non-volatile additives (analogous to the third component); ③ Third Component (Our previous work: *Adv. Mater.* 2024, 36, 2403294)
(*Chinese Journal of Chemistry*, 2021, 39, 381–390)	Review	• **Molecular design of active layer materials** including core modification and side chains modification
**D18:L8-BO (This work)**	**20.85 (100 nm) 19.56 (300 nm)**	• **Molecular design and optimization of regulators**<br>• **The influence of conformational structure of regulator molecules on device performance**<br>• **Dominantly dielectric regulation, accompanied by slight morphology optimization**

Comment 2: The authors achieved a power conversion efficiency as high as 20.85% but did not undergo third-party certification. This is a significant shortcoming for work with such high efficiency.

Reply 2: We agree with the reviewer that a third-party certification is needed. The champion device achieved a certified PCE of 20.38% following the calibration procedures of the South China National Center of Metrology (Figure R21, also referred to as Supplementary Fig. 21).

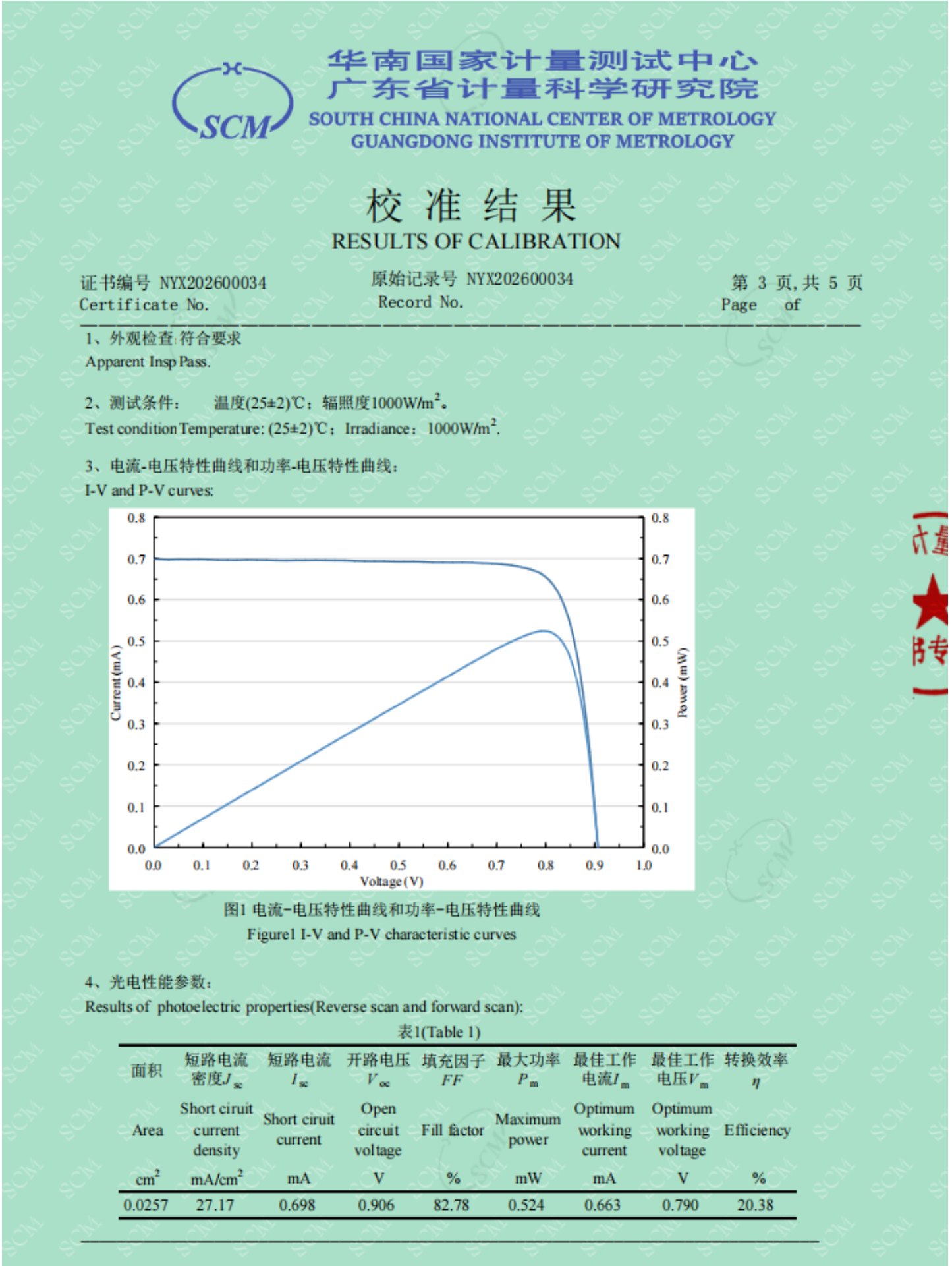

华南国家计量测试中心
广东省计量科学研究院
SOUTH CHINA NATIONAL CENTER OF METROLOGY
GUANGDONG INSTITUTE OF METROLOGY

校 准 结 果
RESULTS OF CALIBRATION

证书编号 NYX202600034 Certificate No. 原始记录号 NYX202600034 Record No. 第 3 页，共 5 页 Page of

1、外观检查:符合要求
Apparent Insp Pass.

2、测试条件： 温度(25±2)℃；辐照度1000W/m²。
Test condition Temperature: (25±2)℃; Irradiance: 1000W/m².

3、电流-电压特性曲线和功率-电压特性曲线：
I-V and P-V curves:

图1 电流-电压特性曲线和功率-电压特性曲线
Figure1 I-V and P-V characteristic curves

4、光电性能参数：
Results of photoelectric properties(Reverse scan and forward scan):

表1(Table 1)

面积 Area	短路电流密度 J_{sc} Short ciruit current density	短路电流 I_{sc} Short ciruit current	开路电压 V_{oc} Open circuit voltage	填充因子 FF Fill factor	最大功率 P_m Maximum power	最佳工作电流 I_m Optimum working current	最佳工作电压 V_m Optimum working voltage	转换效率 η Efficiency
cm^2	mA/cm^2	mA	V	%	mW	mA	V	%
0.0257	27.17	0.698	0.906	82.78	0.524	0.663	0.790	20.38

Figure S21. Certification of the photovoltaic efficiency of the D18:L8-BO device with the treatment of Dr-2 from South China National Center of Metrology.

Comment 3: The design rationale for Dr-1 and Dr-2 is not clearly explained. Dielectric constants can be modulated through various methods, such as incorporating non-conductive polymers, what are the basis of these newly synthesized molecules.

Reply 3: Thank you for the useful comment. The objective of the molecular design rationale is to enhance the dipole moment of dielectric regulators. The design rationale includes the incorporation of highly polarizable atoms or dipolar functional groups to increase local polarizability (eg. halogen atoms), the extension of π-conjugated backbones to improve charge delocalization and molecular polarizability (eg. S···F noncovalent conformational locks), and the introduction of high-permittivity component into the active layer (as dielectric regulator). Changes to the molecular structure of the active layer material exert a profound impact on device performance, with no guarantee of improved final efficiency, thus entailing considerable risks. Adding non-conductive polymers may negatively impact device performance, with most residues remaining inside the device and hard to eliminate. Consequently, the development of physical strategies that can boost the dielectric constant while preserving the chemical structures and electronic integrity of the active layer constitutes a more

controllable and facile approach. Therefore, guided by the aforementioned molecular design concept, we designed the dielectric regulator with high dipole moment to finely tune the dielectric constant of the active layer material. Corresponding descriptions have been added in page 4 of the revised manuscript.

Comment 4: It is vaguely mentioned that Dr-2 exhibits intramolecular F…S interactions, yet no crystal structure is provided. Moreover, would such weak intramolecular interactions remain intact under heating conditions?

Reply 4: We have successfully grown single crystals of Dr-2, and corresponding analysis has been added in revised manuscript (Supplementary Fig. 11 and Supplementary Table 1, also referred to as Figure R22 and Table R8) as follows: Intramolecular noncovalent interactions were investigated via single-crystal X-ray diffraction (SCXRD) measurements. The crystal structure of Dr-2 is presented in Supplementary Fig. 11, with the corresponding detailed crystallographic data summarized in Supplementary Table 1. Dr-2 crystal is isostructural belonging to the monoclinic system with space group $P2_1/_n$. At the equilibrium geometry, the intramolecular S···F distance was determined to be 2.85 Å, which is significantly shorter than the sum of the respective van der Waals radii for S and F (3.27 Å), confirming the existence of intramolecular S···F noncovalent conformational locks. The Dr-2 crystal forms a dimeric structure with two distinct stacking motifs via intermolecular π···π interactions, where the corresponding π···π stacking distances (dπ···π) are 3.59 Å and 3.47 Å for the two stacking modes, respectively.

To clarify the conformational stability of Dr-2 under fabrication temperatures (~373 K), a potential energy surface was obtained at the B3LYP-D3/6-31+G(d) level (Figure R23). The results confirm a thermodynamically stable conformation at a torsion angle of ~25°, and the calculated conformational conversion barrier is 6.92 kcal/mol. It has been reported that thermal energy near room temperature is approximately $2kT$ (where k is the Boltzmann constant and T is absolute temperature) (*J. Phys. Chem. Lett.*, 2016. *7*, 3609). Intramolecular weak interactions remain stable at a given temperature if their conformational conversion barrier exceeds the corresponding thermal energy (*Nat. Commun.*, 2023. *14*, 2500). For Dr-2, this barrier (6.92 kcal/mol) is much higher than the thermal energy (~1.5 kcal/mol) at the maximum fabrication temperature (~373 K). Thus, the intramolecular F…S interactions remain intact and stable under device fabrication conditions.

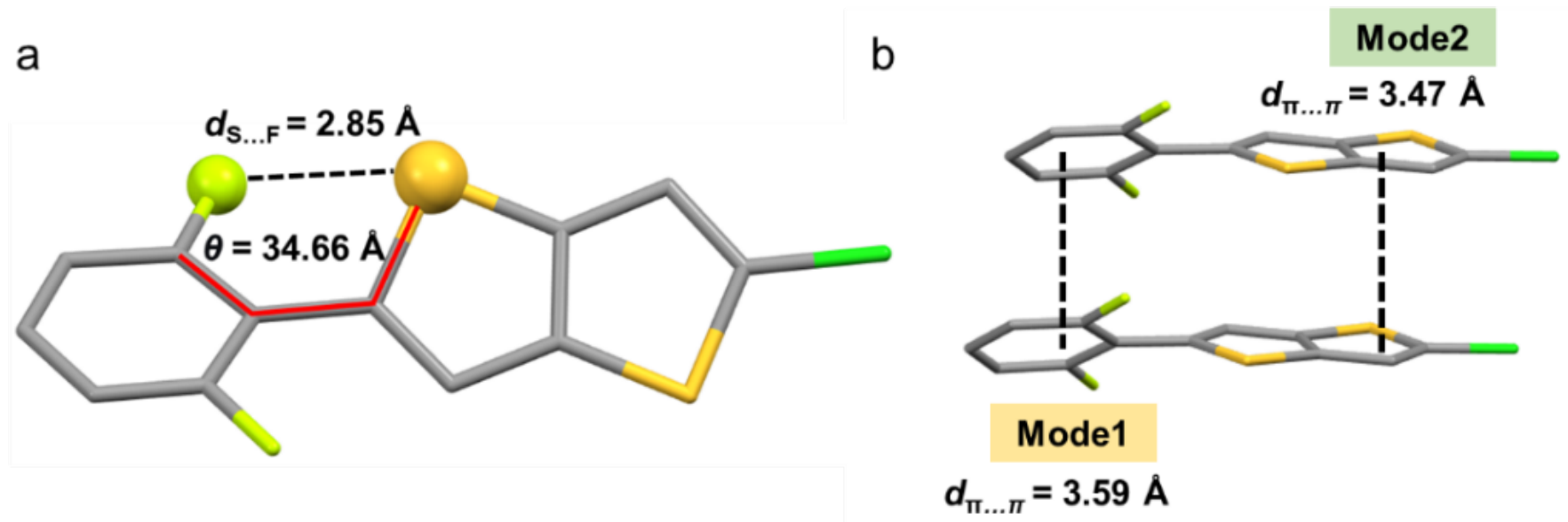

Figure R22. Single-crystal structures and molecular packing of Dr-2.

Table R8. Crystallographic data for Dr-2.

Informations	Dr-2
CCDC Deposition Number	2526058
Empirical Formula	$C_{12}H_5ClF_2S_2$
Formula weight	286.73
Temperature/K	193
Crystal system	monoclinic
Space group	$P2_1/n$
a/Å	3.81710(10)
b/Å	23.0030(6)
c/Å	12.7856(3)
α/°	90
β/°	92.5130(10)
γ/°	90
Volume/Å^3	1121.56(5)
Z	4
ρ_{calc}/(g/cm^3)	1.698
μ/mm^{-1}	6.512
F(000)	576.0
Crystal size/mm^3	0.13 × 0.11 × 0.08
Radiation	CuKα (λ = 1.54178)
2θ range for data collection/°	7.686 to 148.646
Index ranges	$-4 \le h \le 3$, $-28 \le k \le 27$, $-15 \le l \le 15$
Reflections collected	11363
Independent reflections	2272 [R_{int} = 0.0316, R_{sigma} = 0.0241]
Data/restraints/parameters	2272/0/154
Goodness-of-fit on F^2	1.051
Final R indexes [I>=2σ (I)]	R_1 = 0.0242, wR_2 = 0.0656
Final R indexes [all data]	R_1 = 0.0260, wR_2 = 0.0668
Largest diff. peak/hole/e Å^{-3}	0.28/-0.24

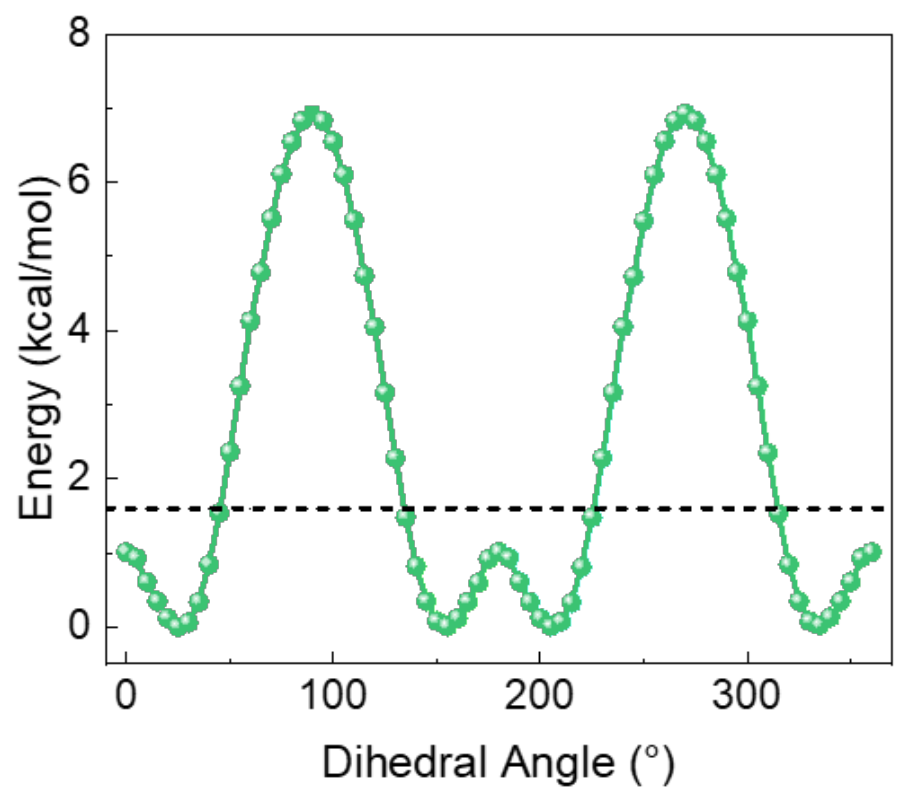

Figure R23. Potential energy surface scan of the Dr-2.

Comment 5: As the most critical evidence for intermolecular interactions, FTIR shows shifts of only 2–3 wavenumbers, which falls within the margin of error. However, the authors exaggerated the significance of these changes.

Reply 5: We thank the reviewer for pointing out the potential impact of instrumental error on the FTIR red shift results, which is a critical consideration for spectral analysis. To verify the reliability of the observed redshift, the FTIR test under each condition was performed in three replicate measurements with strict control of experimental variables (e.g., sample preparation, test temperature, spectral resolution). The red shift was a reproducible observation in all three independent measurements, with no significant deviation among replicate data (Supplementary Fig. 16, also referred to as Figure R14). This consistency confirms that the observed spectral shift is a genuine spectral response to the intermolecular interaction rather than random instrumental error. We have added this key experimental detail in the revised manuscript to make the data more rigorous.

To verify the intermolecular interactions, we employed two-dimensional (2D) liquid-state nuclear magnetic resonance (NMR) spectroscopy with higher resolution for characterization. Corresponding analysis has been added in revised manuscript (Figure 1c, also referred to as Figure R15) as follows: Owing to the nuclear Overhauser effect, NOESY spectra in 2D NMR furnish information about protons that are ≤ 5 Å apart in space. In the 2D 1H−1H NOESY spectra of the L8-BO:Dr-2 solution, a distinct cross-peak between the 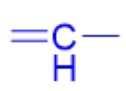 protons of Dr-2 (6.98 ppm) and the 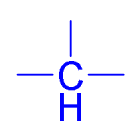 protons of L8-BO (3.18 ppm) were clearly detected, which can verify the possibility of intermolecular interaction between L8-BO and Dr-2.

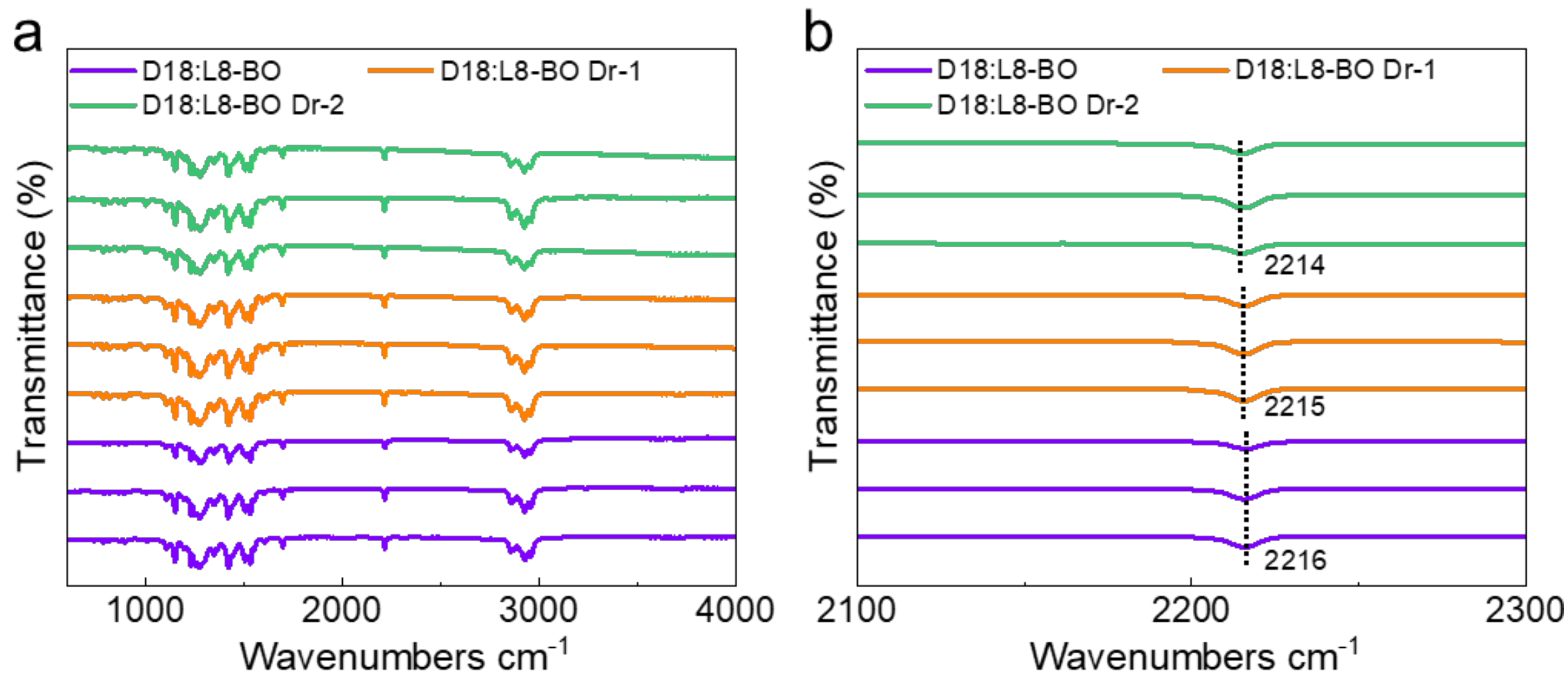

Figure R14. FTIR of pure and blend films with/without regulator materials.

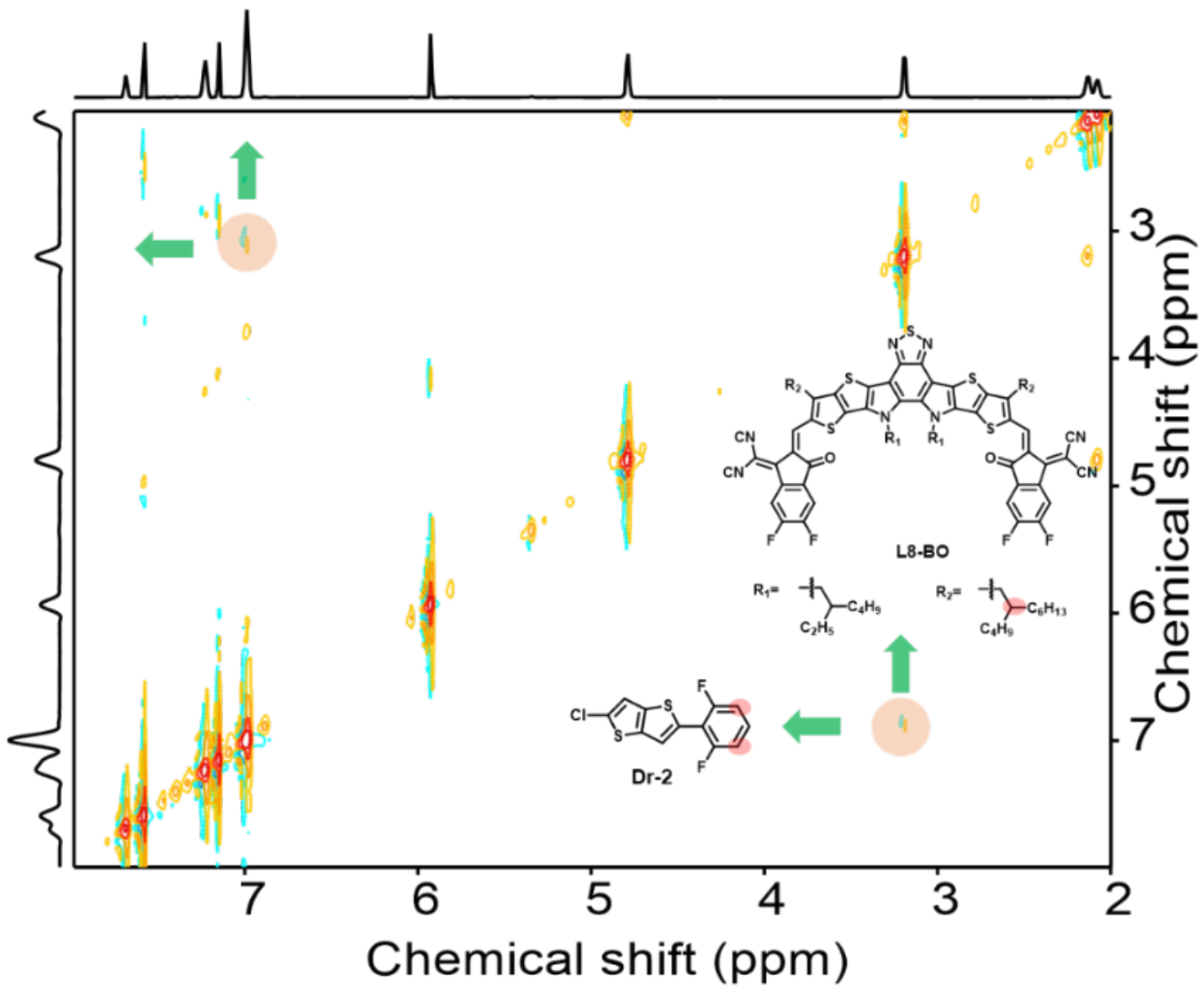

Figure R15. 2D ^{1}H-^{1}H NMR spectra of L8-BO:Dr-2 in deuterated tetrachloroethane (d-TCE) solution.

Comment 6: Morphological analyses, including GIWAXS, do not reveal significant changes, yet the authors claim that "the dielectric regulator effectively controls molecular packing."

Reply 6: We thank the reviewer for this insightful comment regarding the relationship between GIWAXS results and our claim on molecular packing regulation. We would like to clarify that the GIWAXS results show that the introduction of dielectric regulator mainly changes the π-π stacking distance and coherence length of the blend. In our study, the GIWAXS patterns indeed show only marginal changes, indicating that the overall crystalline framework of the bulk heterojunction is largely preserved upon incorporation of the dielectric regulator. To avoid overinterpretation, we have revised the manuscript to "the dielectric regulator can control molecular packing to some extent".

<u>Comment 7:</u> Figure 5f illustrates the process of modulating dielectric constants via additives, but it lacks robust evidence. Furthermore, during the annealing process to remove additives, could the orientation of molecules in the active layer not change?

<u>Reply 7:</u> First, for the D18:L8-BO system without any regulators, the active layer retains its pristine molecular arrangement, as illustrated in process ① (Figure 5f , also referred to as Figure R24). With the addition of the regulator, intermolecular interactions between L8-BO and regulator (supported by 2D NMR data) promote ordered molecular packing, resulting in more aligned dipoles. The ordered arrangement is verified by GIWAXS, which ultimately enhances the uniformity of molecular orientation. Upon optimization of molecular orientation, the dipole alignment also becomes more uniform, leading to an increase in the dielectric constant (processs ②).

During thermal annealing, the regulator molecules sublime out of the thin film, and the high resistance within the solid film leads to negligible changes in the molecular orientation of the donor and acceptor (processs ③)

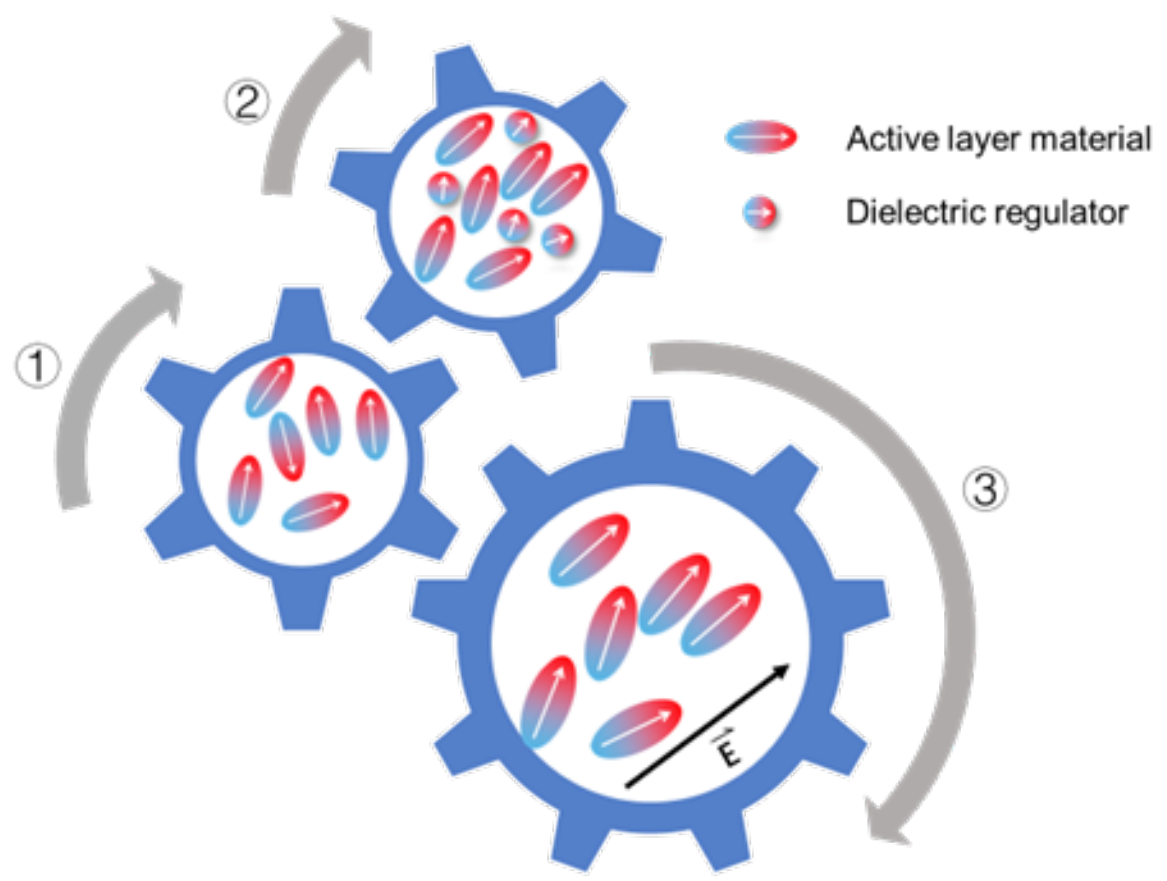

Figure R24. Schematic diagram of dielectric regulator on dielectric constant. ① Active layer without dielectric regulator, ② active layer with dielectric regulator, ③ after annealing of ②.

To investigate the effect of annealing on molecular orientation, we compared the GIWAXS results before and after thermal annealing. Corresponding analysis has been added in revised manuscript (Supplementary Figs. 54-55, also referred to as Figure R25-26) as follows: Prior to thermal annealing (Supplementary Figs. 54-55), the dominant π-π stacking diffraction remains primarily in the out-of-plane direction for both conditions, indicating that the preferential face-on molecular orientation is well preserved. After thermal annealing (Fig. 4b-c), no noticeable redistribution of diffraction peak position between the in-plane and out-of-plane directions is observed, suggesting that annealing does not induce a reorientation of the molecules. Instead, the slightly enhanced diffraction intensity after annealing can be attributed to improved crystallinity following regulator removal, rather than a change in molecular orientation. Above results indicate that the annealing treatment mainly removes residual

dielectric regulator and optimizes the molecular crystallinity without significantly altering the molecular orientation of the active layer (Process ③).

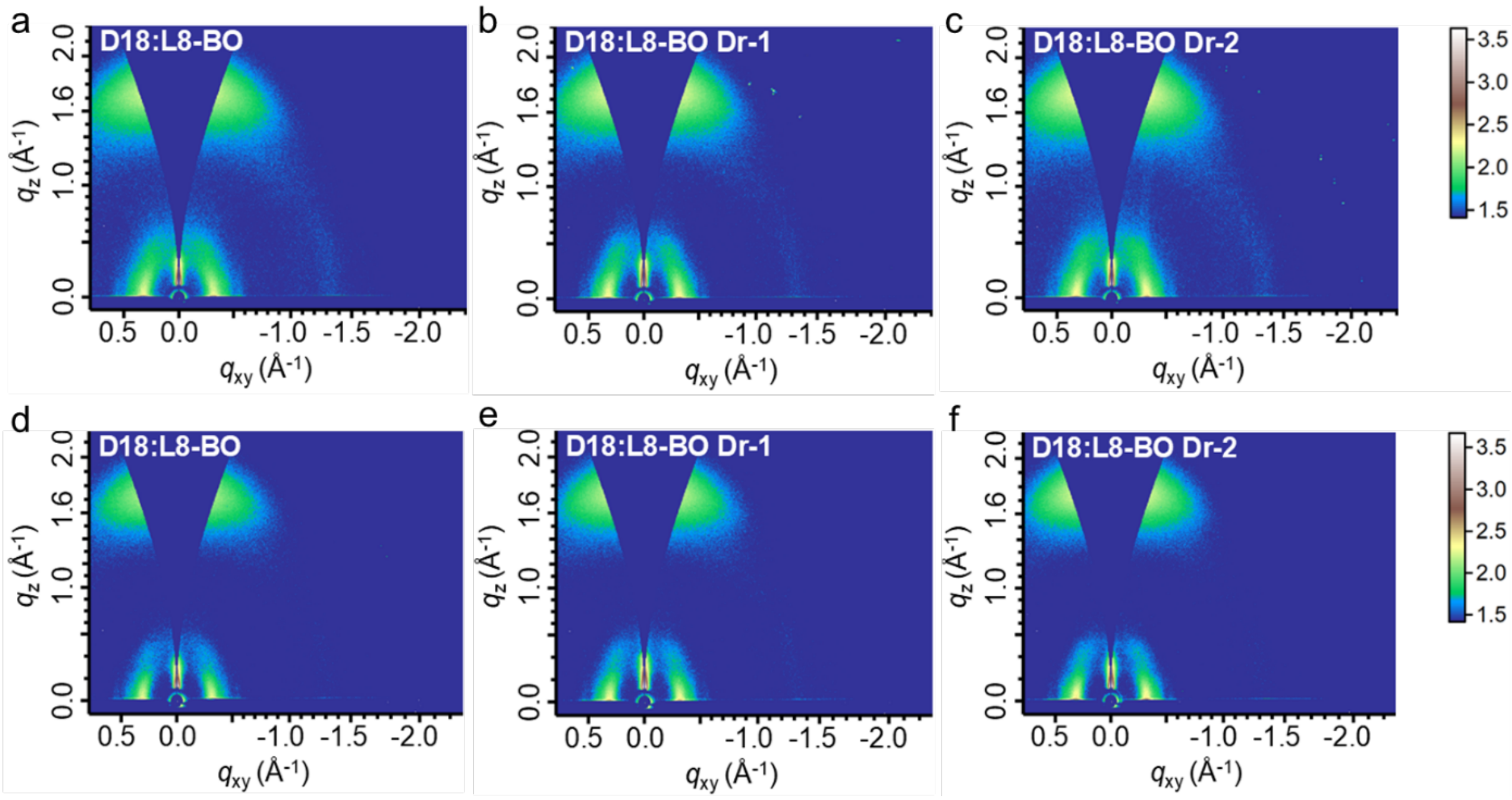

Figure R25. 2D GIWAXS patterns of blend films **a-c** before and **d-f** after thermal annealing.

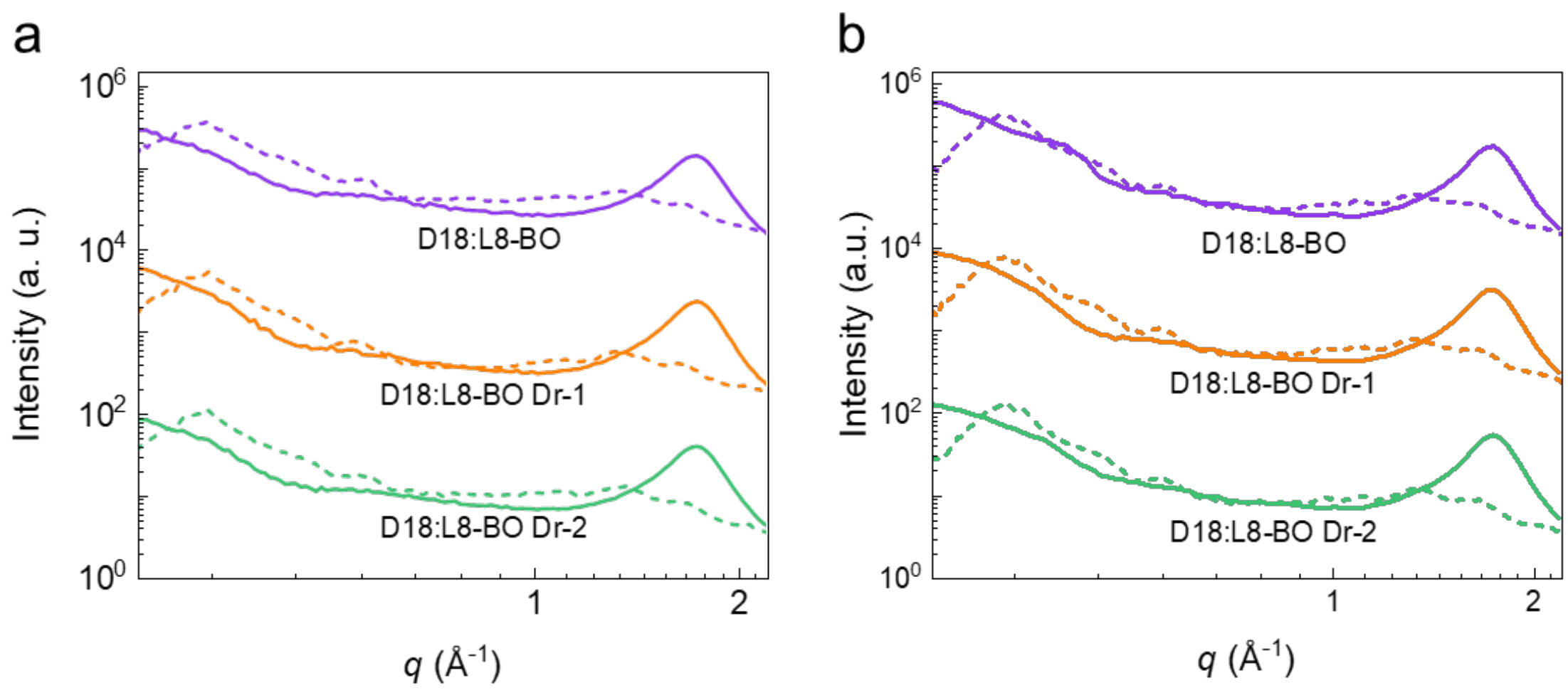

Figure R26. OOP (solid line) and IP (dotted line) extracted line-cut profiles of blend films **a** before and **b** after thermal annealing.
