# Supplementary material for: High performance organic solar cell enabled by manipulating the exciton dissociation and charge transfer via dielectric engineering
Source: Nat Commun. 2026 Jun 16;17:7649. doi: 10.1038/s41467-026-74112-x (PMC13434753; doi:10.1038/s41467-026-74112-x)
Supplement: Supplementary file 4 [file 41467_2026_74112_MOESM4_ESM.zip › date/Supplementary Figure/Supplementary Figure 12.pdf]

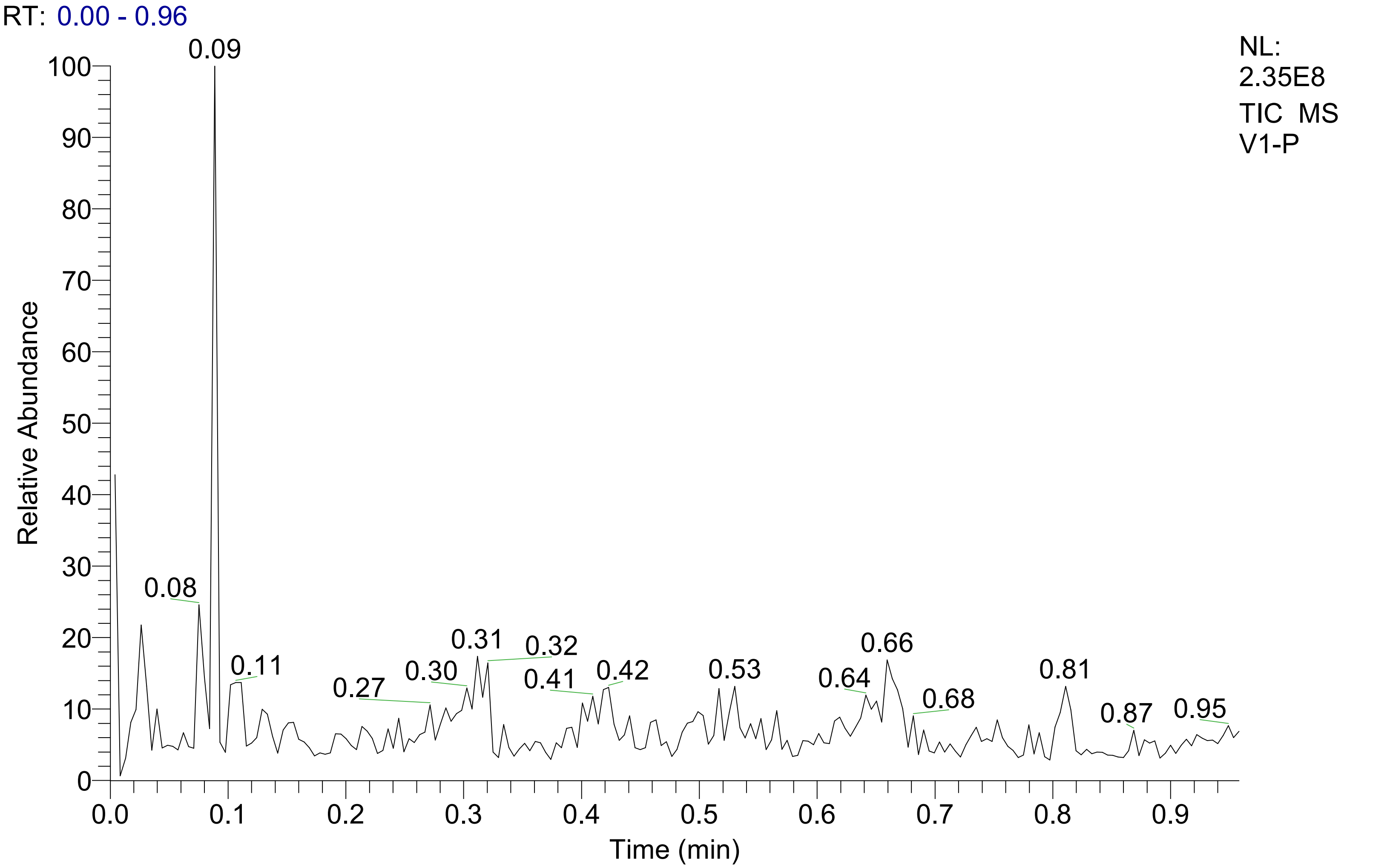
RT: 0.00 - 0.96
NL: 2.35E8
TIC MS
V1-P
Relative Abundance
Time (min)
0.09
0.08
0.11
0.27
0.30
0.31
0.32
0.41
0.42
0.53
0.64
0.66
0.68
0.81
0.87
0.95

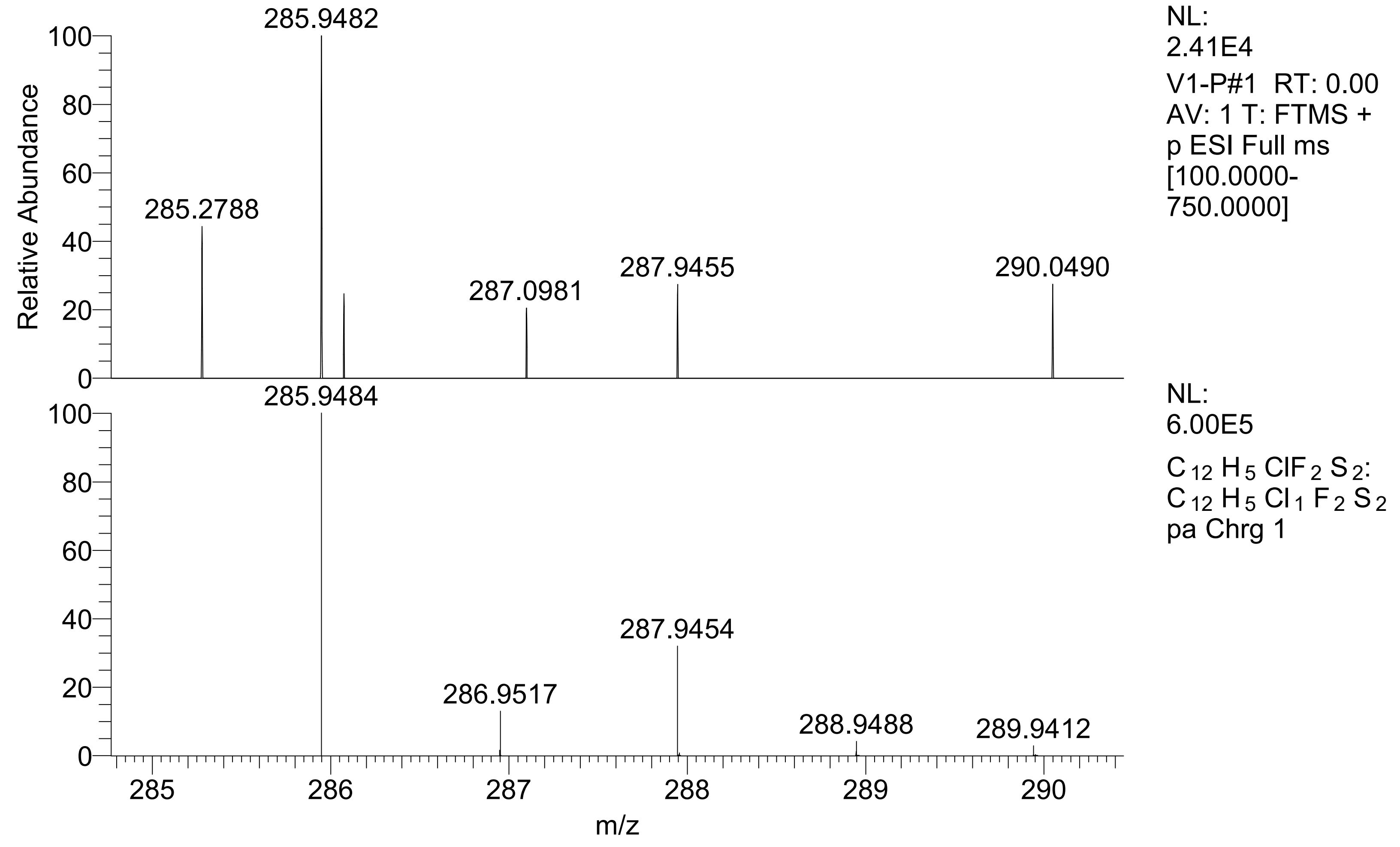
NL: 2.41E4
V1-P#1 RT: 0.00
AV: 1 T: FTMS + p ESI Full ms [100.0000-750.0000]
285.9482
285.2788
287.0981
287.9455
290.0490
NL: 6.00E5
C 12 H 5 ClF 2 S 2: C 12 H 5 Cl 1 F 2 S 2
pa Chrg 1
285.9484
287.9454
286.9517
288.9488
289.9412
Relative Abundance
m/z
