# Supplementary material for: High performance organic solar cell enabled by manipulating the exciton dissociation and charge transfer via dielectric engineering
Source: Nat Commun. 2026 Jun 16;17:7649. doi: 10.1038/s41467-026-74112-x (PMC13434753; doi:10.1038/s41467-026-74112-x)
Supplement: Supplementary file 4 [file 41467_2026_74112_MOESM4_ESM.zip › date/Supplementary Figure/Supplementary Figure 13.pdf]

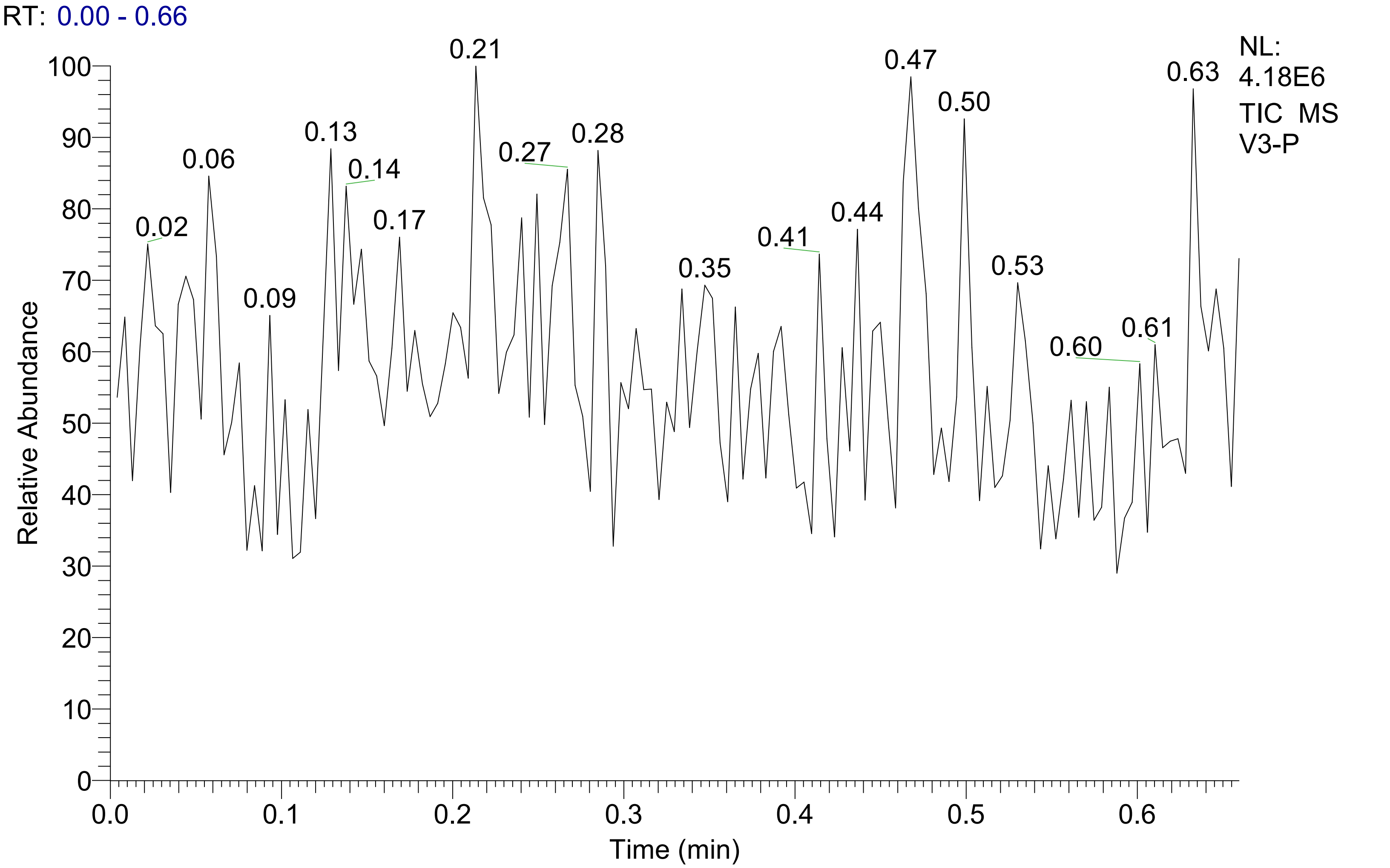
RT: 0.00 - 0.66
NL:
4.18E6
TIC MS
V3-P
0.02
0.06
0.09
0.13
0.14
0.17
0.21
0.27
0.28
0.35
0.41
0.44
0.47
0.50
0.53
0.60
0.61
0.63
Relative Abundance
Time (min)

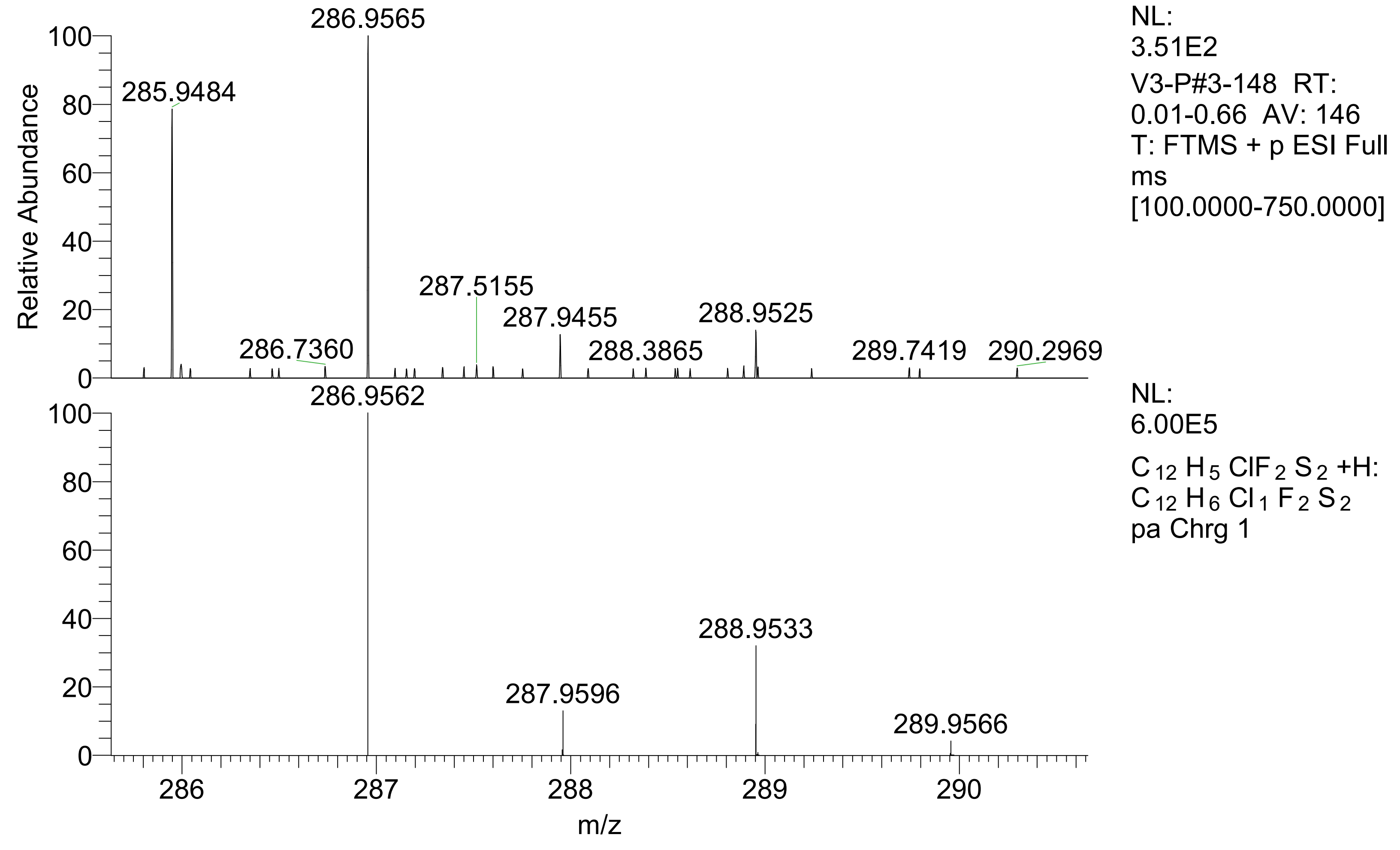
NL:
3.51E2
V3-P#3-148 RT:
0.01-0.66 AV: 146
T: FTMS + p ESI Full
ms
[100.0000-750.0000]
285.9484
286.9565
286.7360
287.5155
287.9455
288.3865
288.9525
289.7419
290.2969
NL:
6.00E5
C 12 H 5 ClF 2 S 2 +H:
C 12 H 6 Cl 1 F 2 S 2
pa Chrg 1
286.9562
287.9596
288.9533
289.9566
Relative Abundance
m/z
